# New imidazole-2-thiones linked to acenaphythylenone as dual DNA intercalators and topoisomerase II inhibitors: structural optimization, docking, and apoptosis studies

**DOI:** 10.1080/14756366.2024.2311818

**Published:** 2024-03-15

**Authors:** Asmaa H. Mohamed, Mohammed B. Alshammari, Ashraf A. Aly, Kamal U. Sadek, Akil Ahmad, Eman A. Aziz, Amira F. El-Yazbi, Eman J. El-Agroudy, Marwa E. Abdelaziz

**Affiliations:** a Chemistry Department, Faculty of Science, Minia University, El-Minia, Egypt; b Chemistry Department, College of Sciences and Humanities, Prince Sattam Bin Abdulaziz University, Al-Kharij, Saudi Arabia; c Department of Pharmaceutical Analytical Chemistry, Faculty of Pharmacy, Alexandria University, Alexandria, Egypt; d Department of Pharmaceutical Chemistry, Faculty of Pharmacy, Alexandria University, Alexandria, Egypt

**Keywords:** New imidazole-2-thiones, DNA intercalators, topoisomerase II inhibitor, doxorubicin, dual anticancer activity

## Abstract

In this article, a new series of 2-((3,5-disubstituted-2-thioxo-imidazol-1-yl)imino)acenaphthylen-1(2*H*)-ones were synthesized. Imidazole-2-thione with acenaphthylen-one gave a hybrid scaffold that integrated key structural elements essential for DNA damage *via* direct DNA intercalation and inhibition of the topoisomerase II enzyme. All the synthesized compounds were screened to detect their DNA damage using a terbium fluorescent probe. Results demonstrated that 4-phenyl-imidazoles **5b** and **5e** in addition to 4-(4-chlorophenyl)imidazoles **5h** and **5j** would induce detectable potent damage in ctDNA. The four most potent compounds as DNA intercalators were further evaluated for their antiproliferative activity against HepG2, MCF-7 and HCT-116 utilizing the MTT assay. The highest anticancer activity was recorded with compounds **5b** and **5h** against the breast cancer cell line MCF-7 which were 1.5- and 3- folds more active than **doxorubicin**, respectively. Therefore, imidazole-2-thione tethered acenaphthylenone derivatives can be considered as promising scaffold for the development of effective dual DNA intercalators and topoisomerase II inhibitors.

## Introduction

Cancer is regarded as the second leading cause of death worldwide in the 21^st^ century^1^. The synthesis of new potent and effective anticancer agents is of constant and growing interest in cancer treatment. As for the discovery of new drug candidates, researchers are committed to take a leap to discover new chemotherapeutic agents that interfere with DNA^2^. Numerous medications including **doxorubicin** and **mitoxantrone** are FDA-approved DNA intercalators; nevertheless, during clinical use, various adverse effects, including cardiotoxicity and the development of secondary malignancies, have been reported. Therefore, one of the main goals of modern medicinal chemistry is to create new safer DNA intercalators for the treatment of cancer. The role of DNA-interfering compounds has different approaches to exert their anticancer activities involving intercalating with the DNA and/or enzymes necessary for relevant DNA functions giving rise to a cellular response change which leads to cell death^3^. Typical DNA intercalators are compounds characterized by their ability to be inserted perpendicularly into DNA to form a DNA-intercalator complex stabilized by different hydrophobic stackings without forming covalent bonds^4^. Polycyclic aromatic hydrocarbons have become one of the key molecular objectives in anticancer activity owing to their ability to be inserted between stacked base pairs of DNA^5^.

DNA topoisomerase enzyme plays crucial roles in employing the topology of DNA and controls many vital functions in the cell cycle^6^. Topoisomerase enzyme is present in both mammalian and bacterial cells which makes it a conspicuous target as antibacterial drugs and as first-line anticancer agents^7^. Inhibition of topoisomerase enzyme has been the focus of anticancer drug discovery. In eukaryotic cells and according to its mechanism of action, topoisomerase is classified into topoisomerase I (Topo I) and II (Topo II)^6^^,^^8^. It has been found that mammalian Topo II worked through two different modes of action and thus categorised into: (a) topo II poisons and (b) topo II catalytic inhibitors;^9^ (a) topo II poisons irreversibly bind to DNA-protein complex *via* covalent bonds and induce DNA double strand breaks (DSBs) which finally results in an apoptotic fate^2^^,^^10^. The second class is (b) catalytic inhibitors which act by binding non-covalently to the Topo II protein complex, preventing Topo II from binding to DNA or to ATP^11^. Many well-known anticancer drugs act as topo II poisons *via* inter-calation with DNA such as: **doxorubicin** and **mitoxantrone**. Although these drugs have worked successfully as anticancer agents on a clinical basis, their use has been limited due to some toxicity and resistance consequences as mentioned^3^^,^^12^.

**Figure 1.**
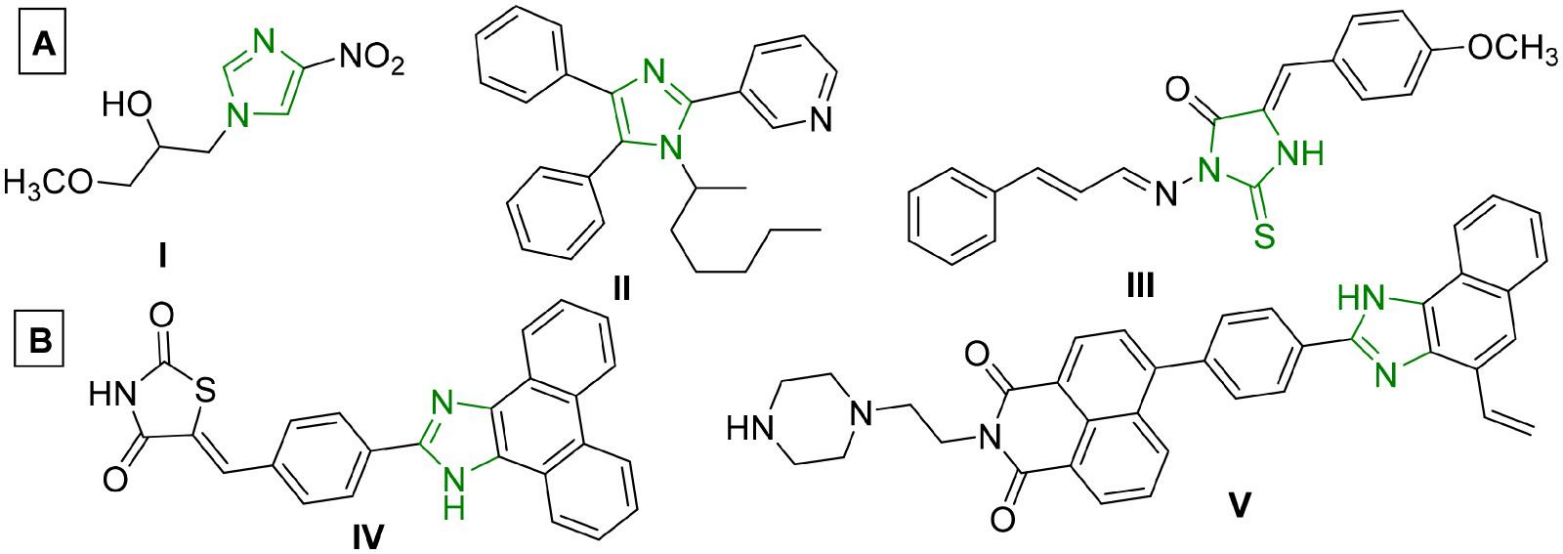
(**A**) Reported imidazole-containing drugs **I-III** as anticancer agents^21–23^, (**B**) Reported drugs bearing imidazole- polynuclear scaffold hybrids **IV-V** as anticancer agents^5^^,^^24^^,^^25^.

Imidazoles are an important class of heterocyclic molecules that are widely used to design medicinally important structural components with various pharmacological applications^13^^,^^14^. Various drugs bearing the imidazole moiety have been reported to have anticancer activity^5^^,^^15^^,^^16^. Imidazole derivatives have also been proven to bind with DNA^17^, and exhibit good anticancer activity against MCF-7, HepG-2 and HCT-116 cell lines^18^^,^^19^. Some imidazole derivatives displayed strong DNA intercalating activity^19^^,^^20^, while other reported compounds demonstrated cytotoxicity at micromolar doses, as seen in **I-III** (Figure 1A)^21–23^^,^^26^^,^^27^. Moreover, acenaphthequinone (acenaphthylene-1,2-dione) is a quinone derivative, widely used as significant starting material for the synthesis of various heterocycles, and as a key intermediate in organic synthesis for different reactions and pharmaceutical applications^28–30^. Some derivatives of acenaphthequinone have been used as biologically active compounds, dyes, pharmaceuticals, drugs, pesticides, and therapeutic agents^31–33^. It was reported that tethering different poly aromatic chromophores with imidazole exhibited significant anticancer activity as in **IV-V** (Figure 1B)^5^^,^^24^^,^^25^.

## Rational design

Literature has discovered three key pharmacophoric components shared by the DNA intercalator and the Topo II toxin **doxorubicin**. The first need is a polynuclear aromatic chromophore that can be placed in between DNA base pairs. Also, a cationic site that may attach to the phosphate group of DNA at physiological pH is the second structural feature. This might be a heterocyclic group that is nitrogen-containing or nitrogen-linked. Moreover, to fill DNA minor grooves, a groove-binding moiety (non-intercalator) is needed as the third component^34^. In addition, as reported, acenaphthoquinone-imidazole hybrids increased overall anticancer activity and yielded promising anticancer agents as topo II inhibitors^24^. Owing to the previously mentioned findings, the current study was carried out as a continuation of our earlier research^35^^,^^36^ and to discover possible ligands with strong anticancer activity and consequently anti-topoisomerase II activity^37^ along with a better safety profile than **doxorubicin**.

**Figure 2.**
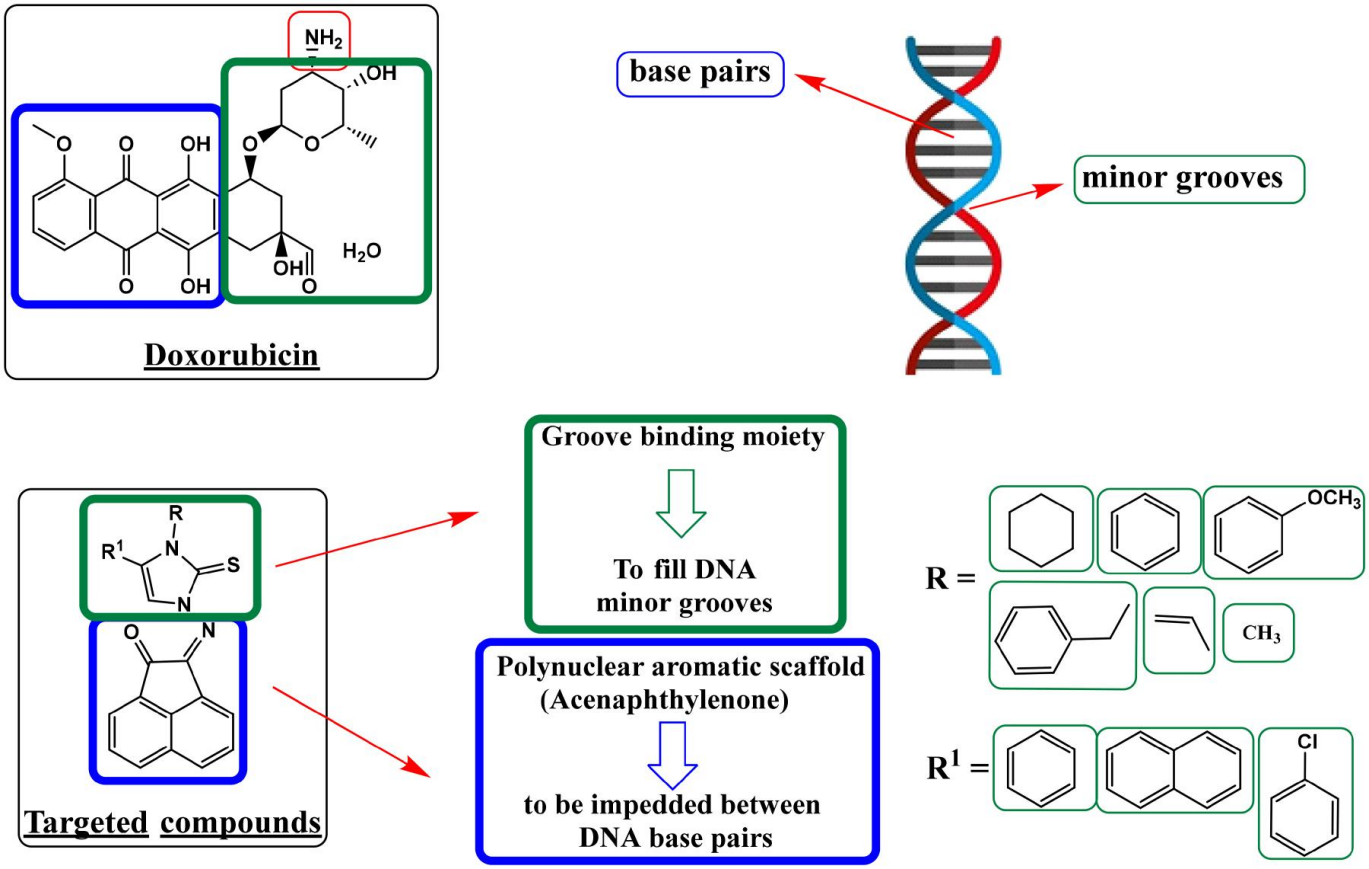
Work approach describes a hypothesis for designing targeted compounds based on structure features required for DNA damage through double helix intercalation and topoisomerase II inhibition, as in standard DNA intercalator **Doxorubicin**.

In the conducted study, series of 2-((3,5-disubstituted-2-thioxo-imidazol-1-yl)imino)acenaphthylen-1(2*H*)-ones were synthesized. The design of newly synthesized compounds took in consideration the hybridization of two structural features for DNA intercalation, acenaphthylenone (the polynuclear aromatic scaffold) and imidazoline-thione (the groove-binding moiety). These hybrids may significantly improve the anticancer activity by dual DNA intercalation and Topo II inhibition (Figure 2). Therefore, newly synthesized compounds were tested for their DNA damage and their antiproliferative effect. Additionally, topoisomerase inhibition assessment was carried out, and molecular docking studies were employed for analysing the findings. Pharmacokinetic profiles and *in-silico* predictions of physiochemical attributes were also performed.

## Results and discussion

### Chemistry

#### Preparation of N-substituted-2-(2-oxoacenaphthylen-1(2H)-ylidene)-hydrazinecarbothioamide derivatives 3a-f

**Scheme 1.**
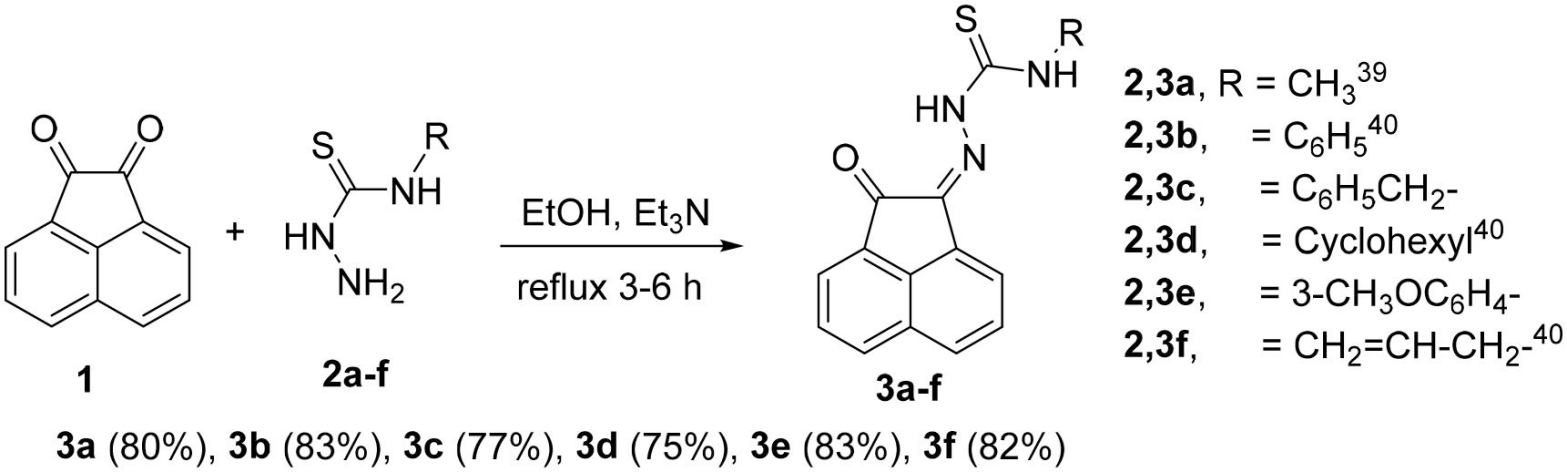
Synthesis of *N-*substituted-2-(2-oxoacenaphthylen-1(2*H*)-ylidene)hydrazine-carbothioamide derivatives **3a-f**.

Condensation of thiosemicarbazide derivatives **2a-f** with acenaphthalene-1,2-dione (**1**) in refluxing ethanol and in the presence of triethylamine (Et_3_N), gave the corresponding *N*-substituted-2-(2-oxoacenaphthylen-1(2*H*)-ylidene)hydrazinecarbothioamide derivatives **3a-f**^38^^,^^39^ (Scheme 1).

#### Synthesis of imidazole-2-thiones 5a-n

**Scheme 2.**
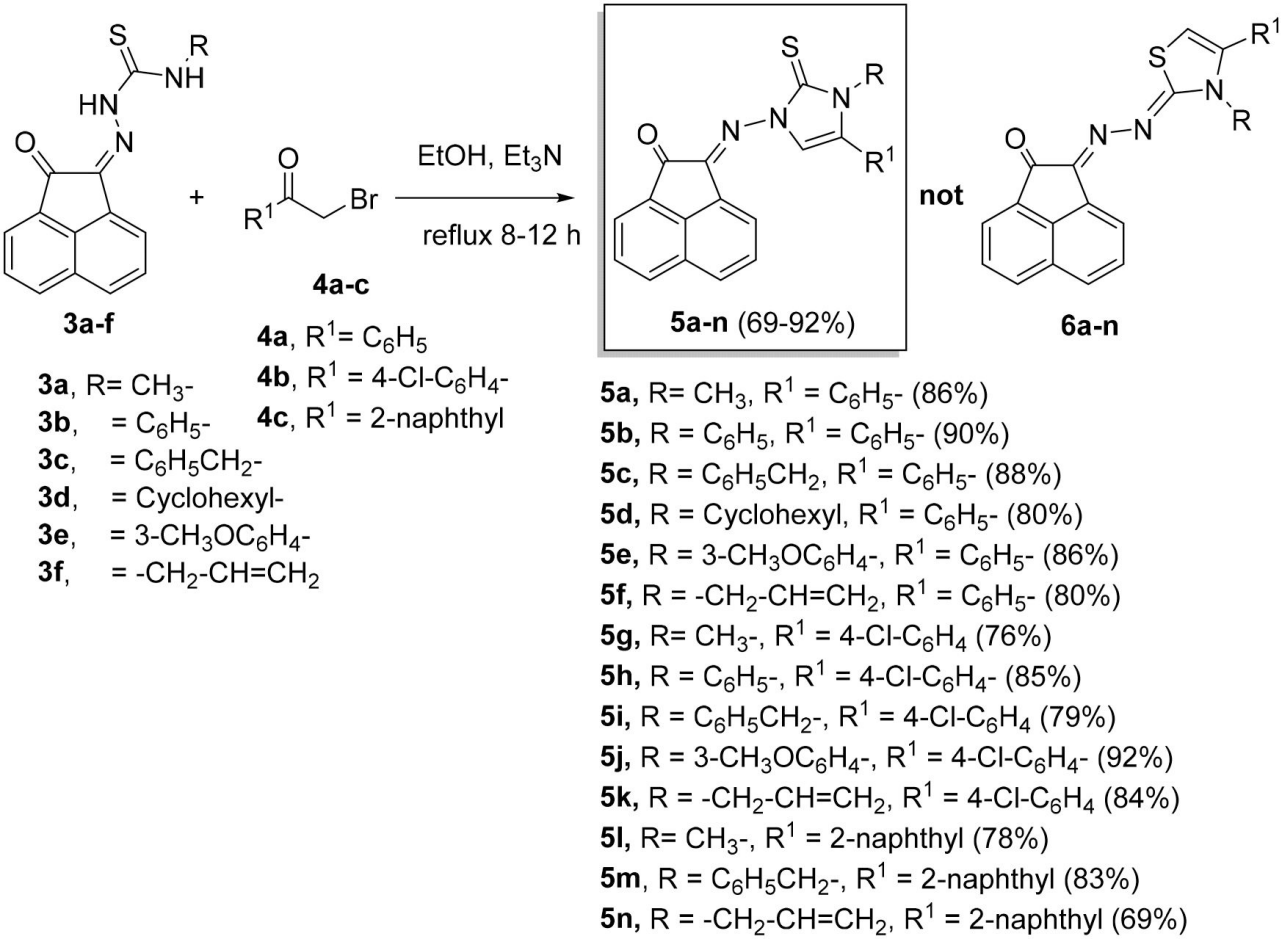
Synthesis of imidazole-2-thiones **5a-n**.

Reaction of compounds **3a-f** with 2-bromo-1-substituted-ethan-1-ones **4a-c** in ethanol and catalyzed by triethylamine (Et_3_N), produced 2-((3,5-disubstituted-2-thioxo-2,3-dihydro-1*H*-imidazol-1-yl)imino)acenaphthylen-1(*2H*)-ones **5a-n** in 69%–92% yields (Scheme 2).

The structure of the newly prepared derivatives of *N*-substituted-2-(2-oxoacenaphthylen-1(2*H*)-ylidene)hydrazinecarbothioamides **3a-f** was proved by IR, mass and NMR spectra in addition to elemental analysis. As for example, the structure of compound **3c** was identified as (*Z*)*-N*-benzyl-2-(2-oxoacenaphthylen-1(2*H*)-ylidene)hydrazine-1-carbothioamide. IR spectrum showed absorptions at *ν* = 3314 (for ΝΗ str), 1607 (for C = N str), and 1023 cm^−1^ (for C = S str). The ^1^H NMR spectrum revealed two singlets at *δ*_H_ = 12.70 and 9.94 ppm for the two protons of the NH groups, whereas the benzyl-CH_2_ protons were resonated at *δ*_H_ = 4.94 ppm (*J* = 6.2 Hz). The ^13^C NMR spectrum showed the C = O, C = S and CH_2_- carbon signals at *δ_C_* =188.5, 178.0 and 47.3 ppm, respectively (see the experimental).

**Figure 3.**
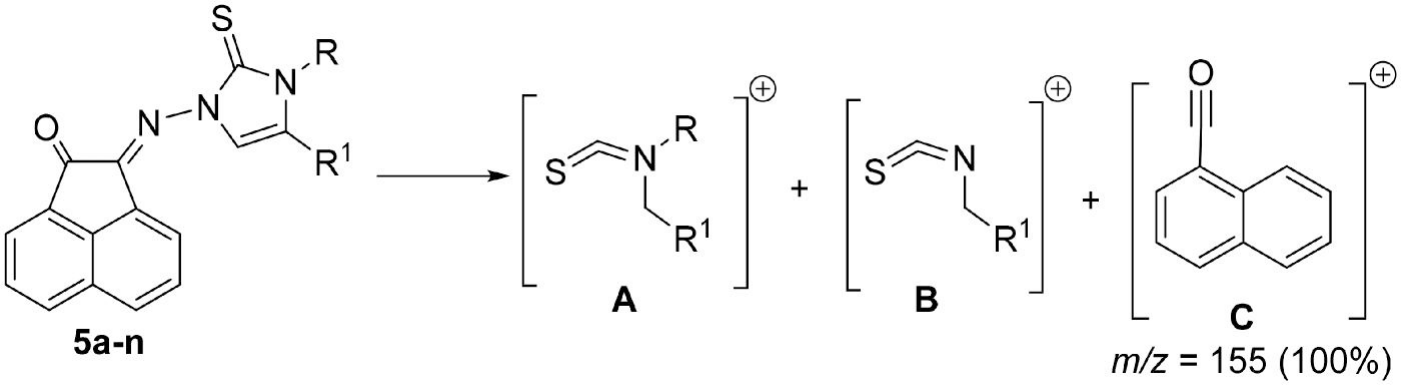
Fragmentation patterns **A**-**C** for mass spectra of compounds **5a-n**.

Also IR, NMR, mass spectra in addition to elemental analysis were used to elucidate the structure of the obtained imidazole-2-thiones **5a-n**. Generally, in the most mass spectra of compounds **5a-n**, two distinctive fragmentation patterns **A** and **B** were noted (Figure 3). Mass spectra of most compounds also showed permanent fragmentation patterns of **C** at *m/z* = 155 (30%–50%) and 154 (100%) as shown in Figure 3.

In ^1^H NMR spectra of **5a-n**, the disappearance of the two NH signals indicating their removal during the reaction pathway. The appearance of the C = S carbon signals at nearly *δ_C_* = 175.8–178.4 ppm in the ^13^C NMR spectra of the formed products, excluded the formation of thiazoles **6a-n** (Scheme 2).

**Figure 4.**
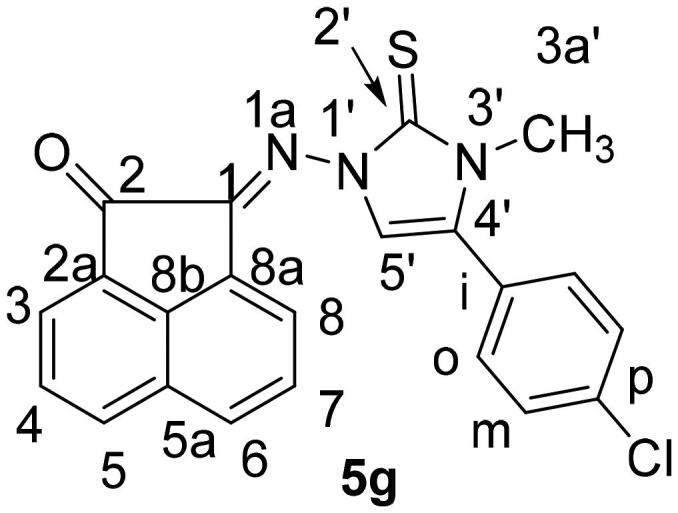
Distinctive carbons of compound **5 g**.

It is noteworthy that the expected thiazole derivatives **6a-n** were not obtained. NMR spectra proved the imidazole structure of **5a-n**. As for example, the ^1^H NMR spectrum of **5c** revealed the CH_2_-Ph and the imidazole-H-5 as two singlets at *δ_H_* = 5.35 and 6.94 ppm, respectively. The ^13^C NMR spectrum showed the CH_2_-Ph and imidazole-CH-5′ at *δ_C_* = 49.4 and 104.0 ppm, respectively. The ^1^H NMR spectrum of compound **5f**, as another example, showed some distinctive protons for aromatic protons as four double-doublets at *δ_H _*= 8.25 (1H, *J* = 8.3, 1.2 Hz), 8.03 (1H, *J* = 8.0, 1.2 Hz) and 7.78 ppm (2H, *J* = 7.8, 1.2 Hz). In addition, two multiplets appeared at *δ_H_* = 7.59–7.49 (2H) and 7.33–7.26 ppm (5H) for the remaining aromatic protons. The imidazole-H-5′ appeared as a singlet at *δ_H_* = 6.62, whereas the allyl protons resonated as a multiplet at *δ_H_* = 5.80–5.75 ppm for the allyl-CH = proton. In addition, two double-doublets appeared at *δ_H_* = 4.97 (*J* = 7.5, 1.2 Hz) and 4.79 ppm (*J* = 7.5, 1.2 Hz) for allyl-CH_2_= protons. Another double-triplet for allyl-CH_2_ protons appeared at *δ_H_* = 4.47 ppm (*J* = 7.5, 1.2 Hz). In ^13^C NMR spectrum of **5f**, the allyl-CH_2,_ allyl-CH_2_ = and allyl-CH = protons resonated at *δ_C _*= 48.6, 104.5 and 129.8 ppm, respectively. The C = O, C = S and *exc-*C = N carbon signals resonated in the ^13^C NMR spectrum at *δ_C_* = 189.1, 175.8 and 146.6 ppm, respectively. The ^1^H NMR spectrum of **5g** (Figure 4) revealed the six aromatic of acenaphythylenone as four doublets; each for one proton, at *δ_H_* = 8.57 (*J* = 6.9 Hz, H-5), 8.28 (*J* = 8.2 Hz, H-6), 8.06 (*J* = 8.4 Hz, H-3) and 8.00 ppm (*J* = 7.0 Hz, H-8). In addition, two double-doublets, each for one proton, were also resonated at *δ_H_* = 7.82 (*J* = 7.9, 7.4 Hz, H-7) and 7.78 ppm (*J* = 8.1, 7.4 Hz, H-4). Whereas, the di-substituted phenyl protons appeared at *δ_H_* = 7.66–7.62 ppm (4H). Imdazole-CH-5 (C-5′) and H-3a’ protons resonated as two singlets at *δ_H_* = 6.93 and 3.63 ppm, respectively. The ^13^C NMR spectrum of **5g** showed the carbon signals of *N*-CH_3_ and imidazole-CH-5 at *δ_C_* = 34.3 and 104.7 ppm, respectively.

**Scheme 3.**
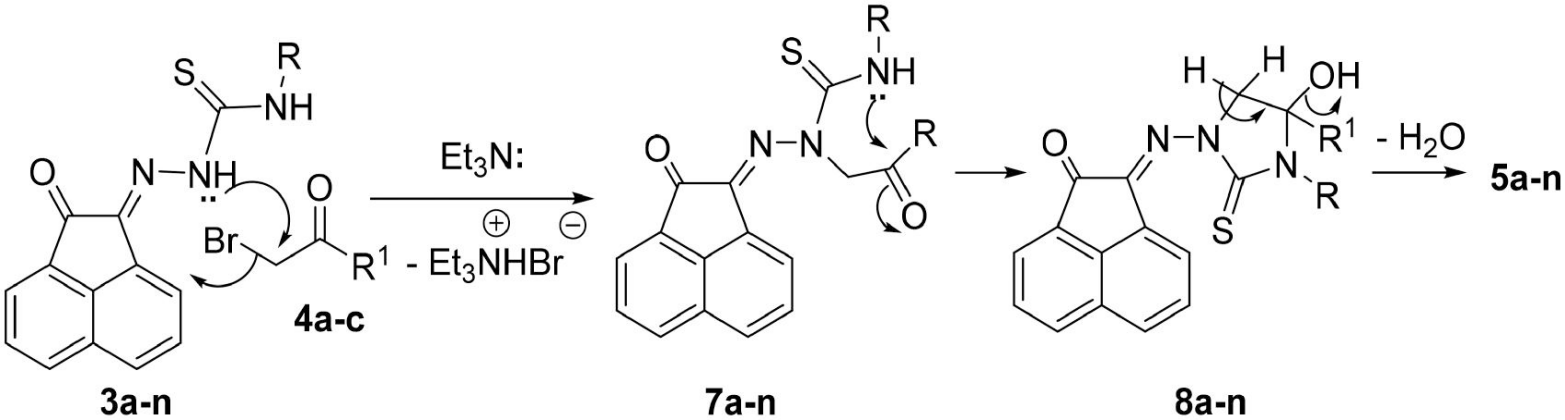
Mechanism describes the formation of **5a-n.**

The reaction mechanism describes the formation of **5a-n** was based upon an initial nucleophilic attack of hydrazone nitrogen lone-pair in **3a-n** to the active methylene of **4a-c** enhancing by the presence of Et_3_N. Thereafter, elimination of triethylamine hydrobromide molecule would then give the intermediates **7a-n** (Scheme 3). Further nucleophilic attack of the other free NH-lone pair to the electrophilic carbonyl carbon would give intermediates **8a-n**. Finally, elimination of water molecule from **8a-n** would give compounds **5a-n** (Scheme 3).

### Biological screening

#### DNA interaction studies

**Figure 5.**
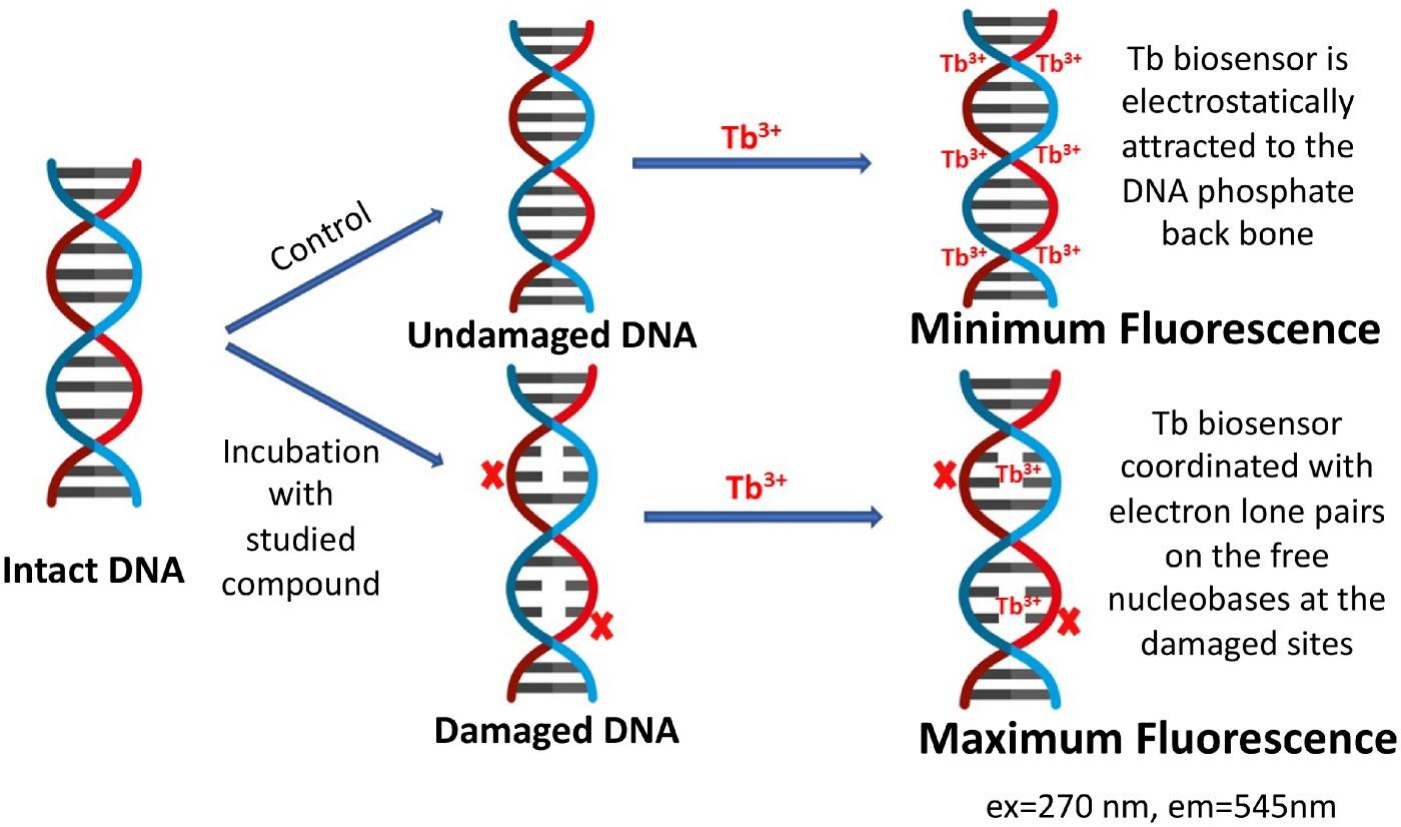
Schematic diagram for the fluorimetric detection of the induced DNA damage using terbium chloride (Tb^3+^) luminescent biosensor.

Luminescence studies were accomplished in order to examine the possible DNA interaction with the studied compounds. Many luminescent biosensors are reported in literature for studying the possibility of DNA interaction with small molecules^40–43^. In this article, we used the luminescent terbium (III) chloride (Tb) biosensor for probing such interaction due to its superior sensitivity, selectivity over many other reported biosensors, in addition to being inexpensive with simple mix-and-read procedure^44^. Figure 5 presents a schematic diagram for the fluorometric detection of the induced DNA damage using Tb biosensor. Tb^3+^ ion in the Tb luminescent probe is a trivalent lanthanide cation having low intrinsic luminescence in aqueous solutions^45^^,^^46^. When Tb biosensor is added to a solution of DNA, it interacts differently with double-stranded DNA (dsDNA) and single-stranded DNA (ssDNA). With dsDNA, Tb^3+^ ions are electrostatically attracted to the negatively charged phosphate backbone of the DNA. On the other hand, with ssDNA, it coordinates with the lone pairs of electrons of the free nucleobases, where upon DNA excitation, energy is transferred to the Tb biosensor causing significant enhancement of its luminescence^47^. Therefore, the assessed compound damages dsDNA, ssDNA sites would be formed opposite to the site of damage and Tb^3+^ would directly coordinate to the unpaired nucleobases in this single-stranded site. Upon excitation at λ_max _= 270 nm, energy will be transferred to Tb^3+^ with significant enhancement in its luminescence proportional to the amount of damage induced to dsDNA.

**Figure 6.**
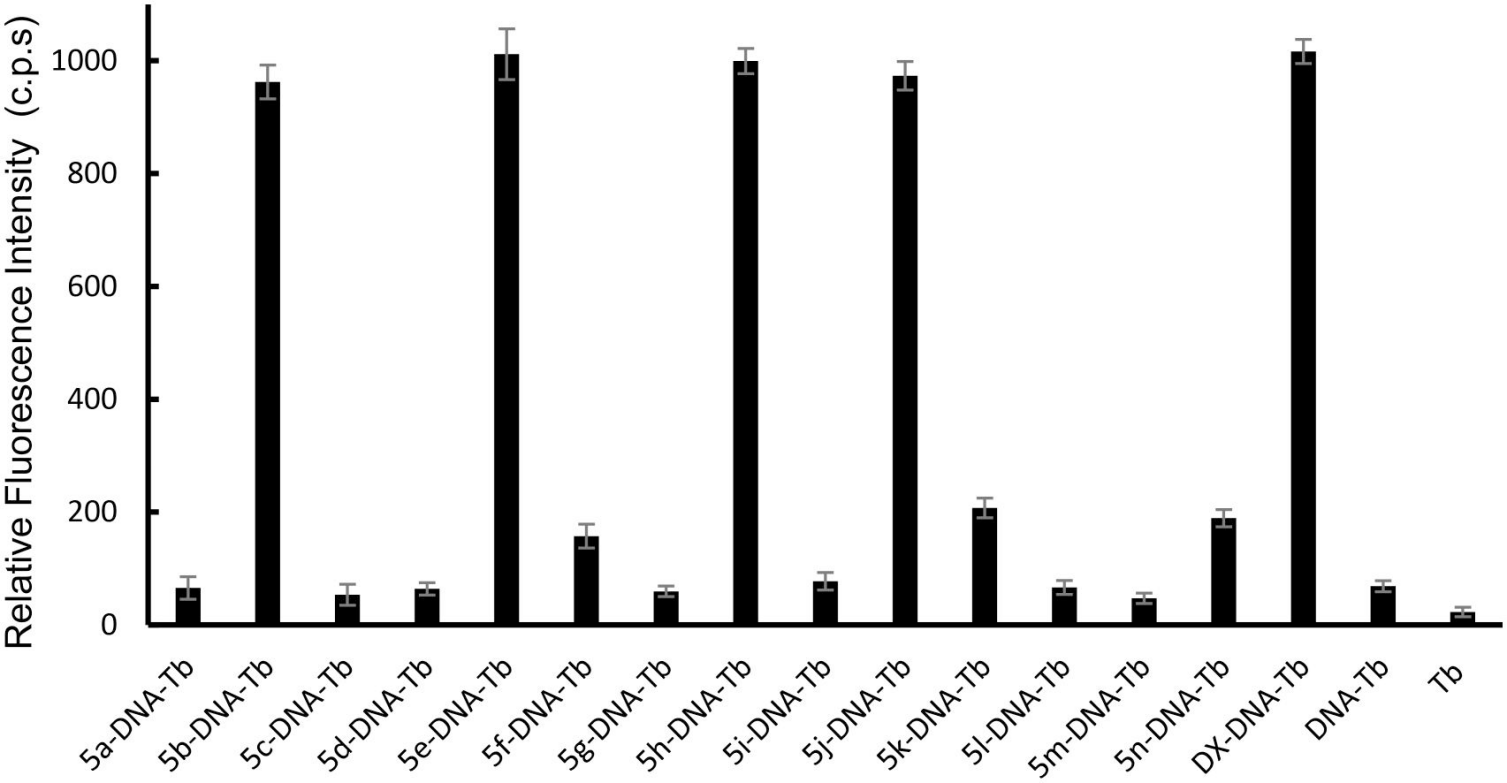
Tb^3+^ luminescence enhancement at λ_max_ = 545 nm after excitation at λmax = 270 nm of the studied compounds compared to reference **doxorubicin** (**DOX**). Controls such as DNA-Tb^3+^ mixture, and Tb^3+^ alone are also presented.

For investigating the DNA interaction potential of the fourteen studied compounds, calf thymus DNA (ctDNA) was incubated with each compound for 24 h and then Tb was added to each mixture. **Doxorubicin** (**DOX**), a commonly used reference for DNA damaging agent was used and treated the same condition as the studied compounds. Tb luminescent intensity was measured at λ_max_ = 545 nm after excitation at λ_max_ = 270 nm. Tb luminescence enhancement of each compound was compared with the luminescence of ctDNA-Tb mixture and Tb solution alone (results are demonstrated in Figure 6). A significant enhancement of Tb luminescence appeared with ctDNA samples incubated with **doxorubicin** and compounds **5b**, **5e**, **5h** and **5j**.

Compounds **5f**, **5k** and **5n** showed minimum enhancement of Tb luminescence. While, the entire remaining compound did not any significant change in Tb luminescence from that of ctDNA-Tb mixture. The significant increase in Tb luminescence is related to the coordination of Tb with free nucleobases at the damage sites in ct DNA that were induced by compounds **5b**, **5e**, **5h**, and **5j**. While the slight increase in the biosensor luminescence indicates that compounds **5f**, **5k**, and **5n** induced minimum DNA damage with the formation of very small number of damaged sites in DNA and thus, less potent to cause DNA damage. The remaining seven compounds did not show any significant interaction with DNA and thus the recorded luminescence was comparable to that of ctDNA-Tb with no compounds added.

**Figure 7.**
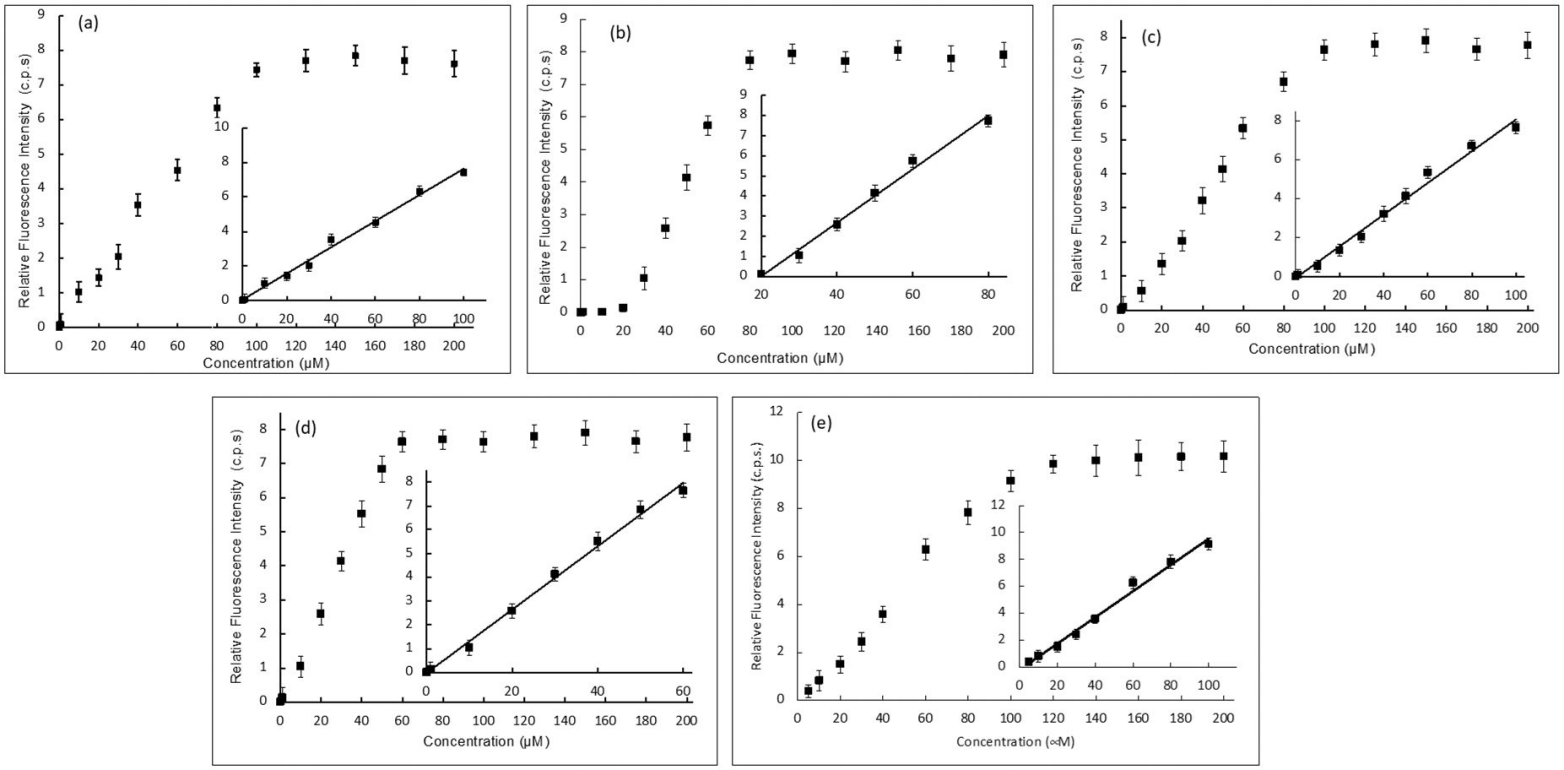
Effect of increasing concentration of (**a**) **5b**, (**b**) **5e**, (**c**) **5h**, (**d**) **5j**, and (**e**) **doxorubicin** (**DOX**) as a reference of the luminescence of Tb- ctDNA complex. The insets show the fit to the linear region of the constructed plots.

The potency of DNA damage induction for each of the four most potent compounds, **5b**, **5e**, **5h**, and **5j**, was further assessed on natural ctDNA and compared to **doxorubicin**. Several concentrations within the range of 0.1 pM–200.0 μM of each of the studied compounds were incubated with ctDNA for 24 h. After which, Tb solution was added to aliquots of such mixtures and the luminescence intensity was recorded. Figure 7 presents Tb luminescence intensity as a function of the compound concentration for various compound/ctDNA/Tb mixtures. At minimum concentration of the studied compounds, minimum Tb luminescence was recorded. This reflects that the DNA molecule is undamaged, intact and acquiring a double helical structure. In this conformation, Tb ions are electrostatically attracted to the negatively charged DNA-phosphate backbone showing no increase in its luminescence. For solutions with higher concentrations of compounds **5b**, **5h**, and **5j** (Figure 7a,c,and d). Tb fluorescence increased proportionally with the concentration until a concentration of 100 and 60 μM for compounds **5b**, **5h**, and **5j**, respectively, where no further increase in the fluorescence occurs. This reflects that more DNA damage is induced with increasing concentrations of the three compounds. At concentrations higher than 100 and 60 μM for compounds **5b**, **5h**, and **5j**, respectively, Tb luminescence remains constant at high fluorescence levels. That indicates that maximum DNA damage has occurred. On the other hand, for compound **5e**, no enhancement of Tb luminescence intensity with increasing compound **5e** concentration until 20 μM (Figure 7b), after which proportional increase in Tb luminescence was recorded with the increase of **5e** concentration until 80 μM, whereas the Tb luminescence remains constant at high luminescence indicating that maximum DNA damage has been attained. Table 1 illustrates the analytical parameters elucidated as shown in Figure 7 for the quantification of the induced DNA damage by the studied compounds. The linearity parameters were calculated from the calibration curve in Figure 7-inset in order to obtain the minimum concentration of the studied compounds that would induce DNA damage. Results show that a concentration as low as 7.2,7,14,78.14 and 12.7 nM of **5b**, **5e**, **5h,** and **5j**, respectively, are enough to induce detectable damage in ctDNA which comparable to **doxorubicin** (**Table 1**) indicating their potency to induce DNA damage in natural DNA with compound **5h** being the most potent to induce direct DNA damage.

**Table 1.** Analytical parameters for the fluorescence measurements of **5b**, **5e**, **5h**, **5j**, and **doxorubicin** as a reference with ctDNA mixtures.

Parameter	5b -DNA	5e -DNA	5h -DNA	5j -DNA	DOX-DNA
Linear dynamic range	0.01pM–100.0 μM	20.0–80.0 μM	0.01pM–100.0 μM	0.01pM–60.0 μM	0.01pM–100.0 μM
Correlation coefficient (r)	0.9915	0.9938	0.9964	0.9911	0.9912
Intercept (a)	6366.2	−263553	−1689	−5822.3	2167.3
Slope (b)	13283	7580	13306	8124.2	8923.2
LOD^a^	7.78 nM	14.2 nM	7.14 nM	12.7 nM	4.09 nM
LOQ^b^	25.9 nM	46.9 nM	23.8 nM	42.4 nM	13.7 nM

^a^ LOD:Limit of detection.

^b^ LOQ: Limit of quantitation.

#### In vitro antitumor evaluation

**Figure 8.**
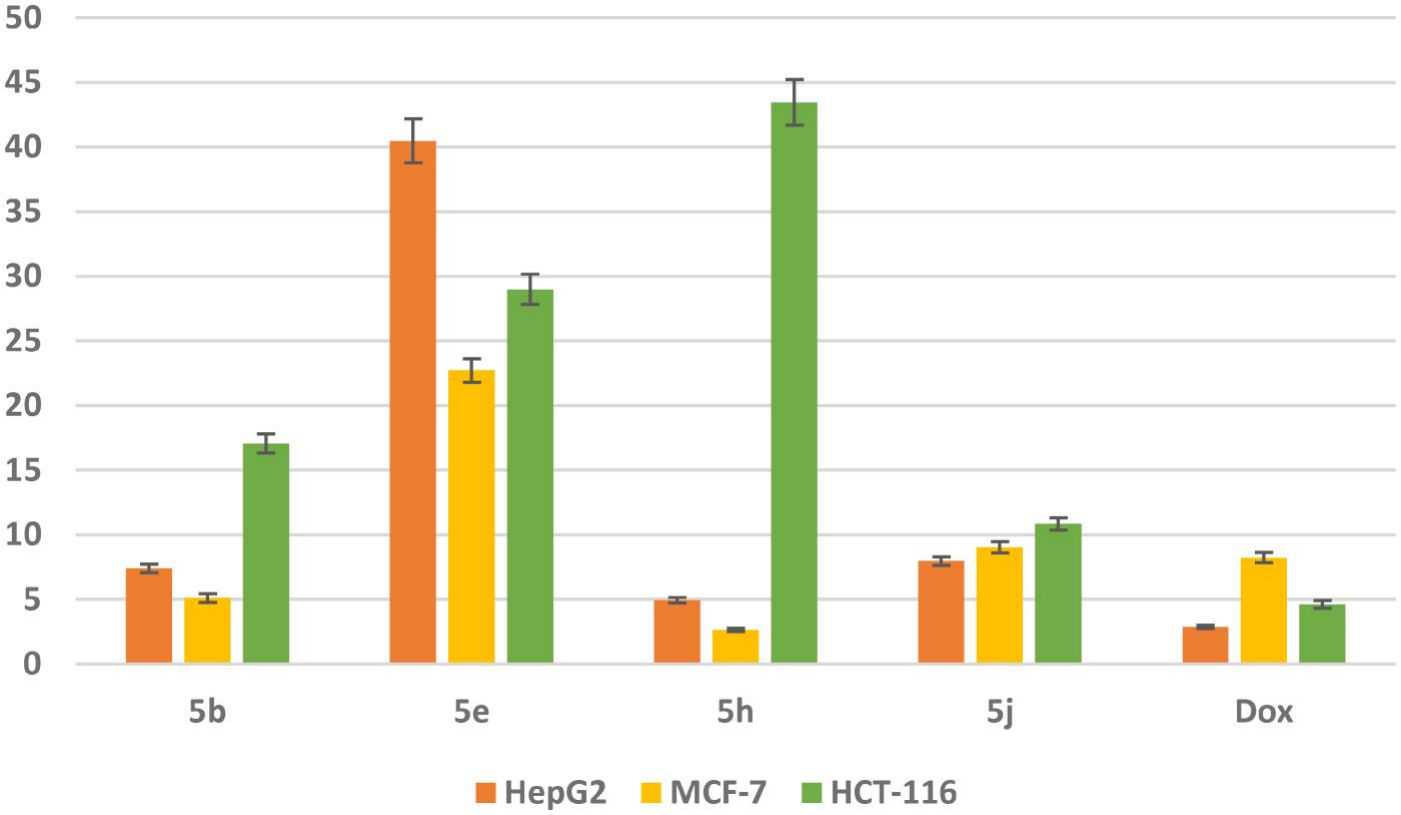
*In vitro* anticancer activity, IC_50_ (μM) of **5b**, **5e**, **5h,** and **5j** in comparison to **Doxorubicin**.

*In vitro cytotoxic activity against three cancer cell lines:* The most potent DNA intercalating compounds **5b**, **5e**, **5h**, and **5j** were *in vitro* evaluated against their anticancer activities against three cancer cell lines namely; hepatocellular carcinoma (HepG2), human breast adenocarcinoma (MCF-7) and human colon cancer (HCT-116) utilising MTT assay^48^ and the most active compounds were evaluated against normal breast cell line (MCF-10a). The IC_50_ values of the newly synthesized compounds were compared to **doxorubicin** as a positive control as shown in Figure 8. The obtained IC_50_ values of the tested compounds displayed different levels of anticancer activity ranging from high to weak activities against all tested cell lines.

Data represented in Figure 8 revealed that compounds **5b** and **5h** exhibit superior anticancer activity against MCF-7 cell line with IC_50_ values 5.105 and 2.65 μM, respectively compared with **doxorubicin** as a reference drug with IC_50_ value of 8.240 μM. Meanwhile, they showed moderate activity against HepG2 cell line with IC_50_ values 7.397 and 4.929 μM, respectively, and weak anticancer activity against HCT-116 cell line with IC_50_ values 17.063 and 43.45 μM, respectively. Moreover, the safety of compounds **5b** and **5h** was assessed against a normal breast cell line (MCF-10a) using the MTT assay. The results revealed that compounds **5b** and **5h** showed a remarkably less cytotoxic effect towards MCF-10a with IC_50_ value of 89.759 and 130.576 μM, respectively compared with Dox with IC_50_ value of 32.416 μM. In addition, compound **5j** showed moderate anticancer activity against three cancer cell lines HepG2 and MCF-7 and HCT-116 with IC_50_ value of 7.9, 964.030 and 10.847 μM, respectively. While compound **5e** exhibited weak anticancer activity against three cancer cell lines.

The selectivity index (SI)^49^ was calculated for the most active compounds **5b** and **5h** against breast cancer cell line and the results revealed that, compounds **5b** and **5h** showed high selectivity index (17.58 and 49.27, respectively) against MCF-7 cell line compared with **doxorubicin** with value of 3.9 (Table 2). Consequently, compounds **5b** and **5h** were the least cytotoxic and highest anticancer activity with high SI value compared to the Dox. These results indicated a successful discovery of new anticancer drug candidates and confirmed that they could be safe drugs against tumours.

**Table 2.** Selectivity Index (SI) of compounds **5b**, **5h,** and doxorubicin against human breast adenocarcinoma (MCF-7).

Compound ID	MCF-7
**5b**	17.58
**5h**	49.27
**Dox**	3.9

The initiation of apoptosis and the stages of the cell cycle are important in the era of cancer treatment. Several imidazole-2-thiones molecules have been reported as apoptosis inducers in several cancer cell lines. Therefore, the two most active compounds **5b** and **5h**, were chosen to evaluat their effect on apoptosis and the cell-cycle profile.

**Figure 9.**
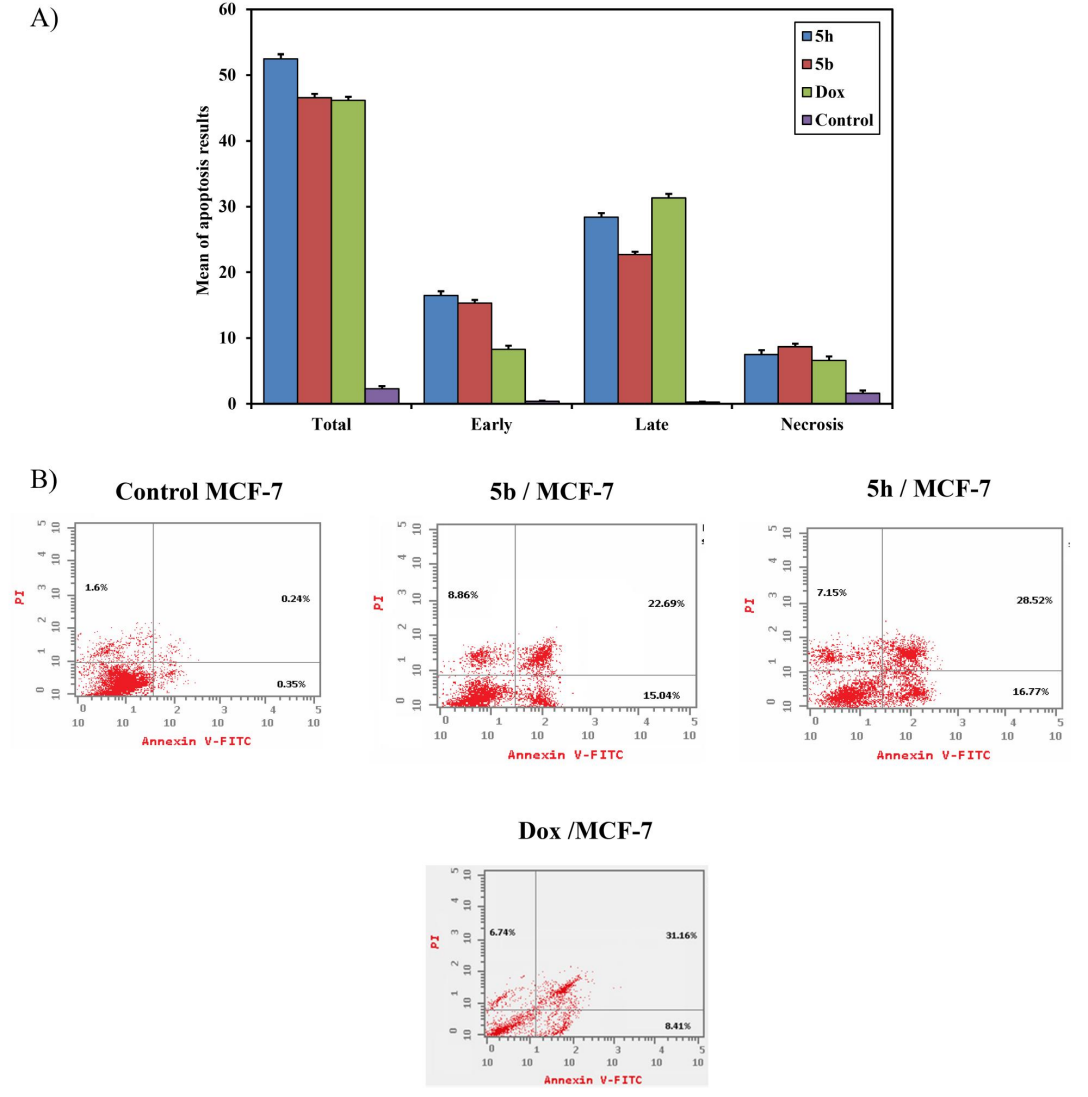
(**A**) A graphical representation of apoptosis induction analysis of compounds **5b, 5h** and **doxorubicin** as reference against MCF-7 at their IC_50_(μM). (**B**) Apoptosis induction analysis of compounds **5b**, **5h**, and **doxorubicin** as reference against MCF- 7 at their IC_50_ (μM).

*Annexin V-FITC/PI apoptosis induction analysis*: The breast carcinoma cell line (MCF-7) was used for revealing the induction of apoptosis and cell-cycle profile of compounds **5b**, **5h**, and **doxorubicin** as a reference drug owing to its significant sensitivity towards those compounds. Flow cytometric annexin V/propidium iodide analysis^50^ was used for detecting the apoptotic effects of the most active and non-toxic anticancer compounds **5b**, **5h**, and **doxorubicin** and applied to MCF-7 cells at concentrations equal to their IC_50_ values (5.105, 2.65 and 8.240 μM, respectively) on the MCF-7 cell line. The results indicated that the selected candidates induce apoptosis of MCF-7 cells at both early and later stages. As shown in Figure 9, the percentage of early apoptosis increased from 0.37 ± 0.11% (control) to 15.3 ± 0.50% and 16.5 ± 0.64% for compounds **5b** and **5h**, respectively. Whereas **doxorubicin** showed an increase in the percentage of early apoptosis by 8.3 ± 0.53%. In addition, the percentage of late apoptosis was increased from 0.26 ± 0.06% (control) to 22.7 ± 0.43% and 28.4b ± 0.62% upon exposure to compounds **5b** and **5h**, respectively. At the same time, **doxorubicin** induced the late cell apoptosis by 31.3 ± 0.62%. So, these findings confirm that compounds **5b** and **5h** induce apoptosis in MCF‐7 cells by total cell apoptosis higher than **doxorubicin** as shown in Figure 9. It can be concluded that the anticancer mechanism of action for compounds **5b** and **5h** is attributed to their apoptosis inducing activity in MCF-7 cells.

**Figure 10.**
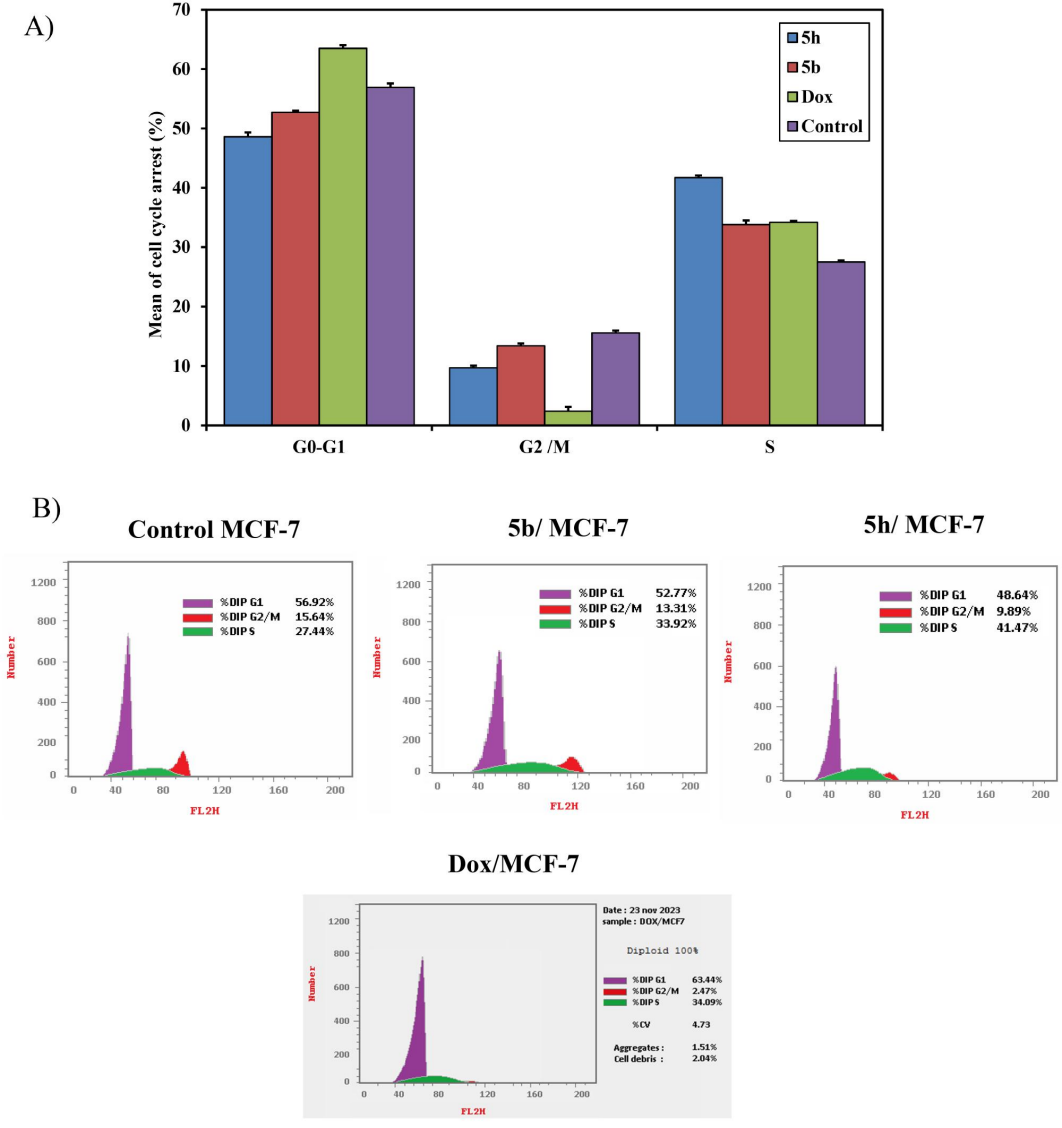
(**A**) A graphical representation of cell cycle analysis of compounds **5b**, **5h,** and **doxorubicin** as a reference against MCF-7 at their IC_50_ (μM). (**B**) Cell cycle analysis of compounds **5b**, **5h**, and **doxorubicin** as a reference against MCF-7 at their IC_50_ (μM).

*Cell cycle analysis:* The most active compounds **5b and 5h** were investigated for their cell cycle profile using DNA flow cytometric analysis on MCF-7 in comparison with **doxorubicin** as a reference drug^22^^,^^51^. Treatment of MCF-7 cells with IC_50_ concentration dose value of those compounds resulted in a significant alteration in cell cycle profile. The results revealed significant increase in the percentage of cell population at the S phase from (27.5 ± 0.28%) control to 33.8 ± 0.70% and 41.7 ± 0.39% upon treatment with compounds **5b** and **5h**, respectively. In addition, **doxorubicin** induced cell cycle arrest at G1/S phase in comparison with the untreated control. Therefore, it can be concluded that compounds **5b** and **5h** inhibited the cell proliferation of MCF-7 cells via cell cycle arrest at the S phase similar to **doxorubicin** as reference drug (Figure 10). For compounds with a mechanism of action similar to **doxorubicin**, it can be expected an arrest at the G2/M phase. However, compounds **5b** and **5h** arrested at the S phase which may be due to the presence of acenaphythylenone, the poly aromatic hydrocarbon (PAH). It was found that the large adducts of PAH to DNA bases can result in a range of chromosomal changes, including frameshift mutations, deletions, S-phase arrest, and chromosomal alterations^52^. According to the literature, it was reported that the polyaromatic hydrocarbons only suppressed the S phase of the cell cycle^53^. A probable explanation for the observed cell cycle arrest at the S phase is the production of DNA damage as a result of DNA intercalation within the cells. During the mitotic S phase, DNA replication and Topo II levels rise^54^^,^^55^. Therefore, those compounds that destroy cells in the S phase aid in stopping DNA and topo II production.

**Figure 11.**
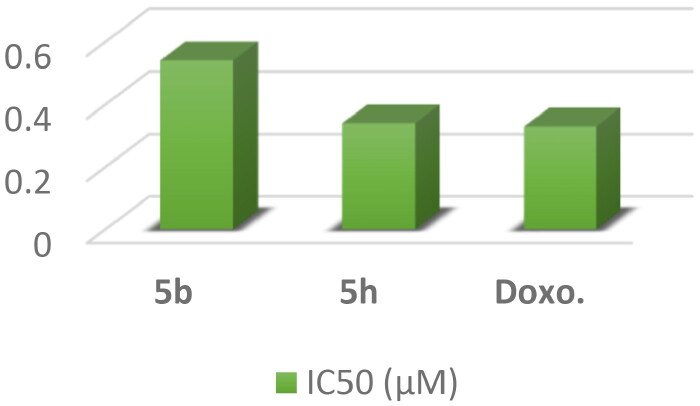
A graphical representation of **5b,** **5h **and **DOX** against Topo II inhibitory activity.

*Topoisomerase II inhibitory activity*: The most active compounds **5b** and **5h** were further examined as topoisomerase II inhibitors using **doxorubicin** as a positive control to expect the possible molecular mechanism underlying the potent anticancer activity of compounds^56^. The results showed that compound **5h** revealed potent inhibitory activity against topo II enzyme with IC_50_ value of 0.34 μM compared with **doxorubicin** (IC_50_ = 0.33 μM). Moreover, compound **5b** showed moderate inhibitory activity against topo II enzyme with IC_50_ value of 0.54 μM (Figure 11).

### Structure activity relationship (SAR)

**Figure 12.**
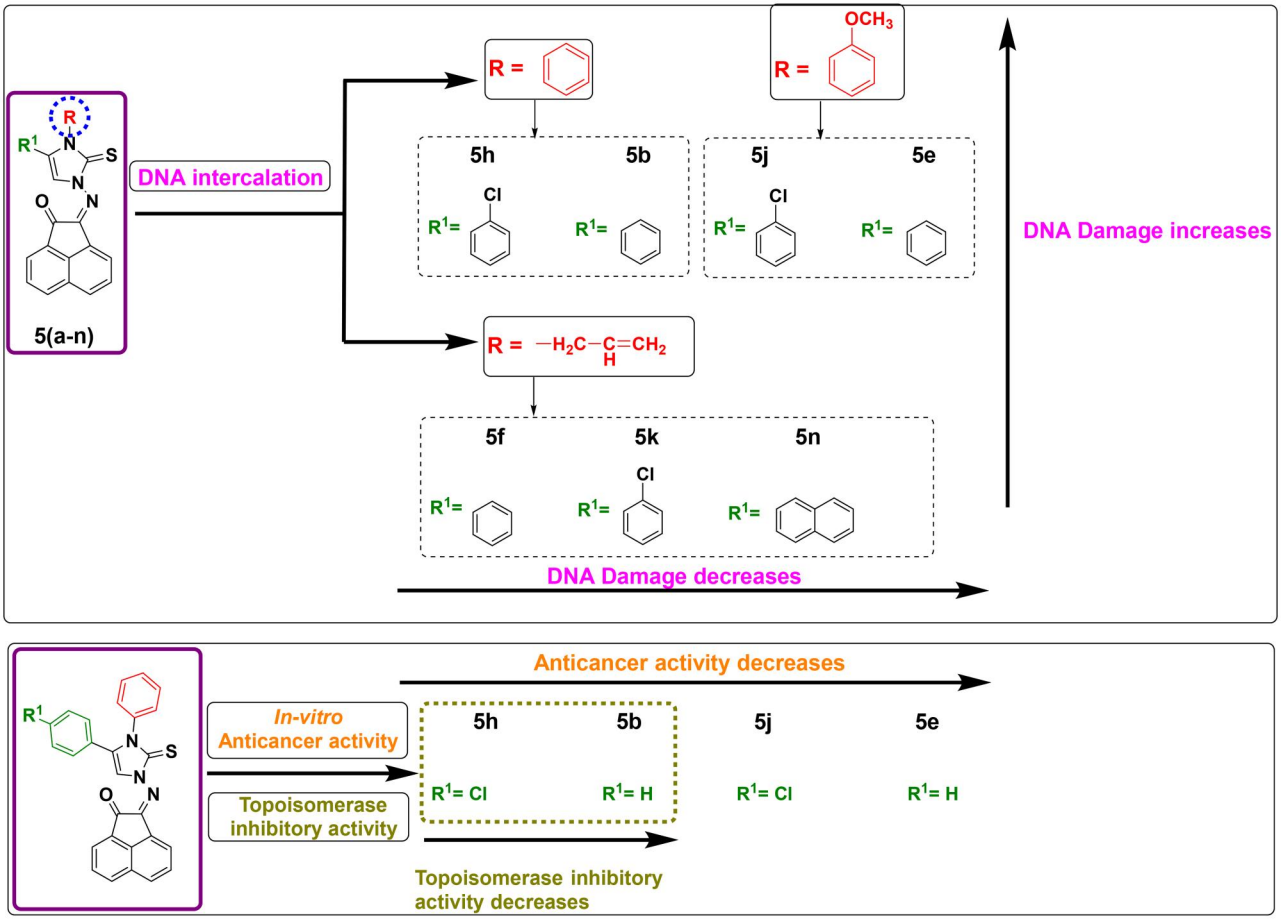
Structure-Activity relationship of DNA damage testing, antiproliferative activity and topoisomerase inhibitory activity for tested compounds.

The findings from biological screening offered a useful profile for the structure-activity relationship and demonstrated a positive influence on the structure optimization of newly synthesized compounds. Among the study, anticancer screening was significantly impacted by the substitution of the imidazole ring at its N-atom or at position 4 with diverse aliphatic and aromatic moieties. The interpreted findings of induction of DNA damage using terbium fluorescent probe were varied in which seven compounds out of fourteen tested ones exhibited damage to DNA. These seven compounds shared an aromatic scaffold at imidazole carbon-4. It was noticed that compounds bearing *N*-phenyl or *N*-methoxy phenyl moiety of imidazole showed the most potent induction of DNA damage as found in **5b**, **5e**, **5h**, and **5j**. It was also noticed that compounds bearing *N*-allyl moiety of imidazole ring showed moderate induction of DNA damage as in **5f**, **5k**, and **5n**. This observation demonstrates that replacing the cyclic aromatic scaffold into an open unsaturated chain moiety decreases the activity as DNA intercalator, while aliphatic substitution (methyl or cyclohexyl) showed no significant activity. On the other hand, the antiproliferative activity was examined the highest in compounds **5b** and **5h** that bearing *N*-phenyl moiety of imidazole-2-thione ring, while methoxy phenyl derivatives **5j** and **5e** exhibited less anticancer potency against selected cell lines. This finding may explain why less bulkiness at imidazole nitrogen is favourable. In addition, the substitution at position 4 of imidazole-2-thione with 4-chlorophenyl moiety (**5h**) instead of unsubstituted phenyl (**5b**) significantly duplicated the topoisomerase inhibitory activity. This may be attributed to the importance of substituted atoms in enhancing the fitting of the protein active site (Figure 12).

### In silico studies

#### Molecular docking

**Figure 13.**
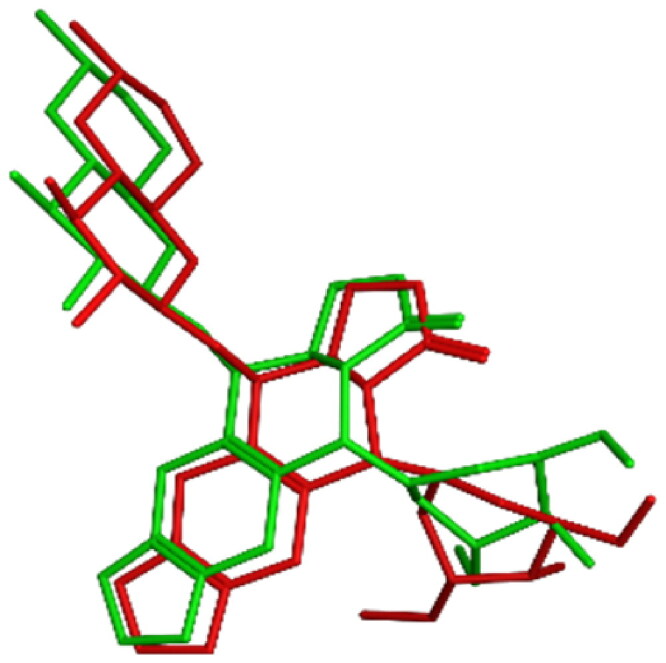
3D representation of the overlay of re-docked ligand (green) to the co-crystallized ligand **EVP** (red) (RMSD =1.06 Å).

To gain further comprehension about the binding mode of topoisomerase II as a potential anticancer target, *in-silico* docking experiments were established for all tested compounds, and comparisons with the *in-vitro* assay results were visualised and anticipated. Studies employing molecular docking were performed on the binding site of human topoisomerase II beta enzyme complexed with DNA (PDB ID: 3QX3) using Molecular operating Environment software (MOE 2022.02)^57^. The purpose was to give an account about the mechanism of action of the most active compounds, their inhibitory activities and their binding affinities to the enzyme active site. The outcomes were contrasted with the co-crystallized ligand: **etoposide** (**EVP**) and to reference drug **doxorubicin** (**DOX**). The proposed docking algorithm was validated by re-docking of the co-crystallized ligand etoposide into the active site of human topoisomerase IIβ DNA complex. The initial poses generated from PDB were retrieved with RMSD of 1.06 Å and with significant overlay of re-docked ligand to the co-crystallized ligand **EVP** (Figure 13). Appropriate RMSD indicated that MOE docking can reliably predict docking poses for the studied compounds and assure the proposed docking protocol^58^.

**Figure 14.**
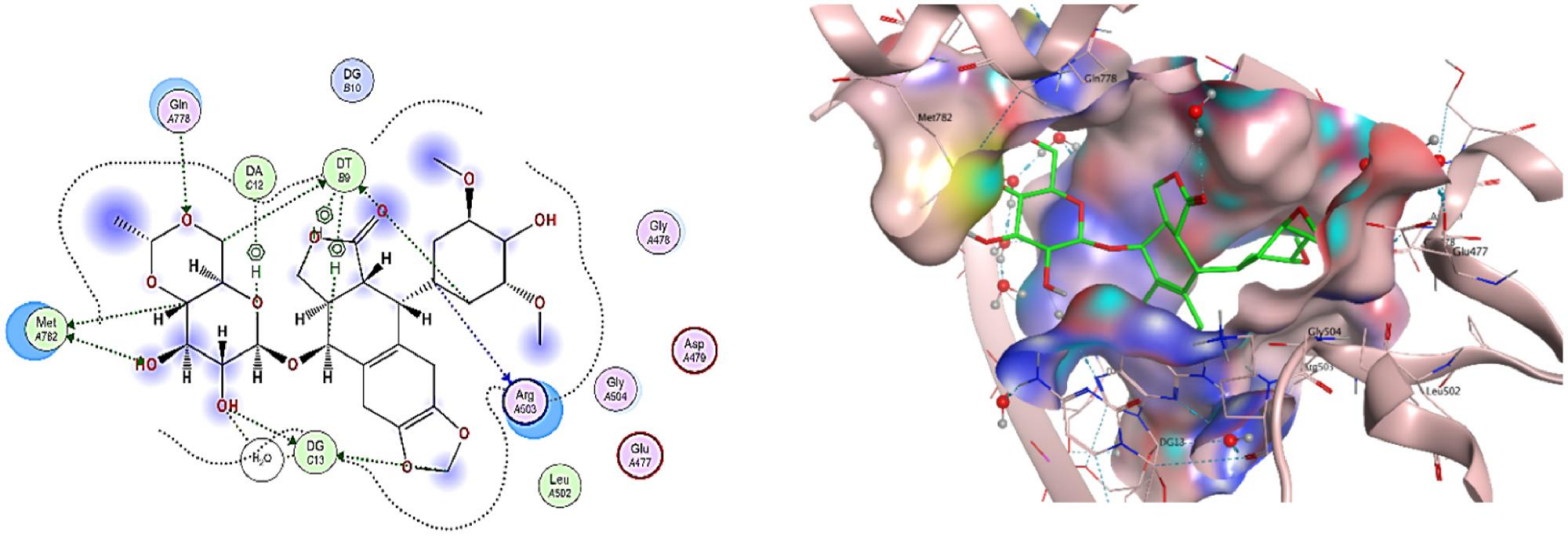
The binding interaction of **EVP** (green) at the pocket of the active site of DNA-Topo IIβ complex in 2D (left panel) and 3D representation (right panel).

It was recorded that interactions of etoposide **EVP** in DNA-Topo IIβ complex encompass some aminoacid residues as; GLU477, ASP479, Arg503, GLN778 and Met782, in addition to some nucleotides such as: guanine13, adenine12 and thymidine 9^2^^,^^3^. Consequently, the re-docking results of the co-crystallized ligand **EVP** to the active site of DNA-Topo IIβ complex showed binding score = −10.092 Kcal/mol. **EVP** displayed many hydrogen bonds interactions between its sugar moiety and Gln778, Met782, in addition to other hydrogen bonds interactions with DT9, DG13 and a water molecule. The cyclohexyl ring in EVP also showed two hydrogen bonds with Arg503 and DT9, while the planar aromatic ring of EVP exhibited a hydrogen bond with DG13 besides other H-pi interactions with DT9 and DA12 (Figure 14).

**Figure 15.**
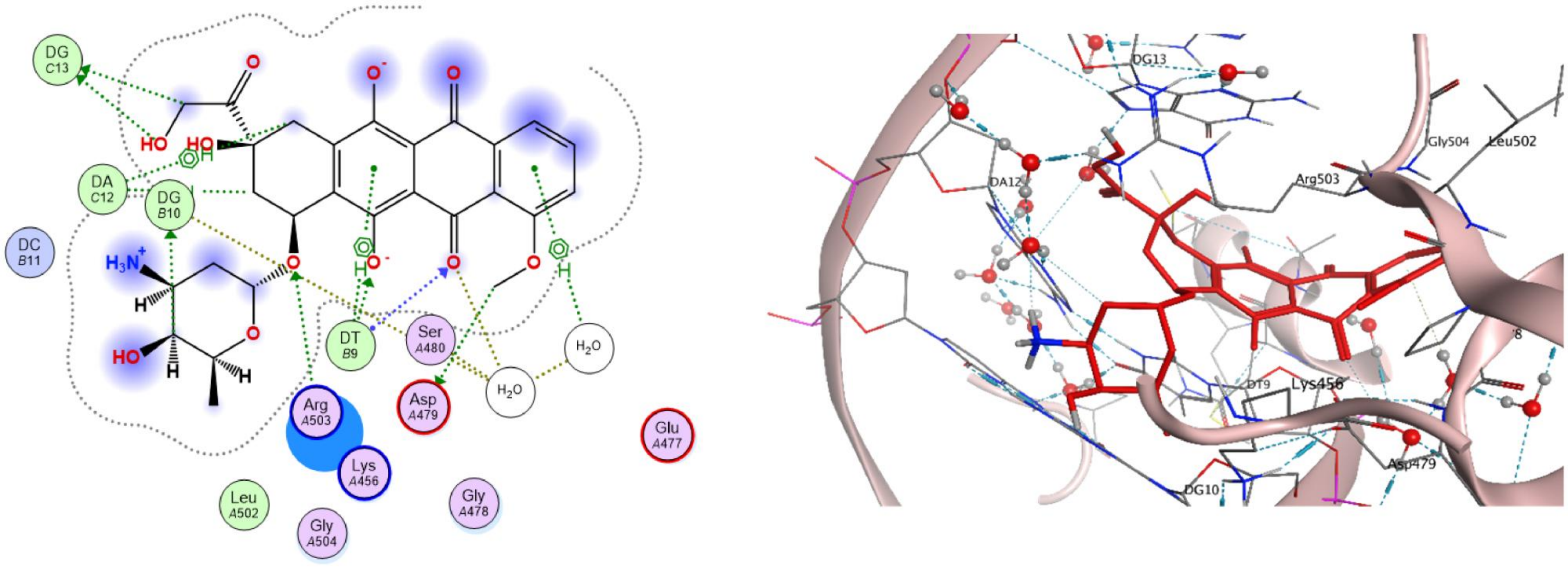
The binding interaction of **doxorubicin** (red) at the active site of DNA-Topo IIβ complex in 2D (left panel) and 3D representation (right panel).

Docking interpretation was performed on **doxorubicin** as reference drug at the active site of DNA-Topo IIβ complex and it showed binding score = −6.41 Kcal/mol and it shared many interactions with crucial aminoacids and nucelobases all with distances less than 4.5 Å as shown in Figure 15. It was observed that methoxy group at C-4 of tetracene ring binds with Asp479 through a hydrogen bond. Also, the carbonyl group at C-5 of tetracene forms two hydrogen bonds with DT9 and water molecule as well as the hydroxy group at C-6. Another hydrogen bond was observed between O-atom at C-7 of tetracene and Arg503. Moreover, the hydroxy acetyl moiety shared two hydrogen bonds with DG13. Also, C-4 of pyran ring binds to DG10 *via* hydrogen bonding. In addition, tetracene ring exhibited four H-pi interactions with DT9, DA12 and with a water molecule.

All newly synthesized compounds underwent docking assessments, and their binding scores and binding modalities were evaluated. As shown in Table 3, compounds **5a-n** were successfully occupied in the active site of topoisomerase II and displayed favourable binding scores between −6.59 and −8.07 Kcal/mol. The former examination was done together with **doxorubicin** and co-crystallized ligand etoposide (EVP). With crucial essential amino acids and nucleobases, they demonstrated various hydrophilic and hydrophobic interactions with accepted distances (less than 4.5 Å). It was evident that the sulphur atom and imidazole ring displayed the most frequent hydrogen bond interactions in the protein pocket. Likewise, Numerous arene-hydrogen interactions were shared by the polyaromatic acenaphthylenone moiety.

**Table 3.** Molecular docking scoring and interactions of tested compounds at the active site of DNA-Topo IIβ (PDB ID: 3QX3) in comparison to reference drugs EVP and DOX.

Tested compound	Binding score	Type of interaction	Binding pattern representation
	(Kcal/mol)	(amino acid residue)	
5a	−6.80	H-acceptor (Gln 778)	SI Figure 95
		H-acceptor (H_2_O molecule)	
5b	−6.59	H-donor (Glu 477)	Figure 16
		H-donor (Asp 557)	
		H-acceptor (H_2_O molecule)	
		2 H-Pi (DG13)	
		4 Pi-H (Arg503)	
		2 Pi-H (DT9)	
		2 Mg metal interaction	
5c	−7.40	2 Pi-H (Arg503)	SI Figure 96
5d	−7.69	H-acceptor (DT9)	SI Figure 97
		H-acceptor (DG 10)	
		H-acceptor (H_2_O molecule)	
		Pi-Pi (DG13)	
5e	−7.76	H-acceptor (DT9)	SI Figure 98
		H-acceptor (H_2_O molecule)	
5f	−7.16	2 H-acceptor (H_2_O molecule)	SI Figure 99
5 g	−7.01	Pi-H (Arg503)	SI Figure 100
		Pi-H (DT9)	
5h	−7.00	2 H-donor (DT9)	Figure 17
		H-donor (Arg503)	
		H-acceptor (Gly478)	
		H-acceptor (Asp479)	
		H-acceptor (H_2_O molecule)	
		H-acceptor (DA12)	
		Pi-H (Arg503)	
		Pi-H (Gly504)	
		Pi-H (DT9)	
5i	−7.64	Pi-H (Arg503)	SI Figure 101
		H-donor (DT9)	
5j	−7.93	2 H-acceptor (H_2_O molecule)	SI Figure 102
		H-acceptor (Asp479)	
		H-acceptor (DT9)	
		H-acceptor (DG10)	
5k	−7.53	H-acceptor (H_2_O molecule)	SI Figure 103
		Pi-Pi interaction (DT9)	
5l	−7.78	H-acceptor (DT9)	SI Figure 104
		H-acceptor (H_2_O molecule)	
5m	−8.07	H-acceptor (DG13)	SI Figure 105
		2 Pi-Pi interaction (DG13)	
		Pi-H (DA12)	
5n	−7.75	Pi-H (Arg503)	SI Figure 106
		H-Pi (DG13)	
		H-Pi (DT9)	
Dox^a^	−6.41	2 H- donor (DG13)	Figure 15
		H- donor (Asp479)	
		H-donor (DG 10)	
		2 H- acceptor (DT9)	
		H- acceptor (Arg503)	
		H-acceptor (H_2_O molecule)	
		2 H-Pi (DA12)	
		Pi-H (DT9)	
		Pi-H (H_2_O molecule)	
EVP^b^	−10.092	2 H- donor (DG13)	Figure 14
		H- donor (Arg503)	
		2 H- donor (DT9)	
		H-acceptor (Gln778)	
		H- donor (Met782)	
		H-acceptor (Met782)	
		H-acceptor (H_2_O molecule)	
		2 H-Pi (DT9)	
		H-Pi (DA12)	

^a^ **Doxorubicin.**

^b^Co-crystallized ligand etoposide.

**Figure 16.**
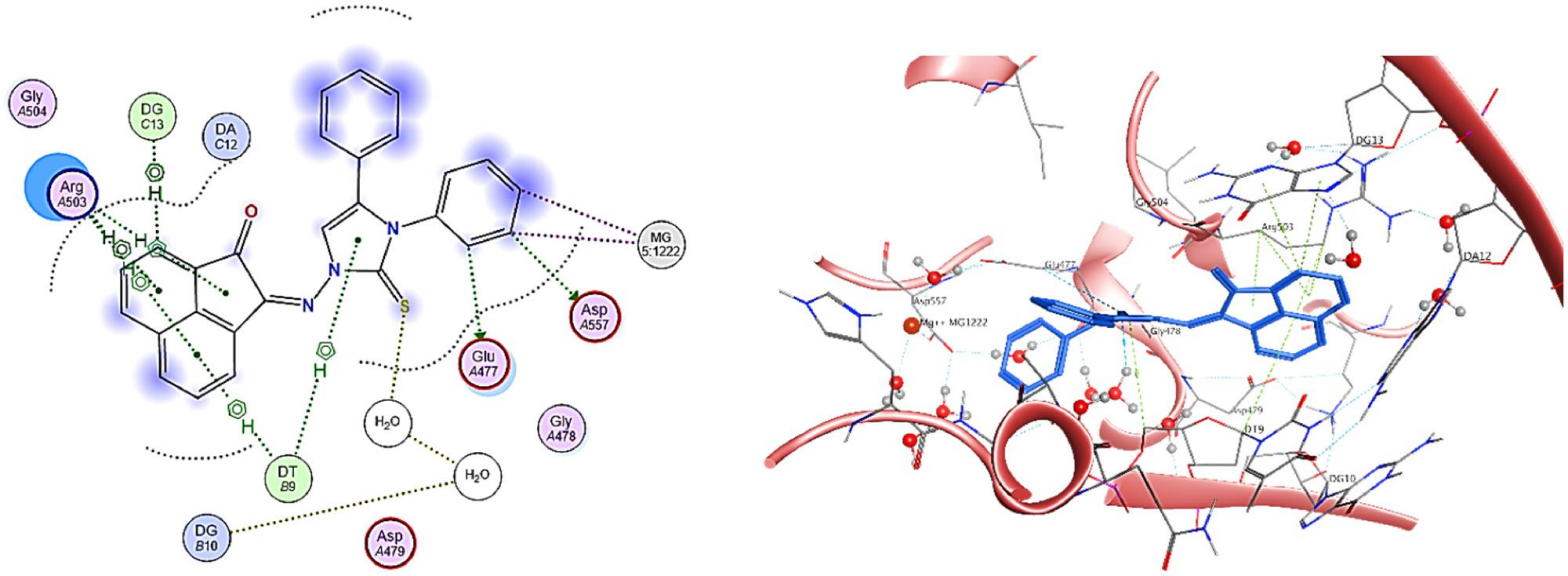
The binding interaction of compound **5b** (blue) at the active site of DNA-Topo IIβ complex in 2D (left panel) and 3D representation (right panel).

Regarding the findings of biological screening as DNA intercalation, anticancer activity and topoisomerase inhibition, it was observed that the most active compounds **5b** and **5h** were shown to have excellent binding modes at the active site of the DNA-Topo II complex with a variety of interactions that are highly similar to those identified with co-crystallized ligand EVP. The binding pattern of compound **5b** was examined and its binding score to DNA-Topo IIβ complex active site was found equal to −6.59 Kcal/mol. It was investigated that compound **5b** shared key aminoacids and nucleobases as found in **EVP** as follows: two hydrogen bonds were observed between phenyl ring substituted on *N*-atom of imidazole ring and Glu477, Asp557, in addition to two Mg metal interactions with the same phenyl moiety. Moreover, sulphur atom of imidazole ring binds to a water molecule through a hydrogen bond whereas imidazole ring binds to DT9 *via* H-pi interaction. It was observed that acenaphthylenone ring involves many H-pi interactions with Arg503, with DG13 and with DT9 with distances less than 4.5 Å (Figure 16).

**Figure 17.**
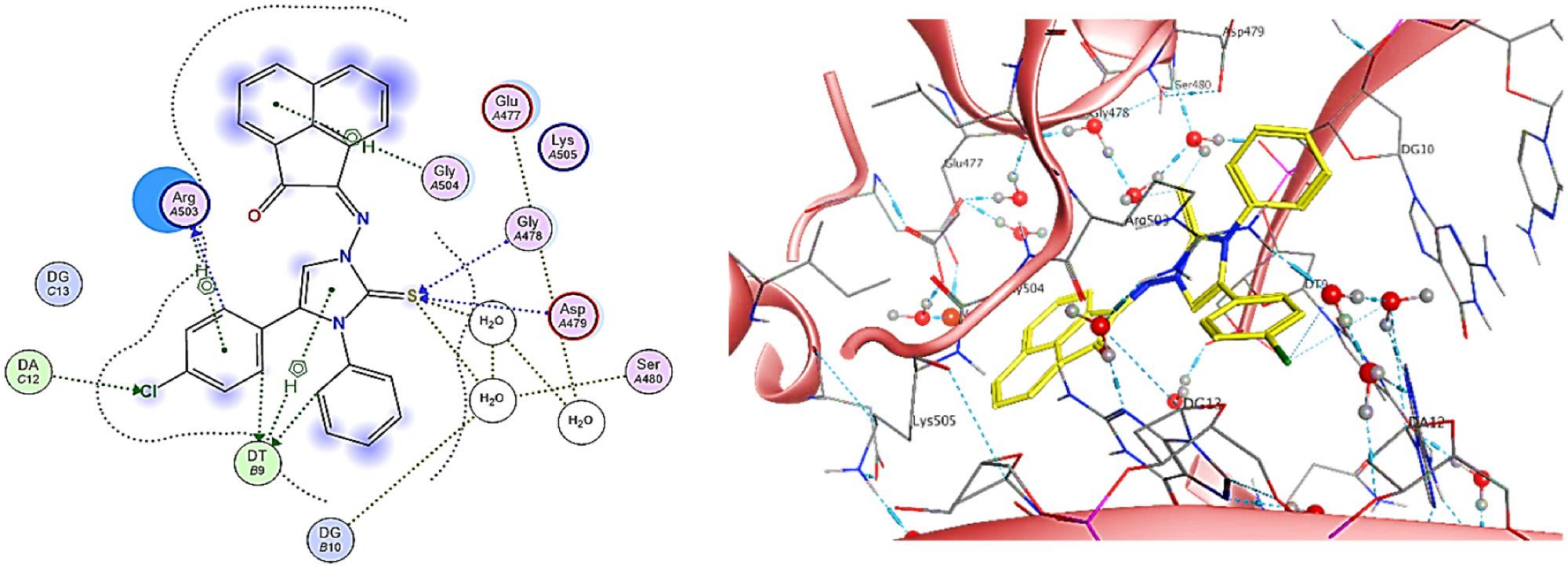
The binding interaction of compound **5h** (yellow) at the active site of DNA-Topo IIβ complex in 2D (left panel) and 3D representation (right panel).

By observing the binding pattern of compound **5h**, it was crystal clear that it showed excellent binding mode at DNA-Topo IIβ complex active site, with binding score = −7.00 Kcal/mol. The docking results showed both hydrogen bond and H-pi interactions between chloro-phenyl ring substituted at C-4 of imidazole ring and Arg503 and another hydrogen bond was observed with DT9. Similarly, hydrogen bonds were examined between chlorine atom and DA12 as well as between the phenyl ring substituted on N-atom of imidazole ring and DT9. Likewise, four hydrogen bonds were observed between sulphur atom of imidazole ring and Gly478, Asp479 and two water molecules all with distances less than 4.5 Å. In addition, two H-pi interactions were detected between acenaphthylenone ring and Gly504, and between imidazole ring and DT9 (Figure 17).

**Figure 18.**
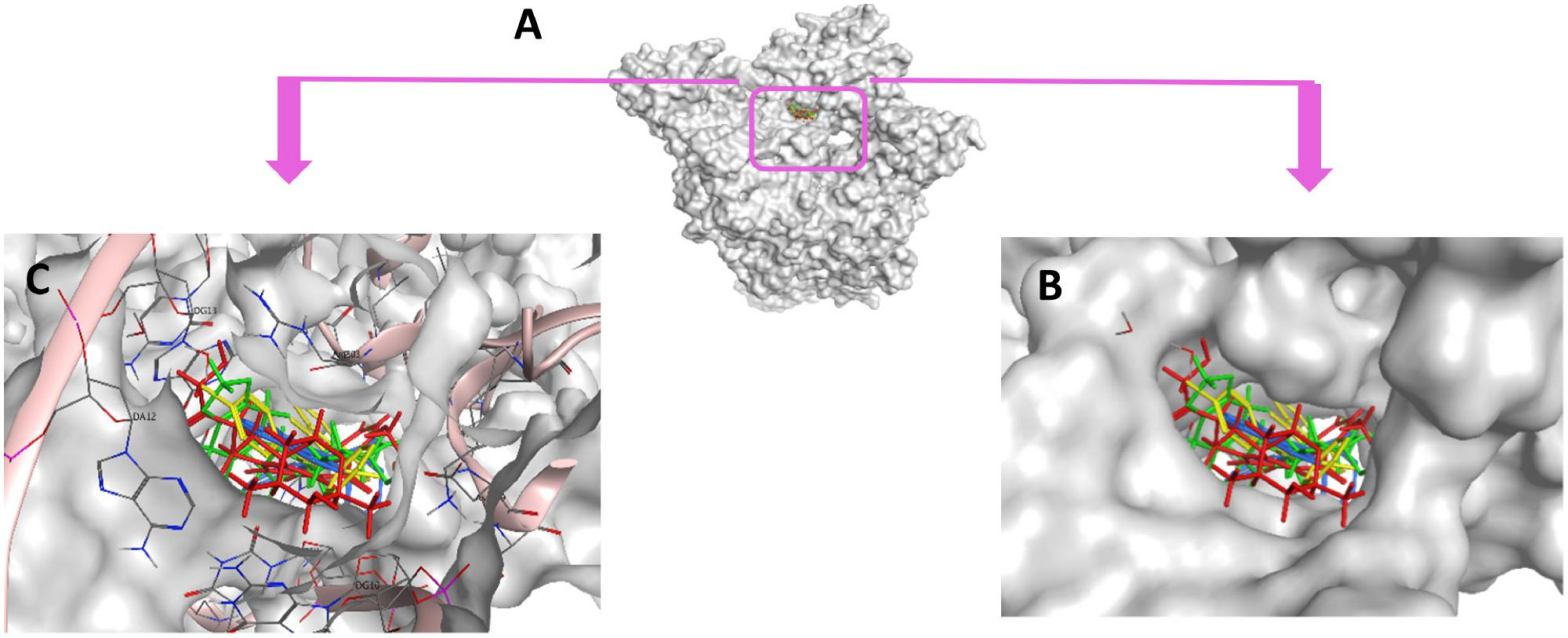
(A) Cartoon representation of the molecular surface of DNA-Topo IIβ complex (PDB:3QX3) showing **EVP** (green), **5b** (blue), **5h** (yellow), **doxorubicin** (red) in the binding pocket. (B) Focused view of the active site. (C) Transparent mode of the cartoon representation illustrating the binding pocket with the essential aminoacids and nucleotides.

Conclusively, docking of **5b** and **5h** along with the reference control **doxorubicin** to DNA-Topo IIβ complex was accurately achieved at the pocket of the active site of target protein in comparison to the co-crystallized ligand etoposide **EVP** (Figure 18). Thus, support the biological screening findings and declare the mechanism of action of these new candidates as topoisomerase inhibitors and as DNA intercalators.

#### In silico predication of physiochemical properties, pharmacokinetic profile and drug likeness optimization measures

The drug-likeness and pharmacokinetic properties were explicated for the active four compounds **5b**, **5e**, **5h**, and **5j** which can be supportive in the context of medicinal chemistry. We employed the automated SwissADME^59^ for the pharmacokinetics and drug-likeness predictions. The evaluations against passive human gastrointestinal absorption (GIA) and blood-brain barrier (BBB) permeation which give some parameters for the pharmacokinetics predictions were extracted from the BOILED-Egg model^59^^,^^60^. The compounds **5b** and **5e** are presented high as seen in Table 4. Also, all molecules were suggested to fail to permeate through BBB, and hence, they are expected to show low incidence for central nervous system (CNS) adverse effects. Permeability glycoprotein (P-gp) is proposed to be the most important member among ATP-binding cassette transporters or ABC-transporters^59^, since it is part of the resistance mechanisms against drugs^59^^,^^62^. All compounds were predicted to escape the P-gp indicating low incidence of developing resistance against human biological membranes.

**Table 4.** *In silico* predictions of the pharmacokinetics and drug-likeness properties for **5b**, ***5e***, ***5h,*** *and* ***5j***.

Code	MW	GIA	BBB	LogP	Veber #violations	PAINS #alerts
**5b**	431.51	High	No	5.79	0	1
**5e**	461.53	High	No	5.76	0	1
**5h**	465.95	Low	No	6.42	0	1
**5j**	495.98	Low	No	6.39	0	1
**Code**	**Pgp substrate**	**CYP1A2 inhibitor**	**CYP2C19 inhibitor**	**CYP2C9 inhibitor**	**CYP3A4 inhibitor**	
**5b**	No	Yes	Yes	Yes	No	
**5e**	No	Yes	Yes	Yes	Yes	
**5h**	No	Yes	Yes	No	No	
**5j**	No	No	Yes	Yes	No	

**GIA:** Human gastrointestinal absorption; **BBB:** blood-brain barrier permeation; **P-gp:** permeability glycoprotein; **CYP1A2, CYP2C19, CYP2C9, CYP2D6,** and **CYP3A4** are the five major isoforms of cytochromes P450 (CYP). **LogP** is calculated as XLOGP3 descriptor ^61^. **Veber #violations**counts the number of violations of Veber rule summarised as: NRB ≤ 10 and TPSA ≤ 140 Å^2^. **PAINS #**alertscounts the number of pan-assay interference compounds/substrucutres. All calculations were performed using SwissADME^59^.

Commonly, 50%–90% of therapeutic molecules are judged to be a substrate of at least one of the five major isoforms of Cytochrome P (CYP) enzymes (CYP1A2, CYP2C19, CYP2C9, CYP2D6, and CYP3A4)^59^^,^^63^^,^^64^. Inhibition of these isoenzymes is unquestionably one cause of pharmacokinetics-related drug-drug interactions leading to toxic or adverse effects^65^^,^^66^. As shown in (Table 4), all molecules were predicted to display inhibition of at least two of the CYP isoforms. This recommends administering these candidates in a sole regime and not in combination with other therapeutic agents. Fortunately, all molecules showed no alert to be a possible PAINS (pan-assay interference compounds)^67^, as shown in Table 4. This stresses that their chemical structures would not interfere in protein assays denoting the *in vitro* results to be robust with minimum artefacts. Lastly, drug-likeness properties underlined that all molecules obey Veber rules ^68^ and highlighting that these candidates would demonstrate good bioavailability profiles.

## Conclusion

In summary, a new series of imidazole‐2‐thiones derived by acenaphythylenones were synthesized and screened for their activity as anticancer agents by detection of their DNA damage using terbium fluorescent probe. Compounds **5b**, **5e**, **5h**, and **5j** pronouncedly induced DNA damage at different concentrations. Further, those compounds were evaluated for their *in vitro* anticancer activity against three cancer cell lines namely; hepatocellular carcinoma (HepG2), human breast adenocarcinoma (MCF-7) and human colon cancer (HCT-116) aided by **doxorubicin** as reference drug owing to its role in DNA damage. Using tumour management utilising microculture MTT assay, compounds **5b** and **5h** demonstrated the highest anticancer activity, with IC_50_ values of 5.105 and 2.65 μM, respectively, compared to the reference drug **doxorubicin** with IC_50_ value of 8.240 μM. In addition, compounds **5b** and **5h** showed a great safety margin against normal breast cell line (MCF-10a) with IC_50_ = 89.759 and 130.576 μM, respectively, better than **doxorubicin** (IC_50_ = 32.416 μM). Furthermore, those compounds exhibited high selectivity on the HepG2 and MCF-7 cell lines higher than **doxorubicin** (safer than **doxorubicin**). In addition, The DNA flow cytometry analysis of compounds **5b** and **5h** indicated that MCF‐7 cells revealed cell cycle arrest at S phase of the cell cycle profile with induction of apoptosis. It was observed that the percentage of late apoptosis was increased from 0.24% (control) to 22.69% and 28.52% upon exposure to compounds **5b** and **5h**, respectively. According to these results, the mechanism of action of the anticancer activity for compounds **5b** and **5h** is attributed to its apoptosis inducing activity in MCF-7 cells. Furthermore, the most active compounds examined as topoisomerase II inhibitors using **doxorubicin** as a positive control and the results showed that compound **5h** exhibited a potent inhibitory activity against topo II enzyme with IC_50_ value of 0.34 μM like that afforded by **doxorubicin** (IC_50_ = 0.33 μM). Analysing the molecular docking results on the active site of DNA-Topo IIβ complex showed fabulous binding mode and scoring values. Whereas the *in silico* physiochemical interpretations were consistent and robust. As a result, compounds **5b** and **5h** emerged as valuable lead compounds with a fruitful matrix that might be useful as antitumor agents, particularly as dual DNA intercalator topoisomerase inhibitors with safety profile.

## Experimental section

### Chemistry

#### Reagents and materials

All materials were obtained from commercial suppliers and used without further purification. Reactions were monitored by TLC (Kieselgel 60 F_254_ precoated plates, E. Merck, Germany), the spots were detected by exposure to UV lamp at 254 nm. Melting points were determined on an electrothermal melting point apparatus (Stuart Scientific Co.) and were uncorrected. NMR spectra were measured in DMSO-*d*_6_ on a Bruker AV-400 spectrometer (Bruker Bio Spin Corp., Billerica, MA, USA) (400 MHz for ^1^H, 101 MHz for ^13^C, and 40.15 MHz for ^15^N) at Florida Institute of Technology, USA. The ^1^H and ^13^C chemical shifts are given relative to internal standard TMS = 0, and external liquid ammonia = 0 for ^15^N. Coupling constants are stated in Hz. Correlations were established using ^1^H-^1^H COSY, and ^1^H-^13^C and ^1^H-^15^N HSQC and HMBC experiments. Vario EL III German CHN Elemental analyser model was used for Elemental analysis.

#### Synthesis of thiosemicarbazones 3a-f

An ethanolic solution of *N*-substituted-hydrazinecarbothioamides **2a-f** (0.01 mol) was added to an ethanolic solution (50 ml) containing acenaphthylene-1,2-dione (**1**, 0.01 mol) with 0.5 ml of triethylamine (Et_3_N). The mixture was refluxed for 3–6 h during which time a yellow precipitate separated out. The reaction mixture was then cooled to room temperature and the precipitate was filtered off and the obtained products **3a-f** were recrystallized.

*(Z)-N-Methyl-2-(2-oxo-1,2-dihydroacenaphthylen-1-ylidene)hydrazinecarbothioamide (****3a****)*: Yield: 80%; yellow crystals (EtOH); m.p. = 295 °C (m.p. = 293 °C, lit.^38^).

*(2-oxoacenaphthylen-1-(2H)-ylidene)-N-phenylhydrazinecarbothioamide (****3b****):* Yield: 83%; yellow crystals (MeOH); m.p. 212–214 °C (m.p.: 208–210 °C, lit.^39^).

*(Z)-N-Benzyl-2-(2-oxoacenaphthylen-1(2H)-ylidene)hydrazine-1-carbothioamide (****3c****):* Yield: 84%, yellow crystals, (EtOH), m.p.: 226–228 °C. IR (KBr, cm^−1^): ν = 3314 (str ΝΗ), 1607 (str C = N), 1023 (str C = S); ^1^H NMR (400 MHz, DMSO-d_6_): δ_H =_ 12.70 (s, 1H, NH), 9.94 (bs 1H, NH), 8.37 (d, J = 8.2; 1H), 8.16 (d, J = 8.3; 1H), 8.10 (d, J = 7.0; 1H), 8.00 (d, J = 7.0; 1H), 7.88 (dd, J = 8.0,7.2; 1H); 7.83 (dd, J = 8.2,7.2; 1H), 7.42 (d, J = 7.2; 2H), 7.37 (dd, J = 7.7.3; 2H), 7.28 (t, J = 7.1; 1H), 4.94 (d, J = 6.2; 2H); ^13^C NMR (100.1 MHz, DMSO-d_6_): δ_C_ = 188.5 (C-2), 178.0 (C-1c), 139.2 (C-8b), 138.4 (C-i), 137.5 (C-1), 132.8 (C-5), 130.4,130.0,129.9 (C-2a, 5a, 8a), 128.8 (C-7), 128.6 (C-4), 128.3 (C-m), 127.4 (C-o), 127.1 (C-p), 127.0 (C-6), 122.4 (C-3), 118.3 (C-8), 47.3 (C-1e). ^15^N NMR: (DMSO-d_6_): δ_N_ = 168.2 (N-1b), 122.3 (N-1d). C_20_H_15_N_3_OS (345.42): Calcd: C, 69.54; H, 4.38; N, 12.17; S, 9.28. Found: C, 69.67; H, 4.42; N, 12.25; S, 9.32.

(*Z)-N-cyclohexyl-2-(2-oxoacenaphthylen-1-(2H)-ylidene)hydrazinecarbothioamide (****3d****):* Yield: 75%; yellow crystals (CH_3_CN); m.p.: 202–204 °C (m.p.: 202–204 °C, lit.^40^).

*(Z)-N-(3'-Methoxyphenyl)-2-(2-oxoacenaphthylen-1(2H)-ylidene)hydrazine-1-carbothioamide (****3e****):* Yield: 84%, yellow crystals (EtOH), m.p.: 230–232 °C. ^1^H NMR (400 MHz, DMSO-*d*_6_): *δ*_H =_ 12.9 (s, 1H; NH), 10.91 (1H; NH), 8.41 (d, *J* = 8.2, 1H; H-5), 8.18 (d, *J* = 8.3, 1H; H-6), 8.17 (d, *J* = 7.2, 1H; H-3), 8.14 (d, *J* = 7.2, 1H; H-8), 7.92 (dd, *J* = 8.2,7.8, 1H; H-4), 7.88 (dd, *J* = 8.1,7.8, 1H; H-7), 7.36 (dd, *J* = 8.4,8.0, 1H), 7.34 (bs, 1H), 7.28 (bd, *J* = 8.0, 1H), 6.88 (dd, *J* = 8.2.3, 1H), 3.80 (s, 3H). ^13^C NMR (100.1 MHz, DMSO-*d*_6_): *δ_C_* = 188.6, 176.3, 159.2, 139.6, 139.4,137.2,132.8,130.4,129.9,129.1,128.9,128.6,127.3,122.5,118.9,117.5,111.6,111.3,55.2 (OCH3). ^15^N NMR: (DMSO-*d*_6_): *δ_N_* = 170.5 (N-1b), 132.0 (N-1d). Calcd: C, 66.47; H, 4.18; N, 11.63; S, 8.87. Found: C, 66.55; H, 4.22; N, 11.60; S, 8.9.

***(****Z)-N-Allyl-2-(2-oxoacenaphthylen-1-(2H)-ylidene)hydrazinecarbothioamide (****3f****):* Yield: 82%; light orange crystals (EtOH), m.p.: 190–191 °C (m.p. 193 °C, lit.^39^).

#### Synthesis of imdazole-2-thiones 5a-n

An ethanolic solution of *N*-substituted-thiosemicarbazones **3a-f** (1 mmol) was added to an ethanolic solution (20 ml) containing a mixture of **2a-c** (1 mmol) and **3a-c** (1 mmol) with 0.5 ml of Et_3_N. The mixture was refluxed for 8–12 h during which time a yellow precipitate separated out. The reaction was followed up by TLC analysis. The reaction mixture was then cooled to room temperature and the precipitate of **5a-n** was filtered off and the obtained products were recrystallized from the stated solvents.

*2-((3-Methyl-4-phenyl-2-thioxo-2,3-dihydro-1H-imidazol-1-yl)imino)acenaphthylen-1(2H)-one (****5a****)*: Yellow crystals, R_f_ = 0.4 (Toluene: EtOAc, 10:2), yield: 86%, m.p. 220–2 °C (EtOH). ^1^H NMR (400 MHz, DMSO-*d*_6_): *δ*_H =_ 8.60 (dd, 1H, *J* = 8.0,1.2 Hz, Ar-H), 8.40 (dd, 1H, *J* = 8.0,1.2 Hz, Ar-H), 8.25–8.20 (m, 2H, Ar-H), 7.55–7.62 (m, 2H, Ar-H), 7.25–7.30 (m, 5H, Ar-H), 6.82 ppm (s, 1H, imidazole-H-5), 3.60 ppm (s, 3H, CH_3_). ^13^C NMR (100.1 MHz, DMSO-*d*_6_): *δ_C_* = 189.1 (C = O, C-2), 176.1 (C = S, C-2′), 146.2 (C = N),141.5, 138.3,132.1,130.3,130.0,129.8 (Ar-C), 129.6,129.4 (Ar-CH), 129.1,129.0 (Ar-2CH), 128.5,128.3,128.2,127.9 (Ar-CH), 121.02 (Ar-CH), 104.1 ppm (imdazole-CH-5, C-5′), 34.3 ppm (CH_3_). MS (70 e.V., %): *m/z* = 370 (M + 1, 100), 269 (M^+^, 78), 289 (5), 180 (36), 154 (32). Calcd. For C_22_H_15_N_3_OS (369.44): C, 71.52; H, 4.09; N, 11.37; S, 8.68. Found: C, 71.70; H, 4.18; N, 11.50; S, 8.80.

*2-((3,4-Diphenyl-2-thioxo-2,3-dihydro-1H-imidazol-1-yl)imino)acenaphthylen-1(2H)-one (****5b****)*: Yellow crystals, R_f_ = 0.4 (Toluene: EtOAc, 10:3), yield: 90%, m.p. 240–2 °C (CH_3_CN). ^1^H NMR (400 MHz, DMSO-*d*_6_): *δ*_H =_ 8.53 (dd, 1H, *J* = 8.0,1.2 Hz, Ar-H), 8.26 (d, 2H, *J* = 8.0, Ar-H), 8.00–8.04 (m, 4H, Ar-H), 7.75–7.82 (m, 4H, Ar-H), 7.52–7.70 (m, 5H, Ar-H), 6.88 ppm (s, 1H, imidazole-H-5). ^13^C NMR (100.1 MHz, DMSO-*d*_6_): *δ_C_* = 189.0 (C = O, C-2), 178.4 (C = S, C-2′), 147.6 (C = N),140.2, 137.0,135.2,131.6,129.9,129.2,129.0 (Ar-C), 128.8,128.7,128.6,128.5,128.3,128.2,127.9 (Ar-2CH), 126.6,121.0 (Ar-CH), 104.8 ppm (imdazole-CH-5, C-5′). MS (70 e.V., %): *m/z* = 432 (M + 1, 100), 431 (M^+^, 61), 289 (5), 180 (37), 154 (57). Calcd. For C_27_H_17_N_3_OS (431.51): C, 75.15; H, 3.97; N, 9.74; S, 7.43. Found: C, 75.30; H, 3.80; N, 9.90; S, 7.50.

*2-((3-Benzyl-4-phenyl-2-thioxo-2,3-dihydro-1H-imidazol-1-yl)imino)-acenaphthylen-1(2H)-one (****5c****)*: Yellow crystals, R_f_ = 0.50 (Toluene: EtOAc, 10:2), yield: 88%, m.p. 212–4 °C (CH_3_OH). ^1^H NMR (400 MHz, DMSO-*d*_6_): *δ*_H =_ 8.26 (t, 1H, *J* = 7.5 Hz, Ar-H), 8.11 (d, 1H, *J* = 7.7 Hz, Ar-H), 7.99–8.03 (m, 2H, Ar-H), 7.81 (dd, 1H, *J* = 7.7 Hz, Ar-H), 7.56–7.52 (m, 5H, Ar-H), 7.36–7.22 (m, 4H, Ar-H), 7.18–7.15 (m, 2H, Ar-H), 6.94 (s, 1H, imidazole-H-5), 5.35 ppm (s, 2H, CH_2_-Bz). ^13^C NMR (100.1 MHz, DMSO-*d*_6_): *δ_C_* = 189.0 (C = O, C-2), 175.8 (C = S, C-2′), 164.0 (C = N), 146.9, 141.2, 138.4,136.2,131.9,131.6,129.9 (Ar-C), 129.8,129.7,129.1,129.0,128.9,128.6 (Ar-CH), 128.5,128.4,128.0,127.4 (Ar-2CH), 124.2,124.1 (Ar-CH), 104.0 (imdazole-CH-5, C-5′), 49.4 ppm (Bz-CH_2_). MS (70 e.V., %): *m/z* = 446 (M + 1, 57), 445 (M^+^, 27), 155 (33), 154 (100). Calcd. for C_28_H_19_N_3_OS (445.54): C, 75.48; H, 4.30; N, 9.43; S, 7.20. Found: C, 75.60; H, 4.40; N, 9.60; S, 7.30.

*2-((3-Cyclohexyl-4-phenyl-2-thioxo-2,3-dihydro-1H-imidazol-1-yl)imino)-acenaphthylen-1(2H)-one (****5d****)*: Yellow crystals, R_f_ = 0.35 (Toluene: EtOAc, 10:1), yield: 80%, m.p. 192–4 °C (CH_3_OH). ^1^H NMR (400 MHz, DMSO-*d*_6_): *δ*_H_ = 8.59 (d, 1H, *J* = 8.0 Hz, Ar-H), 8.27 (t, 1H, *J* = 7.2 Hz, Ar-H), 8.09–8.15 (m, 2H Ar-H), 7.70–7.84 (m, 2H, Ar-H), 7.55–7.65 (m, 5H, Ar-H), 6.80 (s, 1H, imidazole-H-5), 3.86–3.94 (m, 1H, cyclohexyl-H), 2.6–2.8 (m, 2H, cyclohexyl-H), 1.75–1.79 (m, 4H, cyclohexyl-H),1.27–1.45 (m, 2H, cyclohexyl-H),1.10–1.25 ppm (m, 2H, cyclohexyl-H). ^13^C NMR (100.1 MHz, DMSO-*d*_6_): *δ_C_* = 189.0 (C = O, C-2), 176.6 (C = S, C-2′), 145.6 (C = N), 138.3,133.4,133.2,132.0,130.1 (Ar-C), 129.7,129.3 (Ar-CH), 129.2 (Ar-C), 129.0,128.4,128.0,126.60 (Ar-2CH), 121.1 (Ar-CH), 105.1 (imdazole-CH-5, C-5′), 60.0 (CH), 27.7,25.4 (2CH_2_), 24.8 ppm (CH_2_). MS (70 e.V., %): *m/z* = 438 (M + 1, 100), 437 (M^+^, 53), 289 (7), 180 (44), 154 (45), 154 (100). Calcd. for C_27_H_23_N_3_OS (437.56): C, 74.11; H, 5.30; N, 9.60; S, 7.33. Found: C, 74.30; H, 5.40; N, 9.70; S, 7.50.

*2-((3-(3-Methoxyphenyl)-4-phenyl-2-thioxo-2,3-dihydro-1H-imidazol-1-yl)imino)-acenaphthylen-1(2H)-one (****5e****)*: Yellow crystals, R_f_ = 0.35 (Toluene: EtOAc, 10:2), yield: 86%, m.p. 212–4 °C (DMF/CH_3_OH: 2: 10). ^1^H NMR (400 MHz, DMSO-*d*_6_): *δ*_H_ = 8.10–8.07 (m, 1H, Ar-H), 7.90–7.85 (m, 2H, Ar-H), 7.80–7.75 (m, 2H, Ar-H), 7.45–7.40 (m, 2H, Ar-H), 7.30–7.25 (m, 5H, Ar-H), 7.22 (dd, 1H, *J* = 7.1,8.2 Hz, 2H), 7.15–7.10 (m, 1H, Ar-H), 6.90–6.86 (m, 2H, Ar-H), 6.82 (s, 1H, imidazole-H-5), 3.76 ppm (s, 3H, OCH_3_). ^13^C NMR (100.1 MHz, DMSO-*d*_6_): *δ_C_* = 189.0 (C = O, C-2), 176.3 (C = S, C-2′), 159.6 (Ar-*C*-OCH_3_), 147.7 (C = N), 140.5, 138.5,136.1,131.7,131.0,129.9 (Ar-C), 129.8,129.1 (Ar-CH), 128.9,128.6 (Ar-2CH), 128.3,127.7,126.8,124.1,121.12,120.5 (Ar-CH), 114.5 (Ar-CH-*p*), 114.4 (Ar-2CH), 104.7 (imdazole-CH-5, C-5′), 55.5 ppm (OCH_3_). MS (70 e.V., %): *m/z* = 462 (M + 1, 27), 461 (M^+^, 23), 155 (33), 154 (100). Calcd. for C_28_H_19_N_3_O_2_S (461.54): C, 72.87; H, 4.15; N, 9.10; S, 6.95. Found: C, 72.95; H, 4.30; N, 9.00; S, 7.12.

*2-((3-Allyl-4-phenyl-2-thioxo-2,3-dihydro-1H-imidazol-1-yl)imino)acenaphthylen-1(2H)-one (****5f****):* Yellow crystals, R_f_ = 0.45 (Toluene: EtOAc, 10:2), yield: 80%, m.p. 212–4 °C (EtOH). ^1^H NMR (400 MHz, DMSO-*d*_6_): *δ*_H_ = 8.25 (dd, 1H, *J* = 8.1,3.2 Hz, Ar-H), 8.03 (dd, 1H, *J* = 8.0,1.2 Hz, Ar-H), 7.78 (dd, 2H, *J* = 7.8 Hz, Ar-H), 7.59–7.49 (m, 2H, Ar-H), 7.33–7.26 (m, 5H, Ar-H), 6.62 (s, 1H, imidazole-H-5), 5.80–5.75 (m, 1H, Allyl-CH=), 4.97 (dd, 1 H, *J* = 7.1,5.2 Hz, Allyl-CH_2_=), 4.79 (dd, 1H, *J* = 7.1,5.2 Hz, Allyl-CH_2_=), 4.47 (ppm dt, 2H, *J* = 7.1,5.2 Hz, Allyl-CH_2_). ^13^C NMR (100.1 MHz, DMSO-*d*_6_): *δ_C_* = 189.1 (C = O, C-2), 175.8 (C = S, C-2′), 146.6 (C = N), 141.1, 138.4,132.0,131.9,131.6,130.0 (Ar-C), 129.8 (Allyl-CH=), 129.3,129.1,128.8,128.70,128.3,128.0 (Ar-CH), 126.6,124.2 (Ar-2CH), 121.1 (Ar-CH), 117.0 (Allyl-CH_2_=), 104.5 (imdazole-CH-5, C-5′), 48.6 ppm (Allyl-CH_2_). Calcd. for C_24_H_17_N_3_OS (395.48): C, 72.89; H, 4.33; N, 10.63; S, 8.11. Found: C, 73.00; H, 4.40; N, 10.80; S, 8.00.

*2-((4–(4-Chlorophenyl)-3-methyl-2-thioxo-2,3-dihydro-1H-imidazol-1-yl)imino)-acenaphthylen-1(2H)-one (****5 g****)*: Yellow crystals, R_f_ = 0.55 (Toluene: EtOAc, 10:2), yield: 76%, m.p. 262–4 °C (EtOH). ^1^H NMR (400 MHz, DMSO-*d*_6_): *δ*_H_ =8.57 (d, 1H, *J* = 6.9 Hz, H-5), 8.28 (d, 1H, *J* = 8.2 Hz, H-6), 8.06 (d, 1H, *J* = 8.4 Hz, H-3), 8.00 (d, 1H, *J* = 7.0 Hz, H-8), 7.82 (dd, 1H, *J* = 7.7,9.4 Hz, H-7), 7.78 (dd, 1H, *J* = 8.1,7.4 Hz, H-4), 7.66–7.62 (m, 4H; H-*o, m*), 6.93 (s, 1H; H-5′), 3.63 ppm (s, 3H; H-3a’). ^13^C NMR (100.1 MHz, DMSO-*d*_6_): *δ_C_* =189.1 (C = O, C-2), 176.1 (C = S, C-2′), 146.4 (C = N), 140.3, 138.4,134.4,132.1,131.6,131.0,130.0 (Ar-C), 129.4 (Ar-2CH, CH-*o*), 128.9 (Ar-2CH, CH-*m*), 128.8,128.6,128.0,126.5,124.4,121.0 (Ar-CH), 104.7 (imdazole-CH-5, C-5′), 34.3 ppm (*N*-CH_3_, C-3a’). IR (KBr, cm^−1^): ν = 1701 (C = O), 1576 (C = N), 1056 (C = S). MS (70 e.V., %): *m/z* = 405 (M + 2, 12), 404 (M + 1, 24), 403 (M^+^, 15), 307 (40), 289 (15), 180 (8), 155 (100), 137 (66). Calcd. for C_22_H_14_ClN_3_OS (403.88): C, 65.43; H, 3.49; Cl, 8.78; N, 10.40; S, 7.94. Found: C, 65.60; H, 3.40; Cl, 8.90; N, 10.50; S, 8.12.

*2-((4-(4-chlorophenyl)-3-phenyl-2-thioxo-2,3-dihydro-1H-imidazol-1-yl)imino)-acenaphthylen-1(2H)-one (****5h****)*: Yellow crystals, R_f_ = 0.25 (Toluene: EtOAc, 10:2), yield: 85%, m.p. 260–2 °C (CH_3_CN). ^1^H NMR (400 MHz, DMSO-*d*_6_): *δ*_H_ = 8.23 (d, *J* = 8.1, 1H; H-6/5), 7.97 (d, *J* = 6.8, 1H; H-8/3), 7.90 (d, *J* = 8.5, 1H; H-5/6), 7.79 (dd, *J* = 8.0,7.1, 1H; H-7/4), 7.74 (d, *J* = 7.0, 1H; H-3/8), 7.57 (“t”, 2H; H-*m’*), 7.51 (m, 3H; H-*o’, p’*), 7.44 (dd, *J* = 8.2,7.2, 1H; H-4/7), 7.39 (d, *J* = 8.6, 2H; H-*o*), 7.31 (d, *J* = 8.6, 2H; H-*m*), 7.12 ppm (s, 1H; H-5′). ^13^C NMR (100.1 MHz, DMSO-*d*_6_): *δ_C_* = 189.0 (C = O, C-2), 176.3 (C = S, C-2′), 147.8 (C = N), 139.3, 138.5, 137.0 133.7,131.9,131.6 (Ar-C), 130.5(Ar-2CH-, CH-*o*), 129.9,129.2 (Ar-C), 129.0,128.8,128.8 (Ar-CH), 128.6(Ar-2CH-, CH-*m*), 128.4 (Ar-2C), 128.3 (Ar-CH-*p’*), 127.9,126.7,124.1 (Ar-CH), 121.1 (Ar-2CH), 105.4 ppm (imdazole-CH-5, C-5′). IR (KBr, cm^−1^): ν =1698 (C = O, 1082 (C = S). MS (70 e.V., %): *m/z* = 467 (M + 2, 6), 466 (M + 1, 12), 465 (M^+^, 9), 289 (13), 180 (3), 155 (32), 154 (100). Calcd. for C_27_H_16_ClN_3_OS (465.96): C, 69.60; H, 3.46; Cl, 7.61; N, 9.02; S, 6.88. Found: C, 69.80; H, 3.60; Cl, 7.80; N, 9.19; S, 6.94.

*2-((3-Benzyl-4-(4-chlorophenyl)-2-thioxo-2,3-dihydro-1H-imidazol-1-yl)imino)-acenaphthylen-1(2H)-one (****5i****)*: Yellow crystals, R_f_ = 0.35 (Toluene: EtOAc, 10:2), yield: 79%, m.p. 260–2 °C (DMF: EtOH, 2:10). IR (KBr, cm^−1^): ν =1697 (C = O, 1070 (C = S). ^1^H NMR (400 MHz, DMSO-*d*_6_): *δ*_H_ =8.24 (d, *J* = 8.2, 1H; H-6/5), 8.13 (d, *J* = 7.0, 1H; H-3/8), 8.00 (d, *J* = 8.6, 1H; H-5/6), 7.98 (d, *J* = 8.6, 1H; H-8/3), 7.80 (dd, *J* = 8.0,7.2, 1H; H-7/4), 7.58 (dd, *J* = 8.2,7.2, 1H; H-4/7), 7.55 (d, *J* = 8.4, 2H; H-*o*), 7.49 (d, *J* = 8.6, 2H; H-*m*), 7.32 (dd, *J* = 7.6,7.2, 2H; H-*m’*), 7.24 (t, *J* = 7.2, 1H; H-*p’*), 7.15 (d, *J* = 7.3, 1H; H-*o’*), 6.98 (s, 1H; H-5′), 5.35 ppm (s, Bz-CH_2_). ^13^C NMR (100.1 MHz, DMSO-*d*_6_): *δ_C_* =189.0 (C = O, C-2), 175.9 (C = S, C-2′), 147.1 (C = N), 139.9, 138.4,136.1,134.6,131.9,131.6 (Ar-C), 130.9 (Ar-2CH-*o*), 129.9,129.9 (Ar-C), 129.2,128.9 (Ar-CH), 128.6(Ar-2CH*-m*), 128.5,128.0,127.4,126.6 (Ar-CH), 126.3,126.6 (Ar-2CH), 121.1 (Ar-CH-*p*), 105.3 (imdazole-CH-5, C-5′), 49.5 ppm (Bz-CH_2_, C-3a’). MS (70 e.V., %): *m/z* = 481 (M + 2, 3), 480 (M + 1, 7), 479 (M^+^, 5), 180 (3), 155 (32), 154 (100). Calcd. for C_28_H_18_ClN_3_OS (479.98): C, 70.07; H, 3.78; Cl, 7.39; N, 8.75; S, 6.68. Found: C, 70.20; H, 3.82; Cl, 7.50; N, 8.90; S, 6.72.

*2-((4-(4-Chlorophenyl)-3-(3-methoxyphenyl)-2-thioxo-2,3-dihydro-1H-imidazol-1-yl)-imino)acenaphthylen-1(2H)-one (****5j****)*: Yellow crystals, R_f_ = 0.3 (Toluene: EtOAc, 10:1), yield: 92%, m.p. 282–4 °C (DMF: EtOH, 2:10). ^1^H NMR (400 MHz, DMSO-*d*_6_): *δ*_H_ =8.24 (d, *J* = 8.1, 1H; H-6/5, Ar-H), 7.99 (d, *J* = 8.1, 1H; H-5/6, Ar-H), 7.98 (d, *J* = 6.5, 1H; H-8/3, Ar-H), 7.87 (d, *J* = 7.0, 1H; H-3/8, Ar-H), 7.79 (dd, *J* = 7.7,9.3, 1H; H-7/4, Ar-H), 7.46 (dd, *J* = 8.1,7.3, 1H; H-4/7, Ar-H), 7.43 (dd, *J* = 8.1,8.0, 1H; H-5”, Ar-H), 7.41 (d, *J* = 8.6, 2H; H-*o*, Ar-H), 7.34 (d, *J* = 8.6, 2H; H-*m*, Ar-H), 7.21 (dd, *J* = 2.0,2.0, 1H; H-2”), 7.11 (s, imdazole-H-5, 1H; H-5′), 7.08 (dd, *J* = 8.2,4.1, 1H; H-6”, Ar-H), 6.98 (dd, *J* = 6.1,2.0, 1H; H-4”, Ar-H), 3.78 ppm (s, 3H, Ar-OCH_3_). ^13^C NMR (100.1 MHz, DMSO-*d*_6_): *δ_C_* =189.0 (C = O, C-2), 176.2 (C = S, C-2′), 159.7 (Ar-*C*-OCH_3_), 147.9 (C = N), 139.2, 138.5, 138.0,133.7,131.9,131.6,130.7,130.0 (Ar-C), 129.9,129.0 (Ar-2CH), 128.4(Ar-2CH-, CH-*m*), 128.2,128.0,126.8,124.1,121.1,120.5,114.6,114.5 (Ar-CH), 105.3 (imdazole-CH-5, C-5′), 55.5 ppm (OCH_3_, C-3a”). IR (KBr, cm^−1^): *nν* = 1704 (C = O, 1577 (C = N), 1086 (C = S). MS (70 e.V., %): *m/z* = 496 (M + 1, 42), 495 (M^+^, 24), 289 (16), 180 (10), 155 (32), 154 (100). Calcd. for C_28_H_18_ClN_3_O (495.98): C, 67.81; H, 3.66; Cl, 7.15; N, 8.47; S, 6.46. Found: C, 67.93; H, 3.72; Cl, 7.30; N, 8.60; S, 6.60.

*2-((3-Allyl-4-(4-chlorophenyl)-2-thioxo-2,3-dihydro-1H-imidazol-1-yl)imino)-acenaphthylen-1(2H)-one (****5k****)*: Yellow crystals, R_f_ = 0.4 (Toluene: EtOAc, 10:1), yield: 84%, m.p. 262–4 °C (DMF:EtOH, 1:10).^1^H NMR (400 MHz, DMSO-*d*_6_): *δ*_H_ =8.49 (d, 1H, *J* = 7.0 Hz, Ar-H), 8.28 (d, 1H, *J* = 8.2 Hz, Ar-H), 8.06 (d, 1H, *J* = 8.4 Hz, Ar-H), 8.00 (d, 1H, *J* = 6.9 Hz, Ar-H), 7.82 (dd, 1H, *J* = 7.6,7.6 Hz, Ar-H), 7.76 (dd, 1H, *J* = 7.8 Hz, Ar-H), 7.62 (**AB**, 2H, *J* = 8.6 Hz, Ar-H-*o*), 7.50 (**AB**, 2H, Ar-H-*m*), 6.94 (s, 1H, imidazole-H-5), 5.99 (ddt, H, *J*_d_= 17.1,10.2 Hz, *J*_t_ = 5.0 Hz; Allyl-CH=), 5.21 (d, 1H, *J* = 10.4 Hz; Allyl-CH_2_), 5.03 (d, 1H, *J* = 17.4 Hz; allyl-CH_2_), 4.71 (d, 2H, *J* = 4.1 Hz, allyl-CH_2_). ^13^C NMR (100.1 MHz, DMSO-*d*_6_): *δ_C_* =189.0 (C = O, C-2), 175.7 (C = S, C-2′), 146.7 (C = N), 139.8, 138.5,134.6,132.0,131.9,131.6 (Ar-C), 131.0(Ar-2CH-*o*), 130.0 (Ar-C), 129.3 (Allyl-CH=), 128.9(Ar-2CH-*m*), 128.7 (Ar-H), 128.6 (Ar-2CH), 126.6,124.2,121.1 (Ar-CH), 117.0 (Allyl-CH_2_=), 105.1 (imidazole-CH-5, C-5′), 48.6 (Allyl-CH_2_-). IR (KBr, cm^−1^): ν =1699 (C = O, 1089 (C = S). MS (70 e.V., %): *m/z* = 430 (M + 1, 82), 429 (M^+^, 40), 180 (45), 154 (100). Calcd. for C_24_H_16_ClN_3_OS (429.92): C, 67.05; H, 3.75; Cl, 8.25; N, 9.77; S, 7.46. Found: C, 67.15; H, 3.82; Cl, 8.30; N, 9.90; S, 7.54.

*2-((3-Methyl-4-(naphthalen-2-yl)-2-thioxo-2,3-dihydro-1H-imidazol-1-yl)imino)-acenaphthylen-1(2H)-one (****5 l****)*: Yellow crystals, R_f_ = 0.4 (Toluene: EtOAc, 10:2), yield: 78%, m.p. 240–2 °C (EtOH: hexane, 5: 10). ^1^H NMR (400 MHz, DMSO-*d*_6_): *δ*_H_ = 8.59 (d, 1H, *J* = 7.0 Hz), 8.28 (d, 1H, *J* = 8.1 Hz), 8.20 (bs, 1H, Ar-H), 8.08–8.04 (m, 5H, Ar-H), 7.82 (dd, 1H, *J* = 8.0,7.3 Hz), 7.78 (dd, 1H, *J* = 8.2,7.2 Hz, Ar-H), 7.73 (dd, 1H, *J* = 8.1,4.6 Hz), 7.65–7.63 (m, 2H, Ar-H), 7.00 (s, 1H, s, 1H, imidazole-H-5), 3.70 ppm (s, 3H, (*N*-CH_3_, H-3a’). ^13^C NMR (100.1 MHz, DMSO-*d*_6_): *δ_C_* =189.1 (C = O, C-2), 176.1 (C = S, C-2′), 146.3 (C = N), 141.5, 138.3,133.0,132.6,132.1,131.58 (Ar-CH), 130.0,129.5,128.8,128.7,128.4,128.3,128.0,127.7 (Ar-C), 127.3,127.2,127.0,126.5,126.1,124.4,121.0 (Ar-CH), 104.5 imidazole-CH-5, C-5′), 34.5 ppm (CH_3_(*N*-CH_3_, C-3a’). IR (KBr, cm^−1^): ν =1691 (C = O, 1070 (C = S). MS (70 e.V., %): *m/z* = 420 (M + 1, 20), 419 (M^+^, 15), 289 (15), 155 (33), 154 (100). Calcd. for C_26_H_17_N_3_OS (419.50): C, 74.44; H, 4.08; N, 10.02; S, 7.64. Found: C, 74.60; H, 4.18; N, 10.12; S, 7.80

*2-((3-Benzyl-4-(naphthalen-2-yl)-2-thioxo-2,3-dihydro-1H-imidazol-1-yl)imino)-acenaphthylen-1(2H)-one (****5 m****):* Yellow crystals, R_f_ = 0.3 (Toluene: EtOAc, 10:3), yield: 83%, m.p. 332–4 °C (DMF/CH_3_OH, 10: 3). ^1^H NMR (400 MHz, DMSO-*d*_6_): *δ*_H_ = 8.25 (d, 1H, *J* = 8.2 Hz, Ar-H), 8.15 (d, 1H, *J* = 7.0, Ar-H), 8.08 (bs, 1H, Ar-H), 8.05–8.03 (m, 4H, Ar-H), 7.93 (dd, 1H, *J* = 6.2, 6.0 Hz, Ar-H), 7.80 (dd, 1H, *J* = 7.6,7.5 Hz, Ar-H), 7.60 (m, 4H, Ar-H), 7.30 (dd, 2H, *J =* 7.5,7.2 Hz, Ar-H), 7.23 (t, 1H, *J* = 7.3 Hz, Ar-H), 7.17 (d, 2H, *J* = 7.4 Hz, Ar-H), 7.05 (s, 1H s, 1H, imidazole-H-5), 5.42 ppm (s, 2H, bz-CH_2_). ^13^C NMR (100.1 MHz, DMSO-*d*_6_): *δ_C_* =189.0 (C = O, C-2), 175.9 (C = S, C-2′), 146.9 (C = N), 141.1, 138.4,132.9,132.4,131.9,131.5,129.9,129.0,128.9 (Ar-C), 128.5,128.4 (Ar-2CH), 128.3,128.1,127.9,127.6,127.3,127.2 (Ar-CH), 127.1,126.9,126.5 (Ar-CH), 126.2 (Ar-2CH), 125.8,124.2 (Ar-CH), 121.0 (Ar-CH-*p*), 105.0 (imidazole-CH-5) 49.5 ppm (bz-CH_2_). MS (70 e.V., %): *m/z* = 496 (M + 1, 37), 495 (M^+^, 23), 289 (16), 180 (10), 155 (32), 154 (100). Calcd. for C_32_H_21_N_3_OS (495.60): C, 77.55; H, 4.27; N, 8.48; S, 6.47. Found C, 77.70; H, 4.40; N, 8.60; S, 6.53.

*2-((3-Allyl-4-(naphthalen-2-yl)-2-thioxo-2,3-dihydro-1H-imidazol-1-yl)imino)-acenaphthylen-1(2H)-one (****5n****)*: Yellow crystals, R_f_ = 0.2 (Toluene: EtOAc, 10:3), yield: 69%, m.p. 230–2 °C (EtOH). IR (KBr, cm^−1^): ν =1701 (C = O, 1068 (C = S). ^1^H NMR (400 MHz, DMSO-*d*_6_): *δ*_H_ = 8.51 (d, 1H, *J* = 7.0 Hz, Ar-H), 8.28 (d, 1H, *J* = 8.1 Hz, Ar-H), 8.15 (bs, 1H, Ar-H), 8.07–8.04 (m, 5H, Ar-H), 7.83 (dd, 1H, *J* = 8.0,7.1 Hz, Ar-H), 7.76 (dd, 1H, *J* = 8.2,7.2 Hz, Ar-H), 7.72–7.65 (m, 3H, Naphthyl-H), 7.01 (s, 1H, imidazole-H-5), 6.02 (ddt, 1H, *J*_d_= 17.1,10.1, *J*_t_ = 5.0 Hz, Allyl-CH=), 5.23 (dd, 1H, *J* = 10.0,5.9 Hz, Allyl-CH=), 5.07 (dd, 1H, *J* = 17.1,2.0 Hz, Allyl-CH=), 4.78 (d, 2H, *J* = 4.2 Hz, Allyl-CH_2_). ^13^C NMR (100.1 MHz, DMSO-*d*_6_): *δ_C_* =189.0 (C = O, C-2), 175.7 (C = S, C-2′), 146.7 (C = N), 141.0, 139.8, 138.5,134.6,132.0,131.9,131.6 (Ar-C), 131.0 (Ar-2CH), 130.0 (Ar-C), 129.3 (Allyl-CH=), 128.9,128.7 (Ar-2CH), 128.6 128.3,128.0,126.6,126.4,124.2,121.1 (Ar-CH), 117.0 (Allyl-CH_2_=), 105.1 (imidazole-CH-C-5′), 48.6 (Allyl-CH_2_). Calcd. for C_28_H_19_N_3_OS (445.54): C, 75.48; H, 4.30; N, 9.43; S, 7.20. Found: C, 75.60; H, 4.35; N, 9.60; S, 7.30.

### Biological evaluation

All chemicals, solvents, media and kits were purchased from commercial suppliers.

#### DNA interaction studies

*Fluorescence measurements:* Fluorescence spectra were measured between 290 and 800 nm with excitation at 270 nm of 100 µL solutions in a 1 cm path length Suprasil quartz fluorescence cuvette.

The PerkinElmer LS 55 fluorescence spectrophotometer was used to measure the fluorescence. After 24 h of incubation in buffer solution (40 mM Tris, 10 mM NaCl, pH 7.5), aliquots of each DNA-Compound mixture were combined with TbCl_3_ to confirm DNA damage. The final concentrations were 1 g/mL ctDNA, 3 mM TbCl_3_, and 50 M of each compound in buffer solution (40 mM Tris, 10 mM NaCl, pH 7.5). Calf thymus DNA (ctDNA) was combined with various quantities of each substance, ranging from 0.1 pM to 200 M, for the experiment. The ultimate concentrations of 1 g/mL ctDNA and 3 mM were achieved by adding Tb3 + solution to the ctDNA/compound combinations in buffer solution (40 mM Tris, 10 mM NaCl, pH 7.5) after 24 h of incubation.^44^

#### In vitro anticancer screening

The anticancer effect of the most active synthesized compounds **5b, 5e**, **5h**, and **5j** was determined against three cancer cell lines namely: hepatocellular carcinoma (HepG2), human breast adenocarcinoma (MCF-7) and human colon cancer (HCT-116) utilising MTT assay^48^ (Supplementary file). Compounds **5b** and **5h** were screened for their effects on the normal breast cell line MCF‐10a. Cells at a density of 1 × 104 were seeded in a 96‐well plate at 37 °C for 48 h under 5% CO_2_. After incubation, the cells were treated with different concentrations of the prepared molecules and incubated for 24 h. MTT dye was added after 24 h of drug treatment and incubated for 4 h at 37 °C. Also, 100 μl of dimethyl sulphoxide was added to each well to dissolve the purple formazan formed. The colour intensity of the formazan product, which represents the growth condition of the cells, was quantified using an enzyme‐linked immunosorbent assay (ELISA) plate reader at 570 nm. The experiments were carried out with at least three replicates and they were repeated at least three times.

#### Annexin V-FITC/PI apoptosis induction analysis

The BioVision® annexin-V-FITC apoptosis detection kit was used to detect apoptosis in MCf-7 cells as per the manufacturer’s instructions. Apoptosis was then quantified by flow cytometry at 488 nm using the FITC signal detector (typically FL1) and PI staining by the phycoerythrin emission signal detector (typically FL2). Briefly, 1–5 × 10^5^ cells were were quickly centrifuged and collected. Compounds 5b and 5h were applied to cells for 24 h at their IC_50_ concentrations before they were resuspended in 500 μl of binding buffer. Annexin-V-FITC and PI were added. After that, they were kept at room temperature for five minutes in the dark. An exclusive signal detector was used to examine the binding of annexin-V to FITC.^50^

*Cell cycle analysis:* A FACS Calibur flow cytometer was used to measure the amount of DNA in the cell cycle analysis at 488 nm in accordance with the manufacturer’s recommendations. Briefly, chosen compounds **5b** and **5h** were applied to 2 × 10^5^ cells/well for 24 h at their IC_50_ concentrations. Cells were treated, rinsed twice, and then re-suspended in ice-cold PBS (phosphate buffer saline). After washing, 0.7 ml of 100% ethanol was added, and the mixture was then allowed to sit at 20 °C for 20 min. After washing, 500 μl of RNase was added, and then the mixture was incubated for 30 min. After that, PI was added, and the mixture was incubated without light for 30 min^22^^,^^51^.

#### Statistical analysis of the data

Data were fed to the computer and analysed using IBM SPSS software package version 20.0. **(**Armonk, NY: IBM Corp**)**. Quantitative data were expressed as mean and standard error One way **ANOVA test** was used for comparing the four studied groups and followed by **Post Hoc test (Tukey)** for pairwise comparison. Significance of the obtained results was judged at the 5% level (Supplementary file).

#### In vitro topoisomerase II inhibitory assay

The most active anti-proliferative members were analyzed for their Topo II inhibitory activities. **Doxorubicin** was used as a positive control. Compounds **5b** and **5h** were selected to be evaluated against topo II [MBS#942146] using human DNA topoisomerase II-β (TOPII-β) ELISA kit according to manufacturer’s instructions^56^ (Supplementary file).

### In silico studies

#### Molecular docking (Supplementary file)

Computer-aided docking experiments were performed using Molecular Operating Environment (MOE 2020.09)^57^ software (Chemical Computing Group, Montreal, Canada). The crystallographic structure of Human topoisomerase II beta in complex with DNA and etoposide (PDB ID: 3QX3) was retrieved from Protein Data Bank.

By adding hydrogens, calculating partial charges, and minimising energy using the Amber 10: EHT Force Field with a root mean square deviation (RMSD) gradient of 0.1 kcal/mol, the database of tested compounds was obtained. The downloaded protein was further processed by removing repetitive chains while maintaining certain nucleotides and water molecules that are important for ligand binding. The MOE QuickPrep methodology was then used to optimise structural issues, perform 3D protonation, and calculate partial charges. The test compounds’ best score values and advantageous binding conformations were determined using the MOE Dock application’s default procedure, which uses the triangle matcher placement approach with London dG as the primary scoring function. To further optimise the induced fit receptor technique, a force field-based scoring function (GBVI/WSA dG) was used to choose poses that displayed the protein’s maximum hydrogen-bond, ionic, and hydrophobic interactions. The energy scores in kcal/mol for the complexes generated between the ligand conformers and the binding sites were included in the output database. Finally, visual inspection and analysis of the interactions with active site residues were performed on the generated docking poses. The default position was the one with the highest score and the best ligand-enzyme interaction. Visual inspection and analysis of interactions with active site residues were done on the docking poses that were obtained. The default position was the one with the highest score and the best ligand-enzyme interaction.

#### In silico predication of physiochemical properties, pharmacokinetic profile and drug likeness optimization measures

The *in silico* predictions of the pharmacokinetics properties were performed using SwissADME^59^ for the pharmacokinetics and drug-likeness predictions. The evaluations against (GIA) and (BBB) permeation which give some parameters for the pharmacokinetics predictions were extracted from the BOILED-Egg model^59^^,^^60^.

## Supplementary Material

Supplemental Material

## Data Availability

Data set generated during and/or analysed during the current study are available from the corresponding author on reasonable request.
