# Supplementary material for: New imidazole-2-thiones linked to acenaphythylenone as dual DNA intercalators and topoisomerase II inhibitors: structural optimization, docking, and apoptosis studies
Source: J Enzyme Inhib Med Chem. 2024 Mar 15;39(1):2311818. doi: 10.1080/14756366.2024.2311818 (PMC10946275; doi:10.1080/14756366.2024.2311818)
Supplement: Supplemental Material [file IENZ_A_2311818_SM3160.pdf]

New imidazole-2-thiones linked to acenaphthylenone as dual DNA intercalators and topoisomerase II inhibitors: structural optimization, docking, and apoptosis studies

Asmaa H. Mohamed,[1] Mohammed B. Alshammari,[2]* Ashraf A. Aly,[1*] Kamal U. Sadek,[1] Akil Ahmad,[2] Eman A. Aziz,[1] Amira F. El-Yazbi,[3] Eman J. El-Agroudy,[4] Marwa E. Abdelaziz[4]

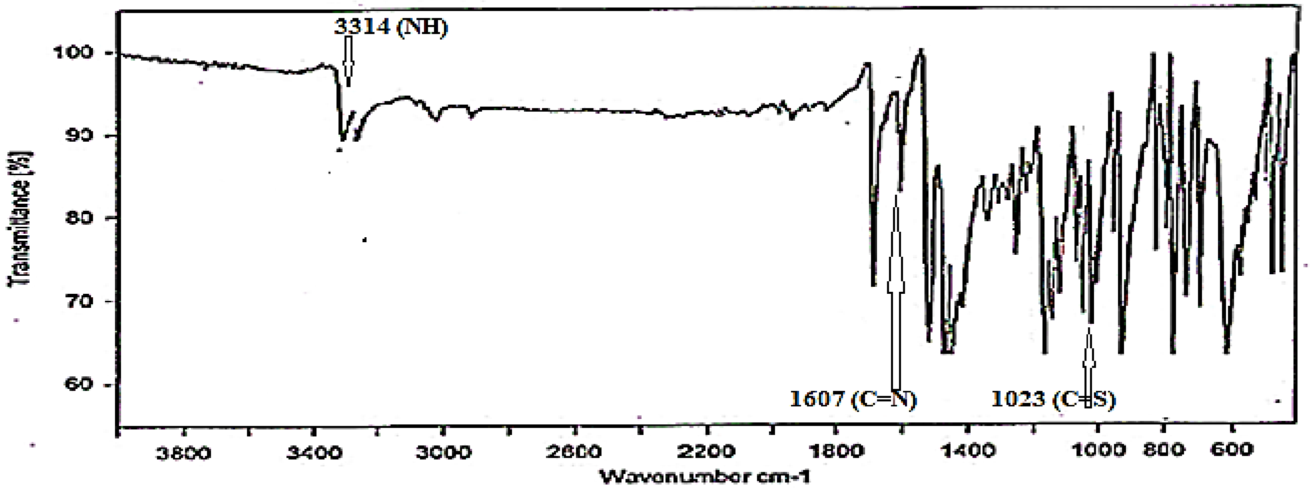

SI Figure 1. IR spectrum of compound **3c**

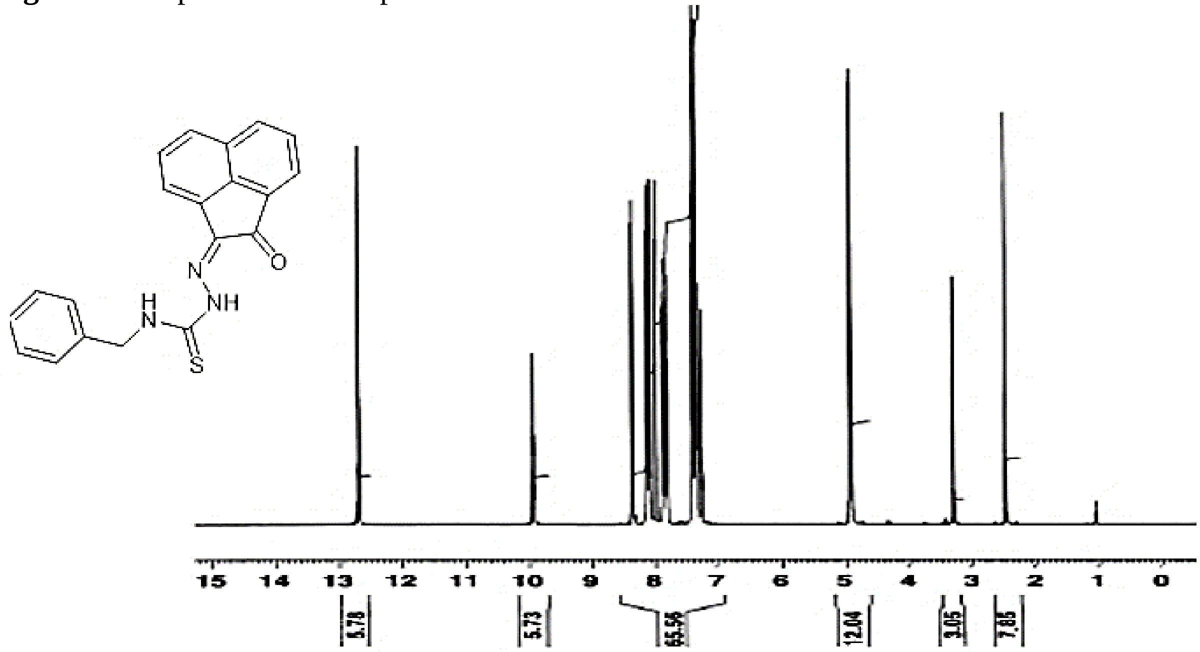

SI Figure 2. ^{1}H NMR spectrum of compound **3c**.

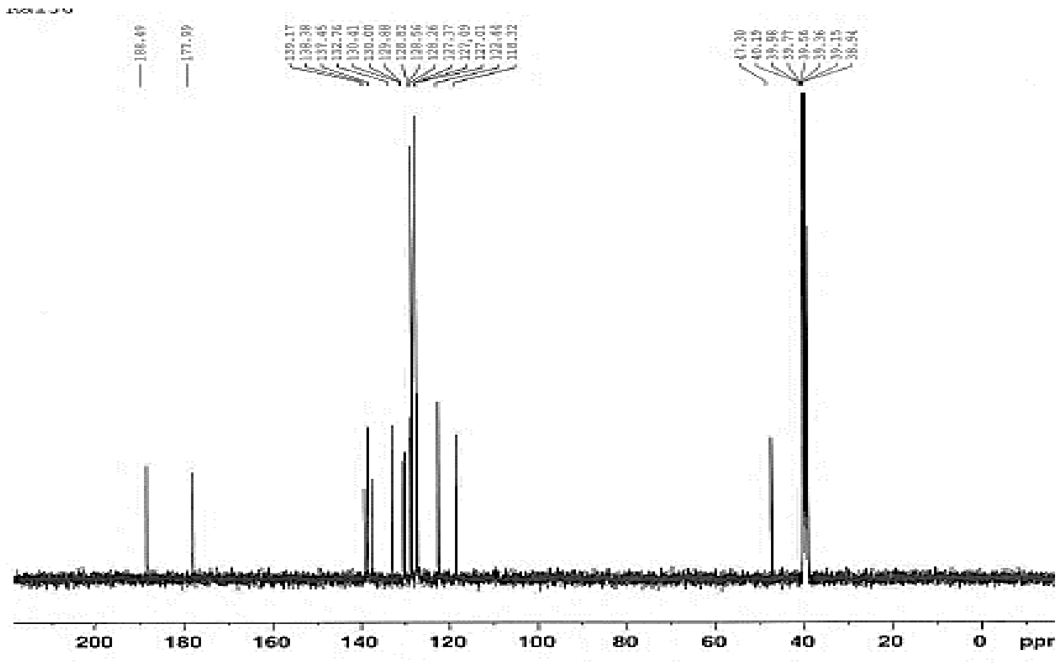

SI Figure 3. ^{13}C NMR spectrum of **3c**

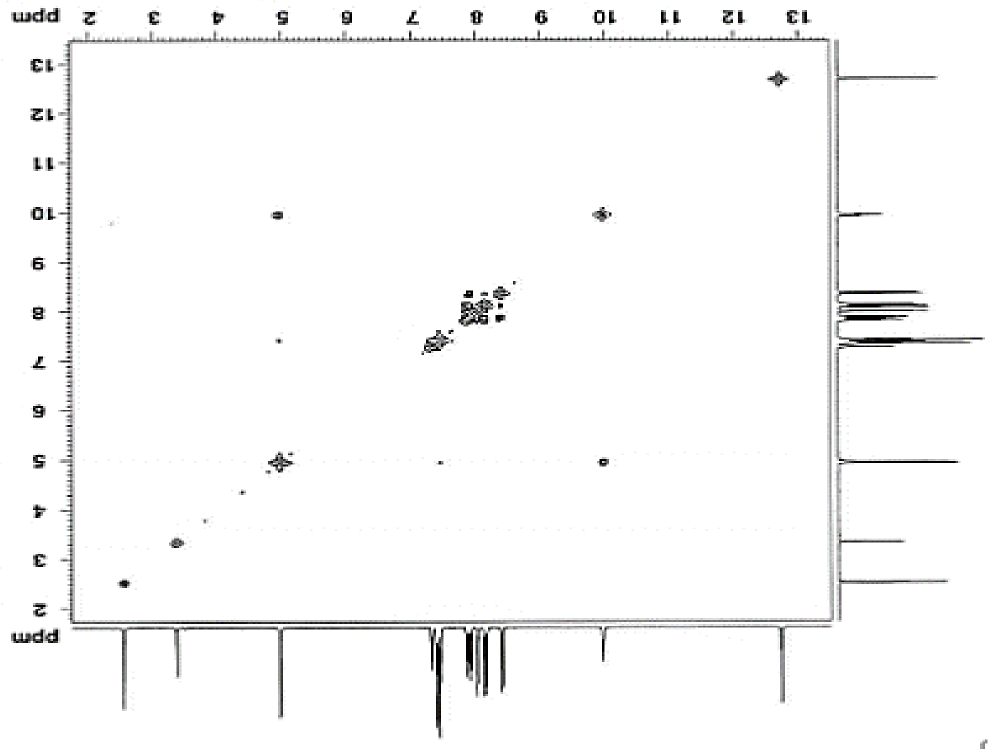

SI Figure 4. COSY H H spectrum of **3c**

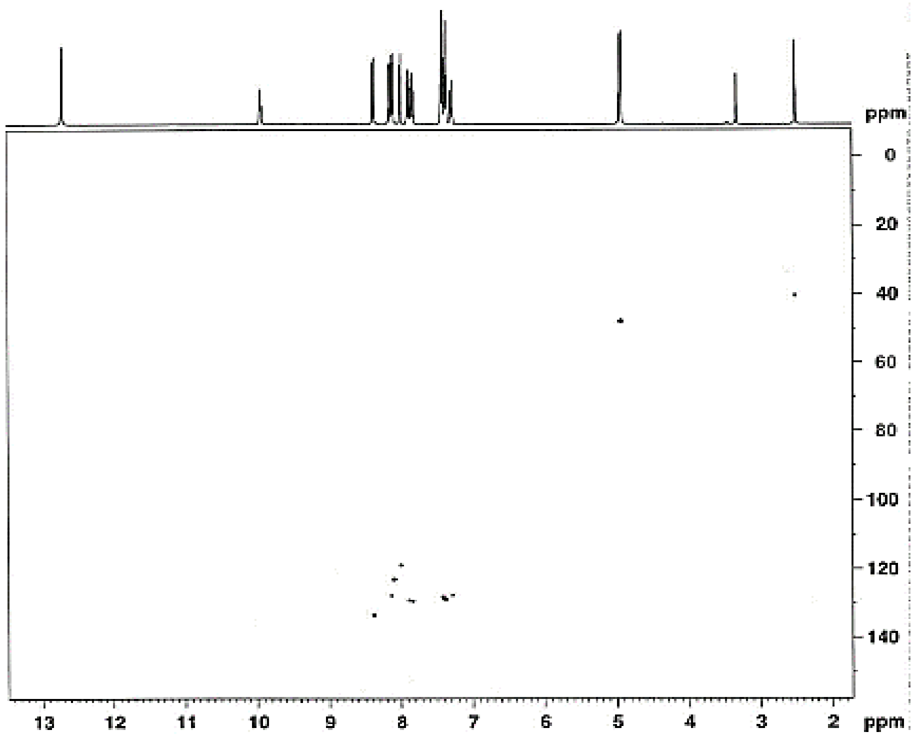

SI Figure 5. ^{1}H ^{13}C HSQC spectrum **3c**

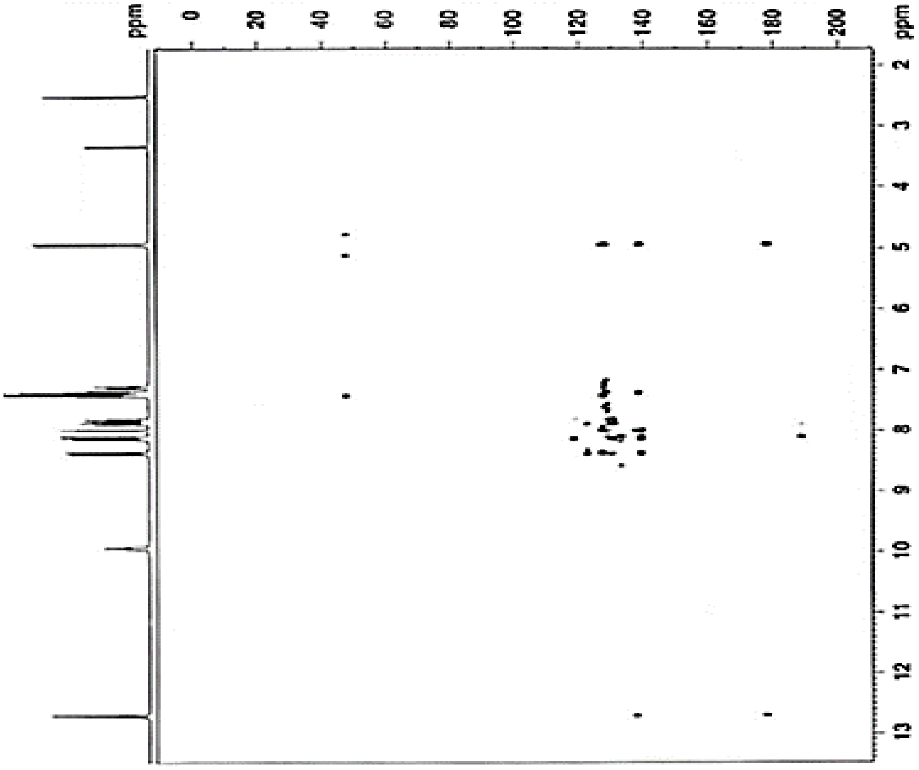

SI Figure 6. ^{1}H ^{13}C-HMBC spectrum **3c**

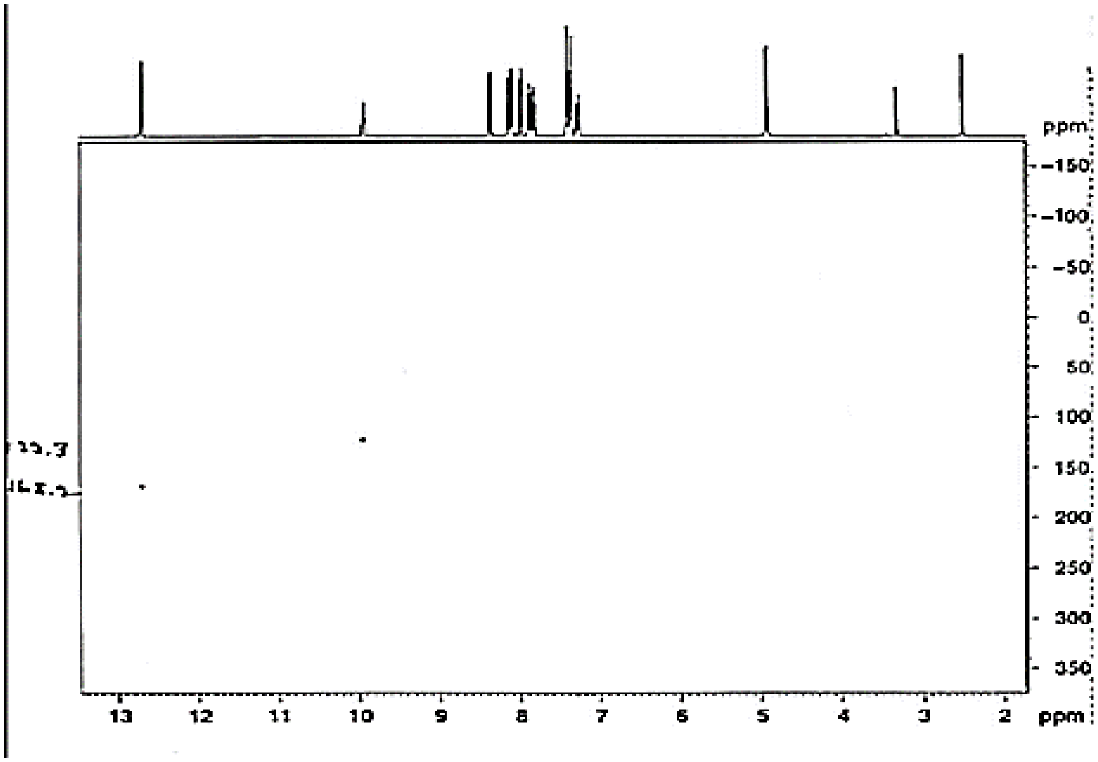

SI Figure 7. ^{1}H ^{15}N HSQC spectrum **3c**

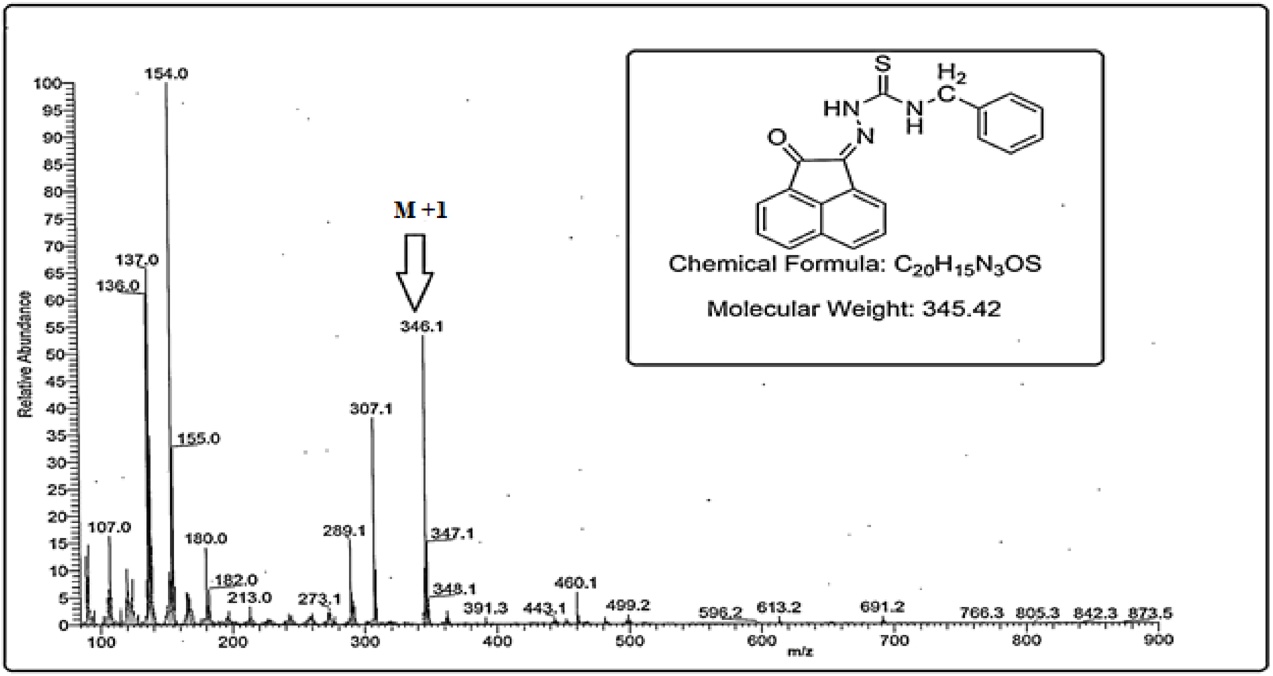

SI Figure 8. Mass spectroscopy of compound **3c**.

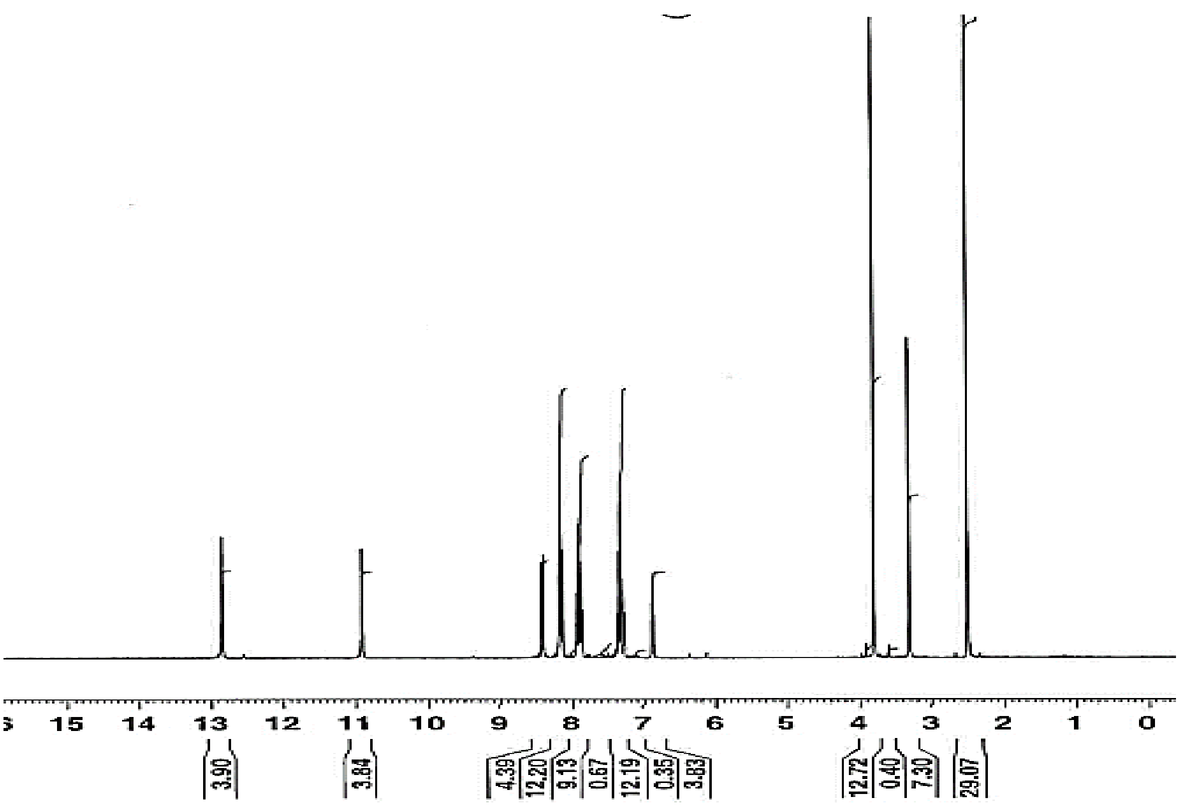

SI Figure 9. ^{1}H NMR spectrum of **3e**

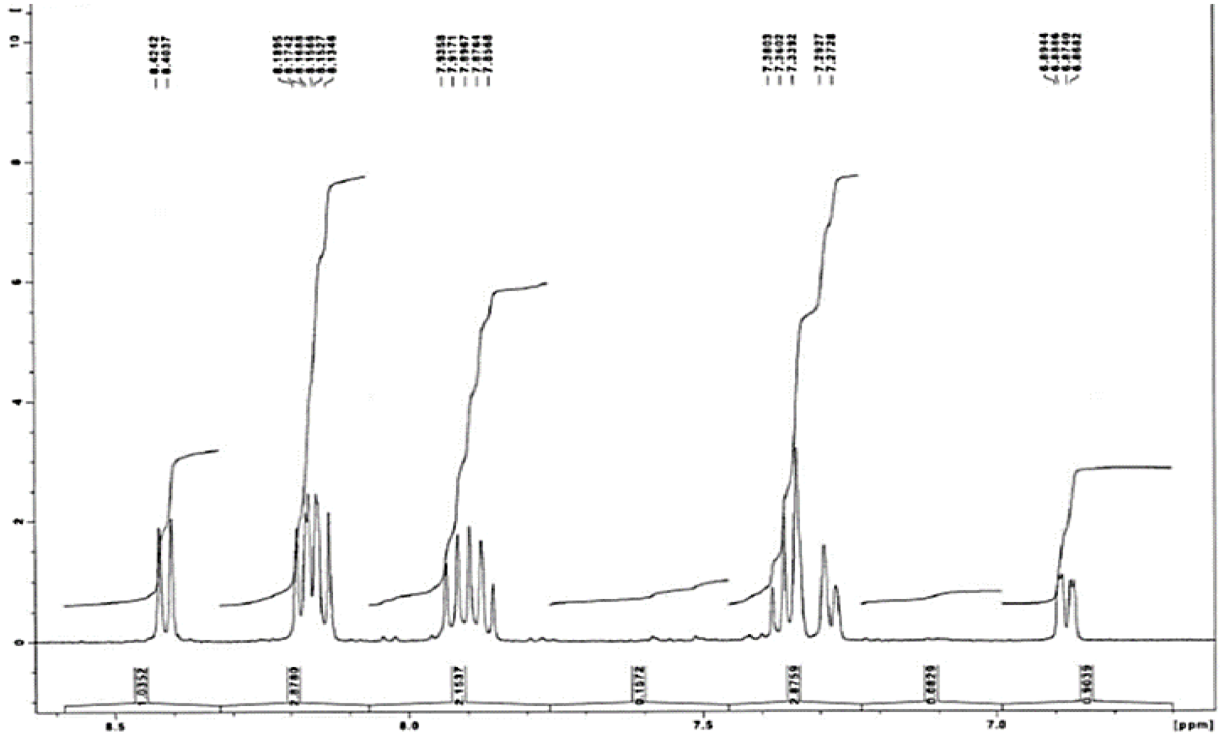

SI Figure 10. Expanded ^{1}H NMR spectrum of **3e**

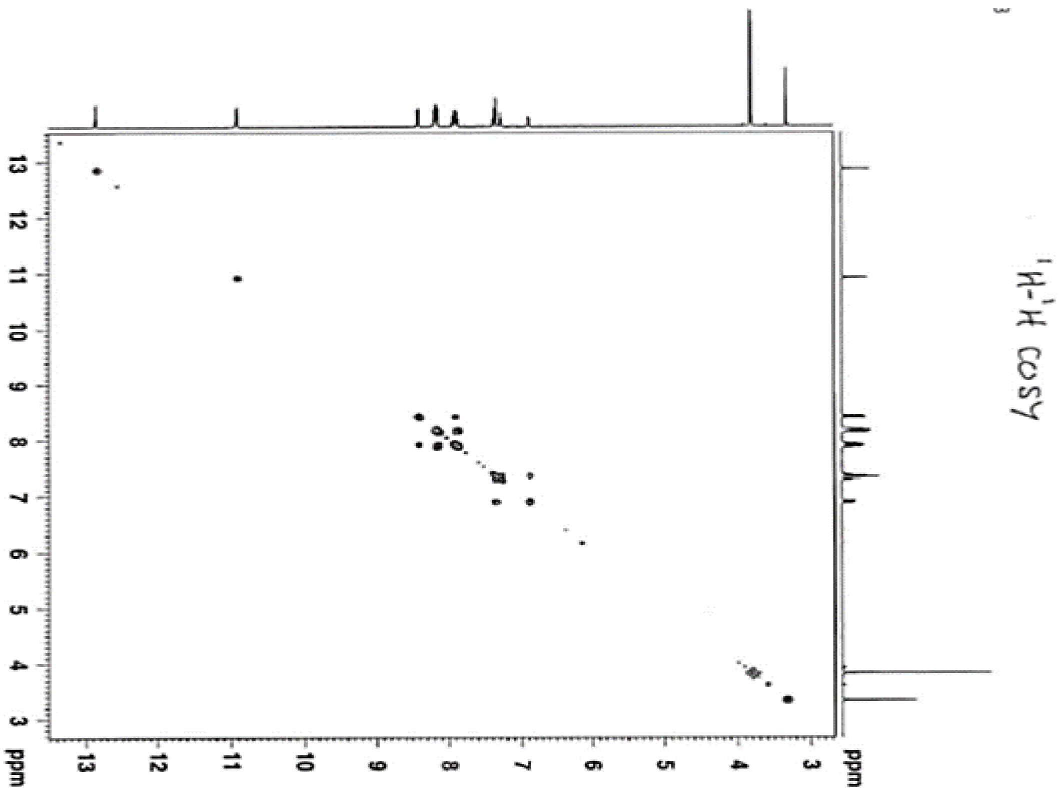

SI Figure 11. COSY H H spectrum of **3e**

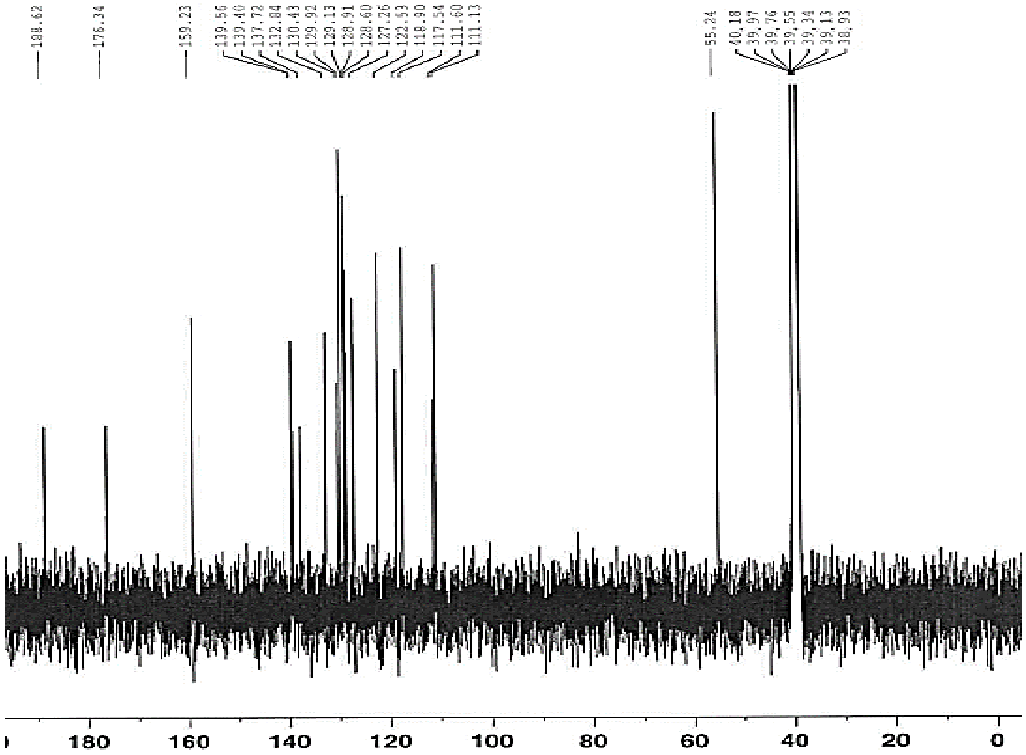

SI Figure 12. ^{13}C NMR spectrum of **3e**

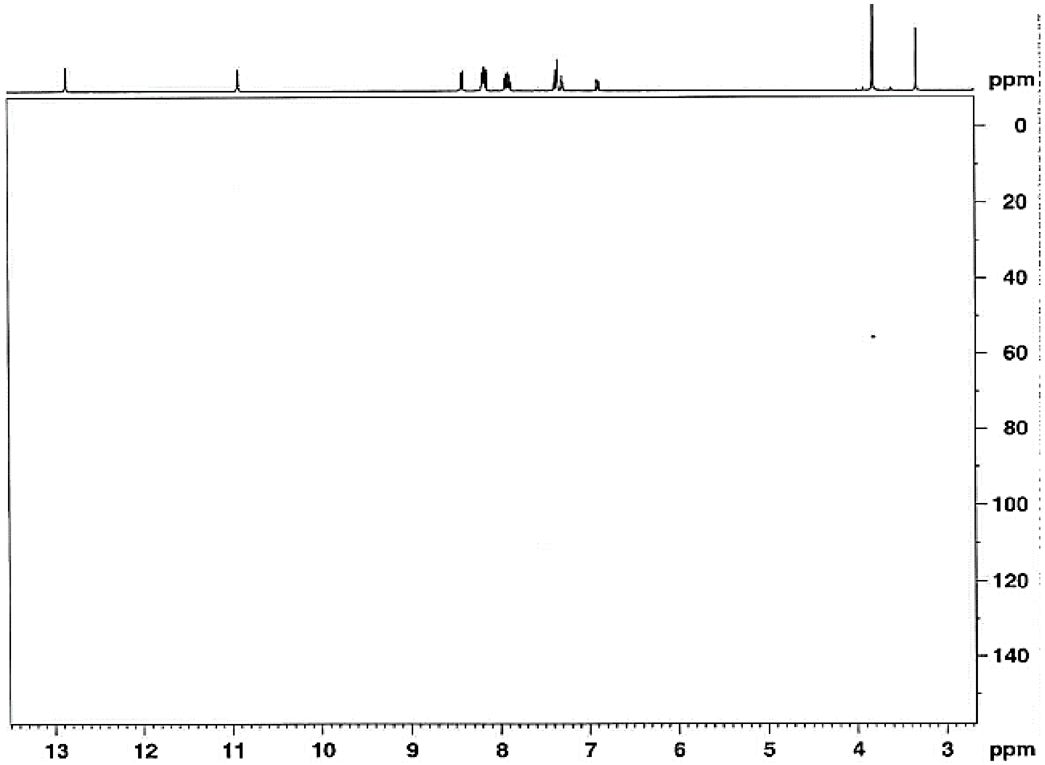

SI Figure 13. ^{1}H ^{13}C HSQC spectrum **3e**

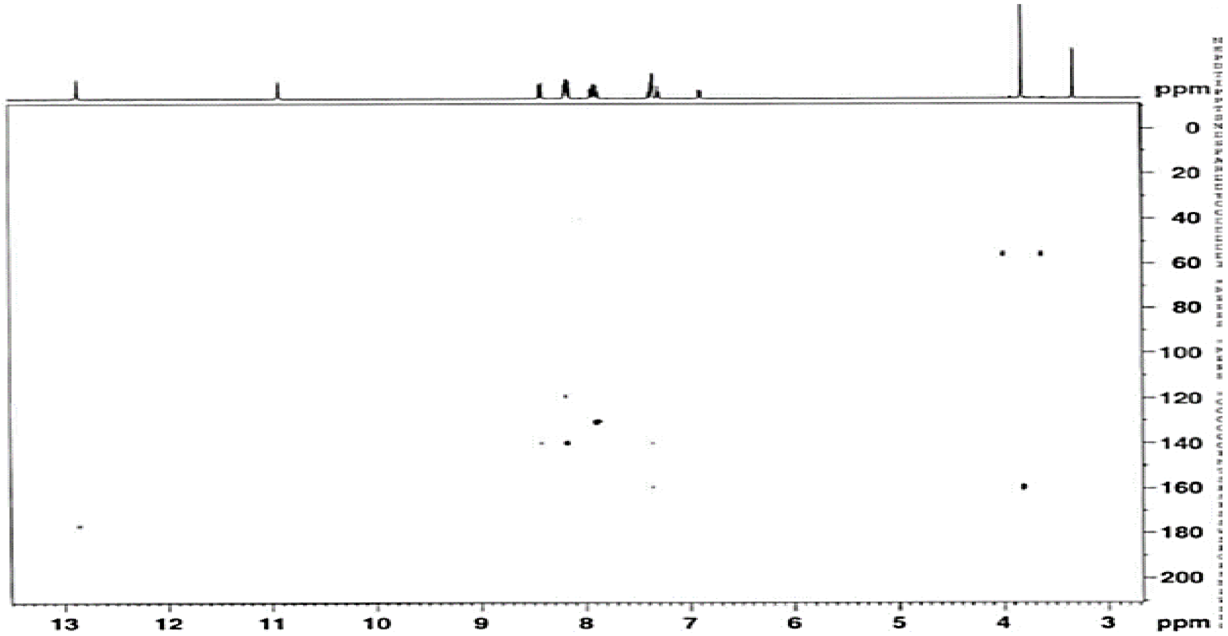

SI Figure 14. ^{1}H ^{13}C-HMBC spectrum **3e**

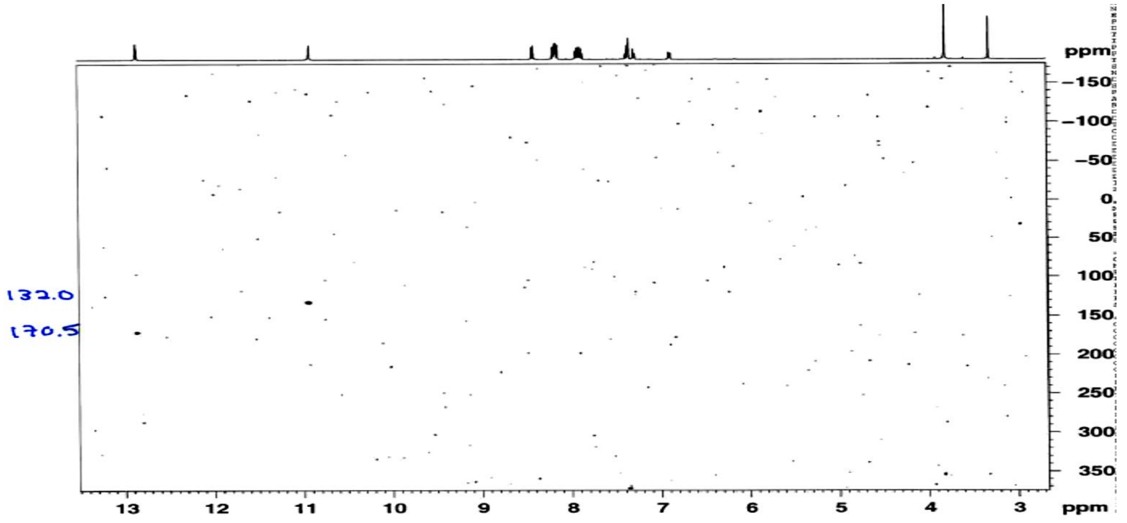

SI Figure 15. ^{1}H ^{15}N HSQC spectrum **3e**

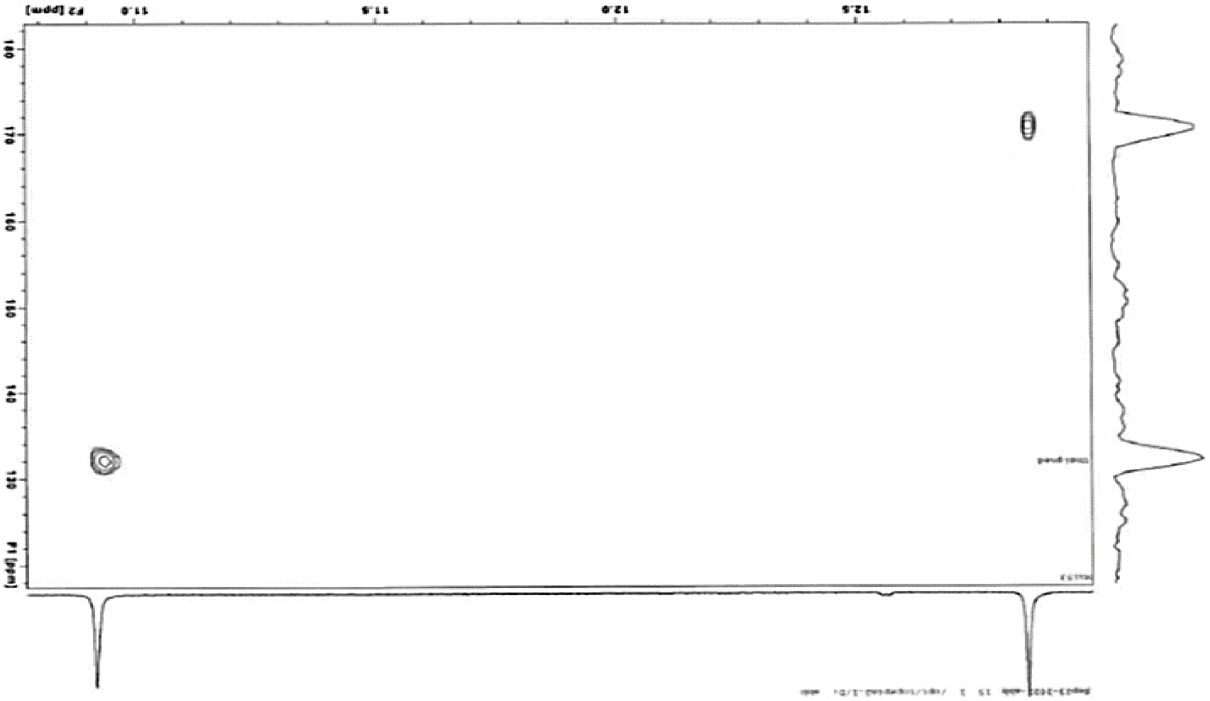

SI Figure 16. Expanded ^{1}H ^{15}N HSQC spectrum **3e**

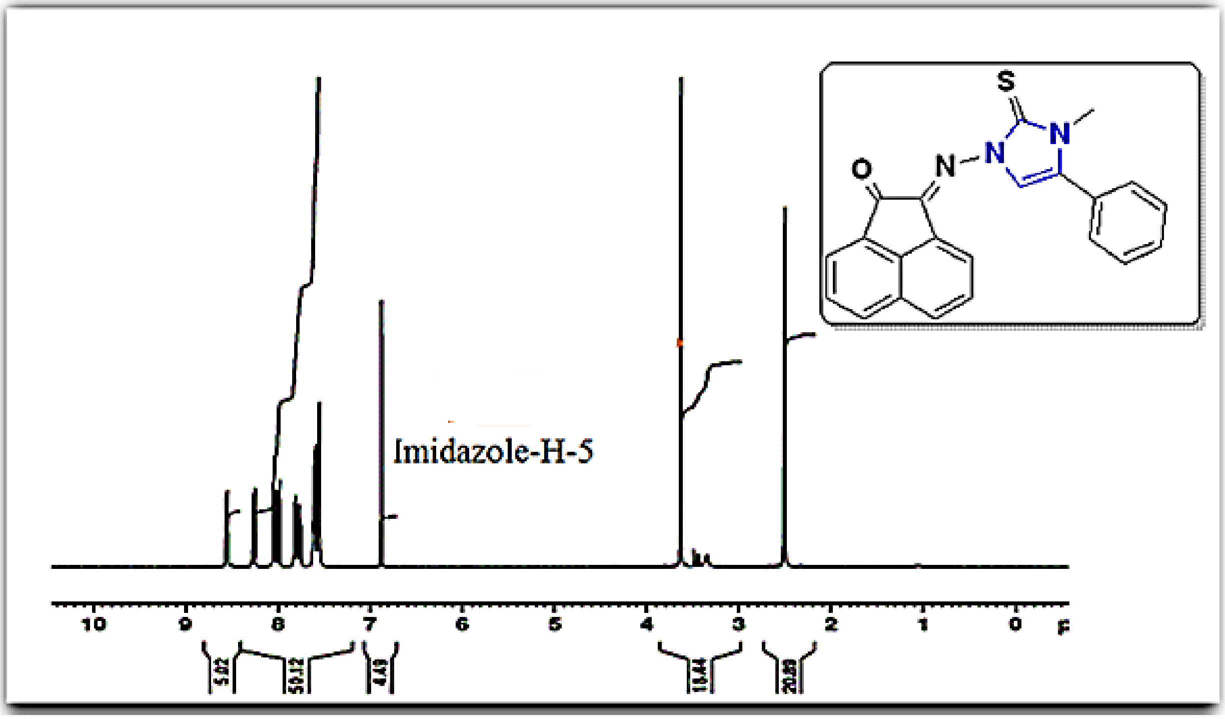

SI Figure 17. ^{1}H NMR spectrum of **5a**

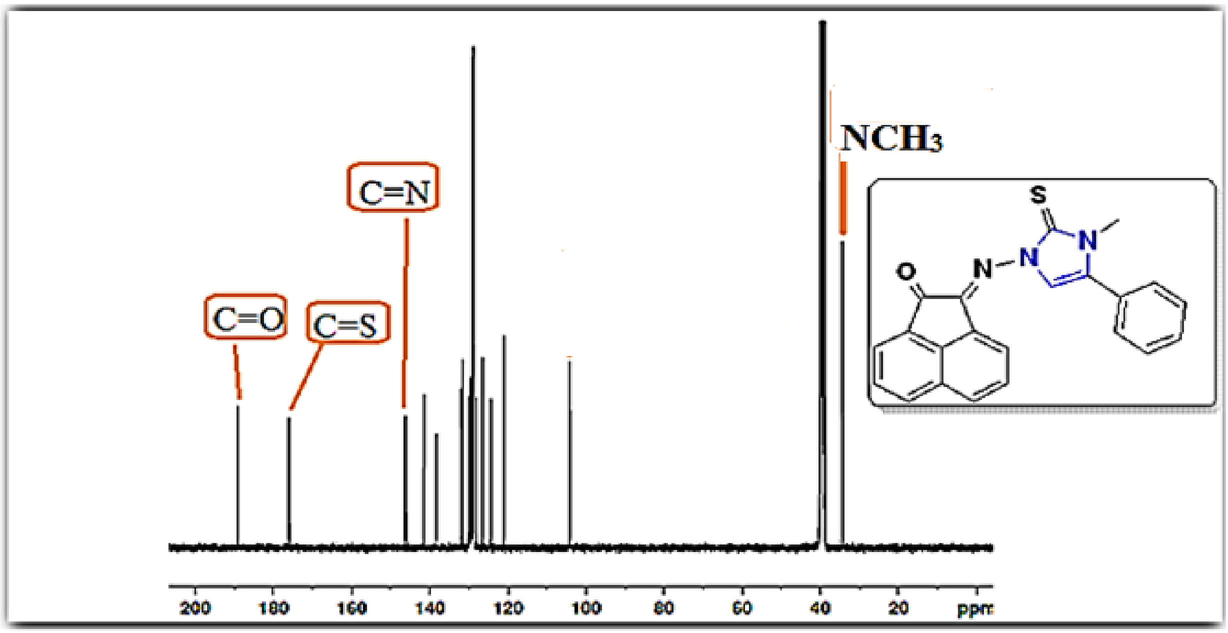

SI Figure 18. ^{13}C NMR spectrum of **5a**

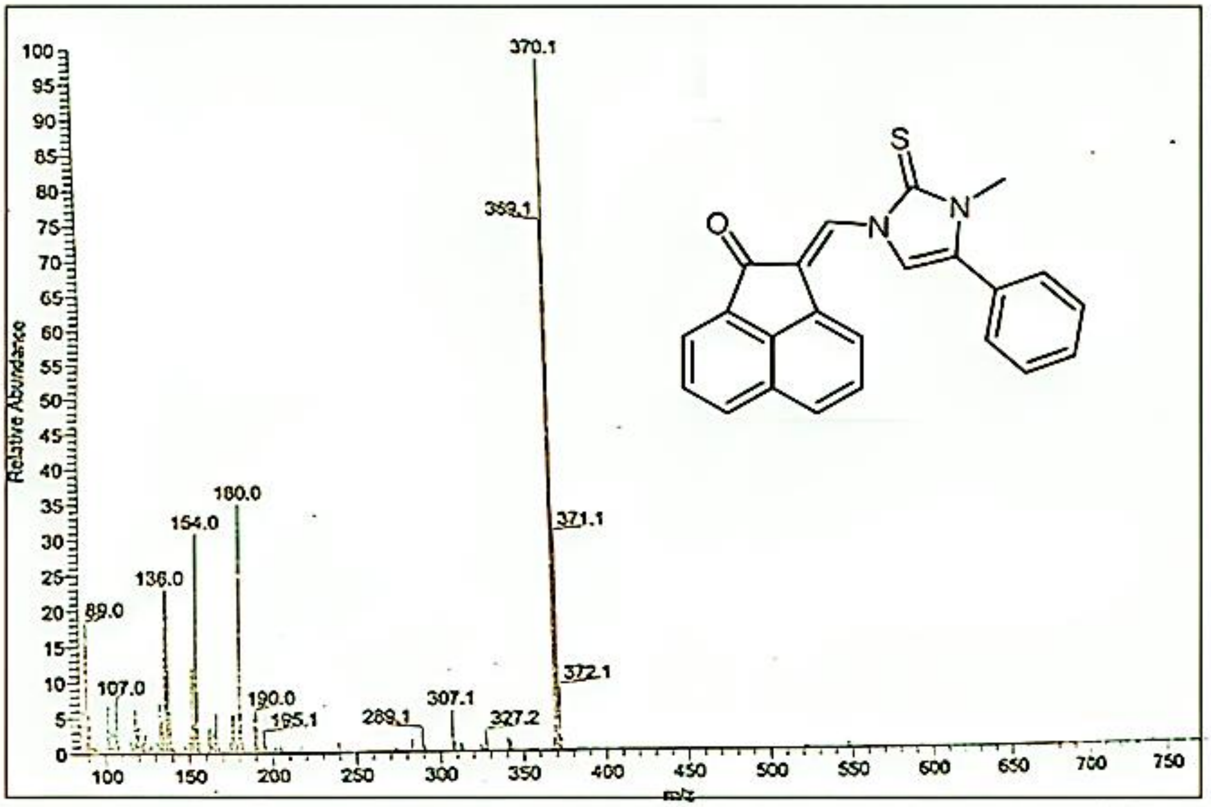

SI Figure 19. Mass spectroscopy of **5a**

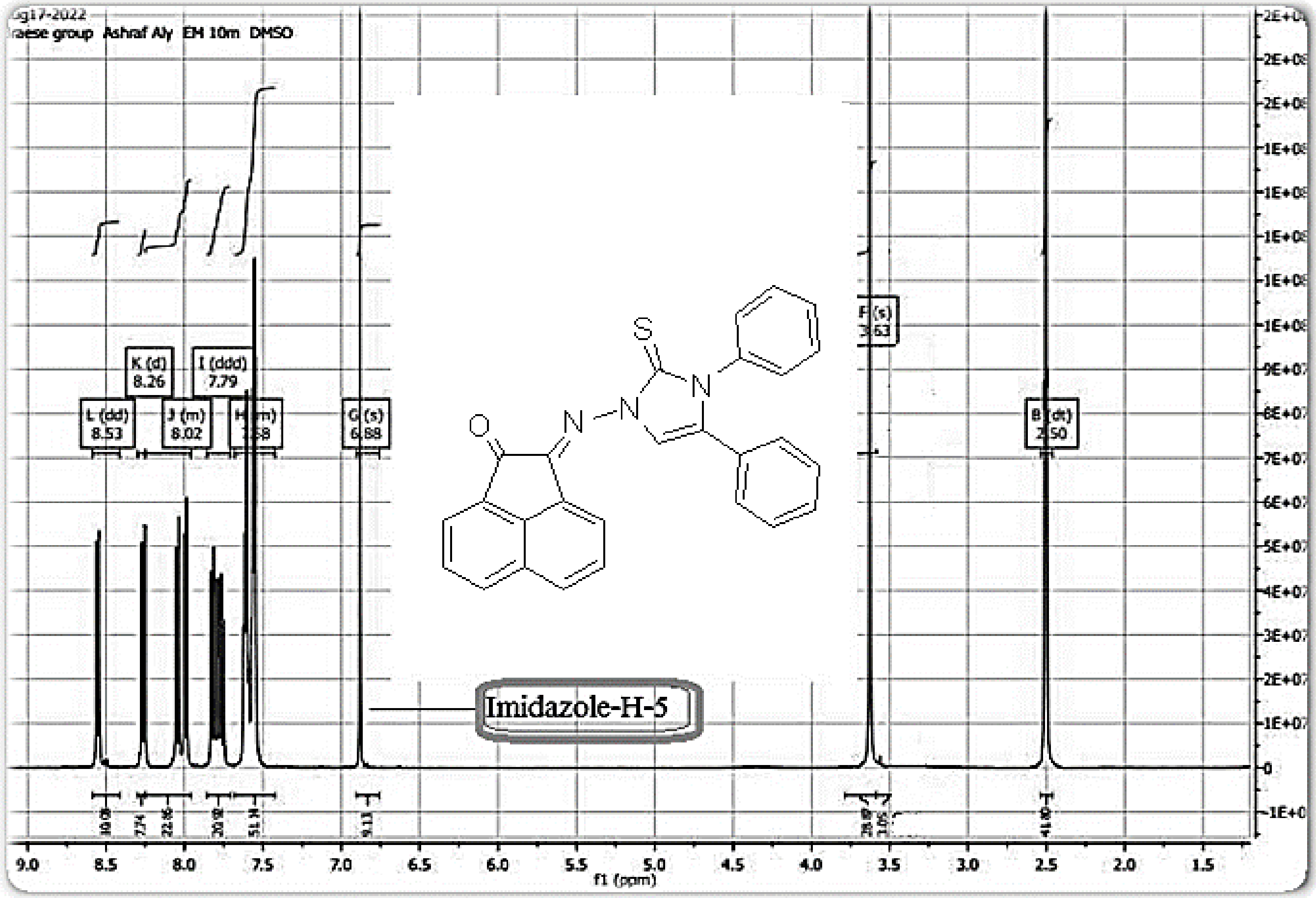

SI Figure 20.^{1}H NMR spectrum of **5b**

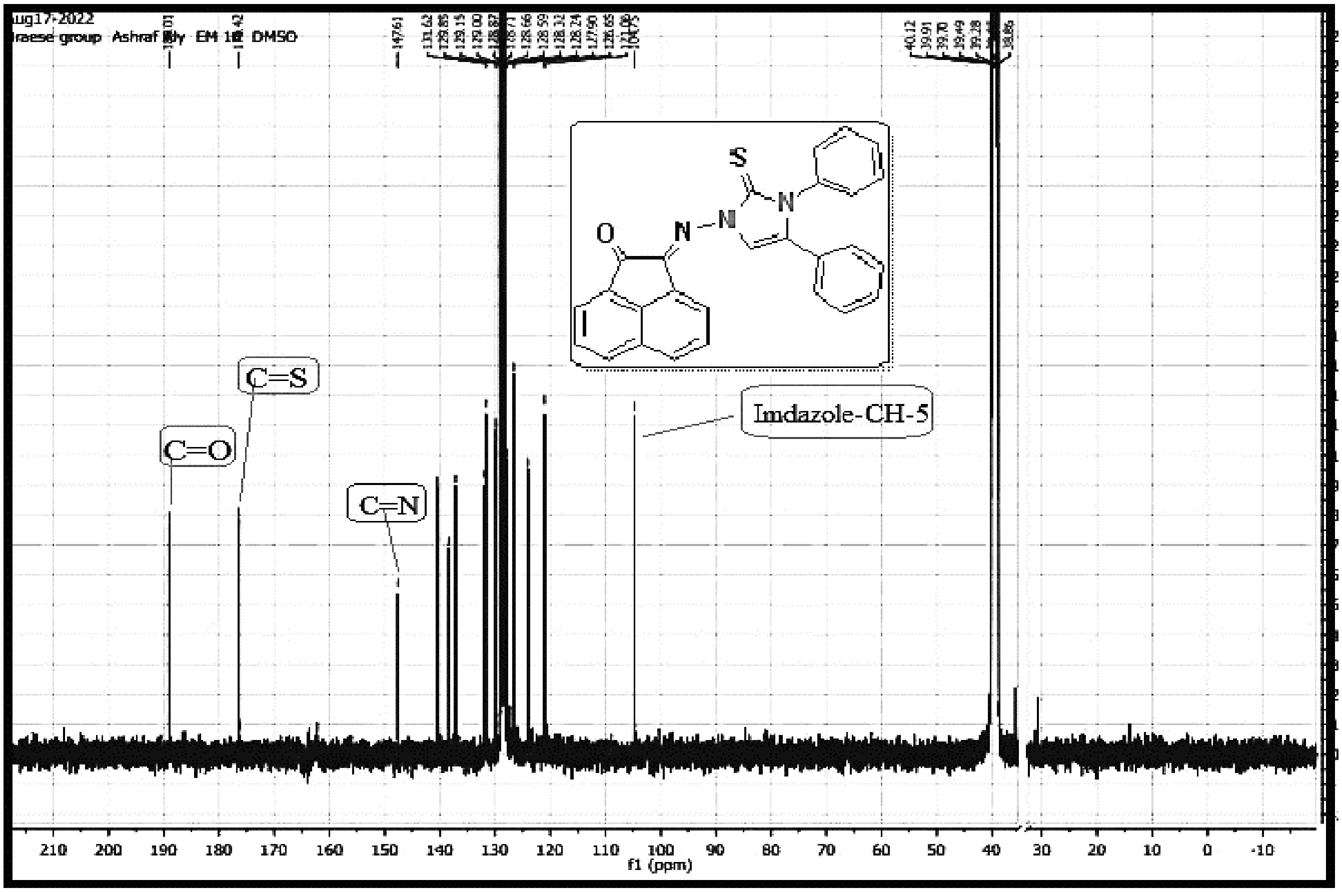

SI Figure 21.^{13}C NMR spectrum of **5b**

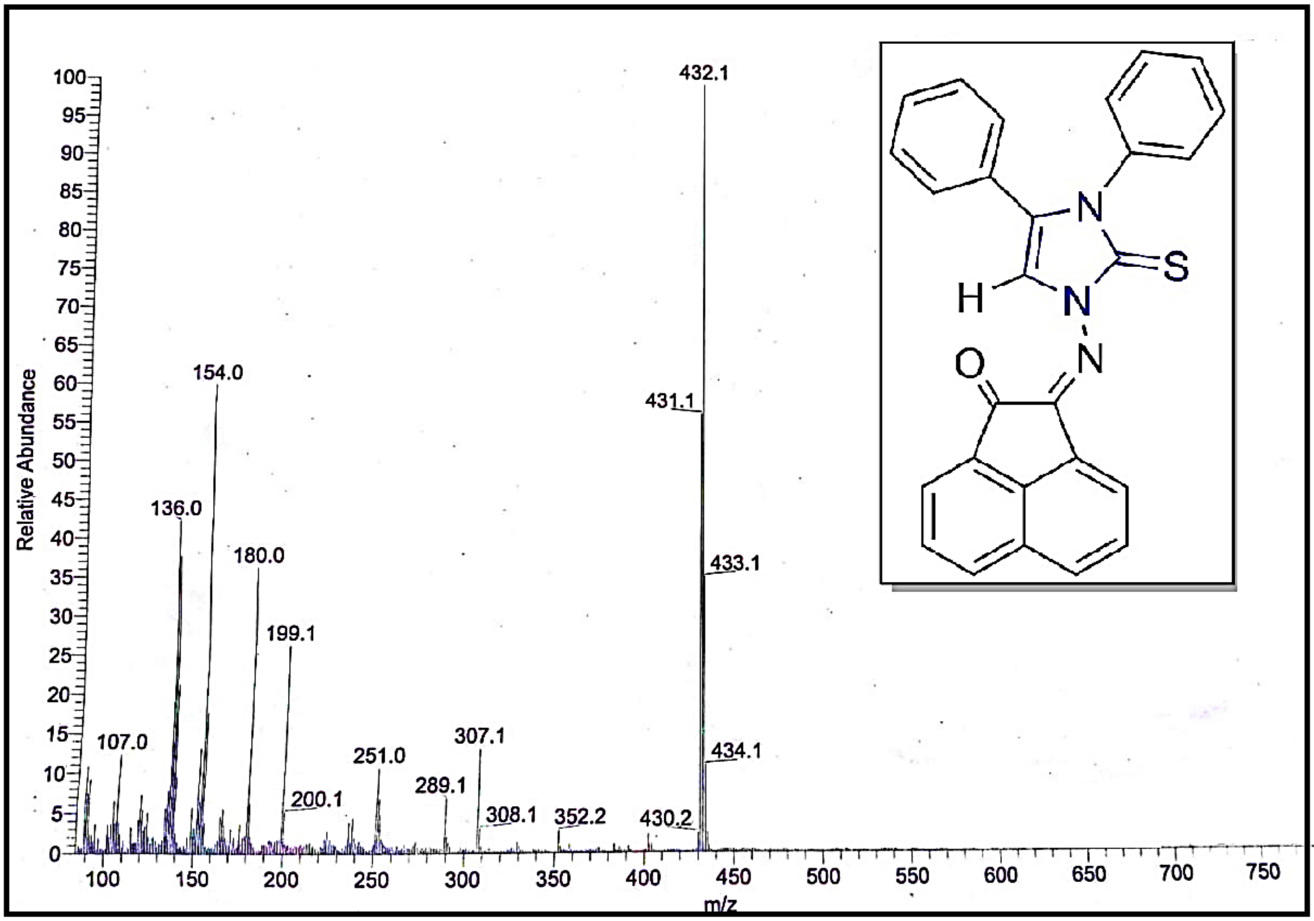

SI Figure 22. Mass spectroscopy of **5b**

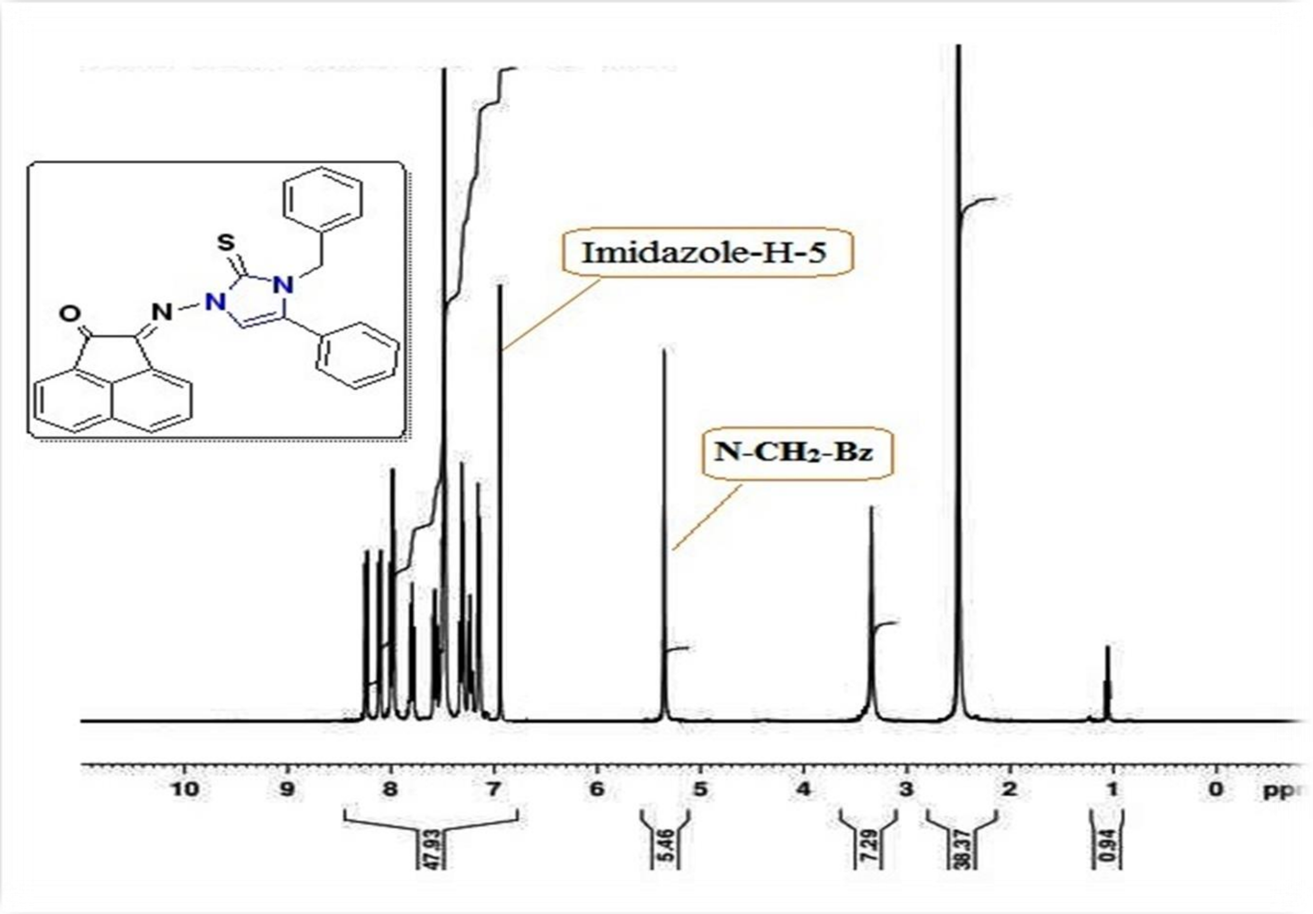

SI Figure 23. ^{1}H NMR spectrum of **5c**

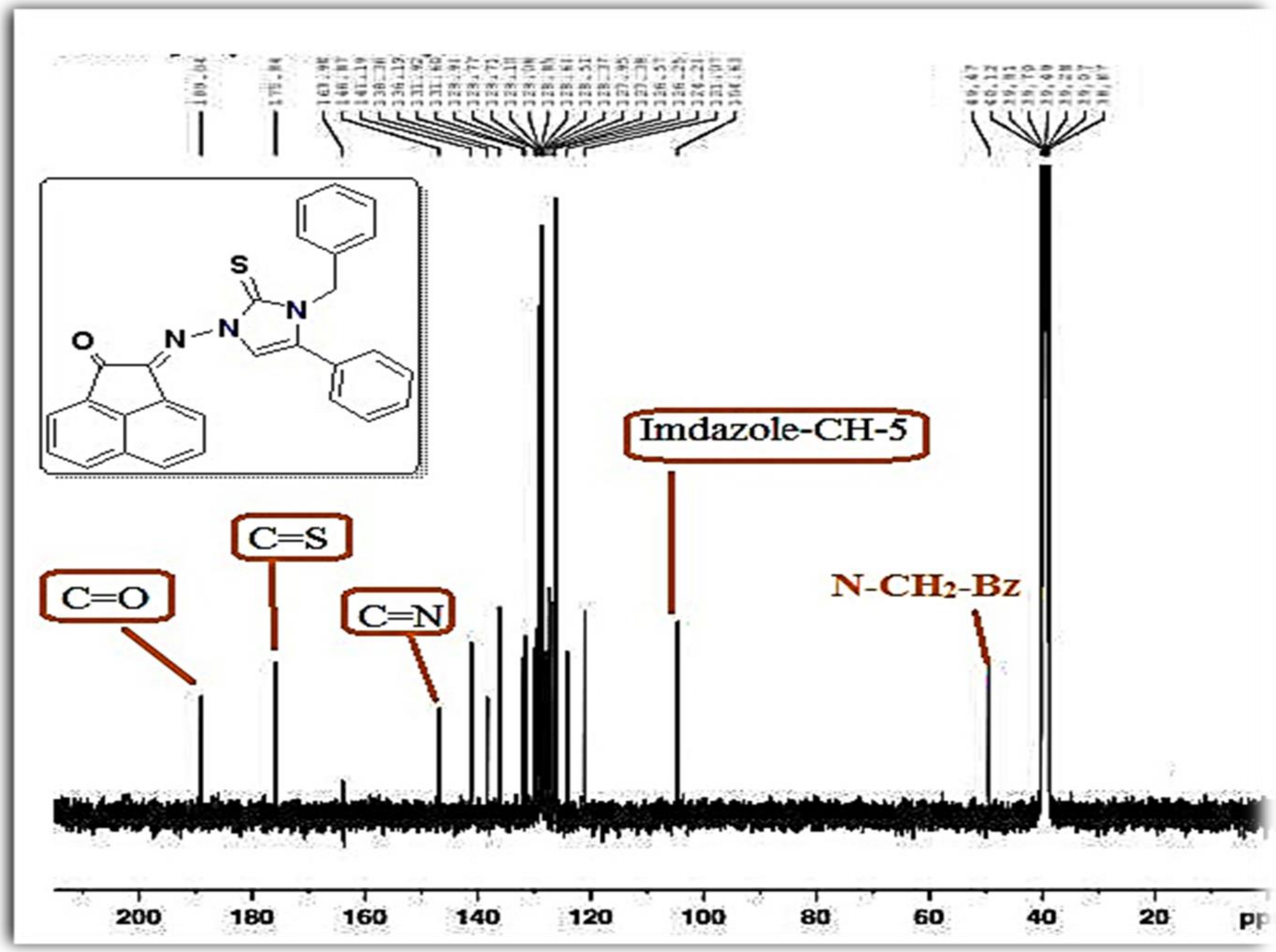

SI Figure 24. ^{13}C NMR spectrum of **5c**

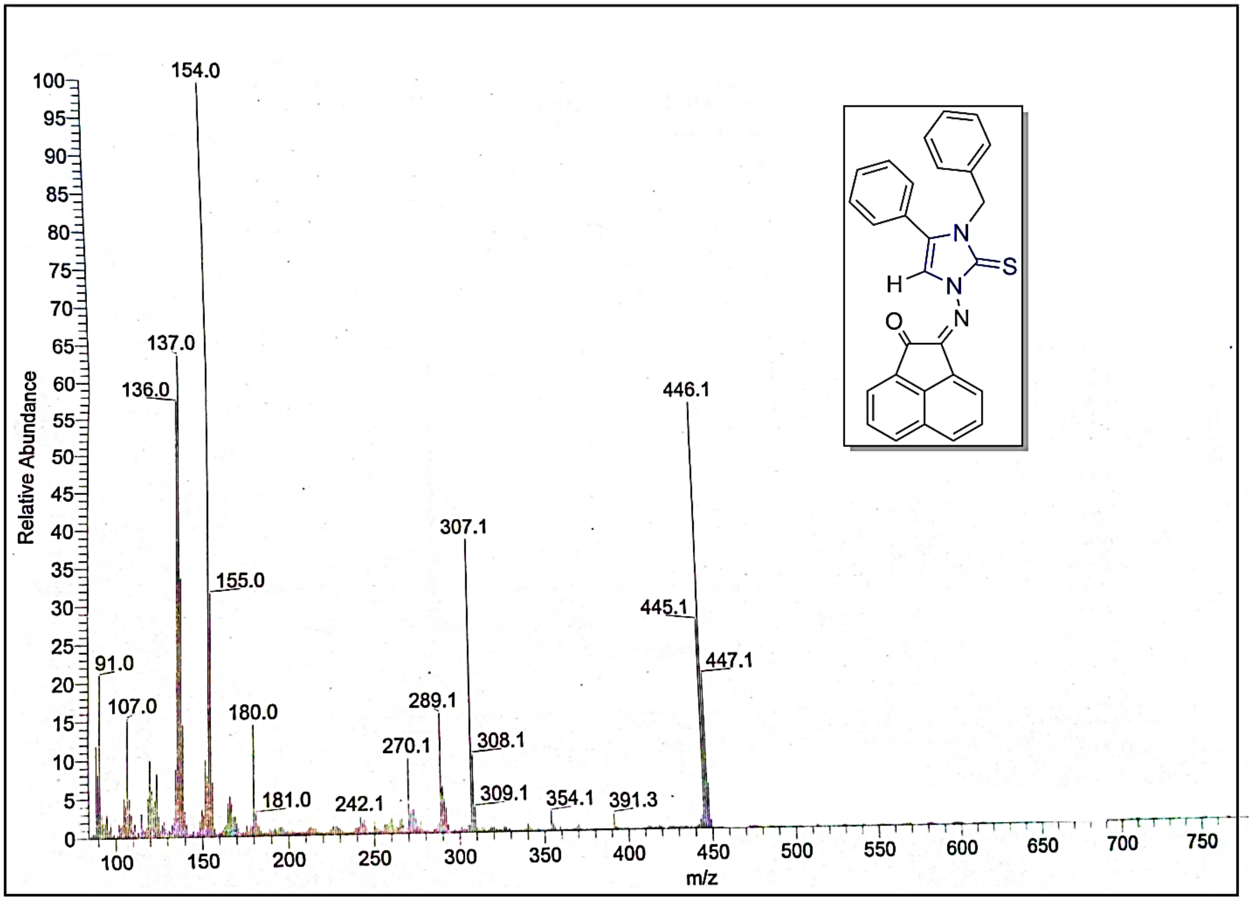

SI Figure 25. Mass spectroscopy of **5c**

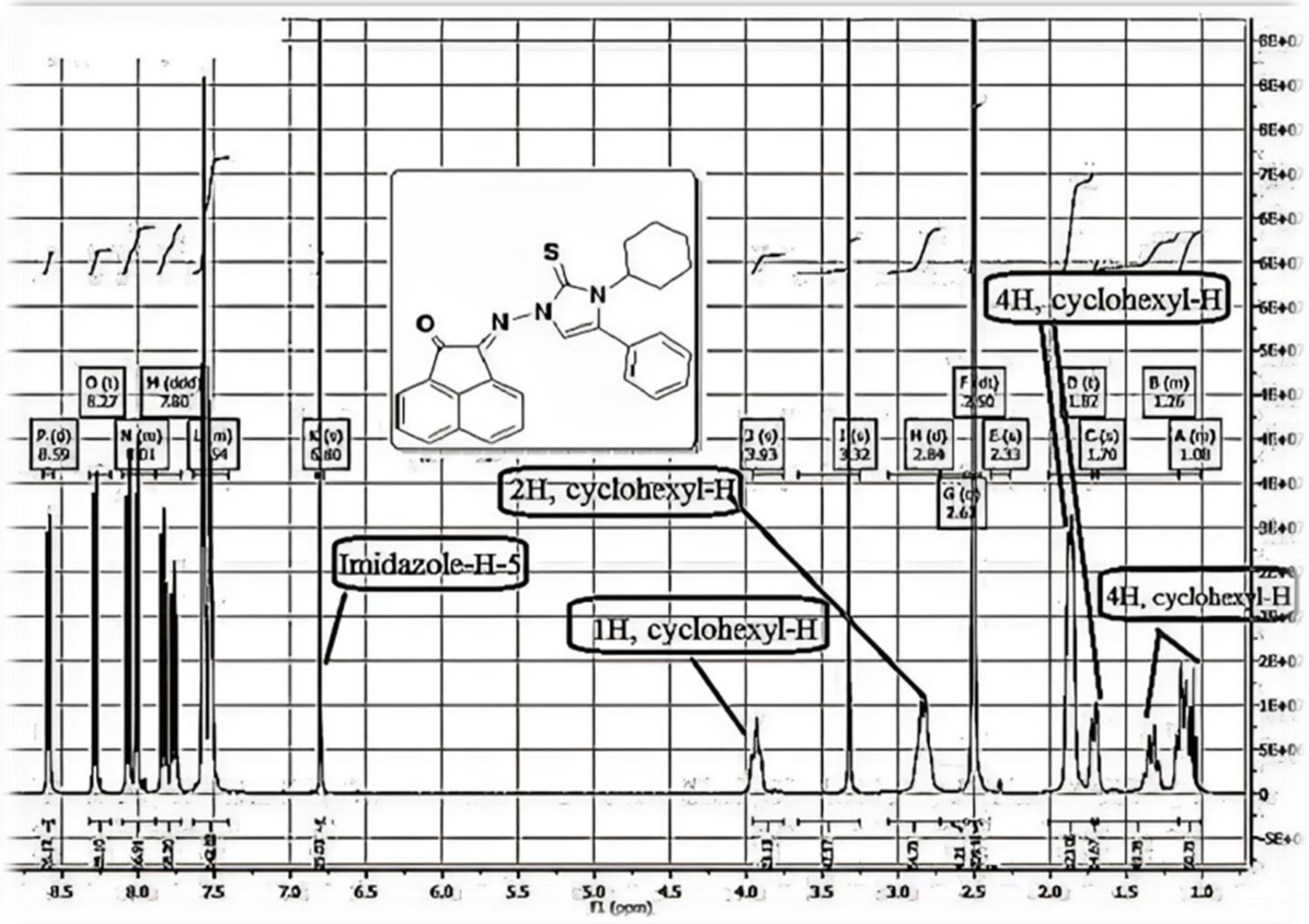

SI Figure 26. ^{1}H NMR spectrum of **5d**

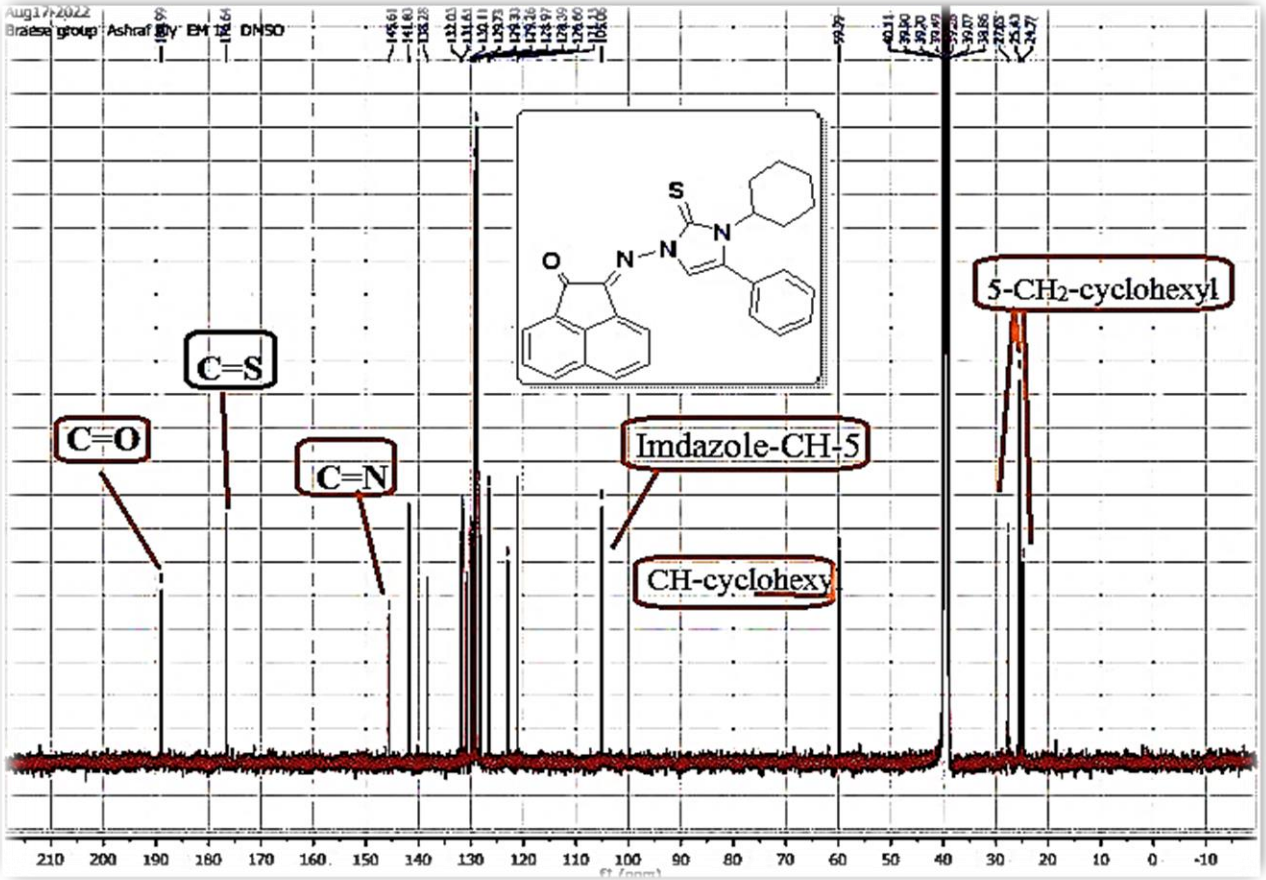

SI Figure 27. ^{13}C NMR spectrum of **5d**

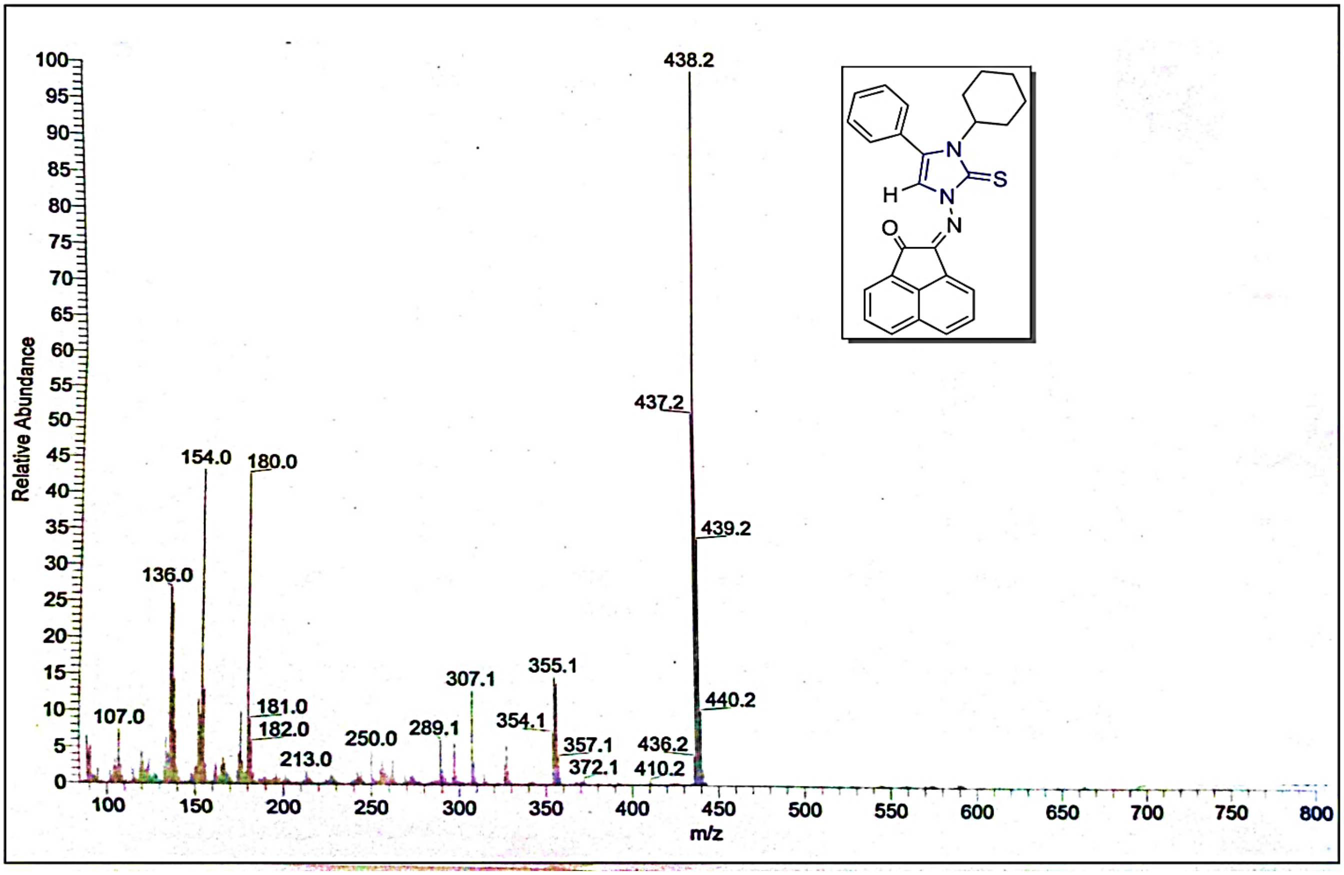

SI Figure 28. Mass spectroscopy of **5d**

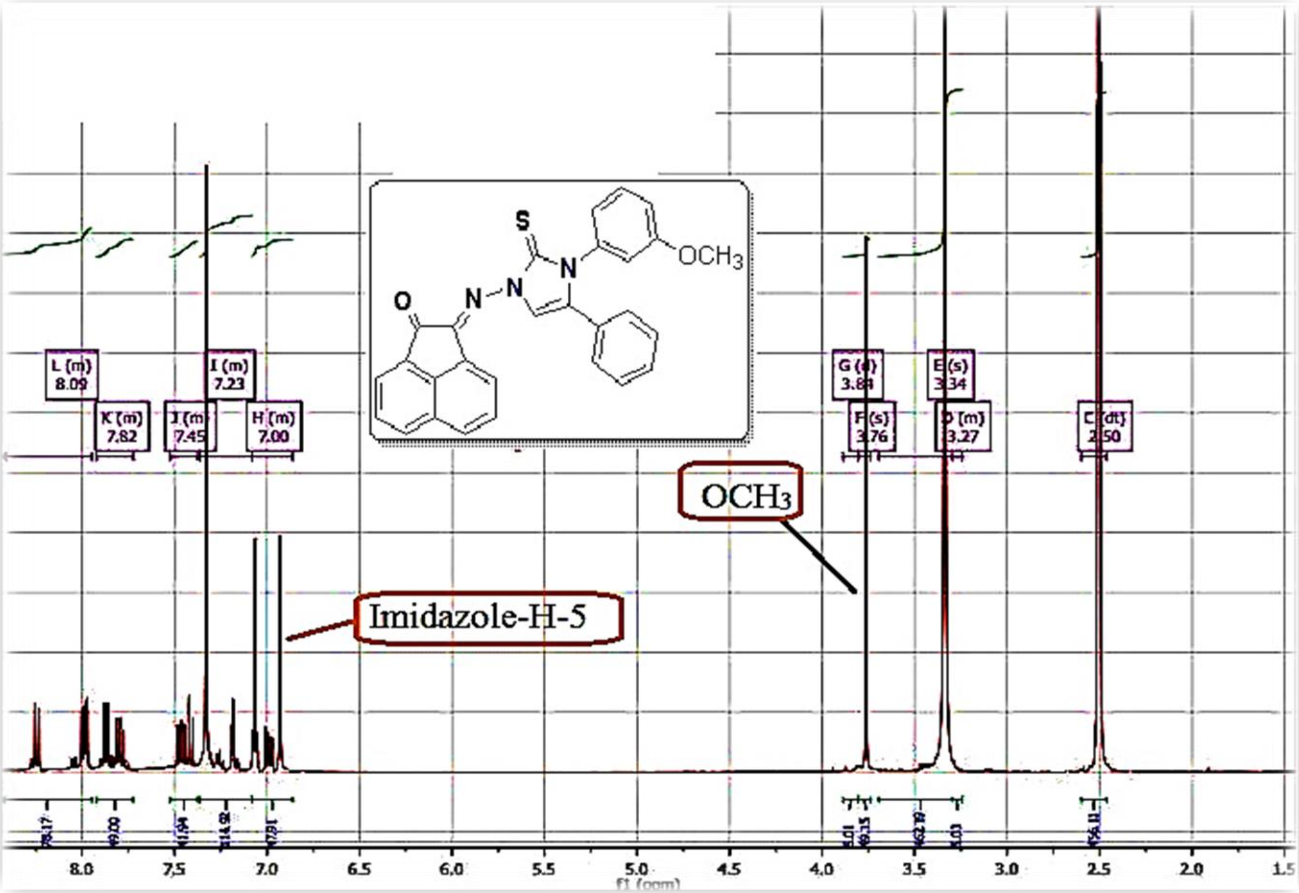

SI Figure 29. ^{1}H NMR spectrum of **5e**

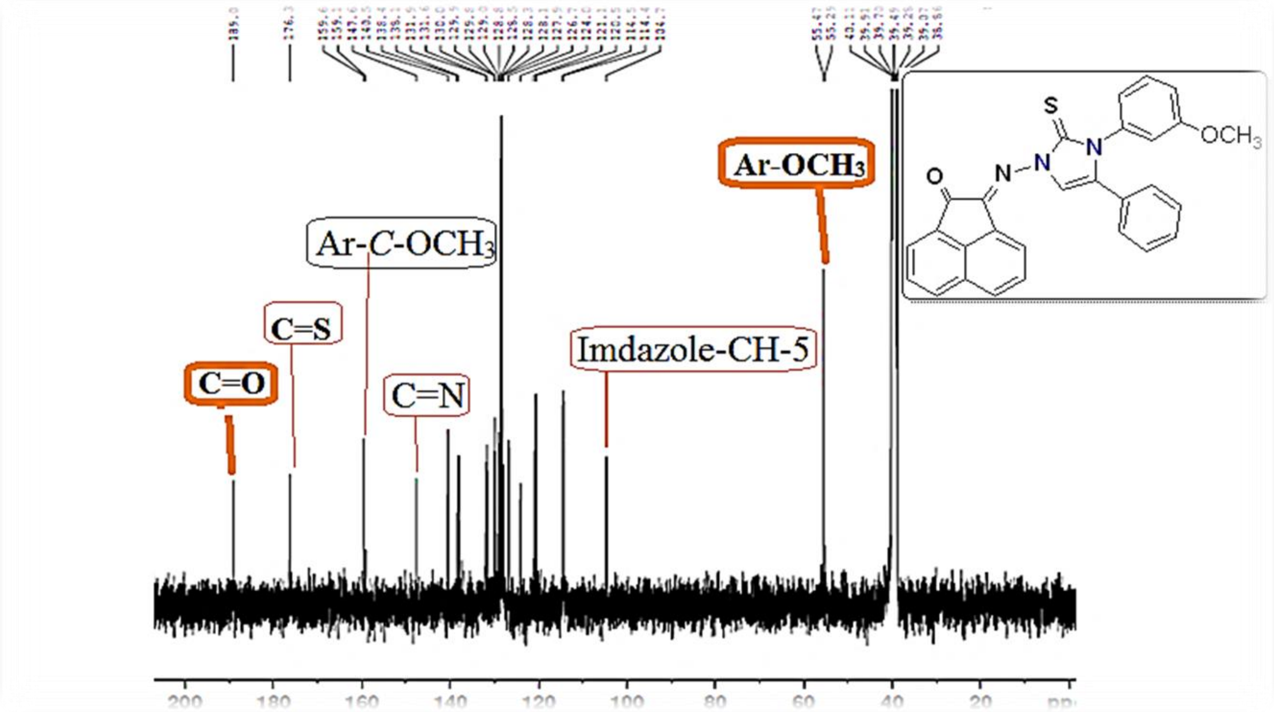

SI Figure 30. ^{13}C NMR spectrum of **5e**

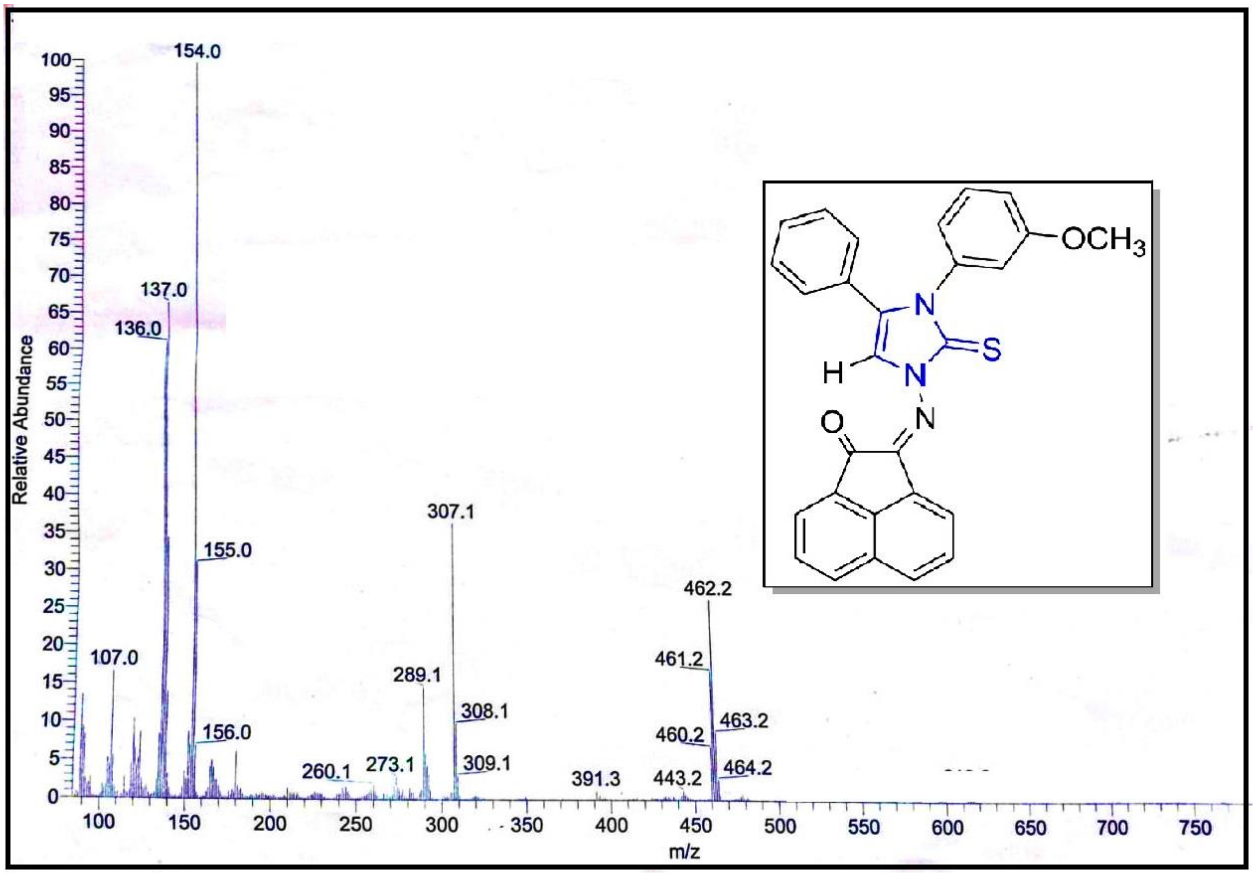

SI Figure 31. Mass spectroscopy of **5e**

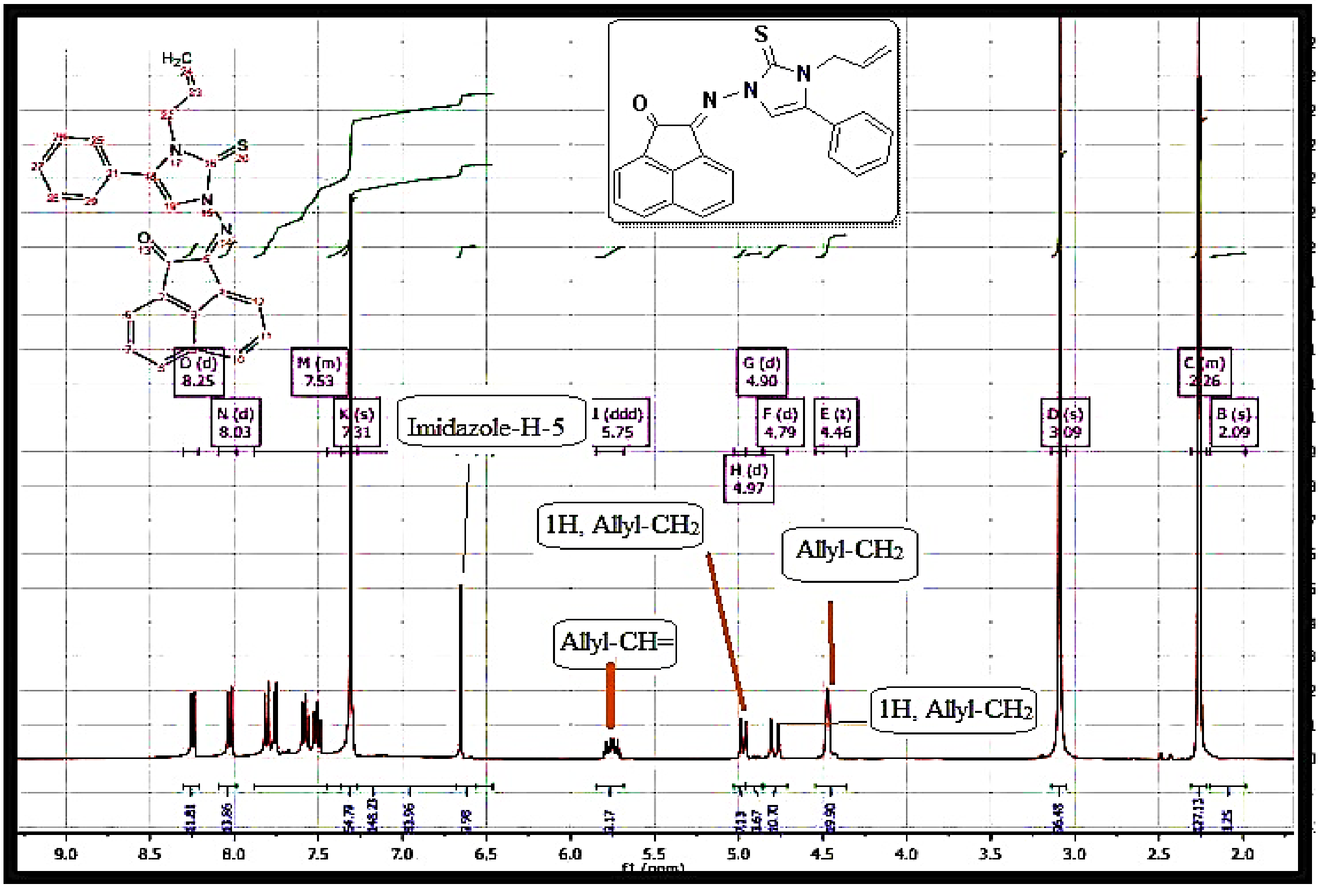

SI Figure 32. ^{1}H NMR spectrum of **5f**

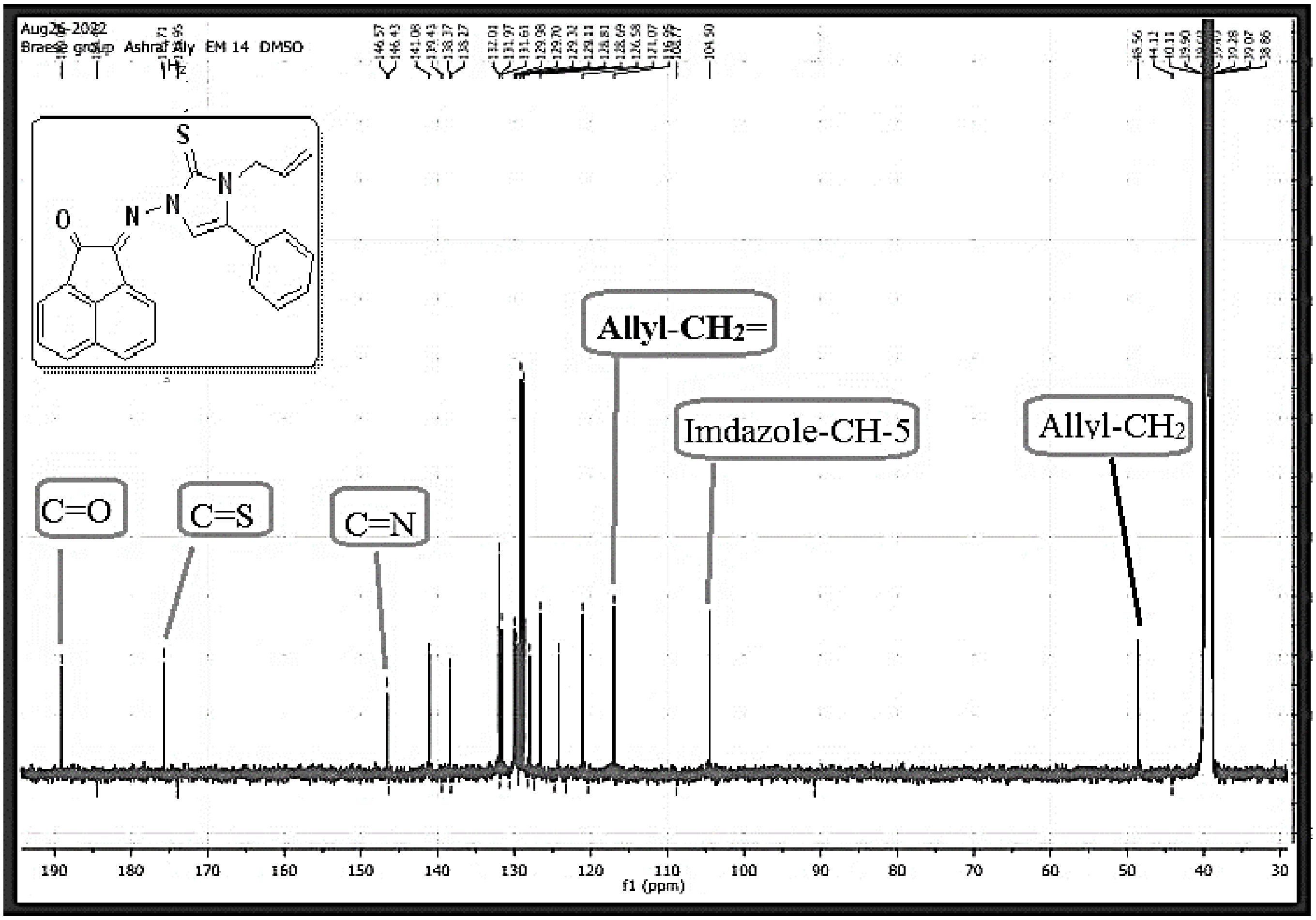

SI Figure 33. ^{13}C NMR spectrum of **5f**

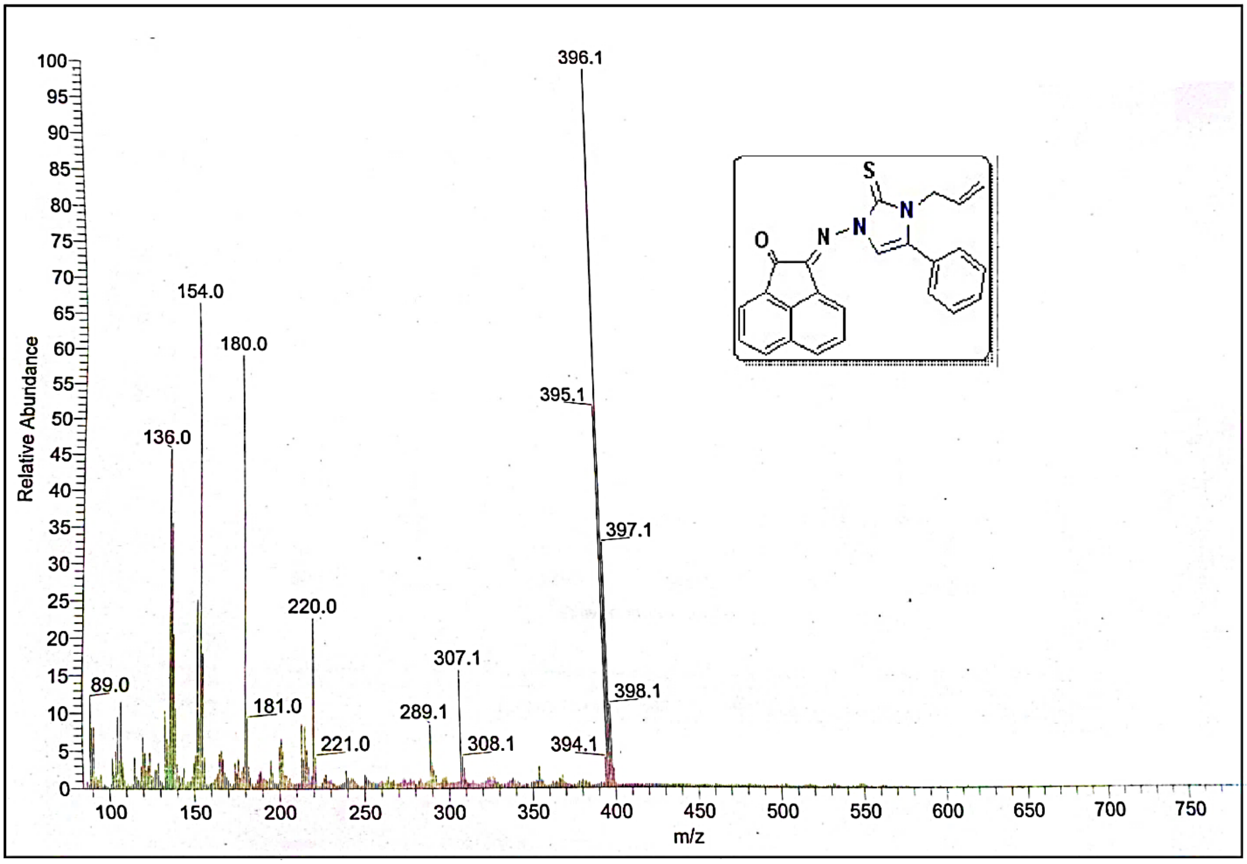

SI Figure 34. Mass spectroscopy of **5f**

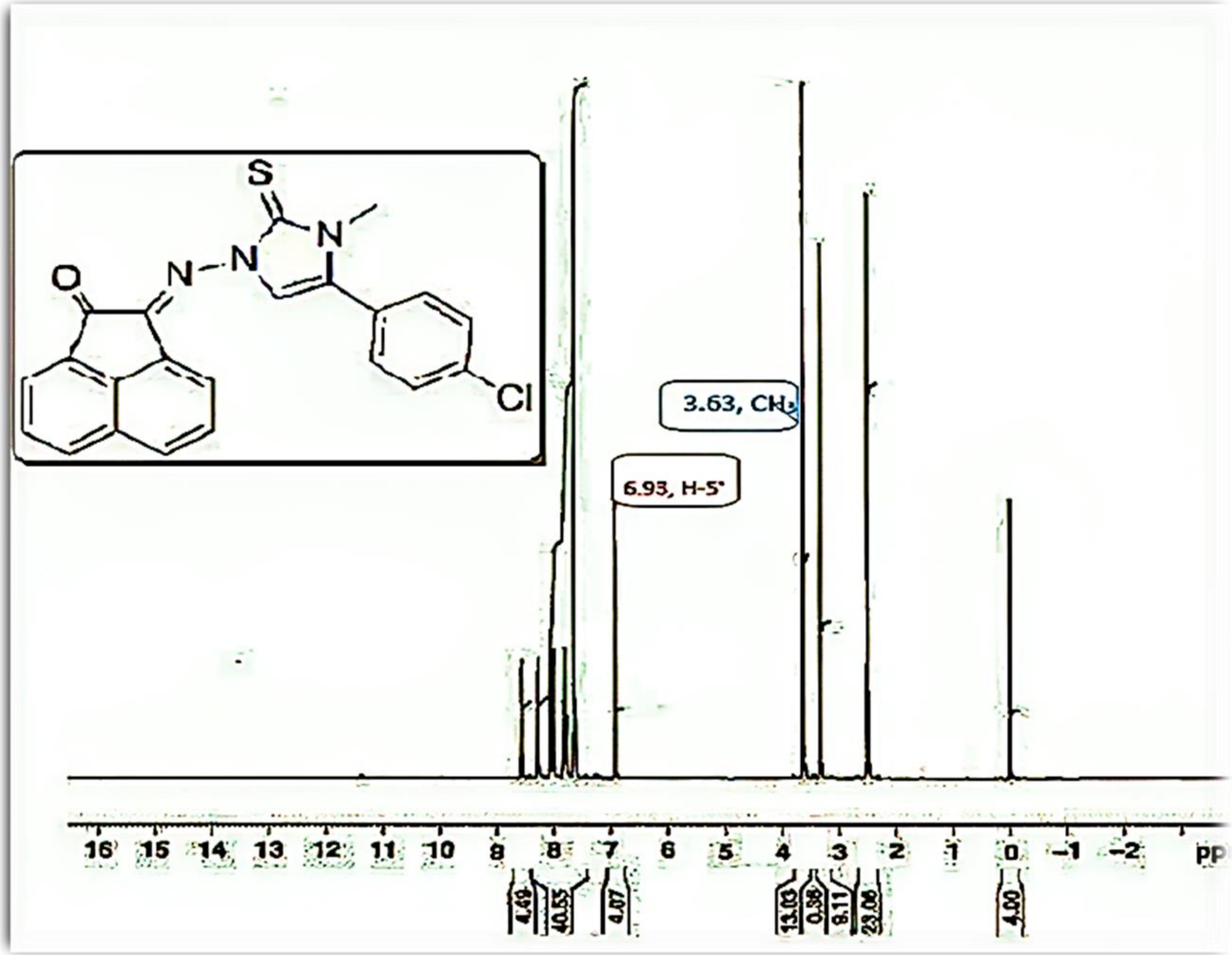

SI Figure 35. ^{1}H NMR spectrum of **5g**

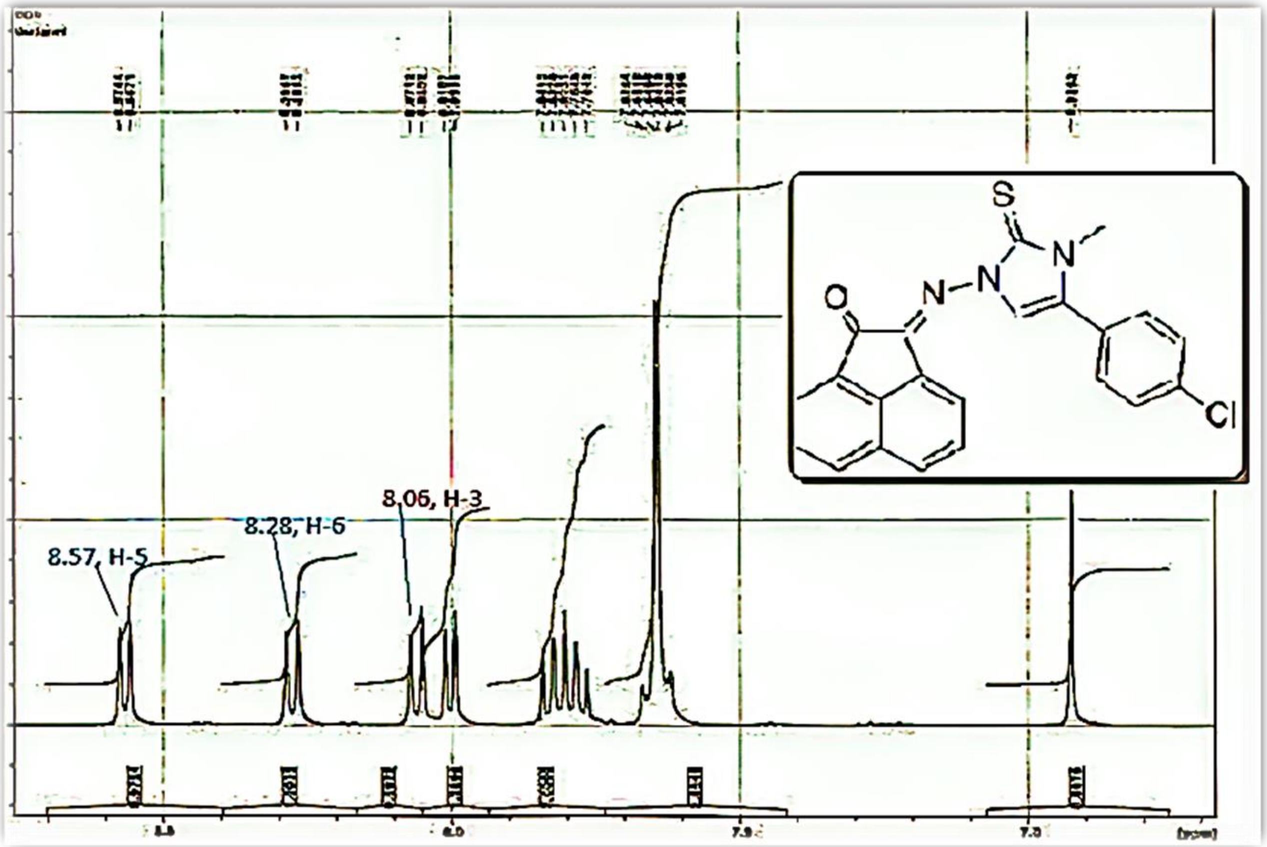

SI Figure 36. Expanded ^1H NMR spectrum of **5g**

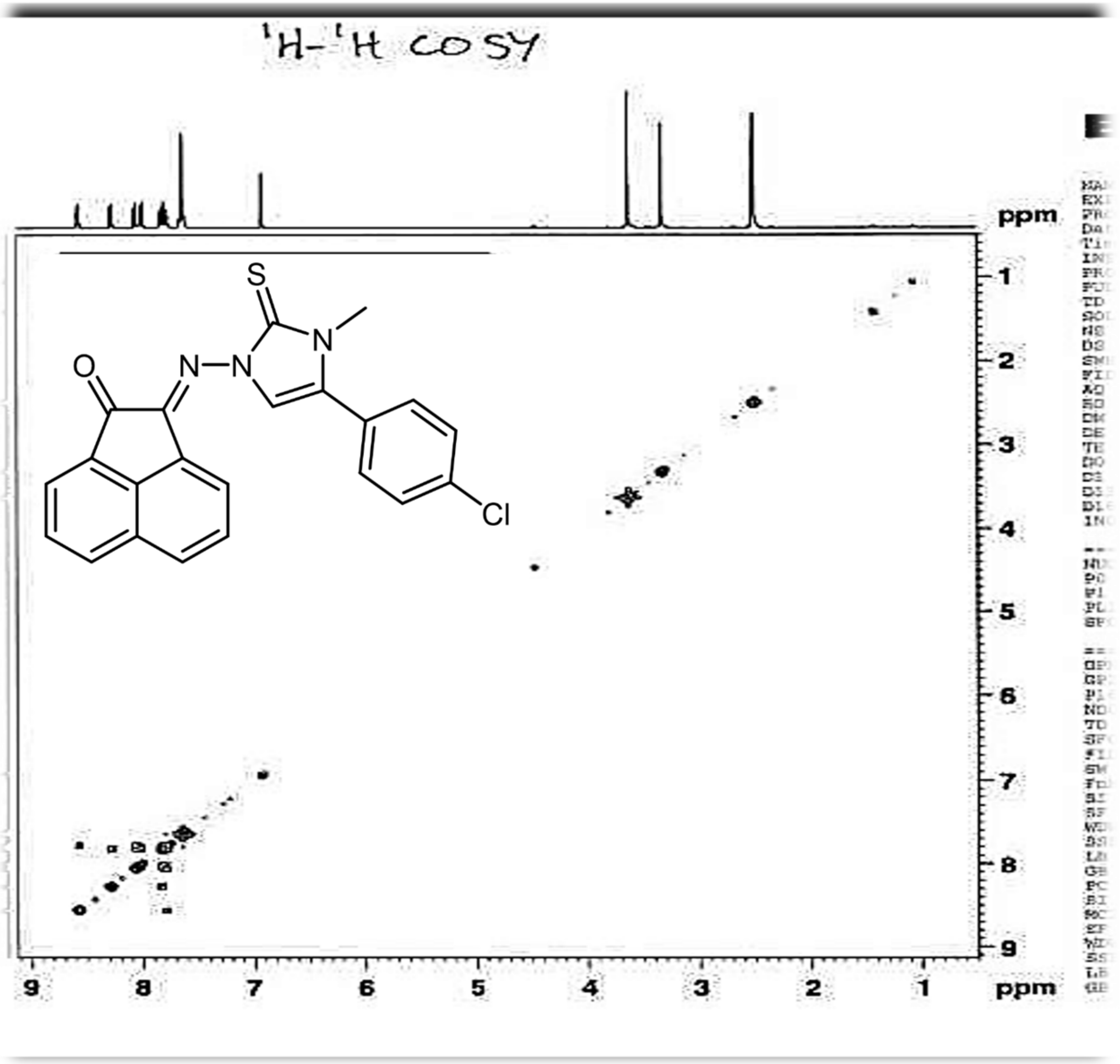

SI Figure 37. COSY H-H spectrum of **5g**

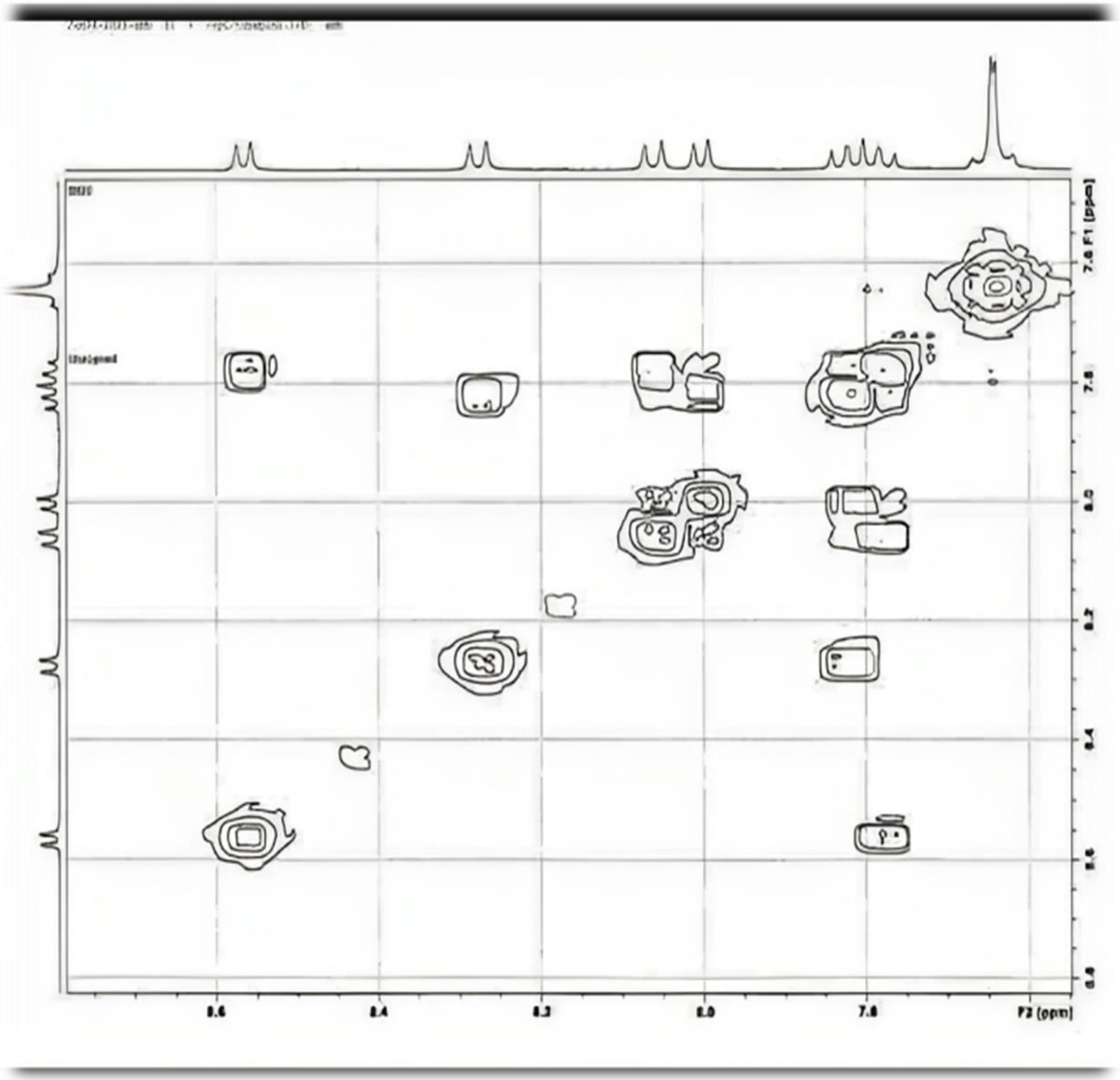

SI Figure 38. Expanded COSY H-H spectrum of **5g**

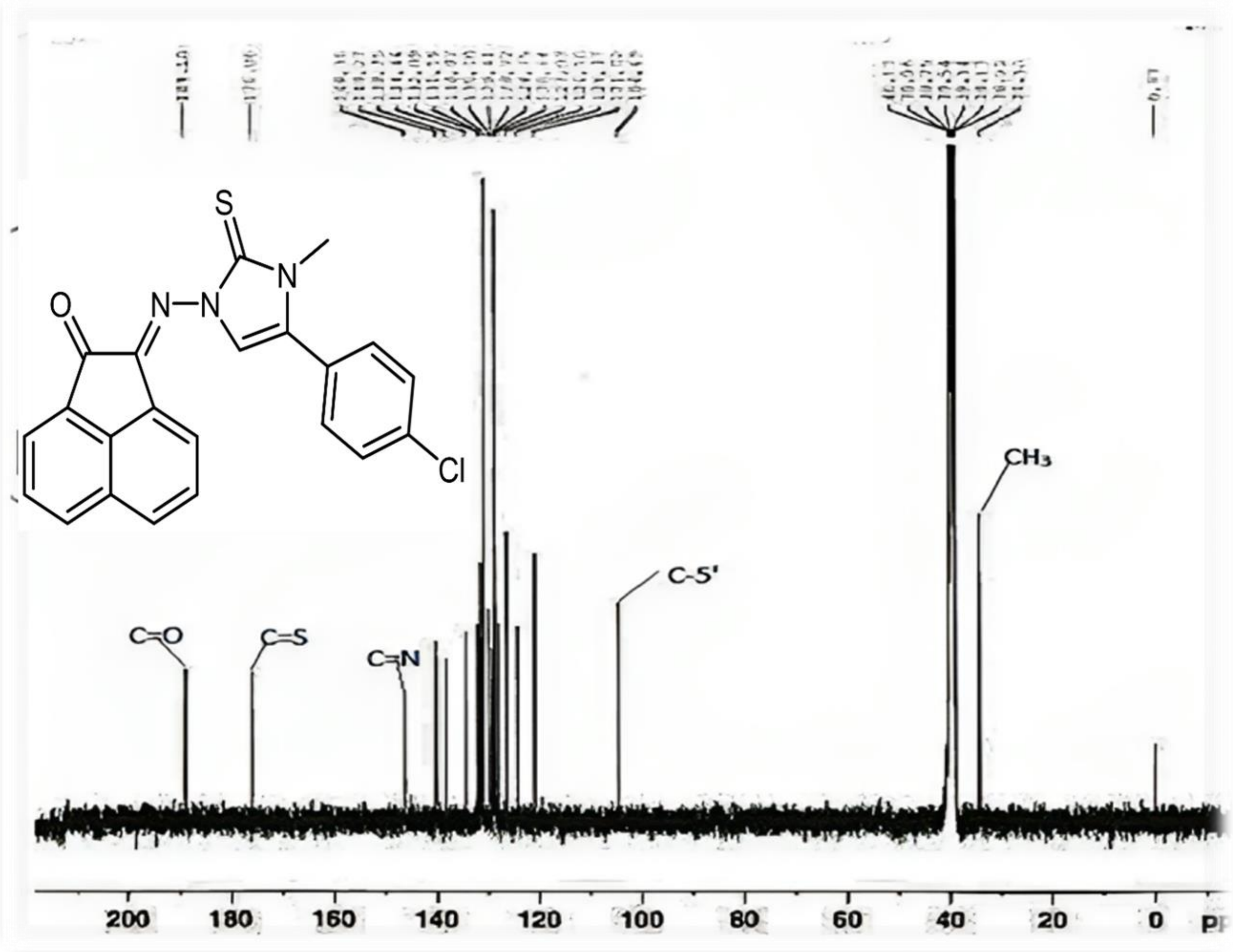

SI Figure 39. ^{13}C NMR spectrum of **5g**

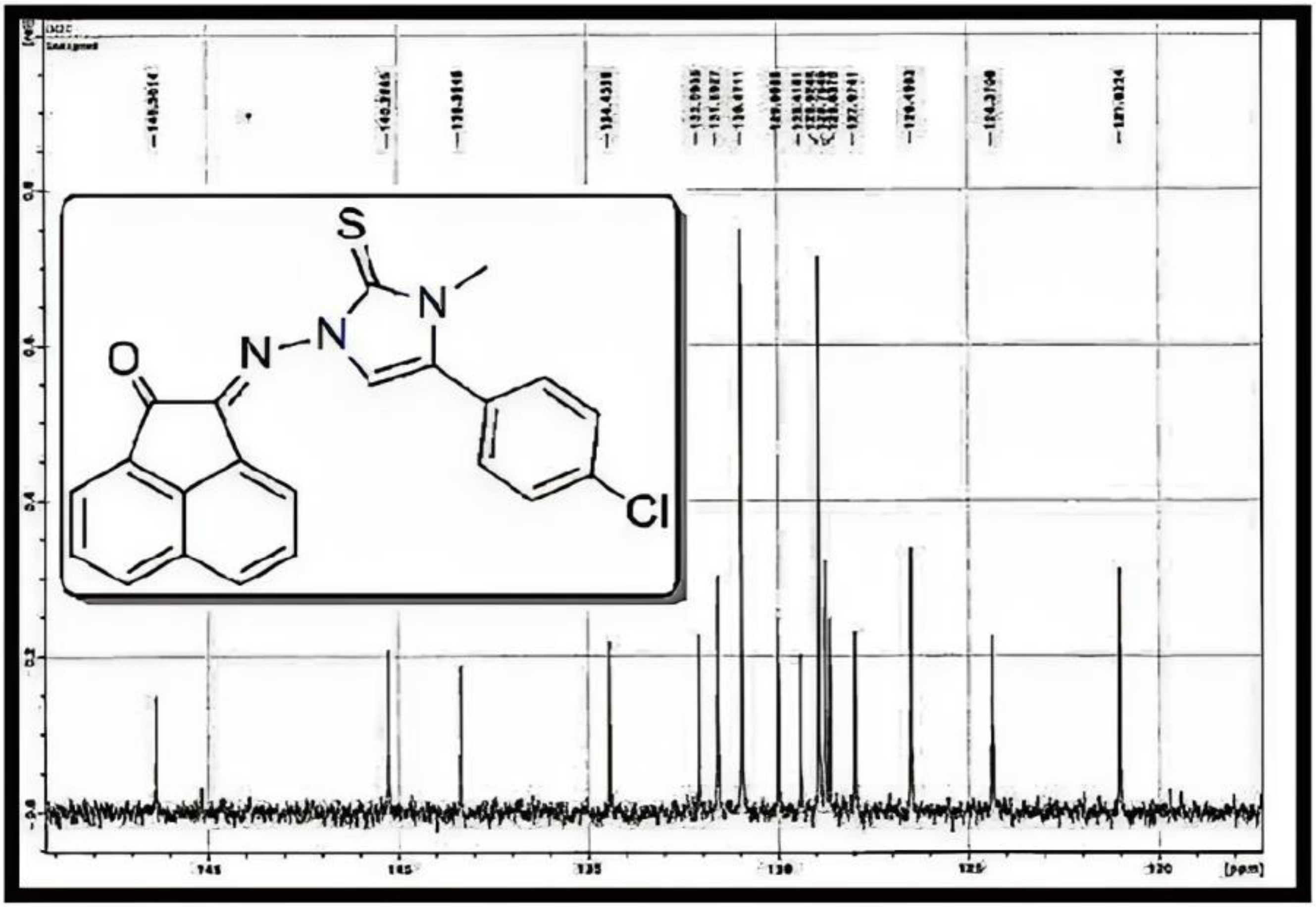

SI Figure 40. Expanded ^{13}C NMR spectrum of **5g**

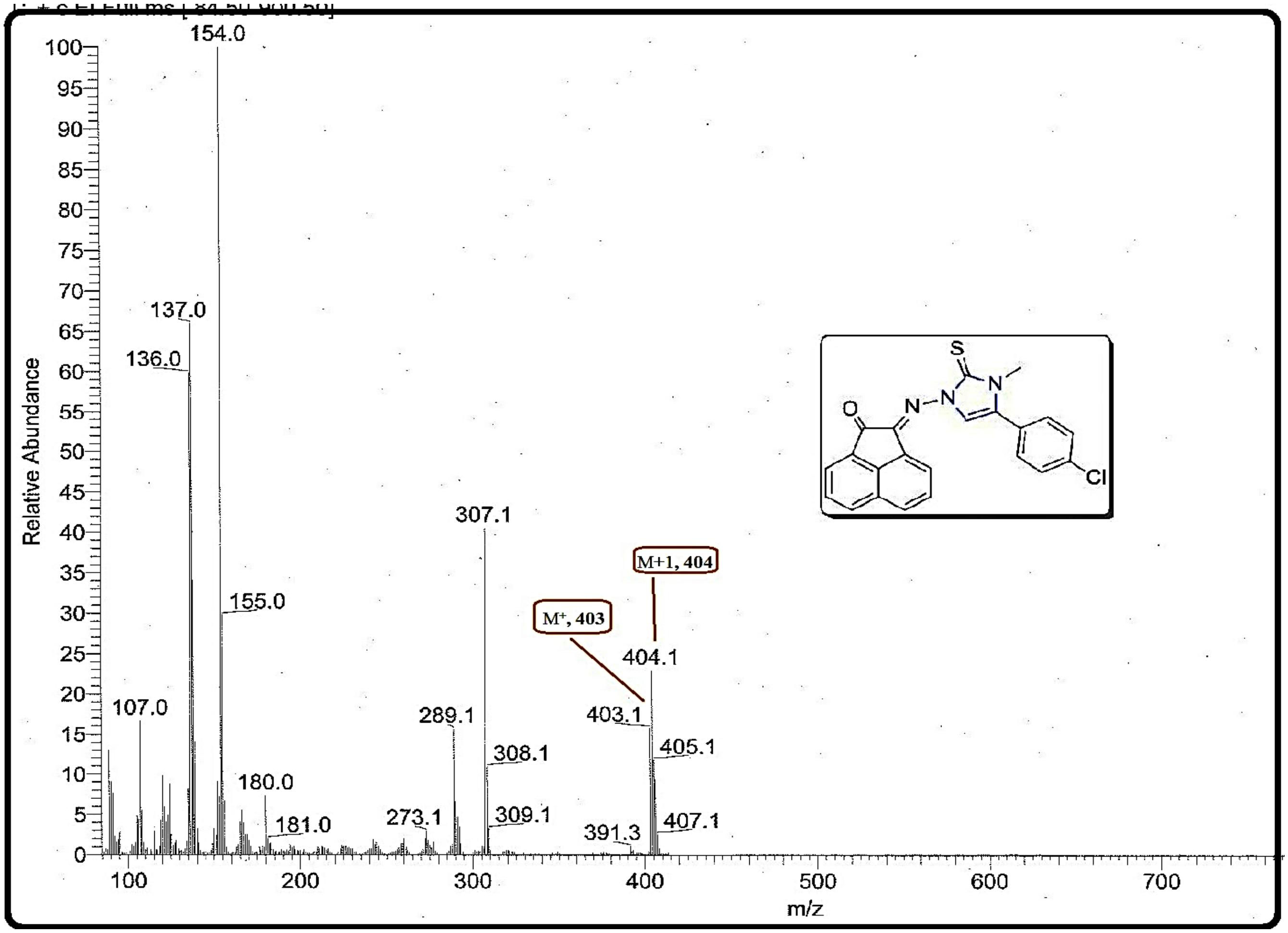

SI Figure 41. Mass spectroscopy of **5g**

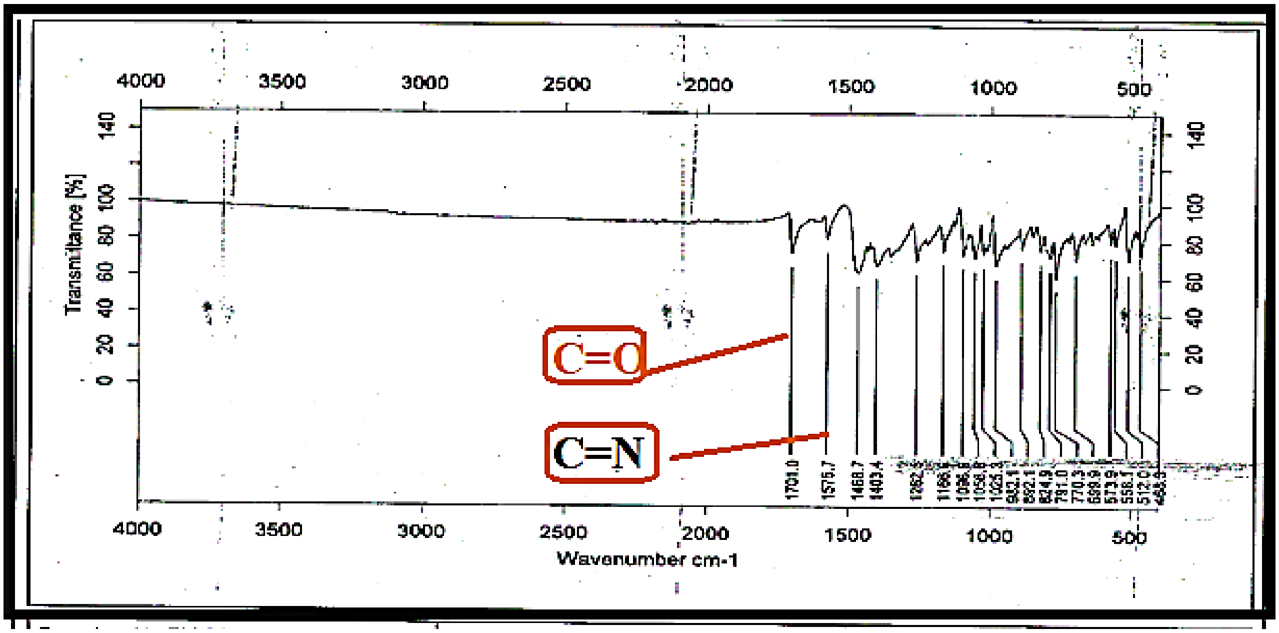

SI Figure 42. IR spectrum of compound **5g**

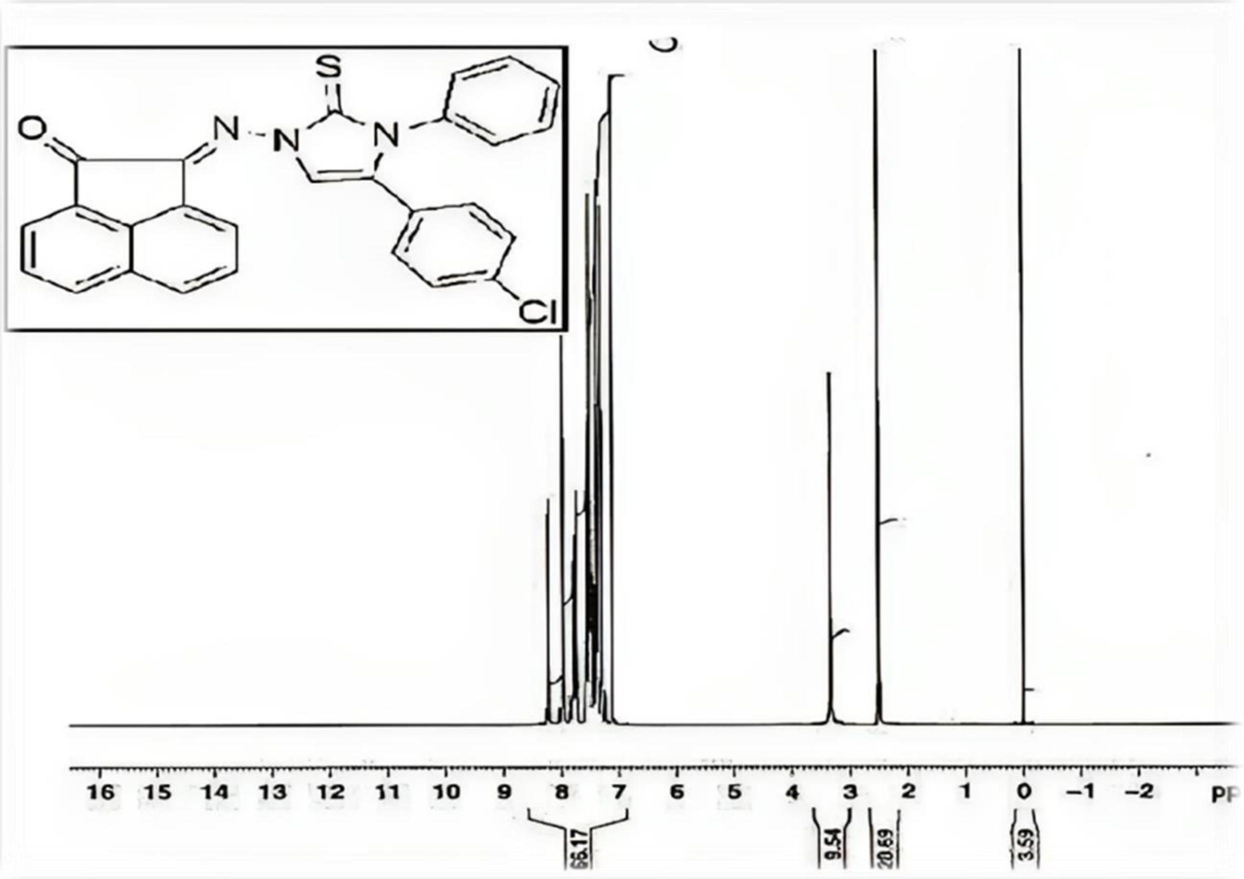

SI Figure 43. ^{1}H NMR spectrum of **5h**

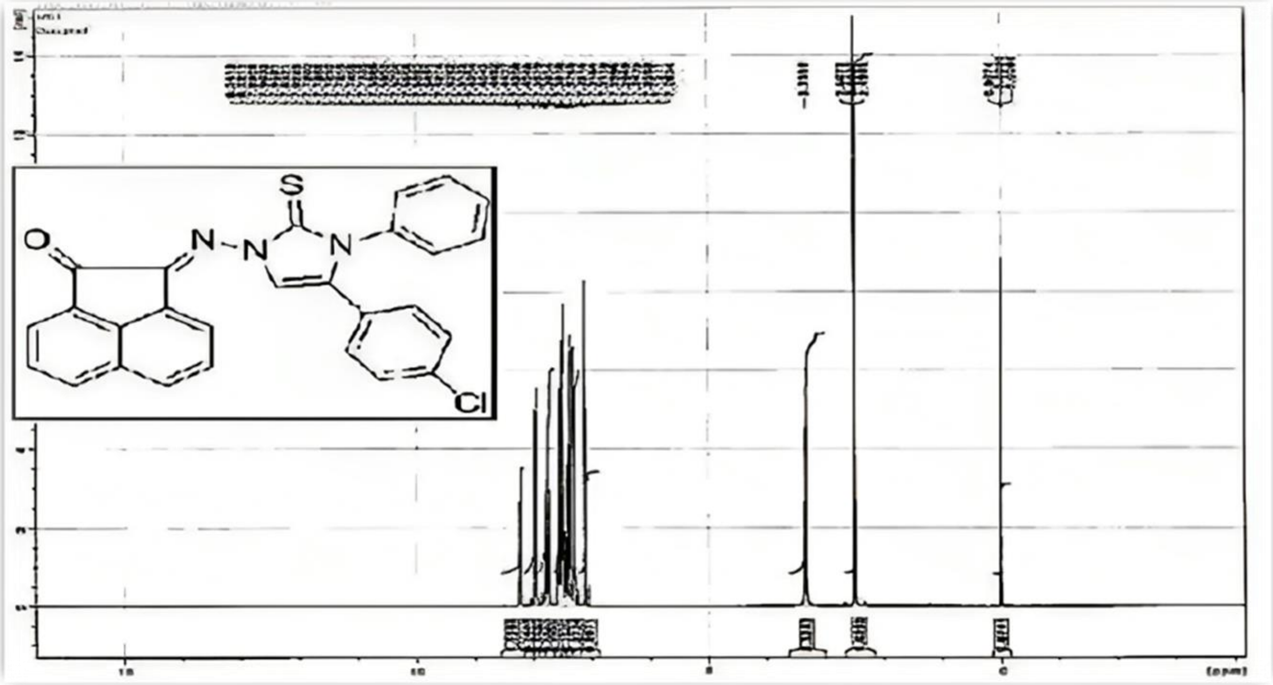

SI Figure 44. Expanded ^{1}H NMR spectrum of **5h**

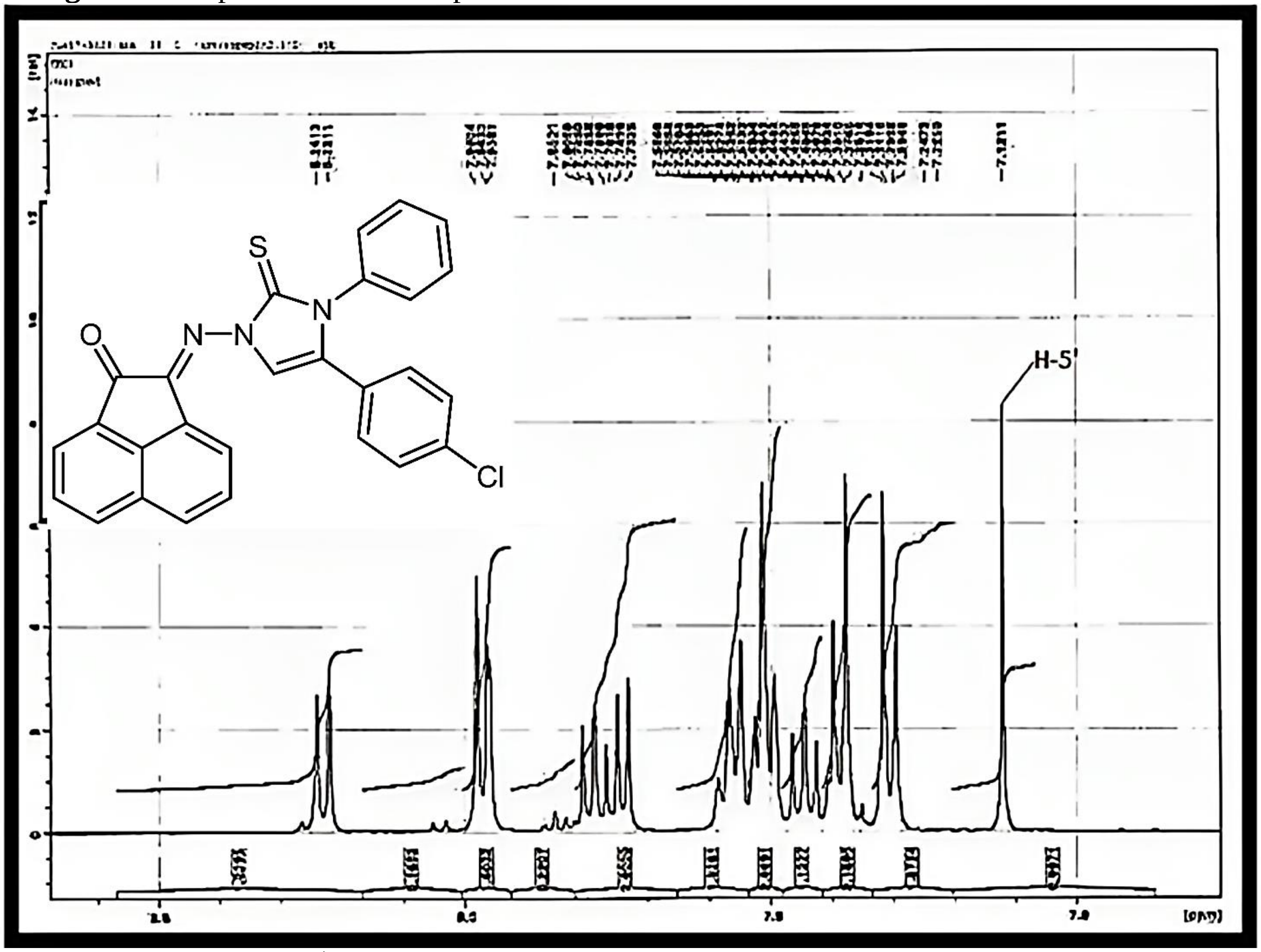

SI Figure 45. Expanded ^{1}H NMR spectrum of **5h**

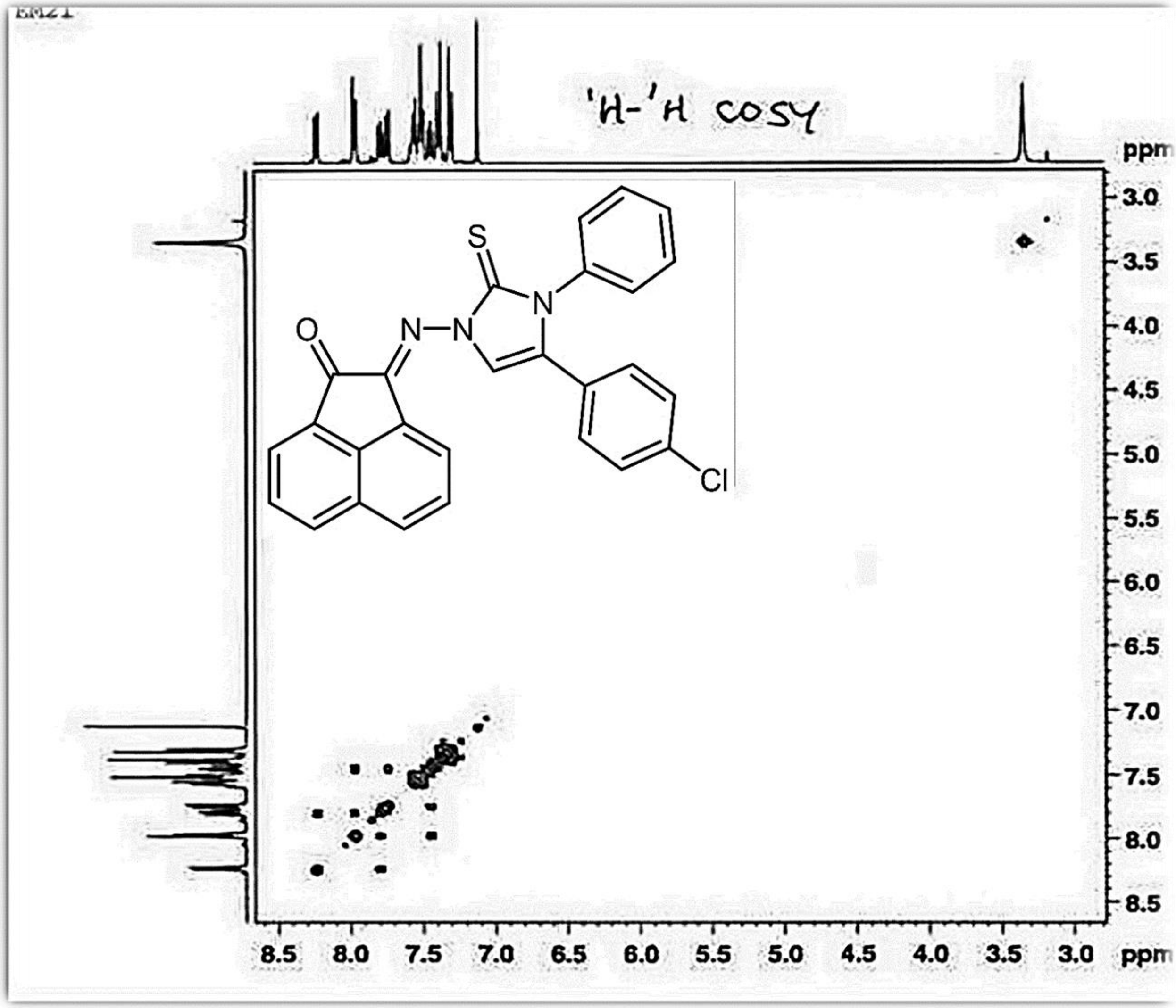

SI Figure 46. COSY H-H spectrum of **5h**

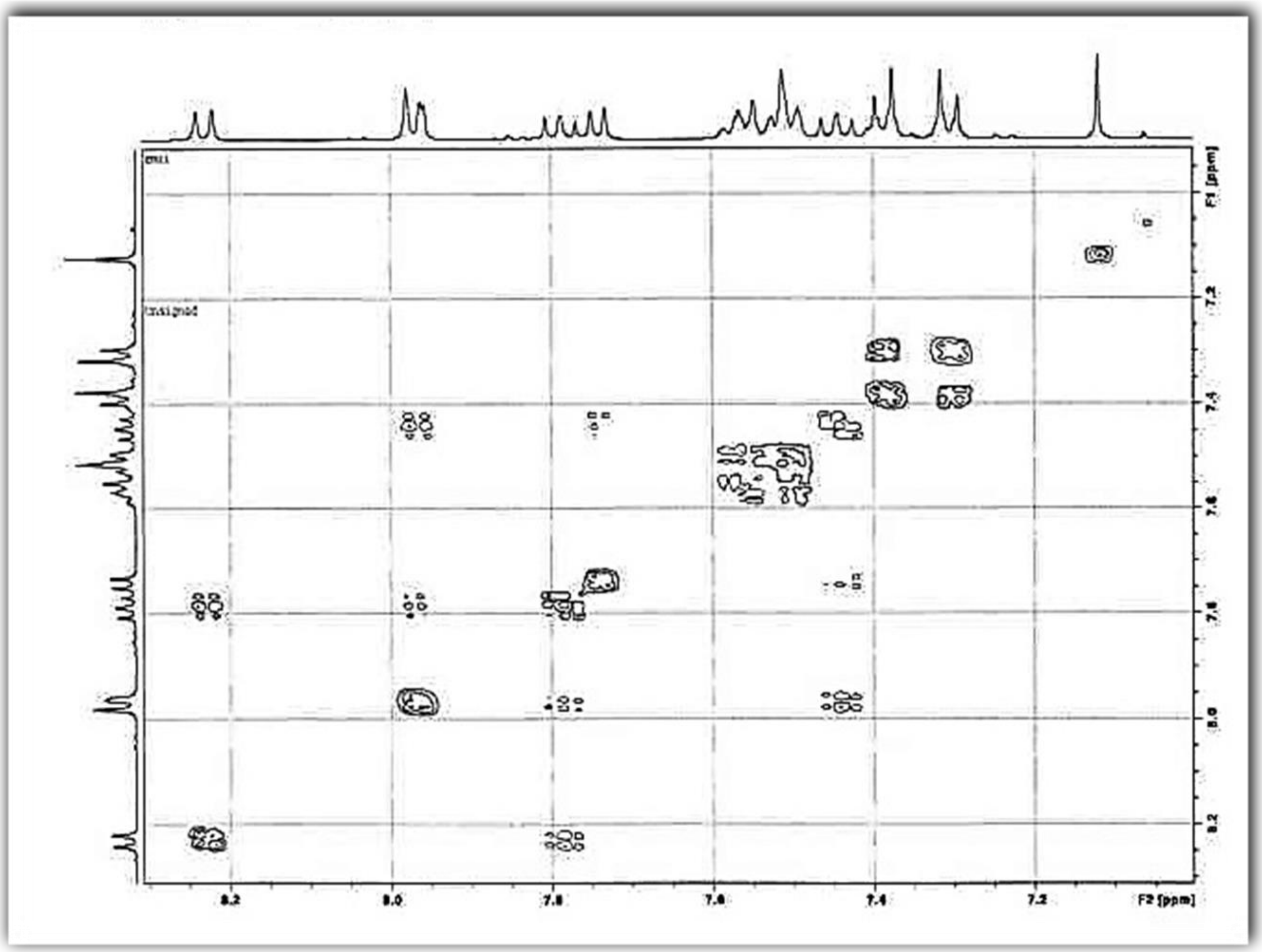

SI Figure 47. Expanded COSY H-H spectrum of **5h**

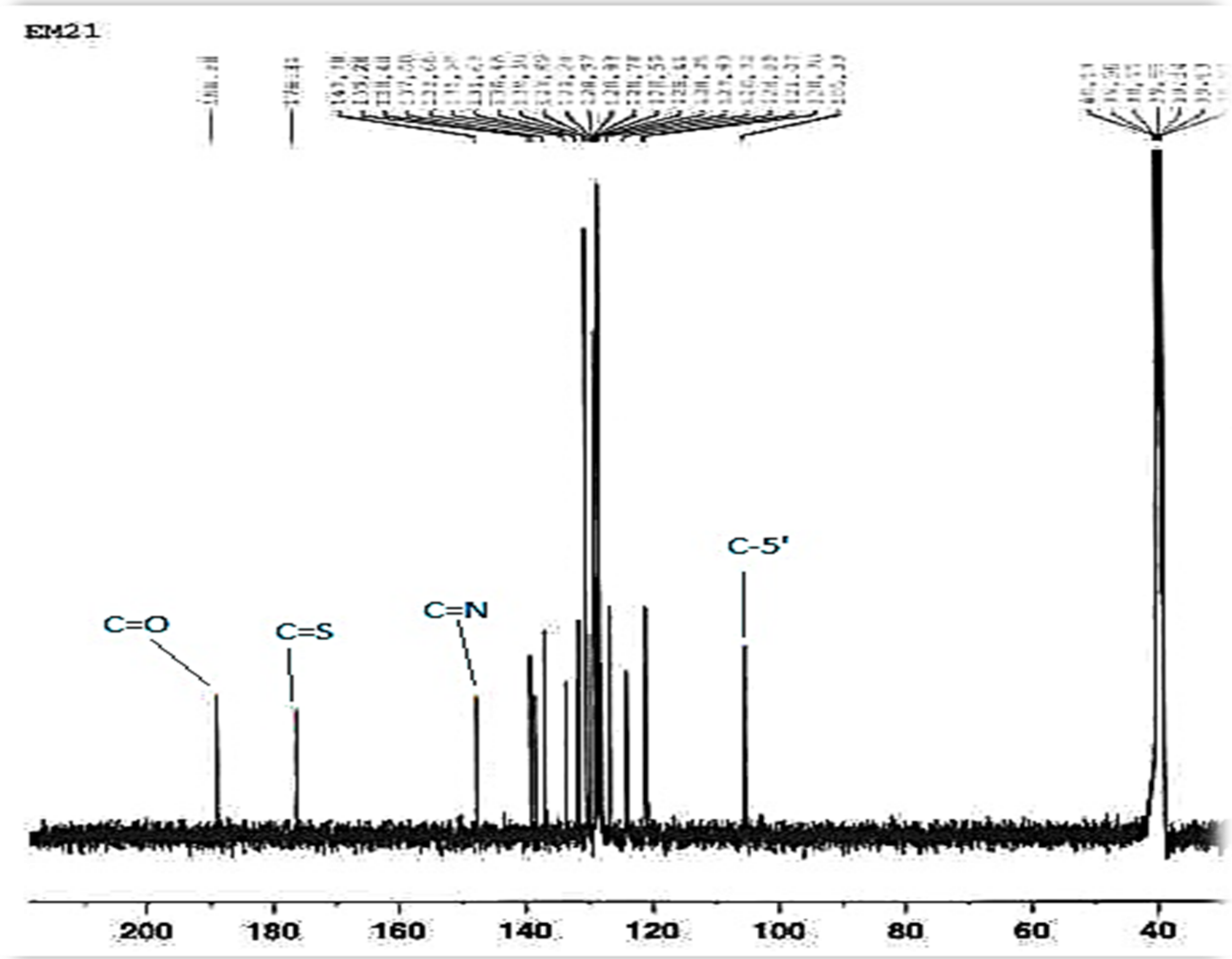

SI Figure 48.^{13}C NMR spectrum of **5h**

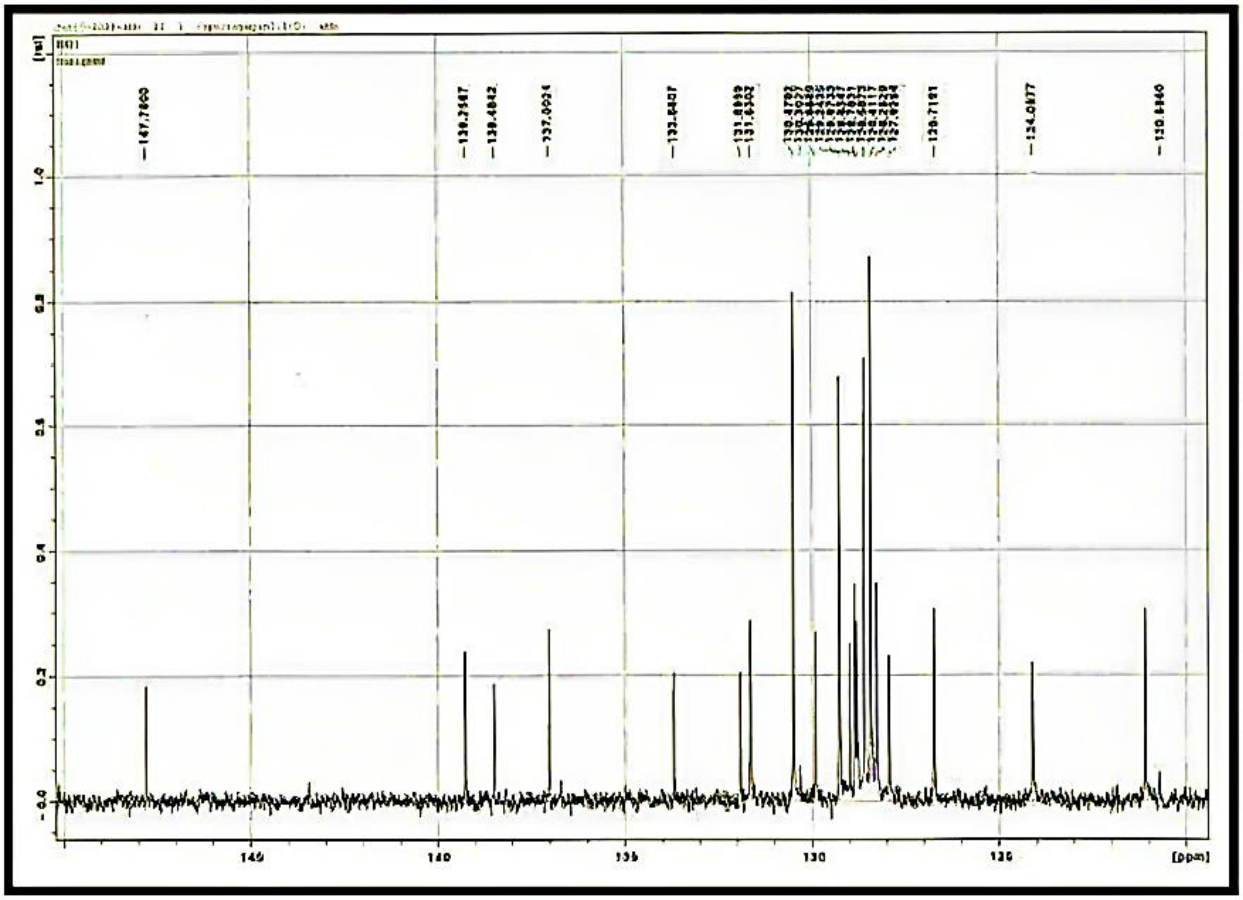

SI Figure 49. ^{13}C NMR spectrum of **5h**

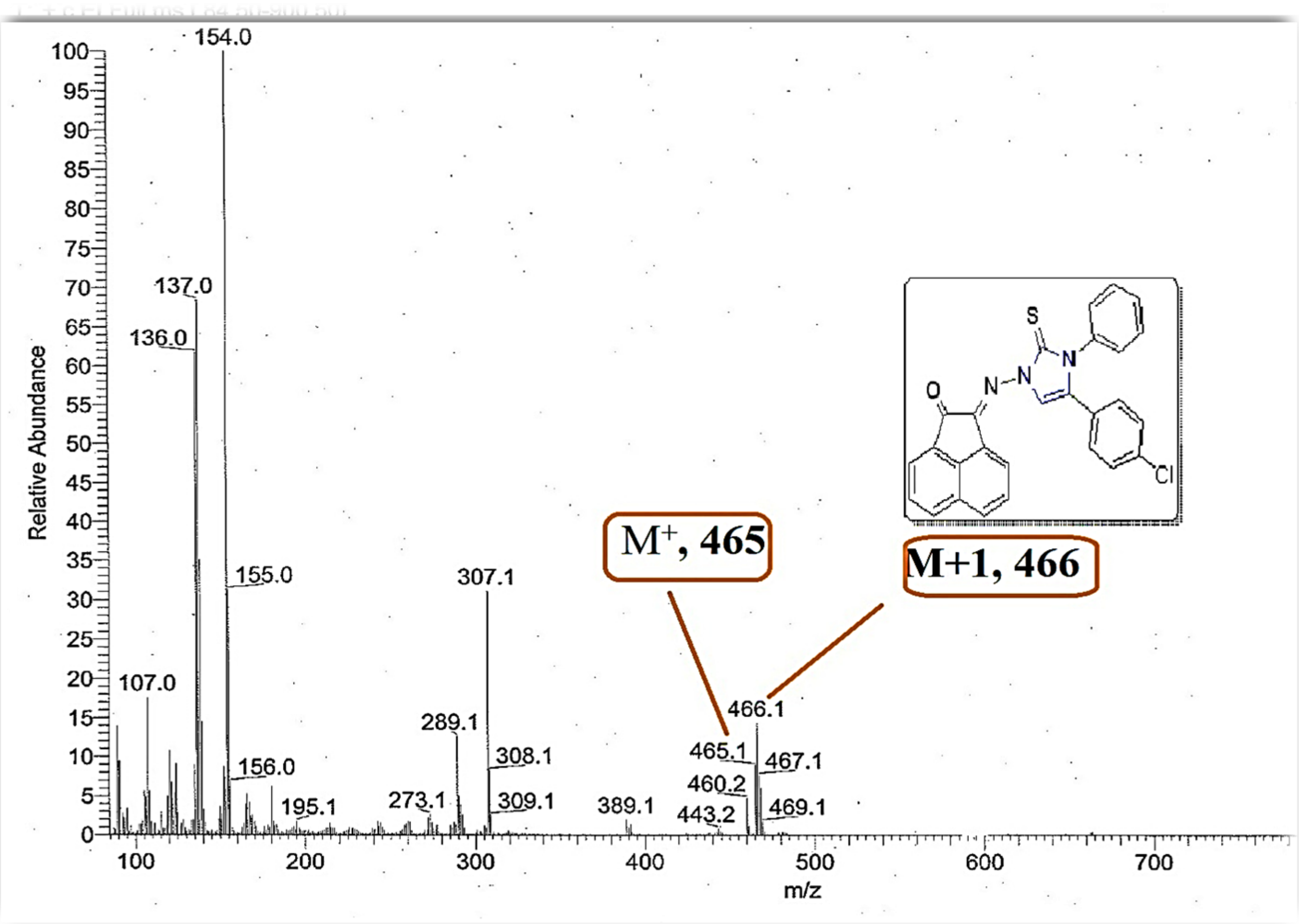

SI Figure 50. Mass spectroscopy of **5h**

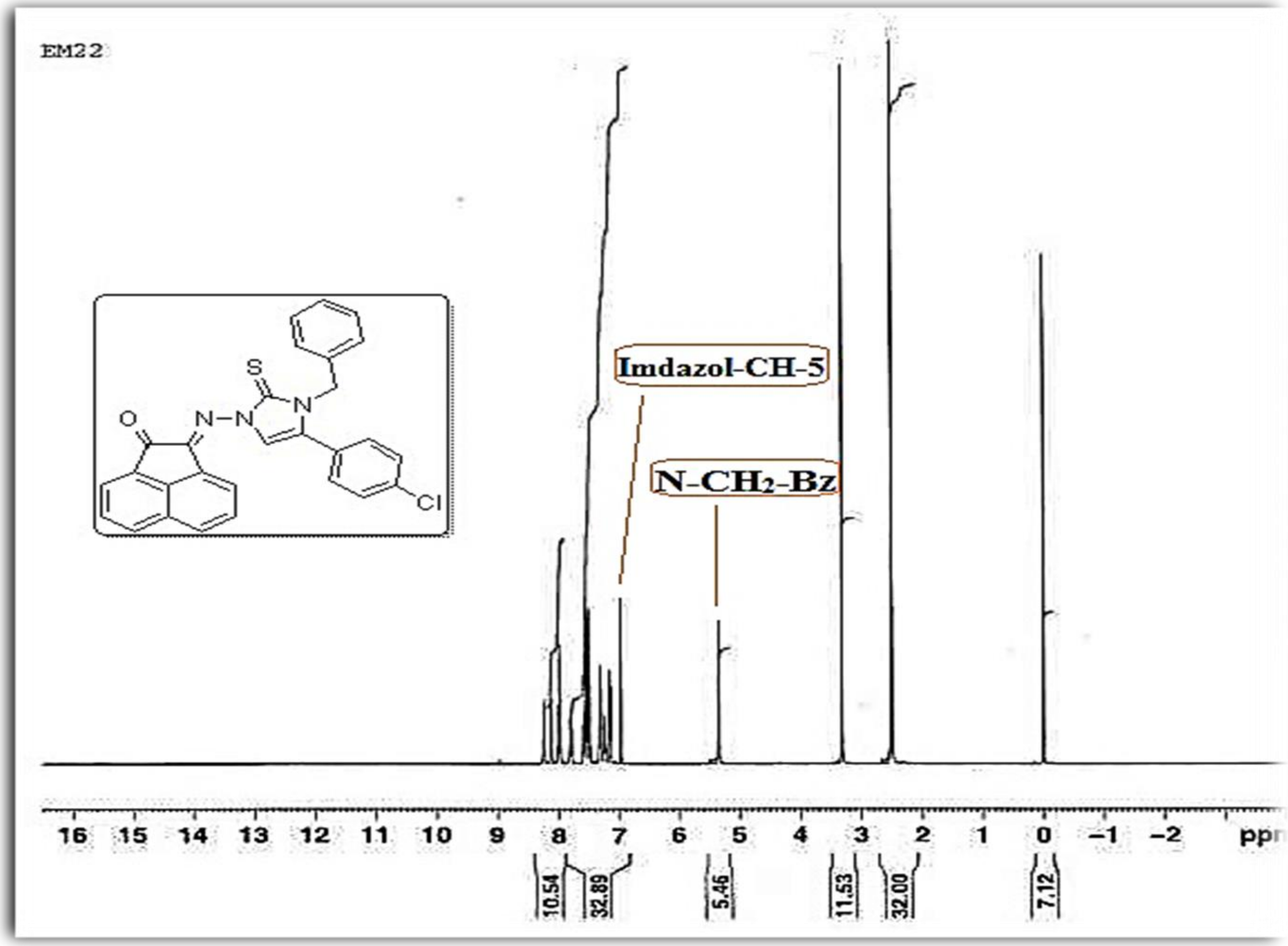

Figure 51. ^{1}H NMR spectrum of **5i.**

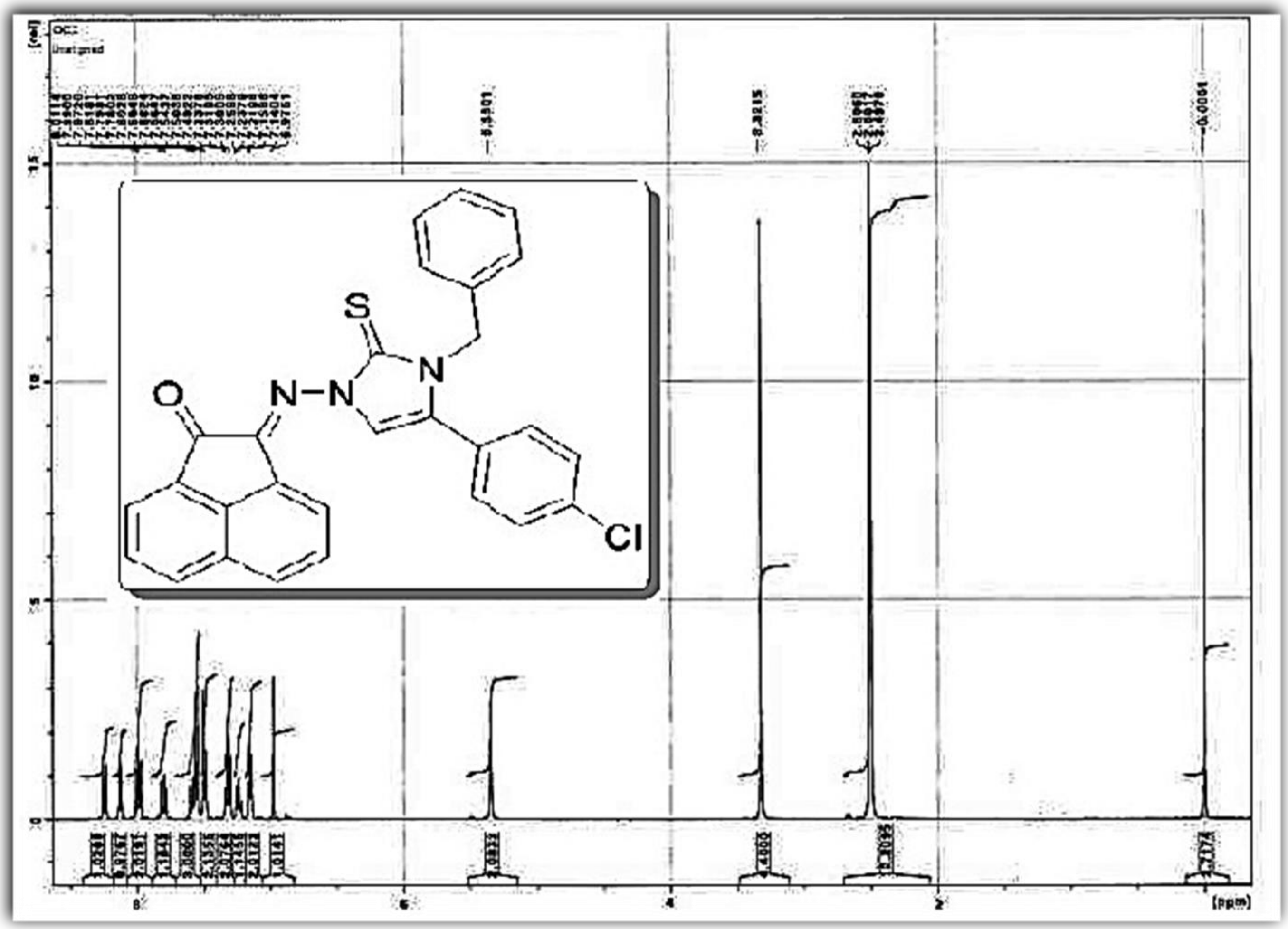

SI Figure 52. Expanded ^{1}H NMR spectrum of **5i**

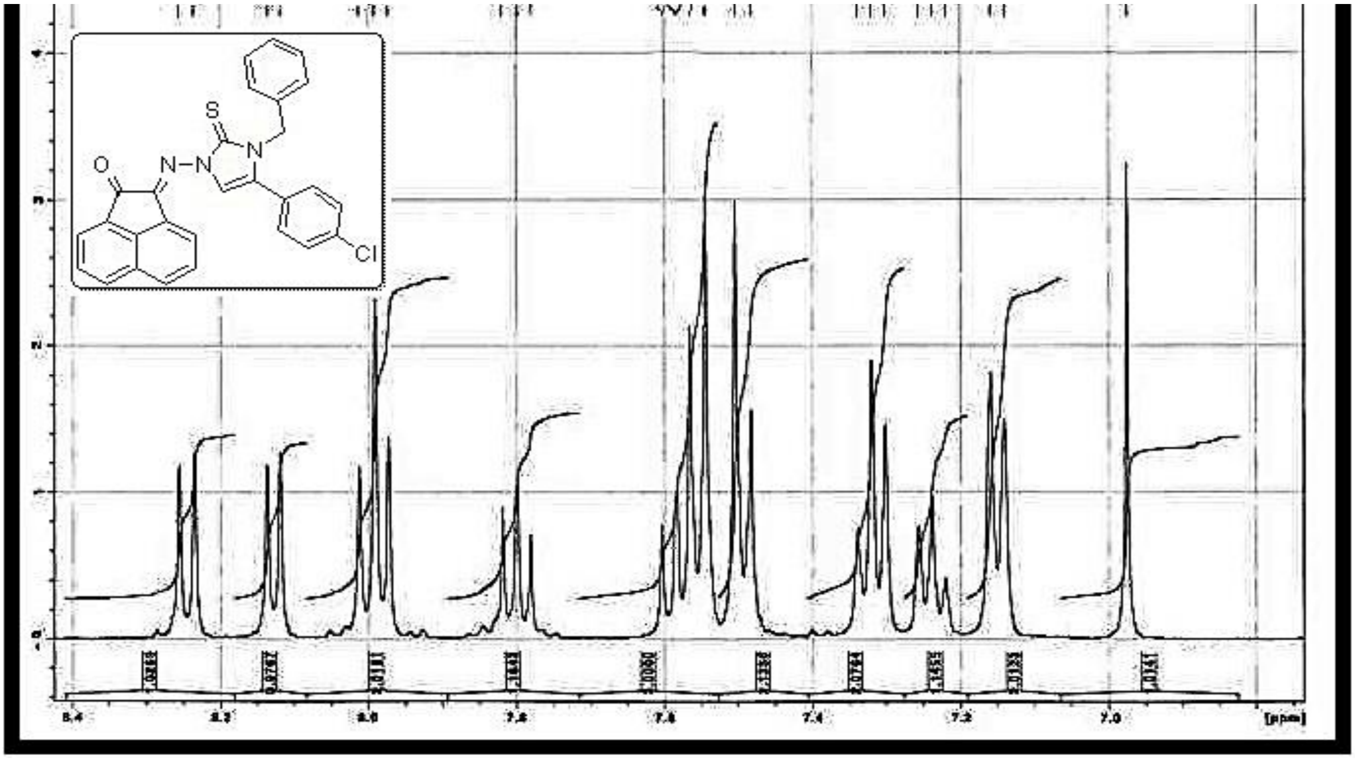

SI Figure 53. Expanded ^{1}H NMR spectrum of **5i**

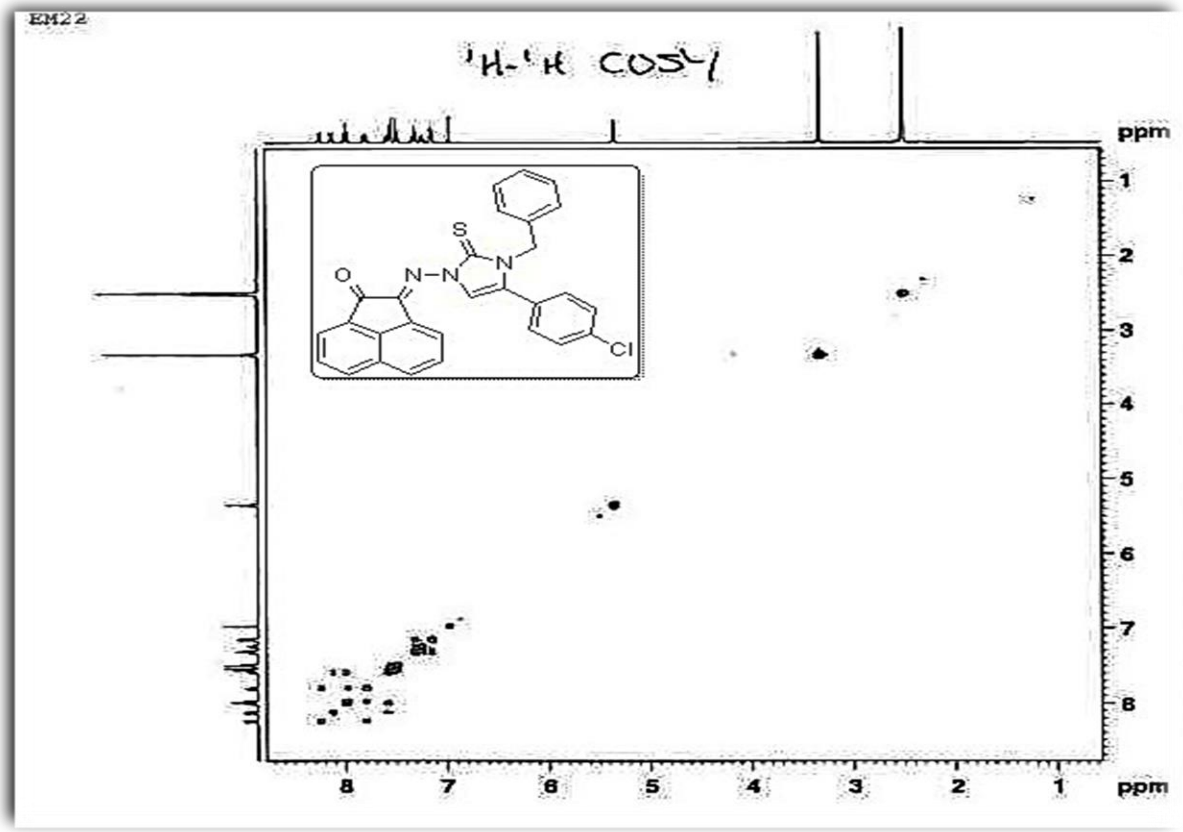

SI Figure 54. COSY H-H spectrum of **5i**

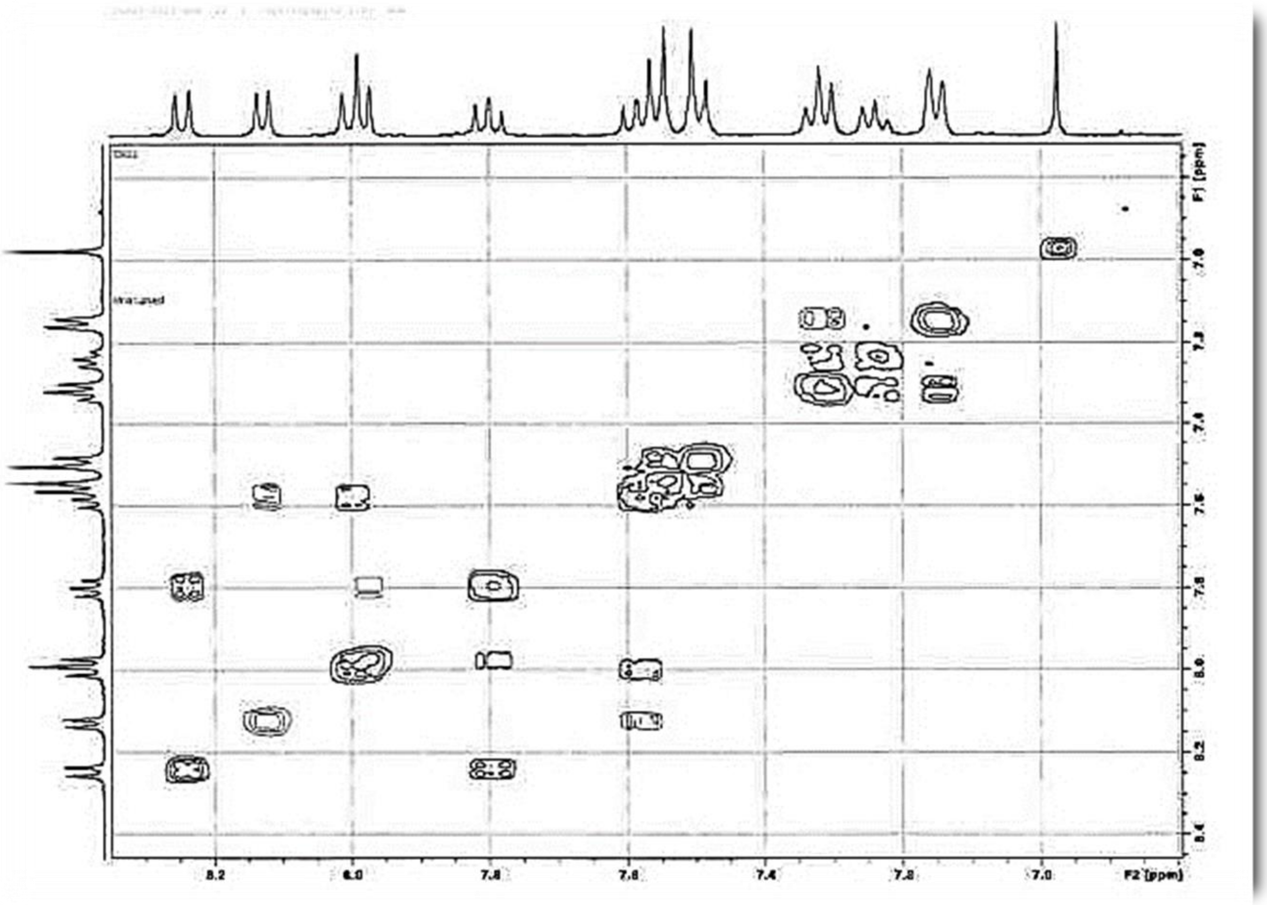

SI Figure 55. Expanded COSY H-H spectrum of **5i**

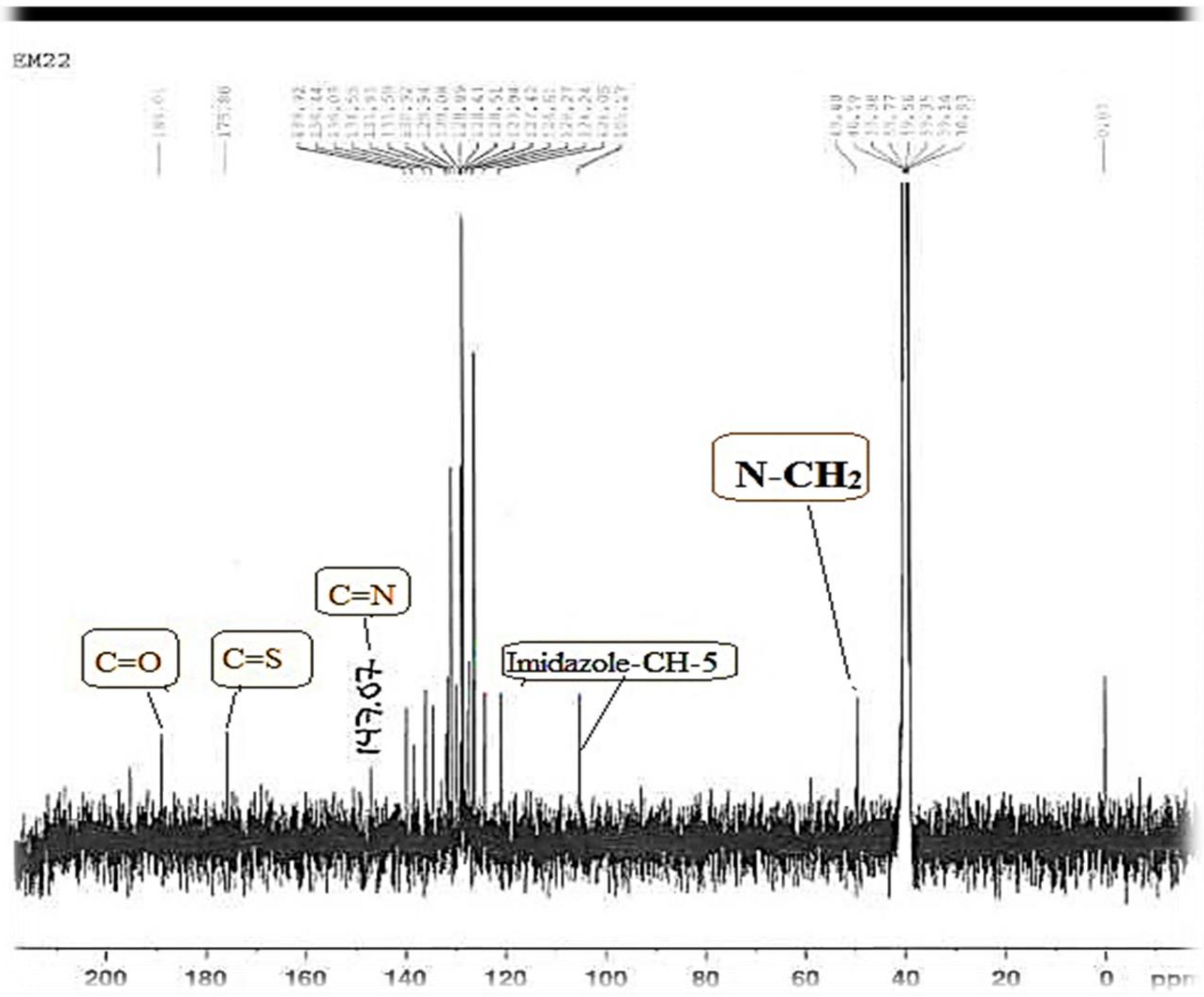

SI Figure 56. ^{13}C NMR spectrum of **5i**

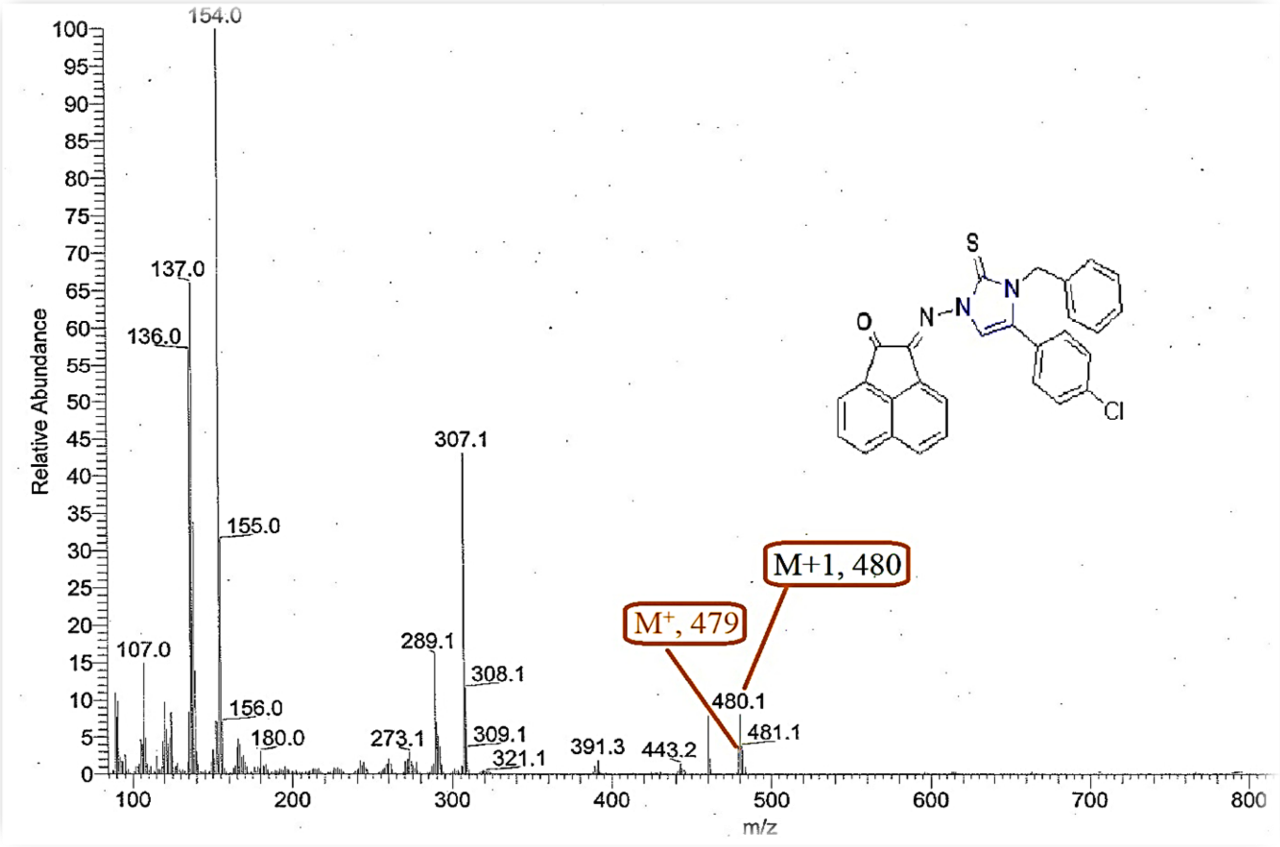

SI Figure 57. Mass spectroscopy of **5i**

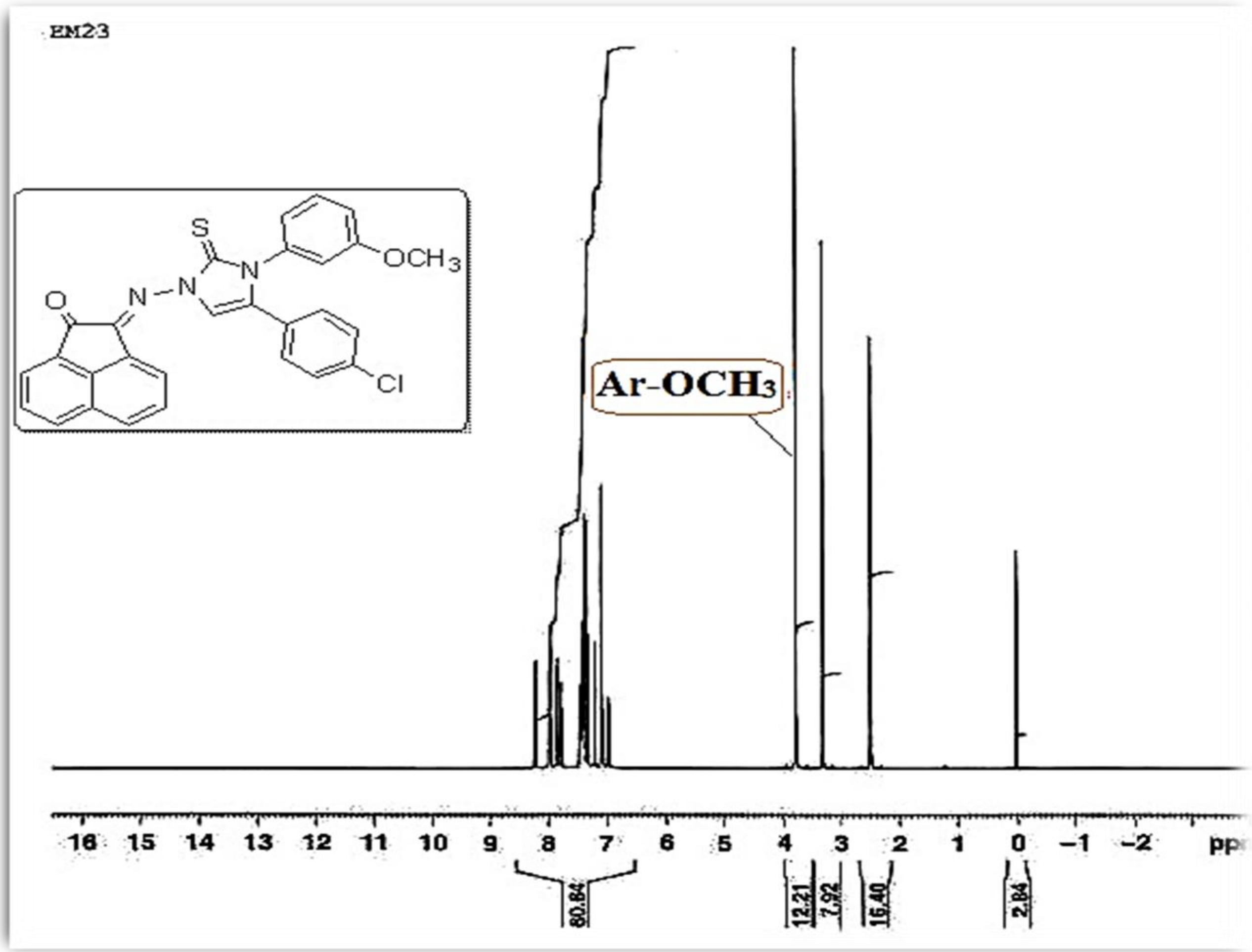

SI Figure 58. ^{1}H NMR spectrum of **5j**

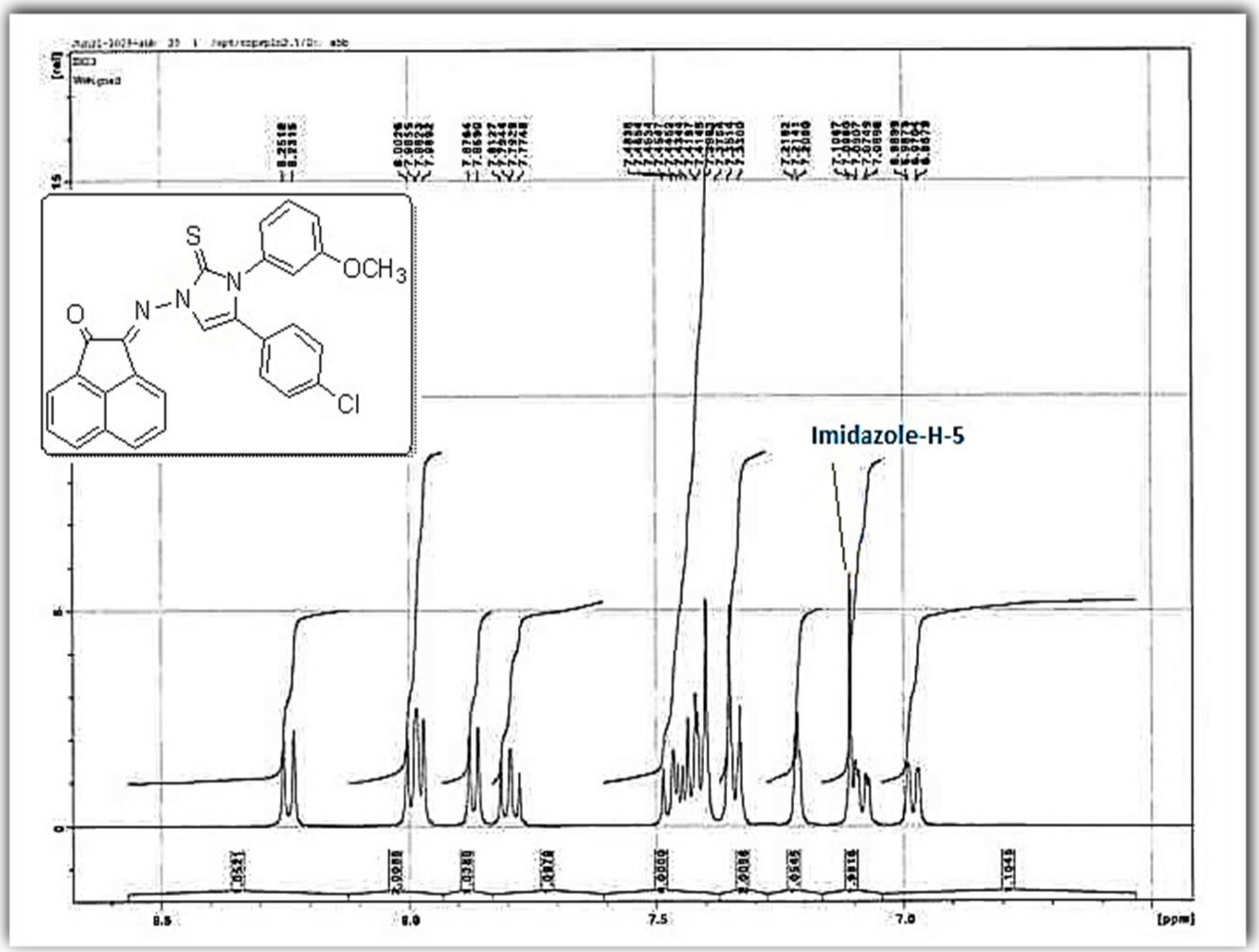

SI Figure 59. Expanded ^{1}H NMR spectrum of **5j**

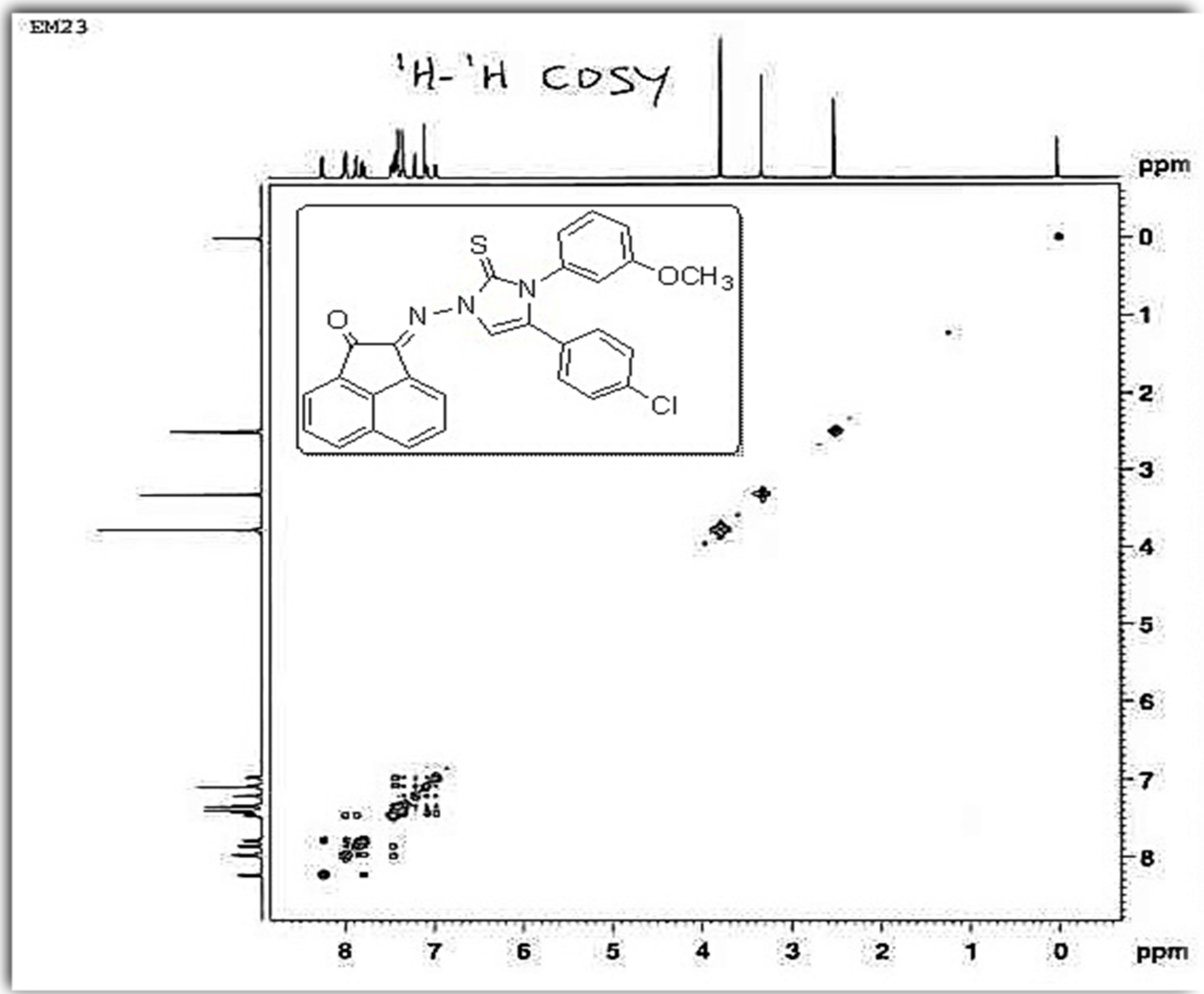

SI Figure 60. COSY H-H spectrum of **5j**

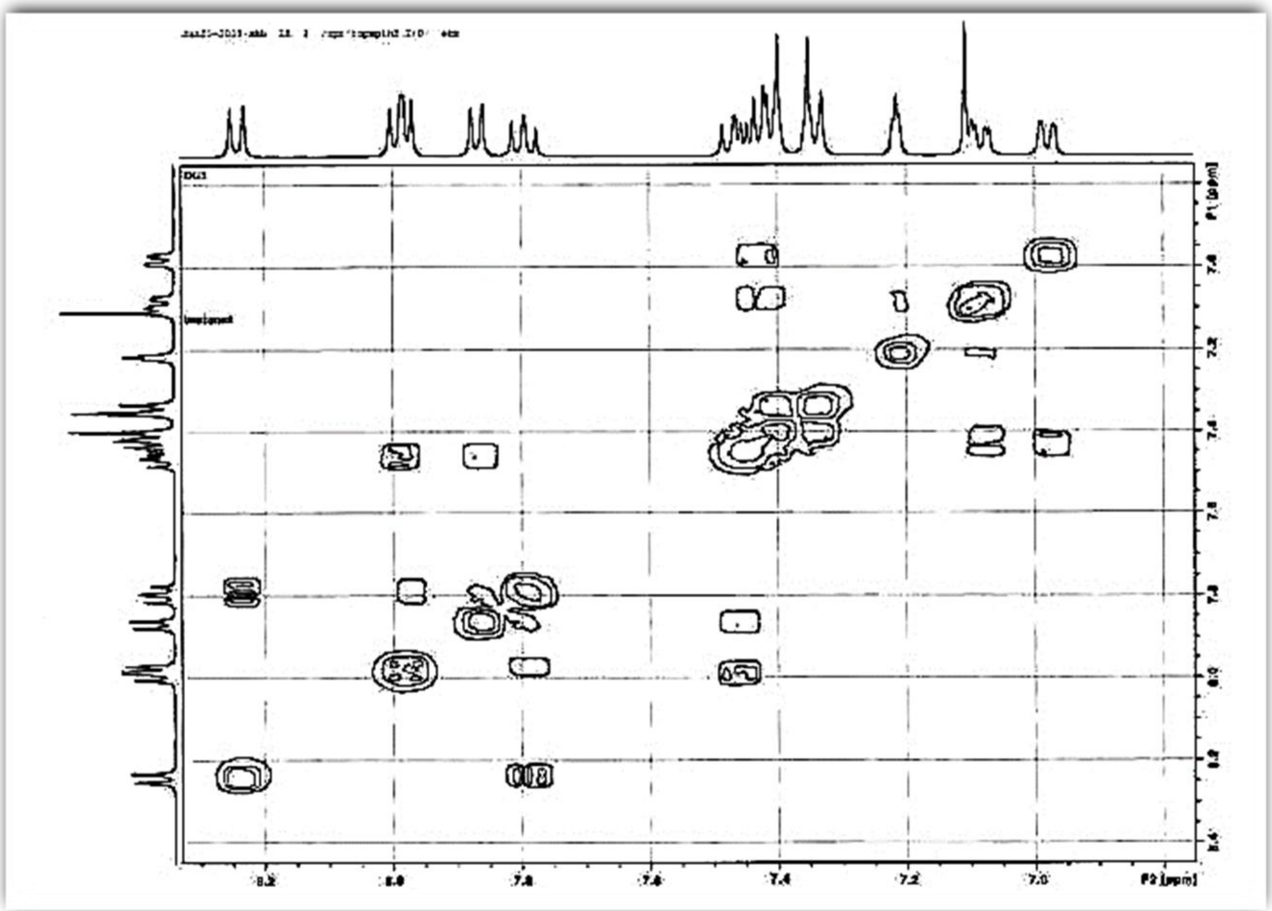

SI Figure 61. COSY H-H spectrum of **5j**

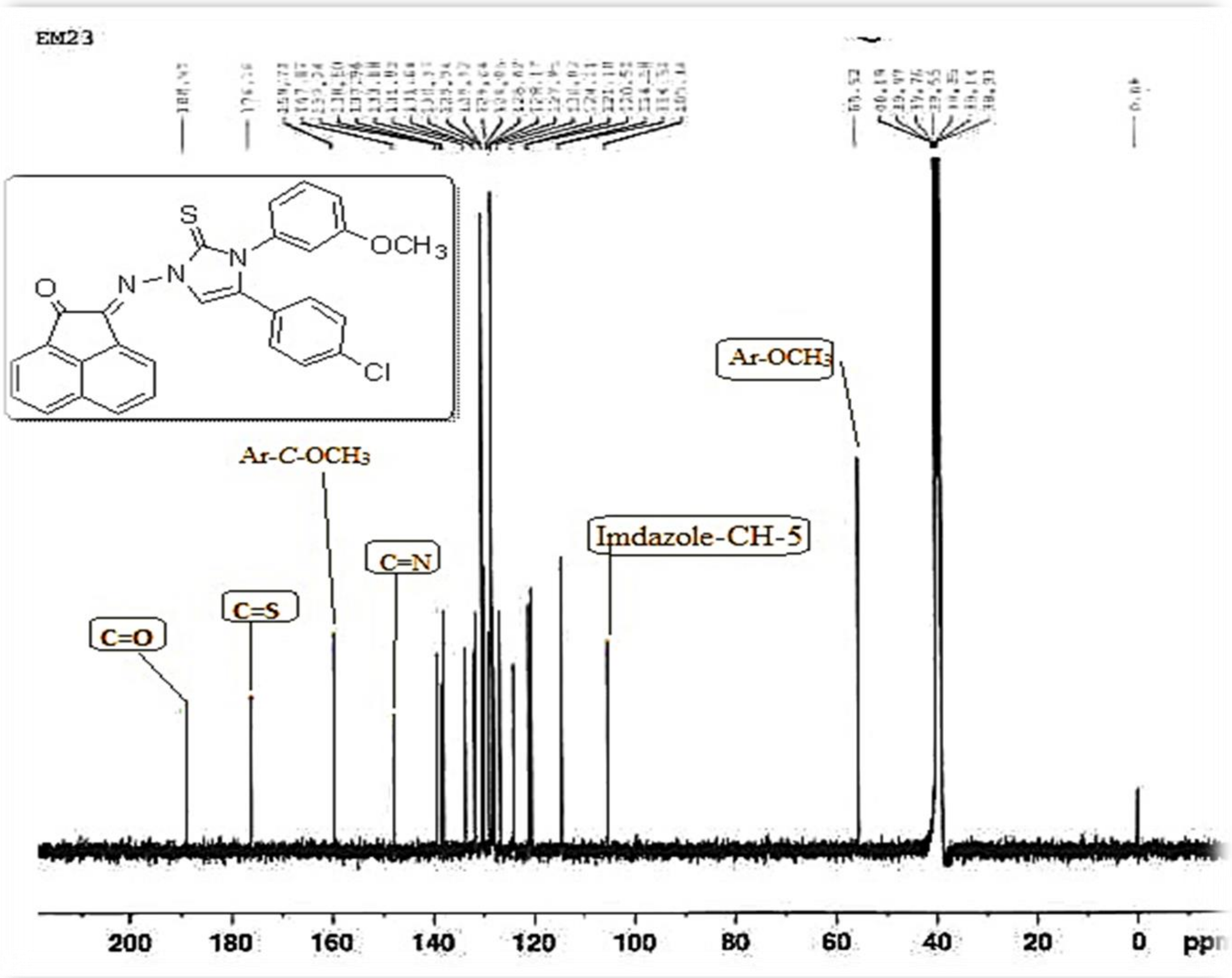

SI Figure 62. ^{13}C NMR spectrum of **5j**

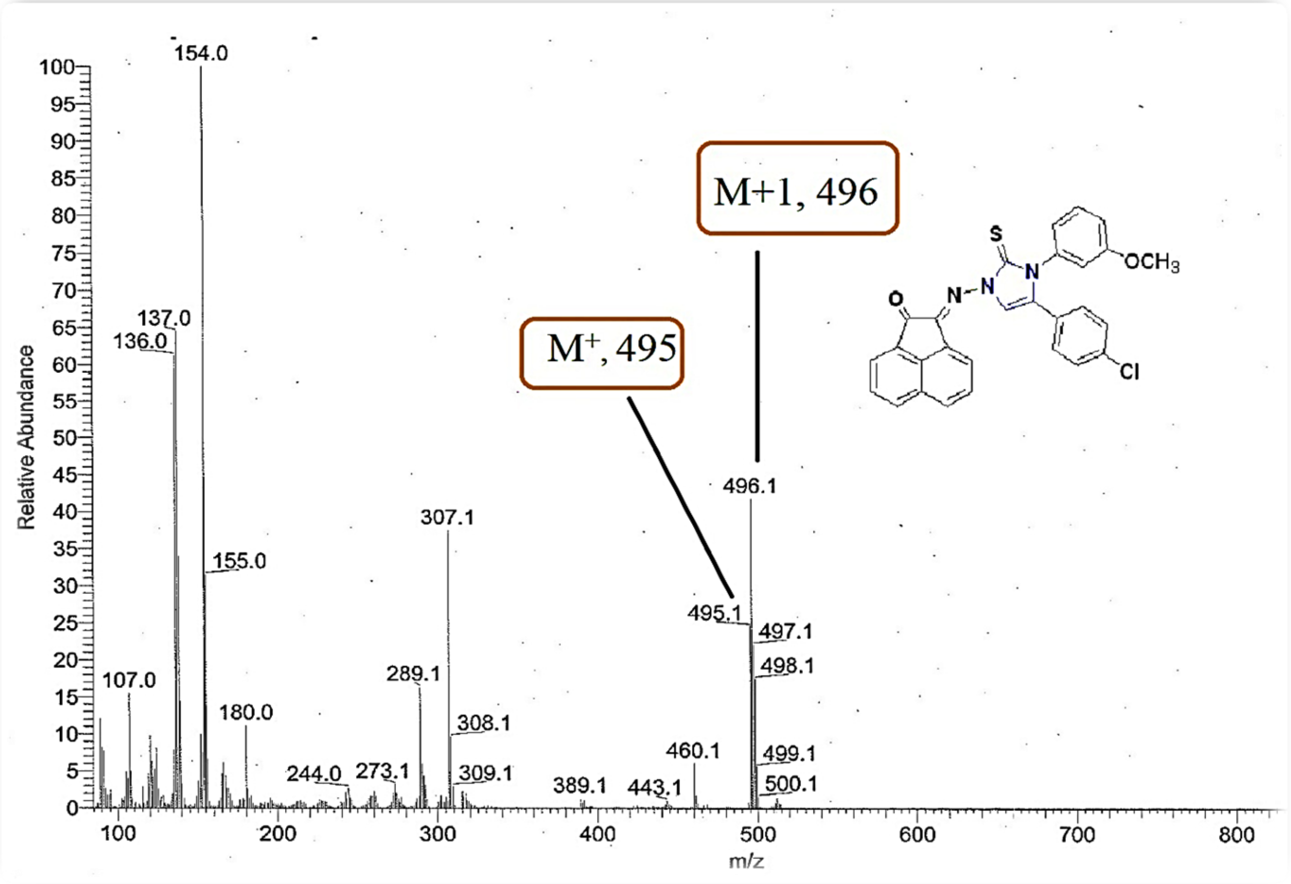

SI Figure 63. Mass spectroscopy of **5j**

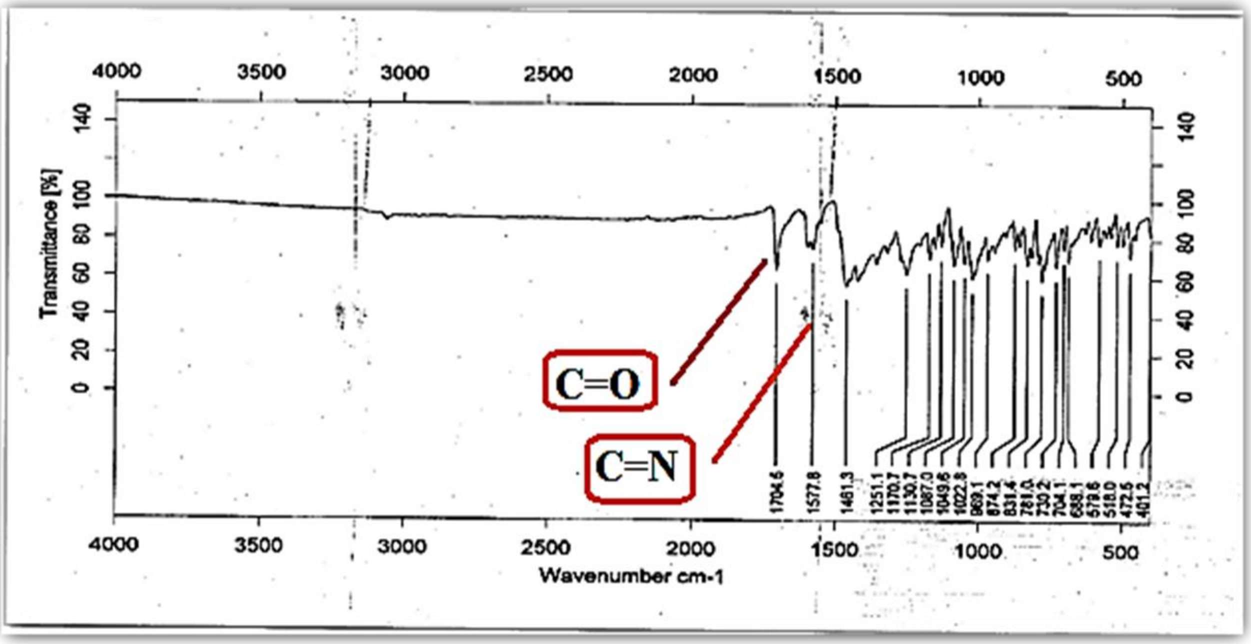

SI Figure 64. IR spectrum of compound **5j**

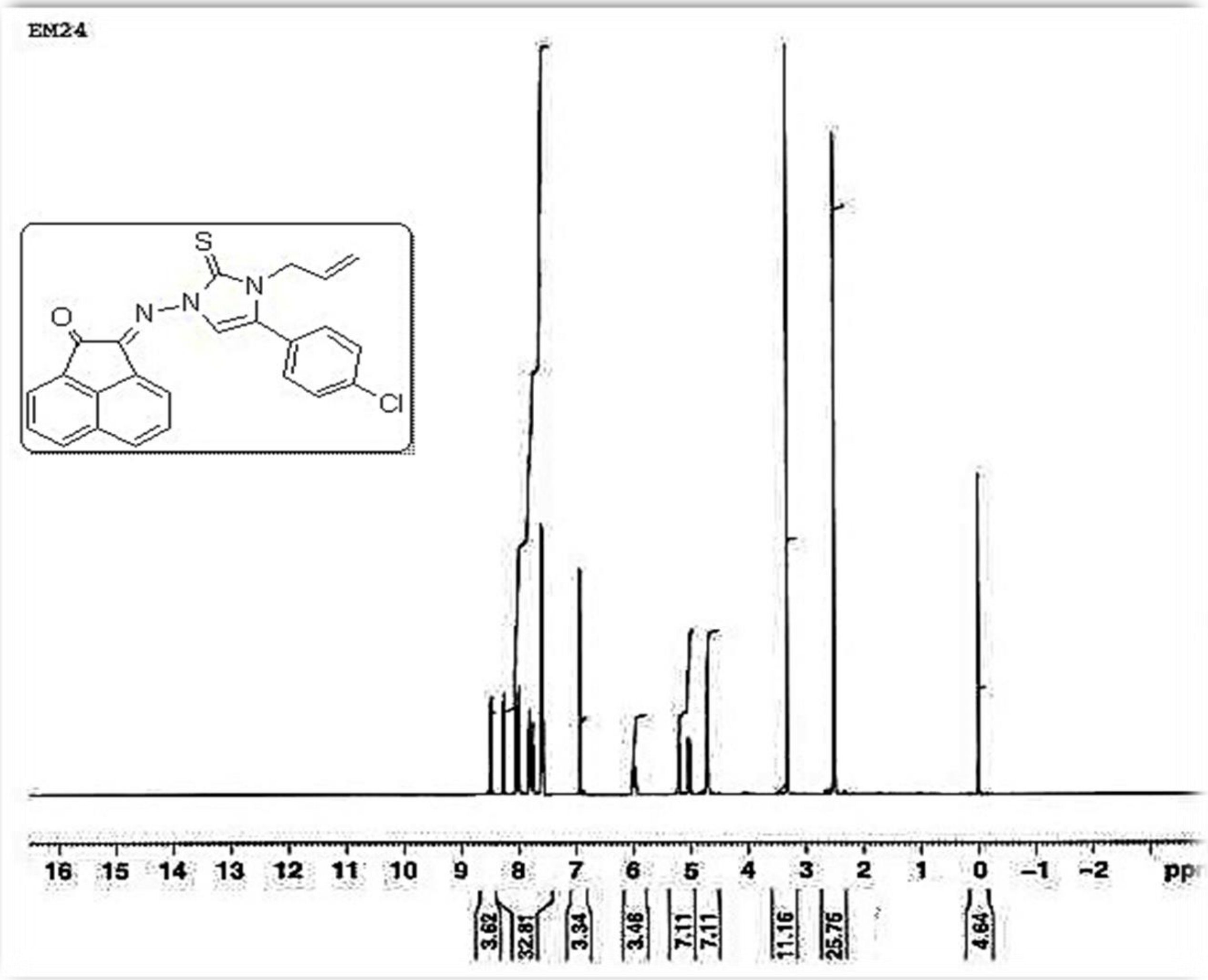

SI Figure 65. ^{1}H NMR spectrum of **5k**

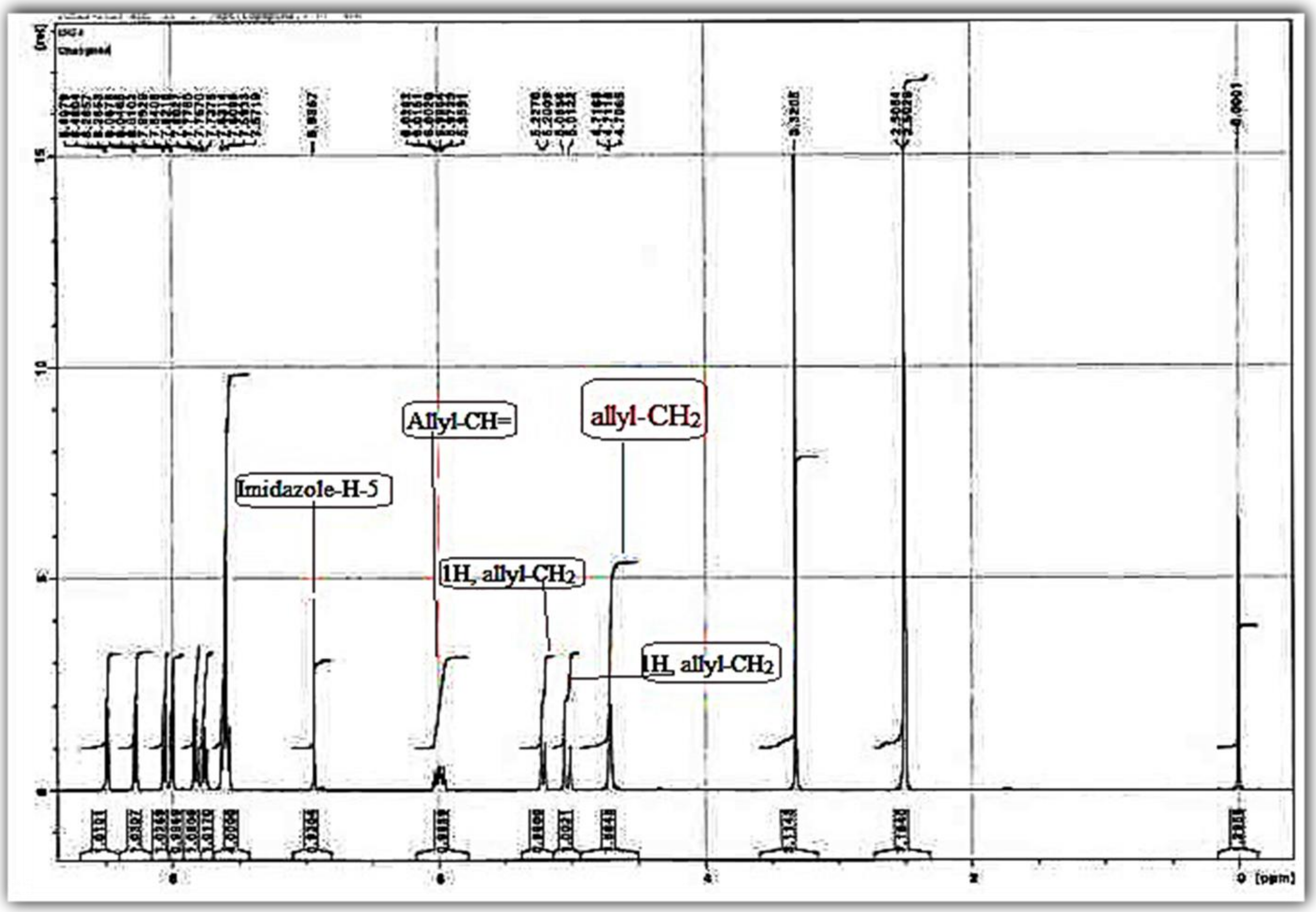

SI Figure 66. Expanded ^{1}H NMR spectrum of **5k**

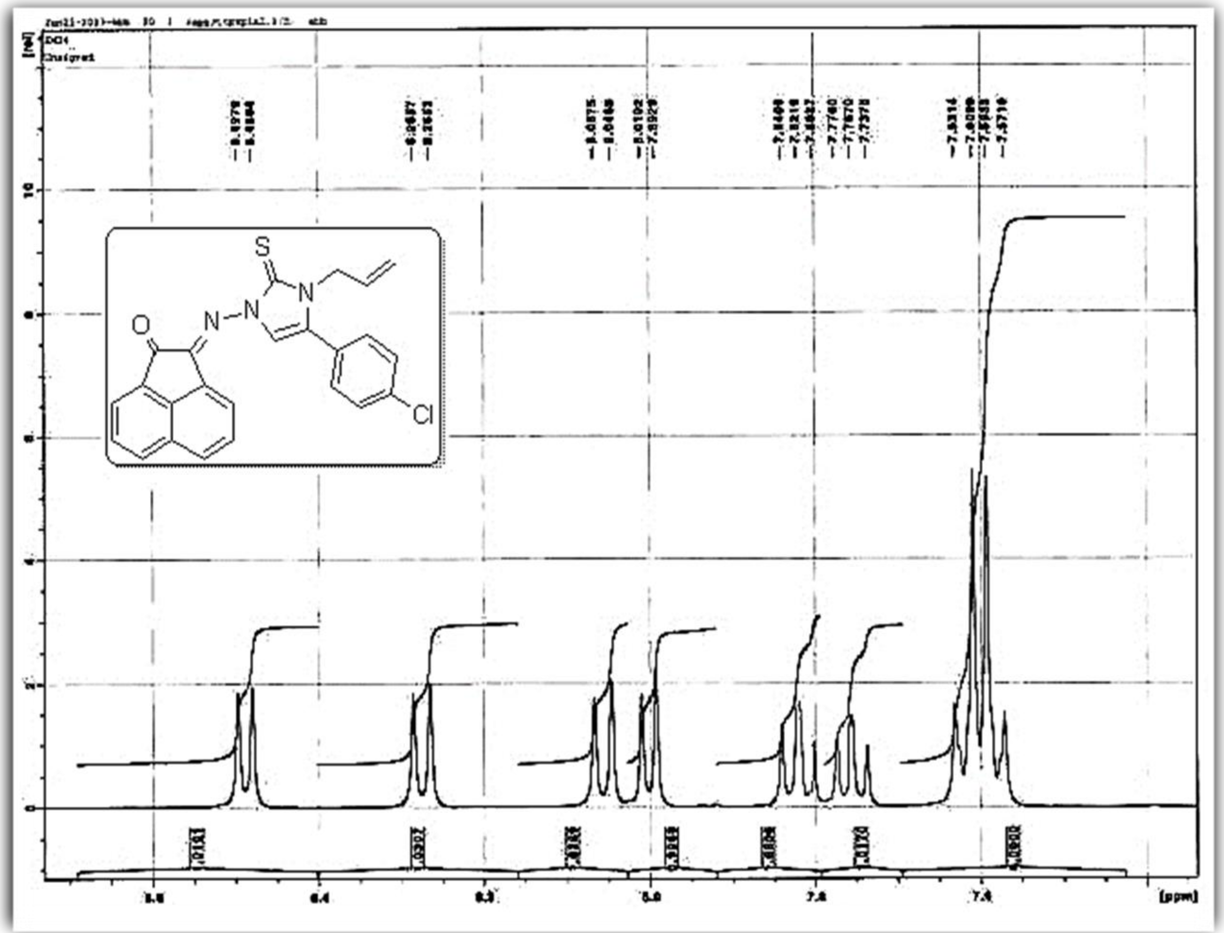

SI Figure 67. Expanded ^{1}H NMR spectrum of **5k**

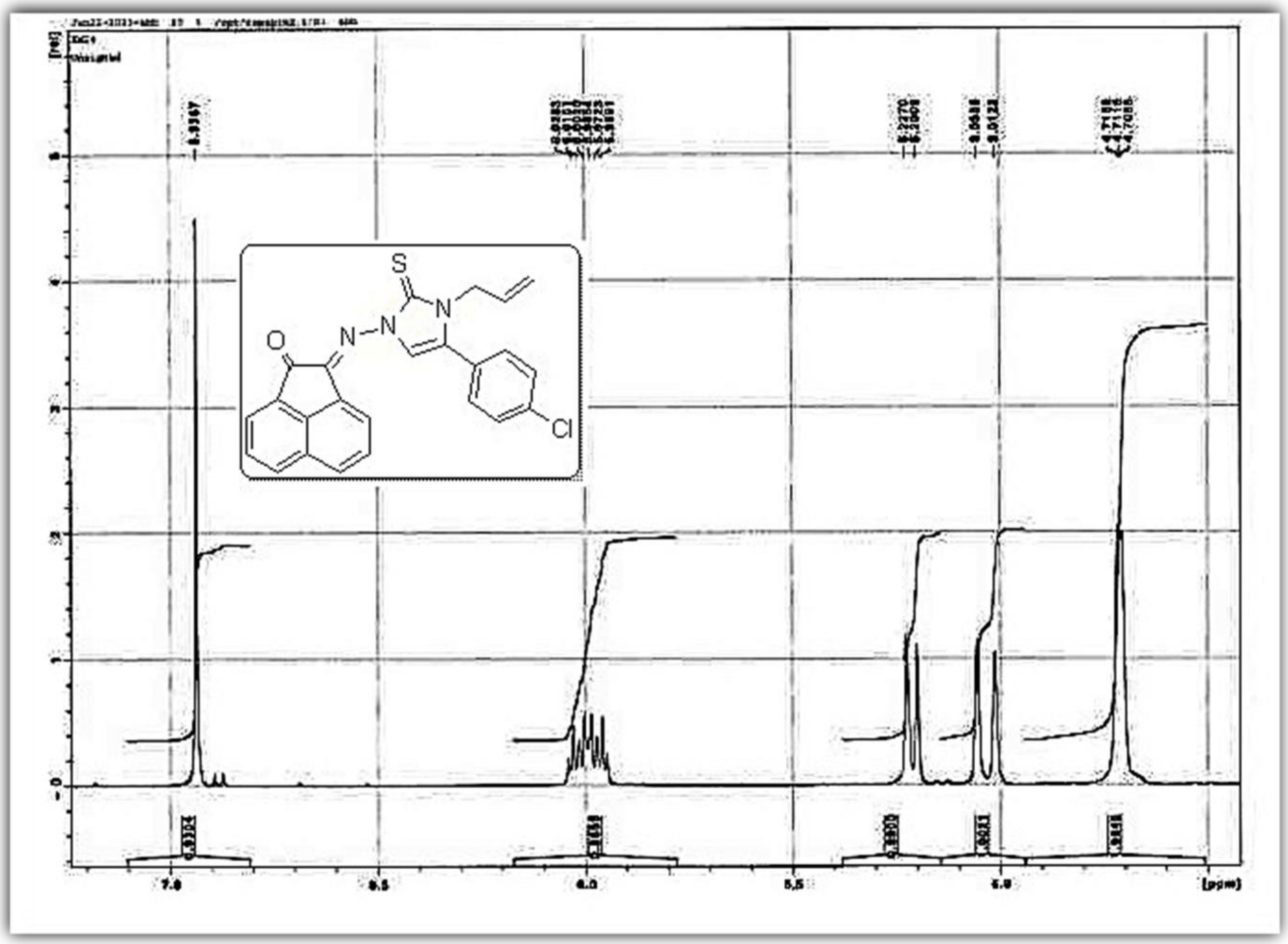

SI Figure 68. Expanded ^{1}H NMR spectrum of **5k**

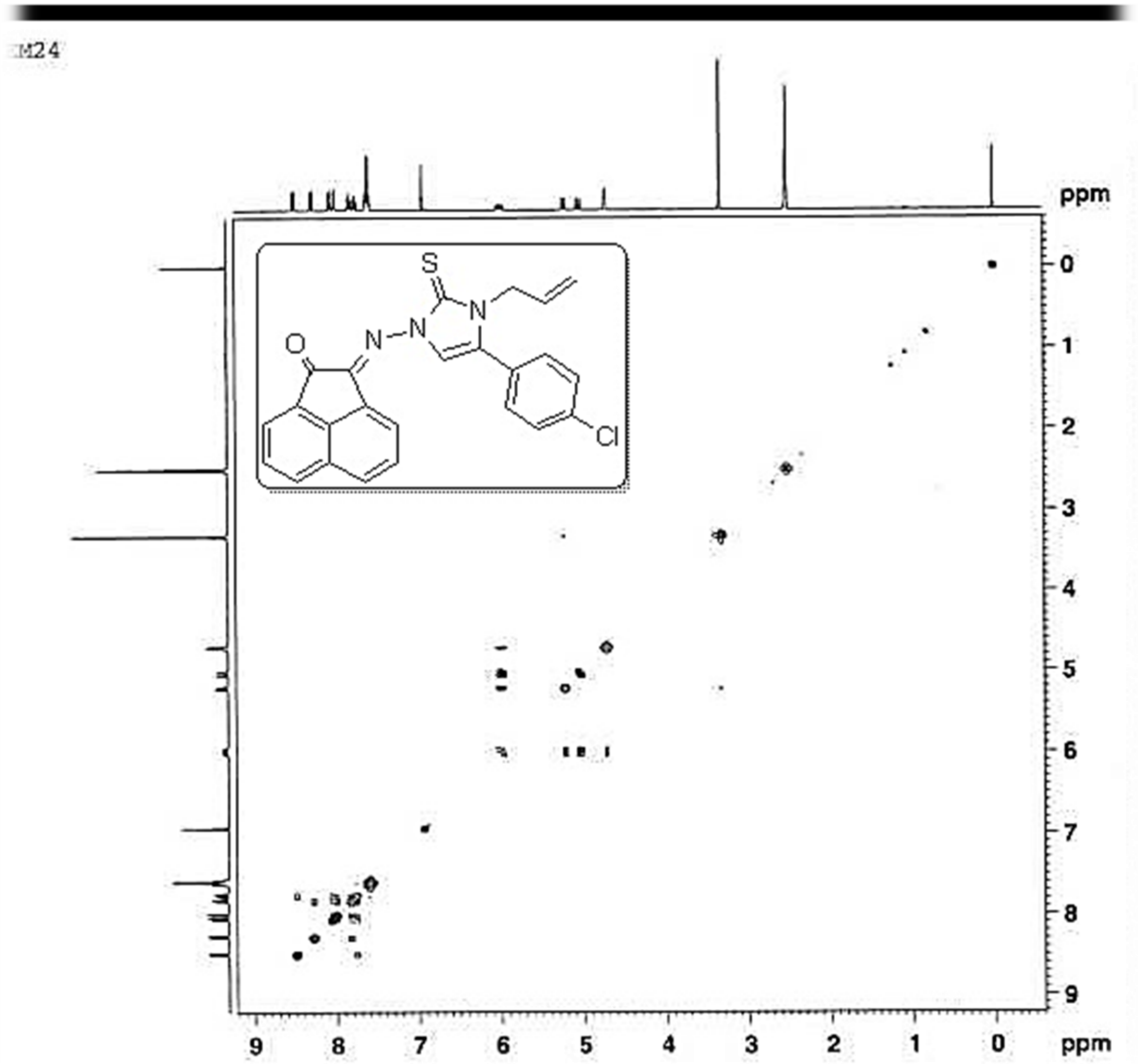

SI Figure 69. COSY H-H spectrum of **5k**

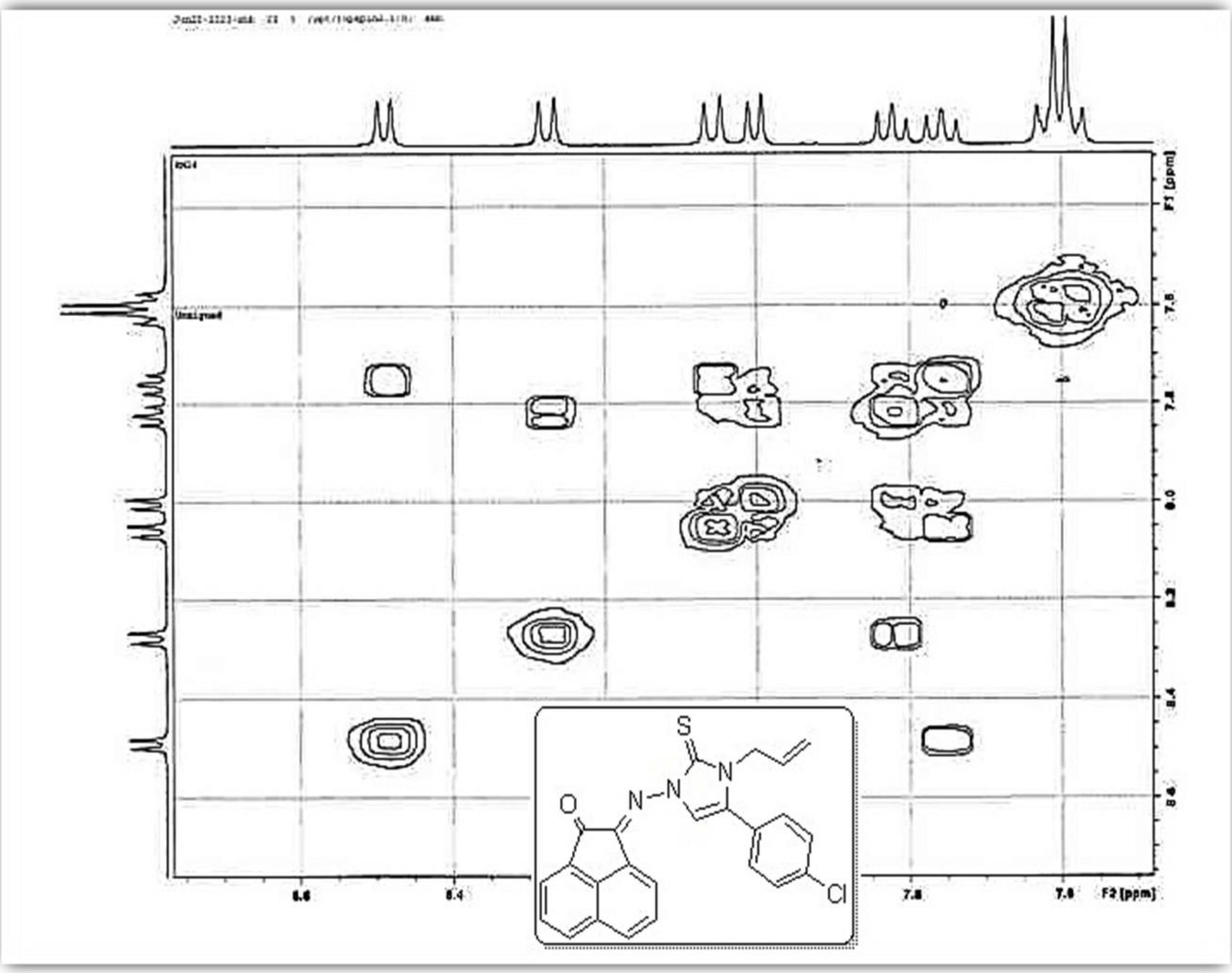

SI Figure 70. Expanded COSY H-H spectrum of **5k**

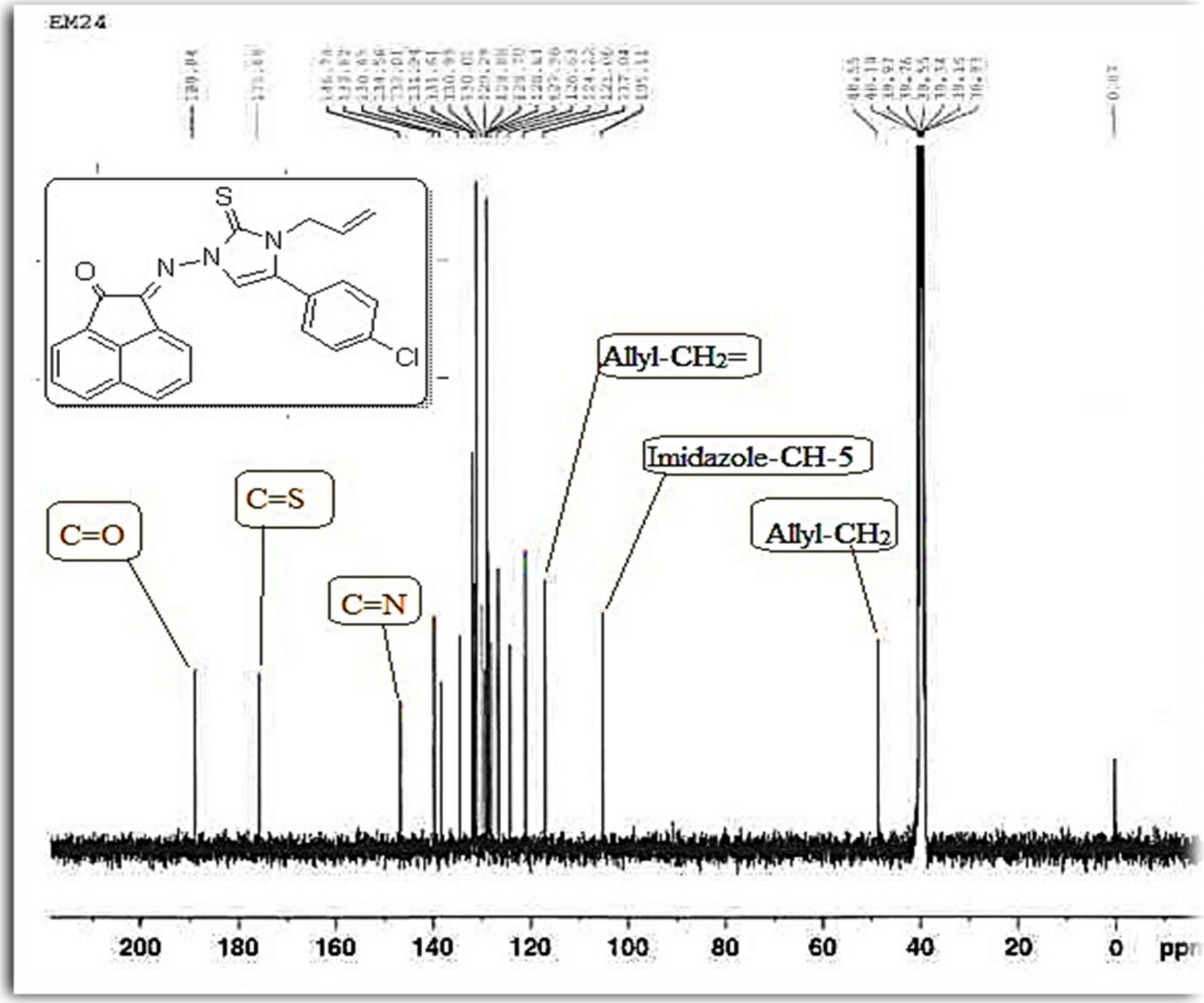

SI Figure 71. ^{13}C NMR spectrum of **5k**

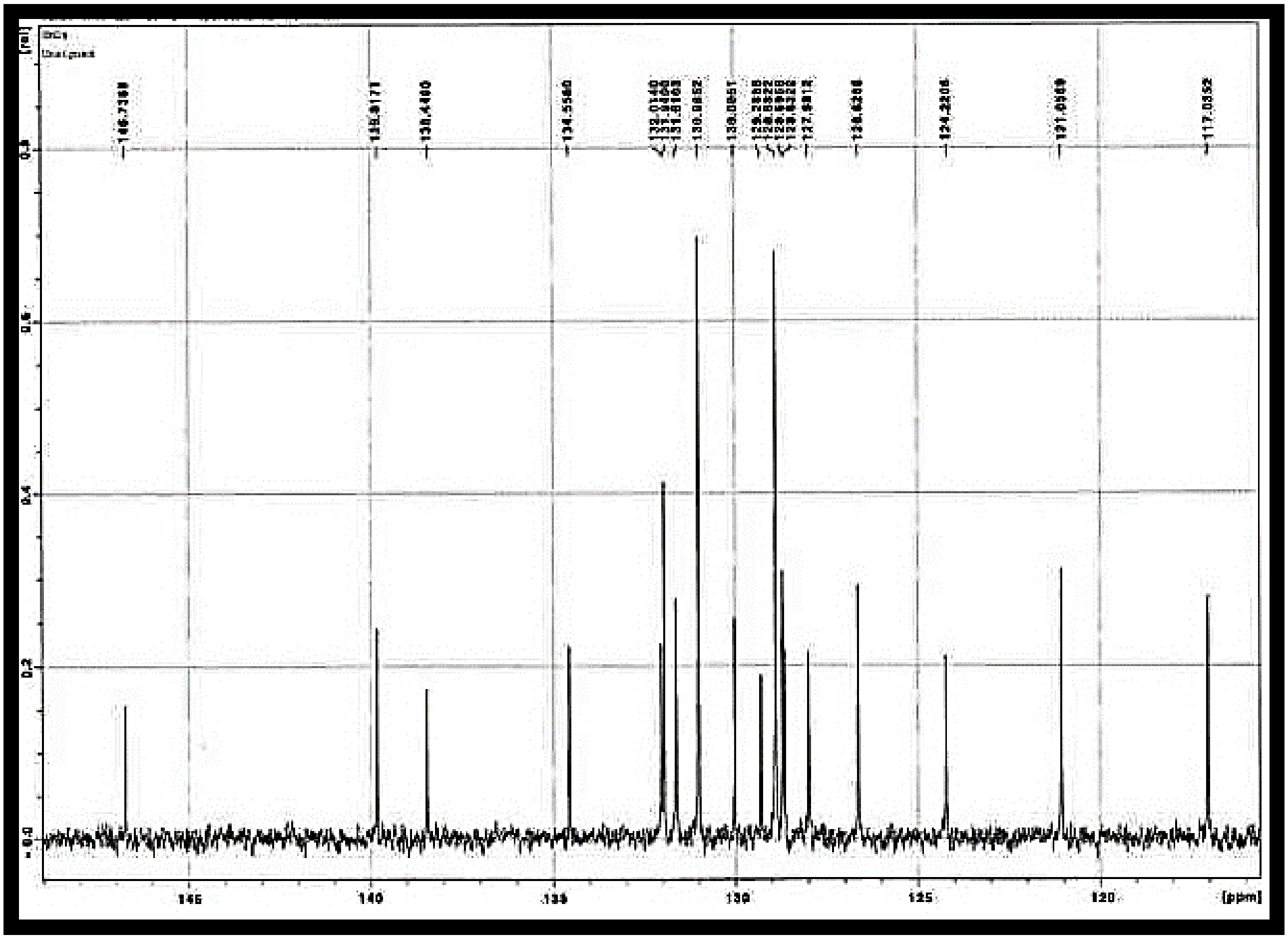

SI Figure 72. Expanded ^{13}C NMR spectrum of **5k**

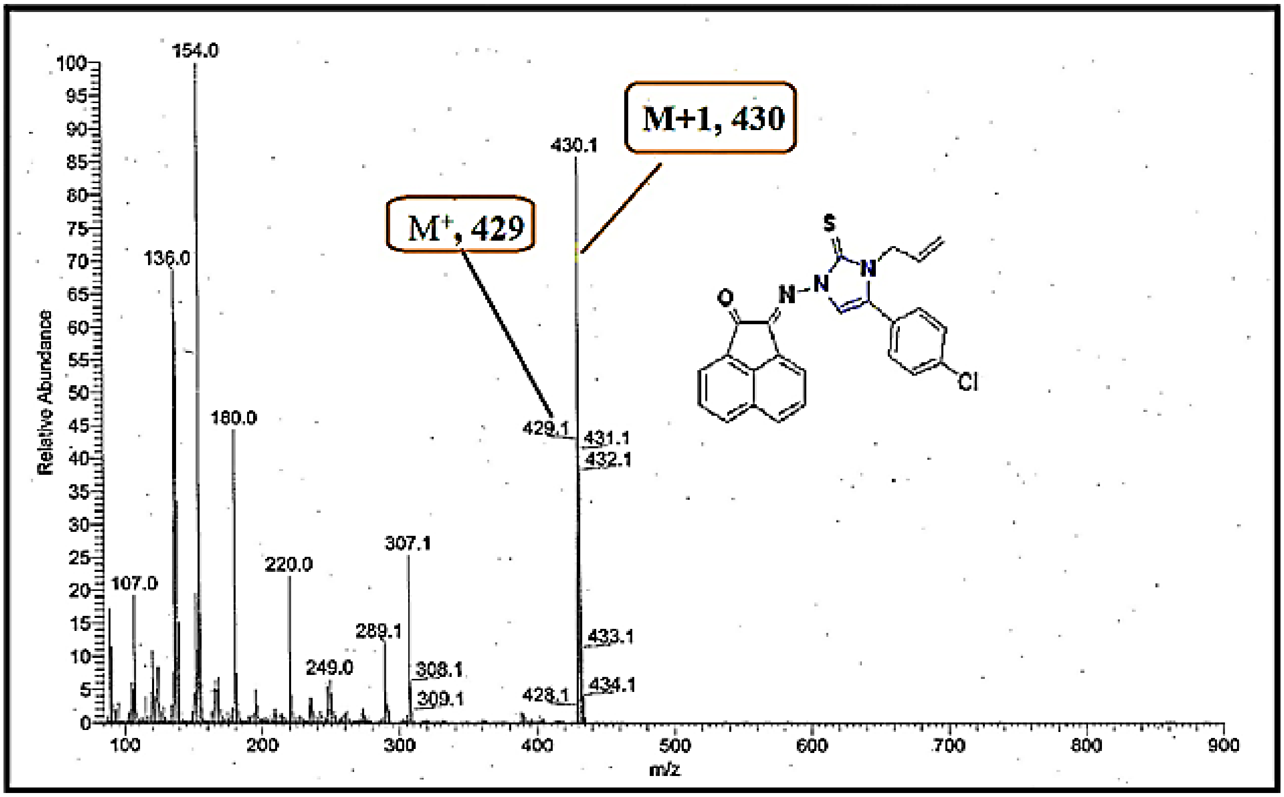

SI Figure 73. Mass spectroscopy of **5k**

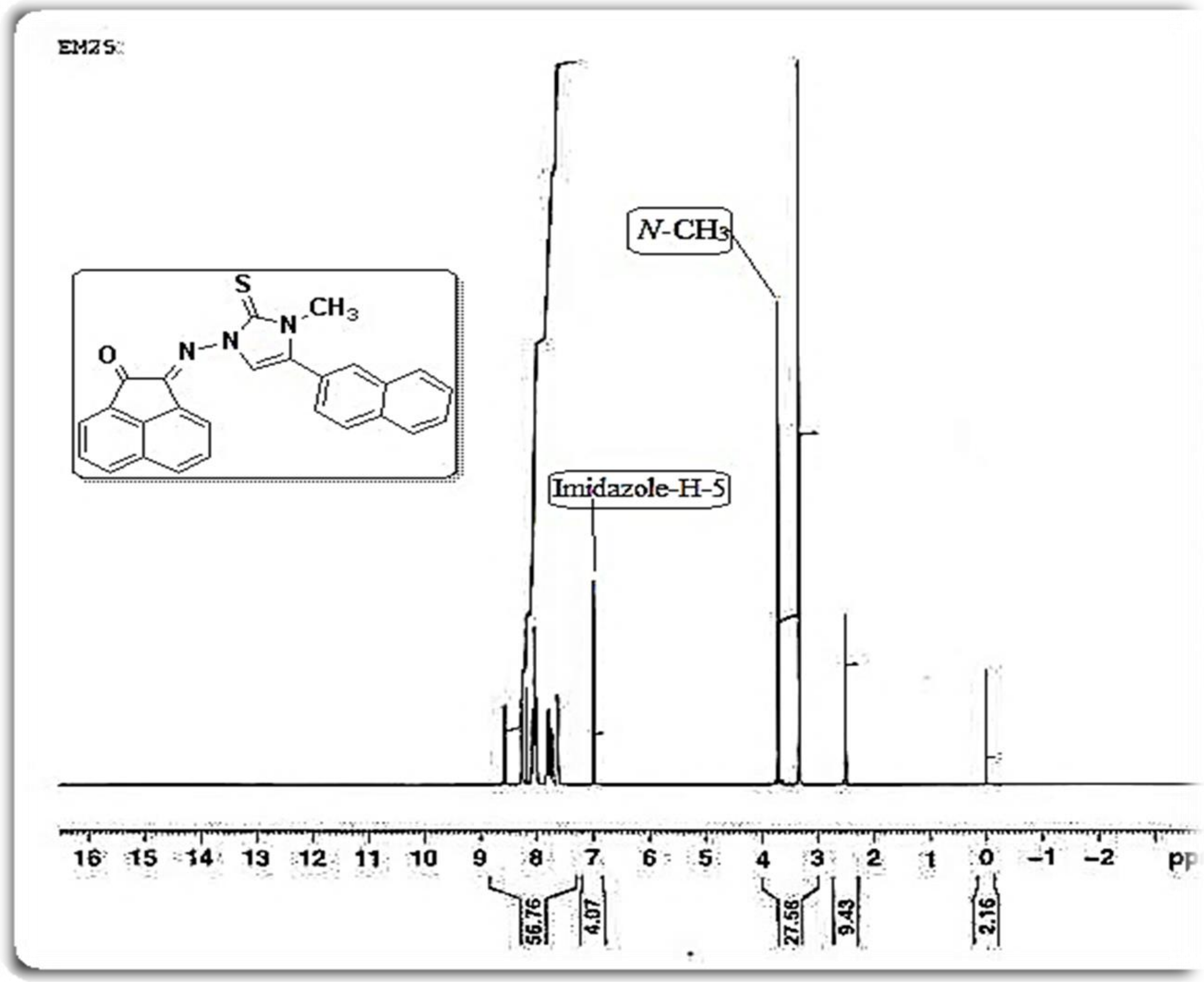

SI Figure 74.^{1}H NMR spectrum of compound **5l**

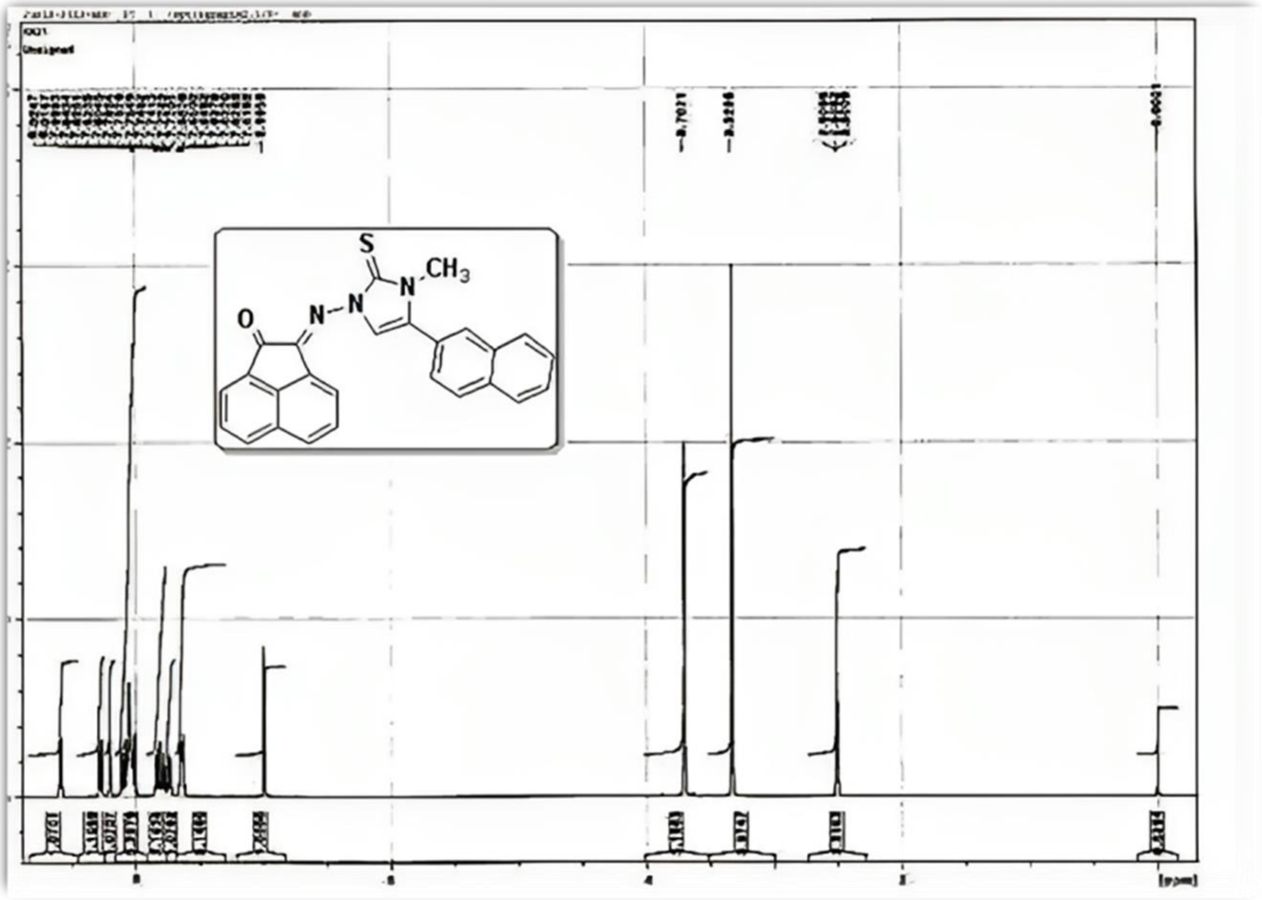

SI Figure 75. Extended ^{1}H NMR spectrum of compound **5l**

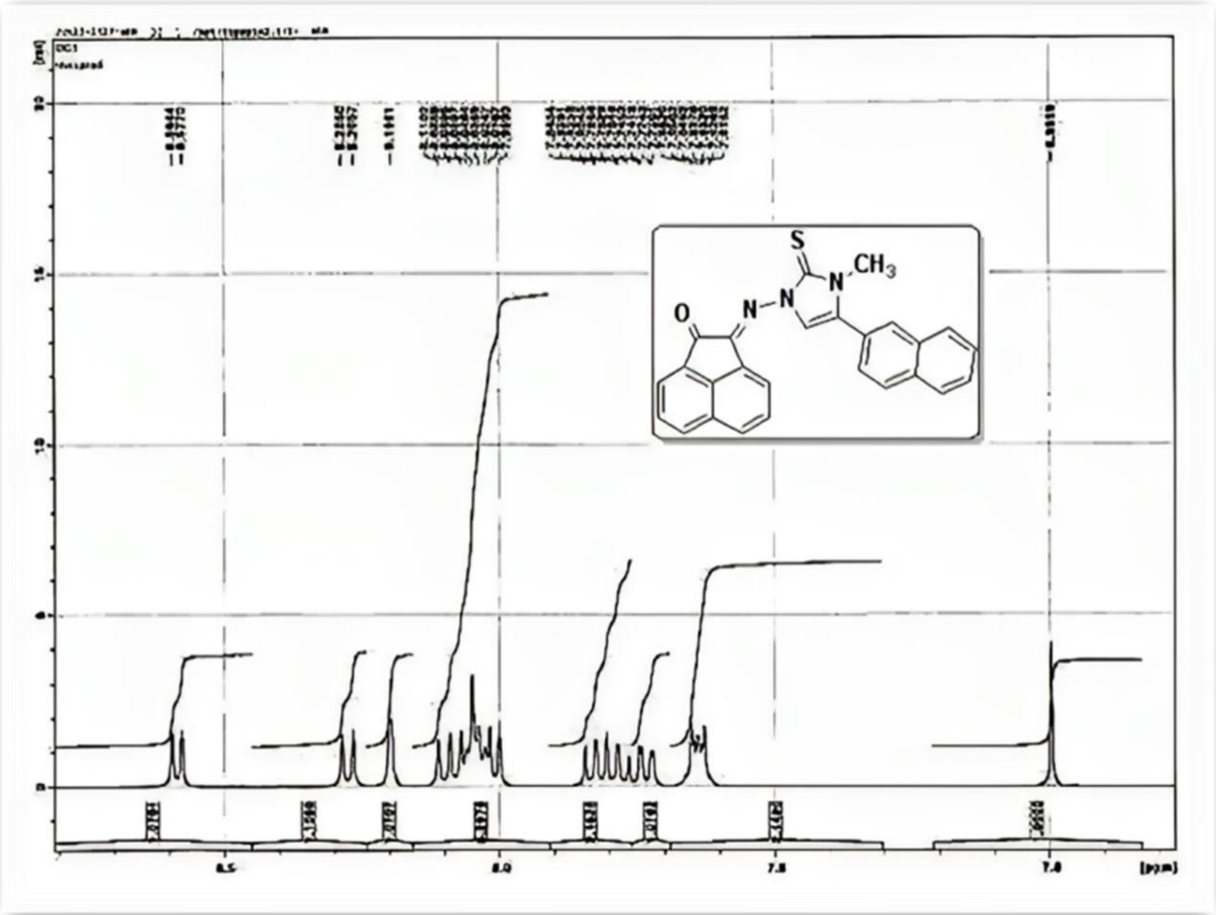

SI Figure 76. Extended ^{1}H NMR spectrum of compound **5l.**

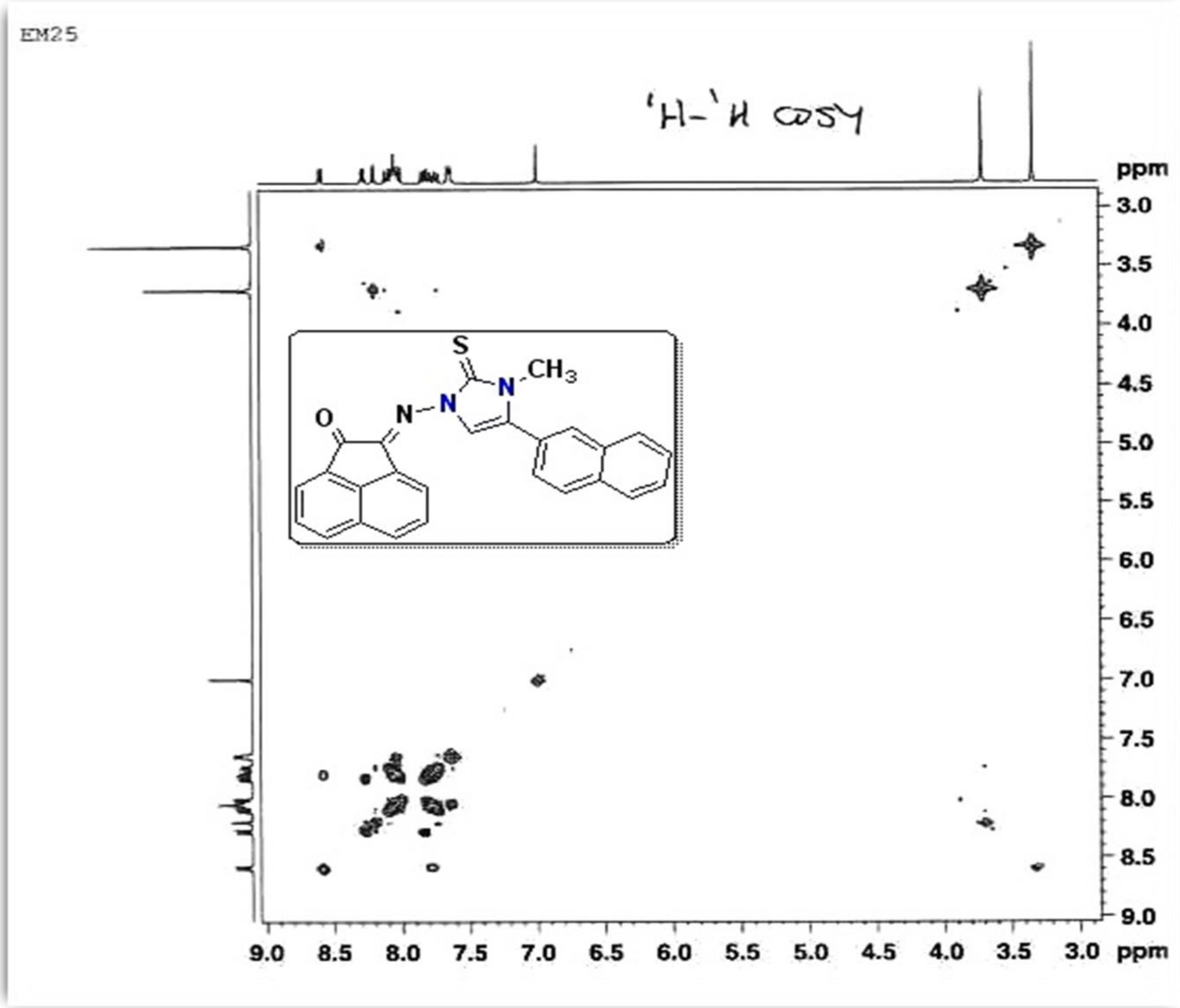

SI Figure 77. COSY H-H spectrum of compound **5l**

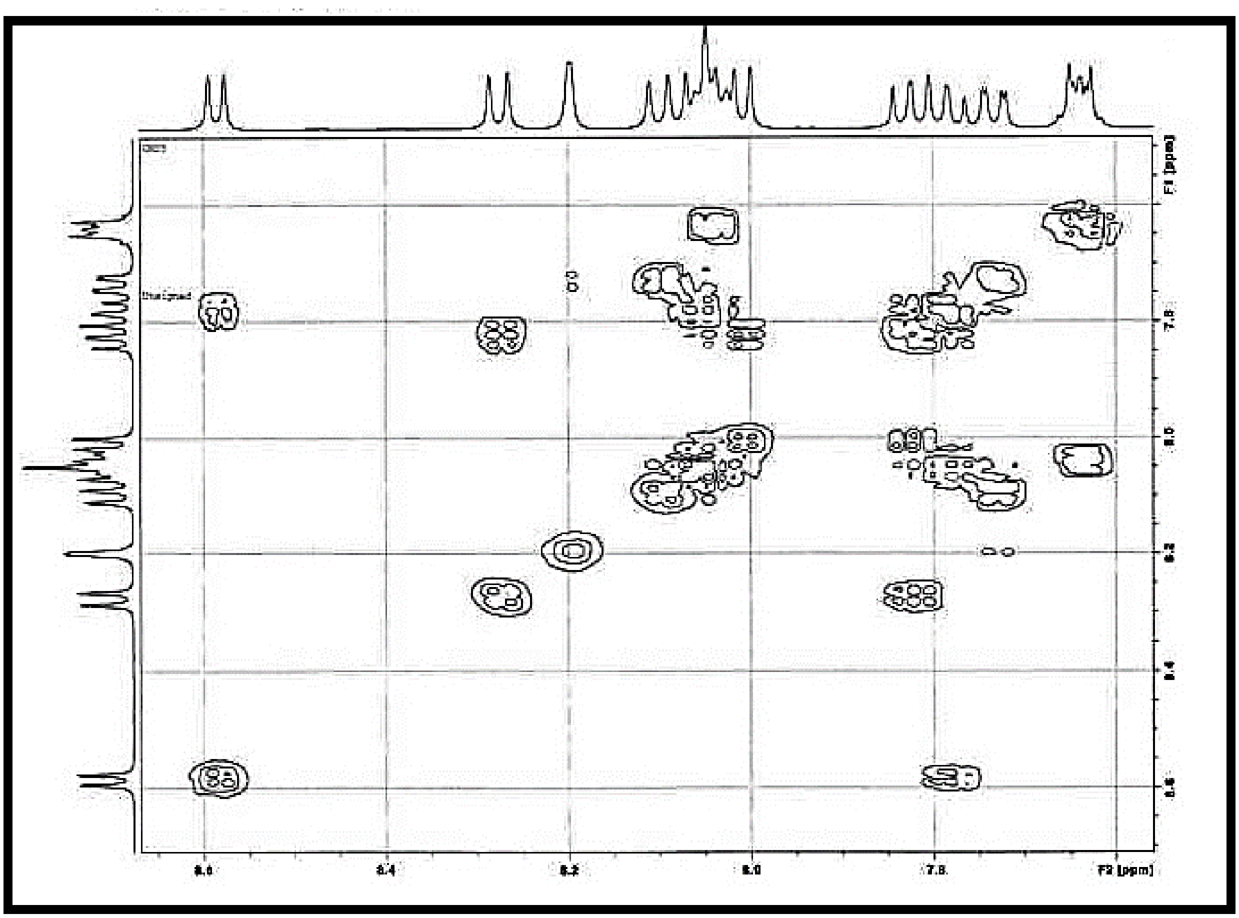

SI Figure 78. Extended COSY H-H spectrum of compound **5l**

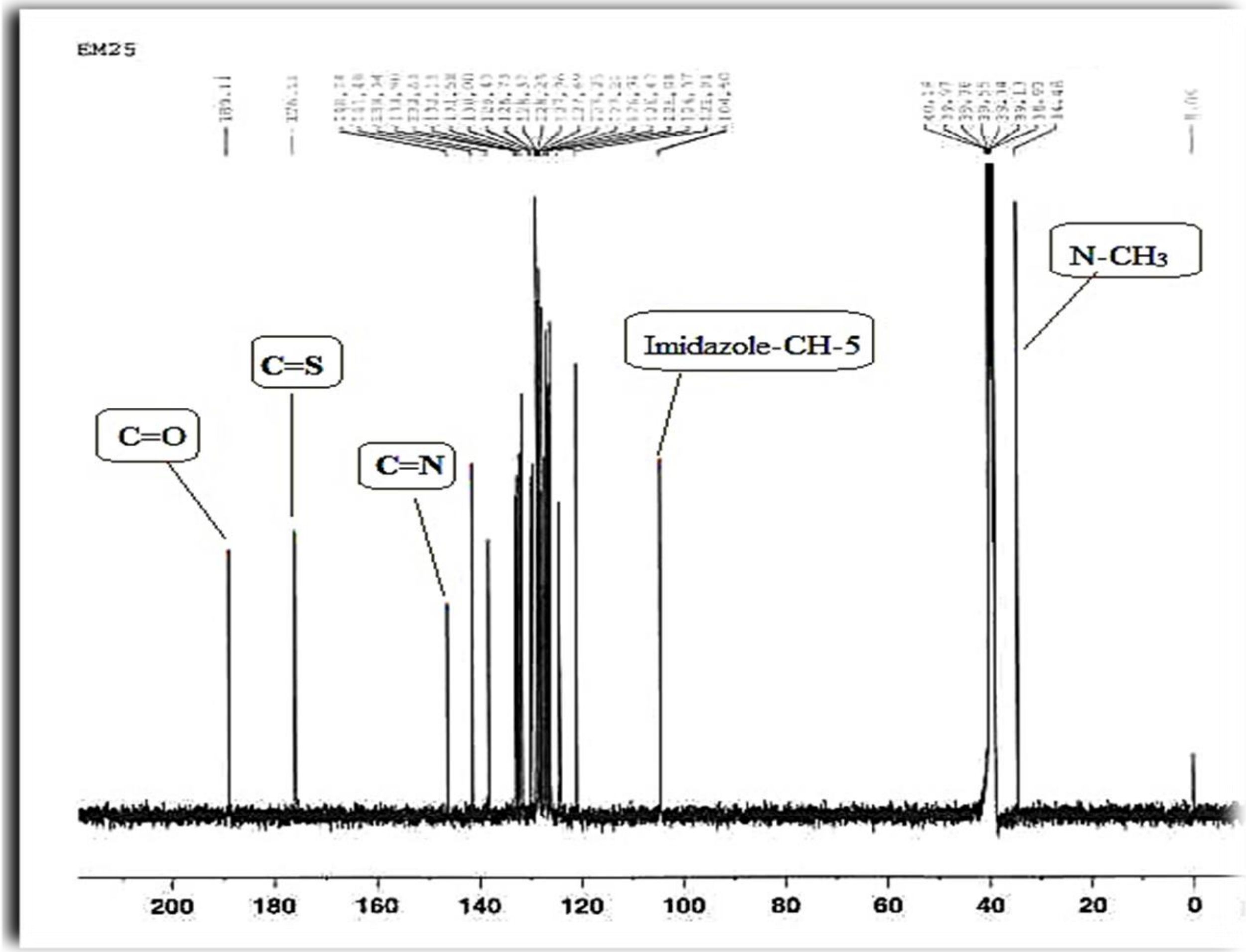

SI Figure 79.^{13}C NMR spectrum of compound **5l**

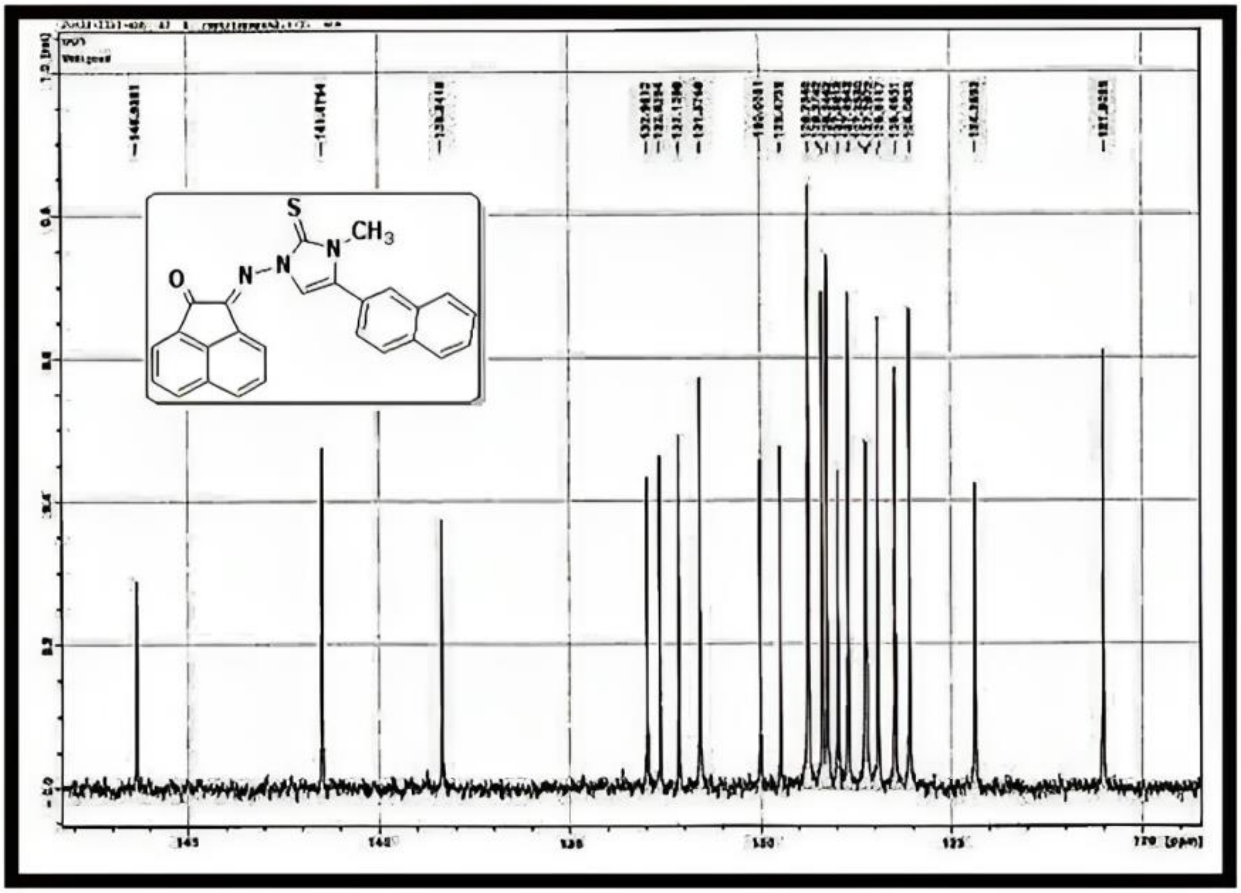

SI Figure 80. Extended ^{13}C NMR spectrum of compound **5l**

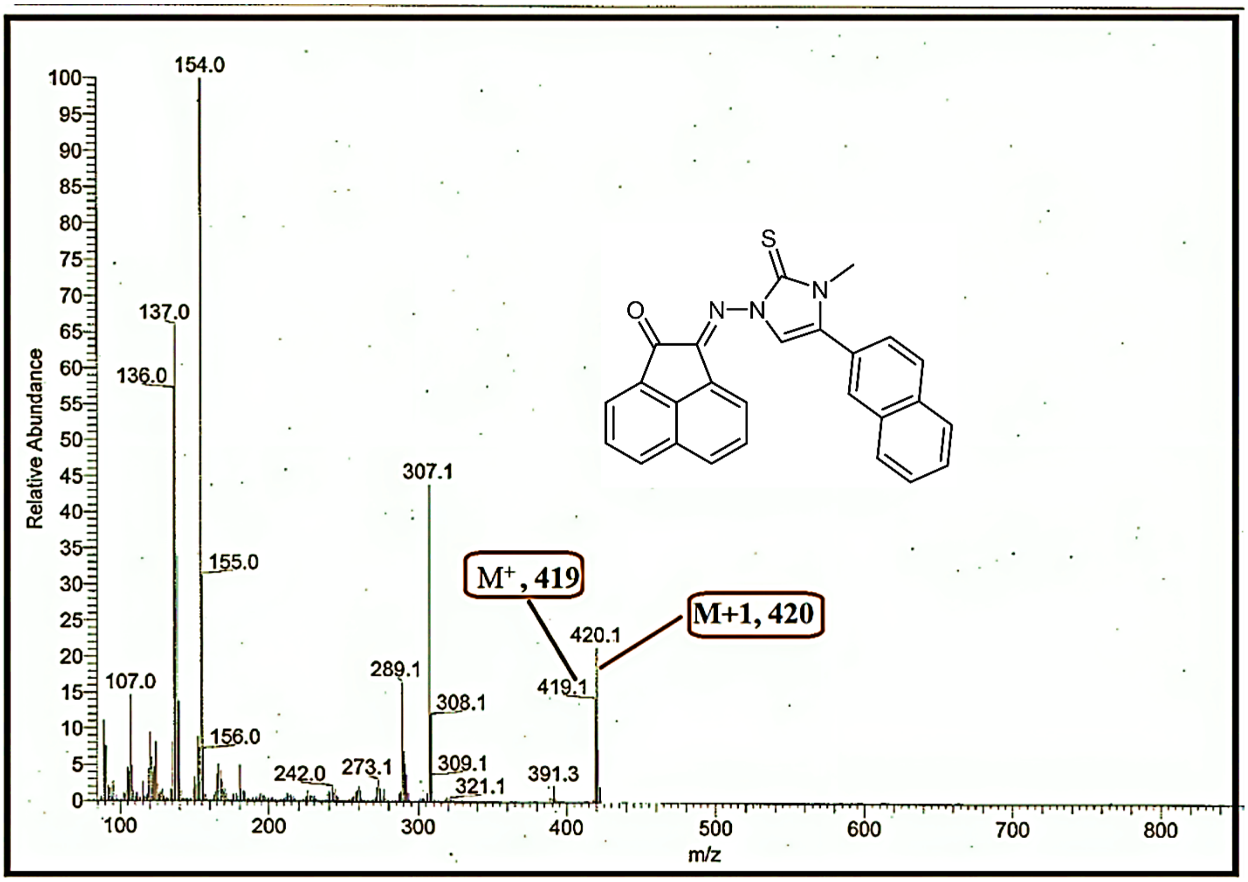

SI Figure 81. Mass spectroscopy of **5l**

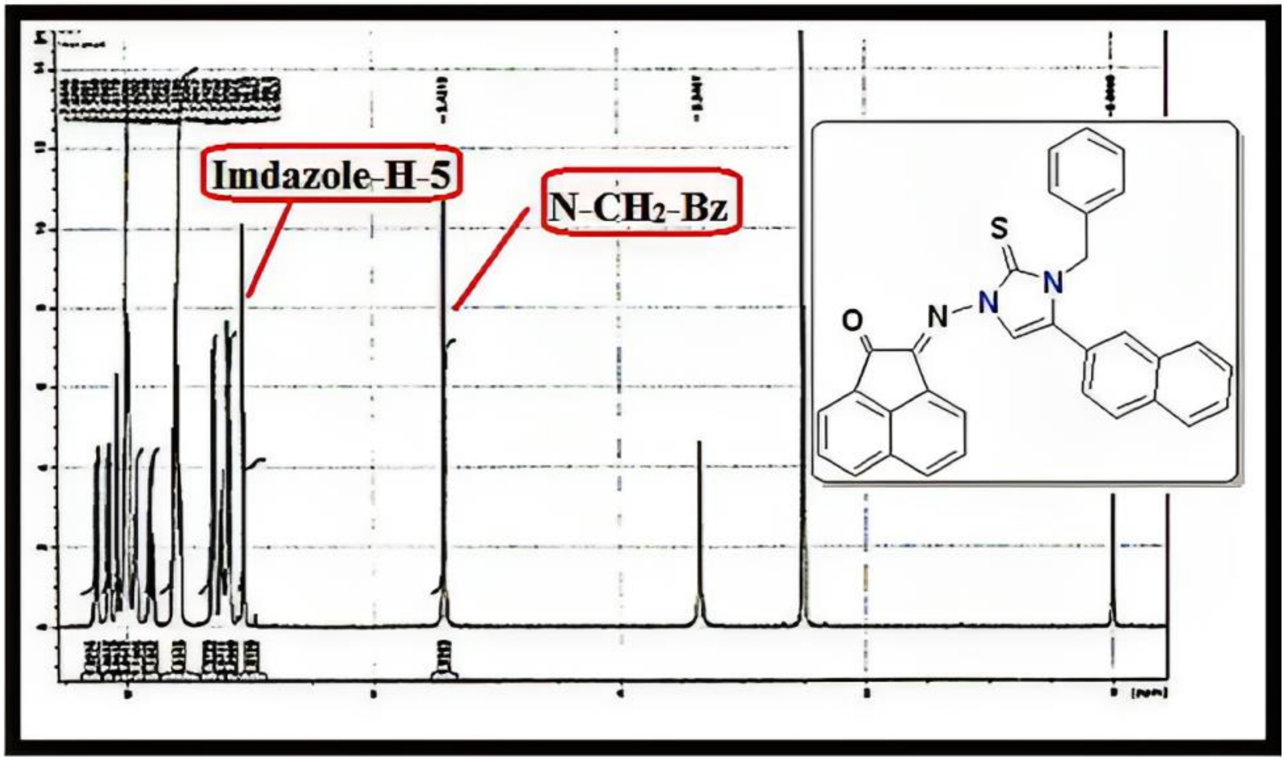

SI Figure 82. ^{1}H NMR spectrum of compound **5m.**

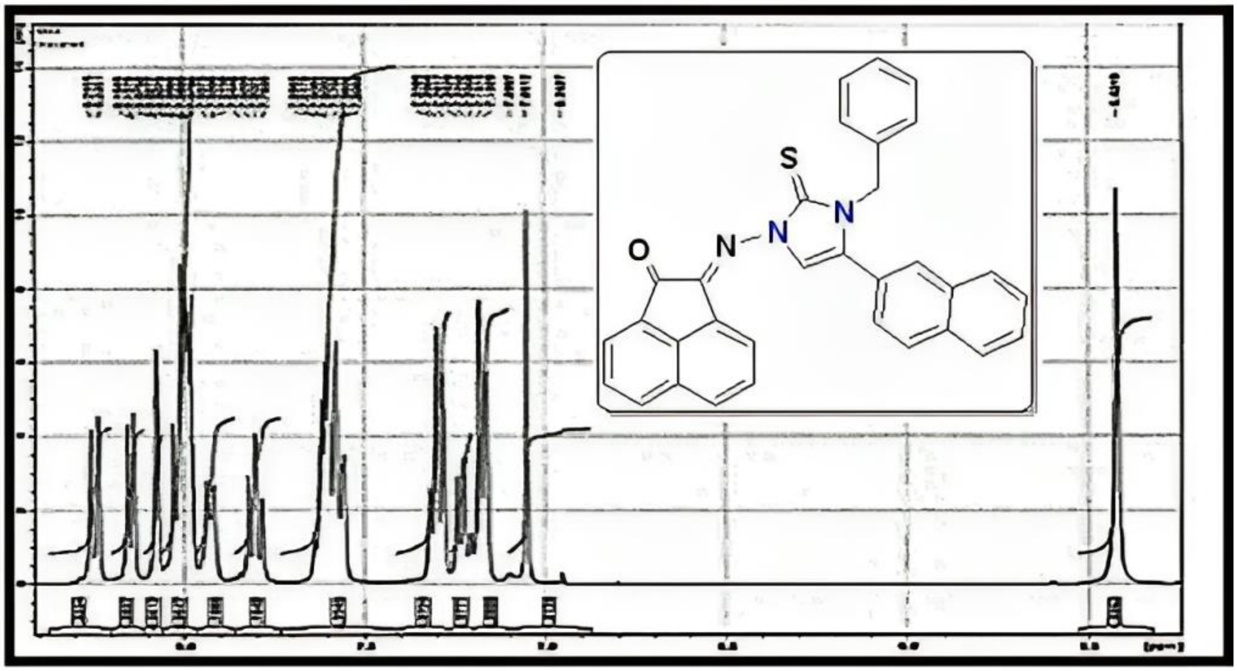

SI Figure 83. Expanded ^{1}H NMR spectrum of compound **5m**

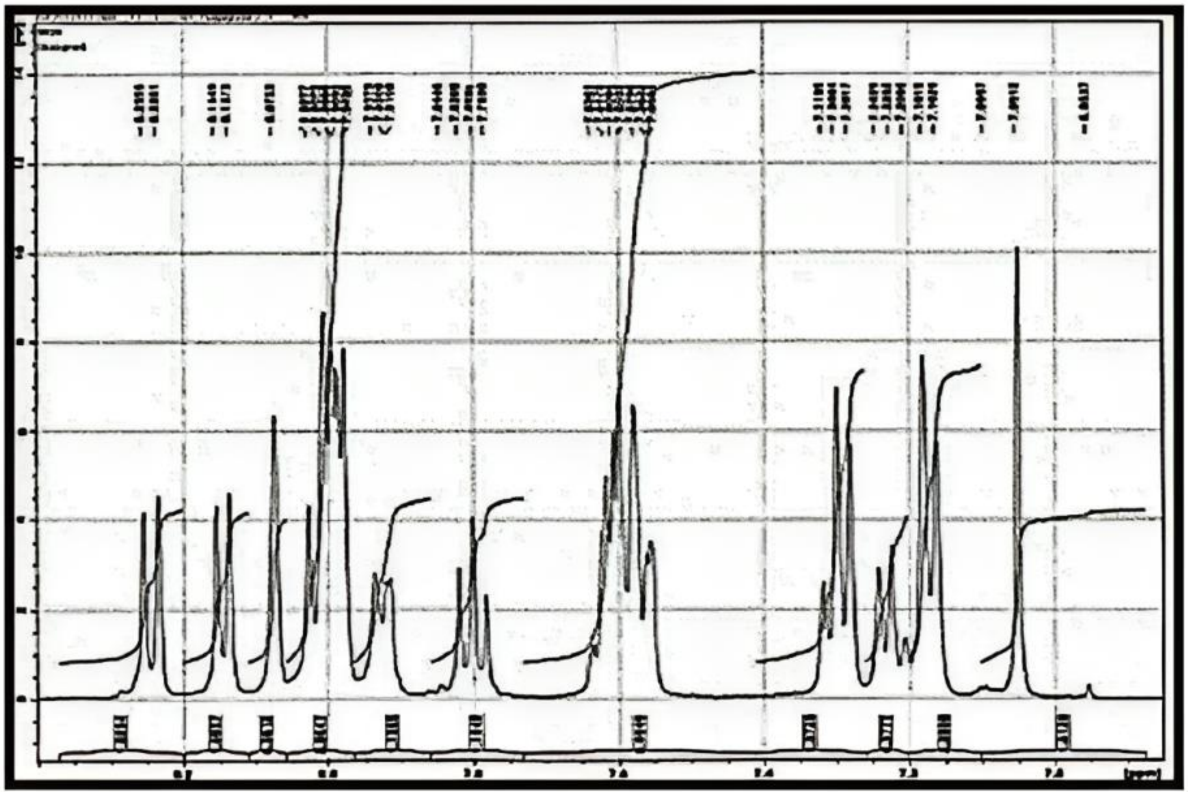

SI Figure 84. Expanded ^{1}H NMR spectrum of compound **5m**

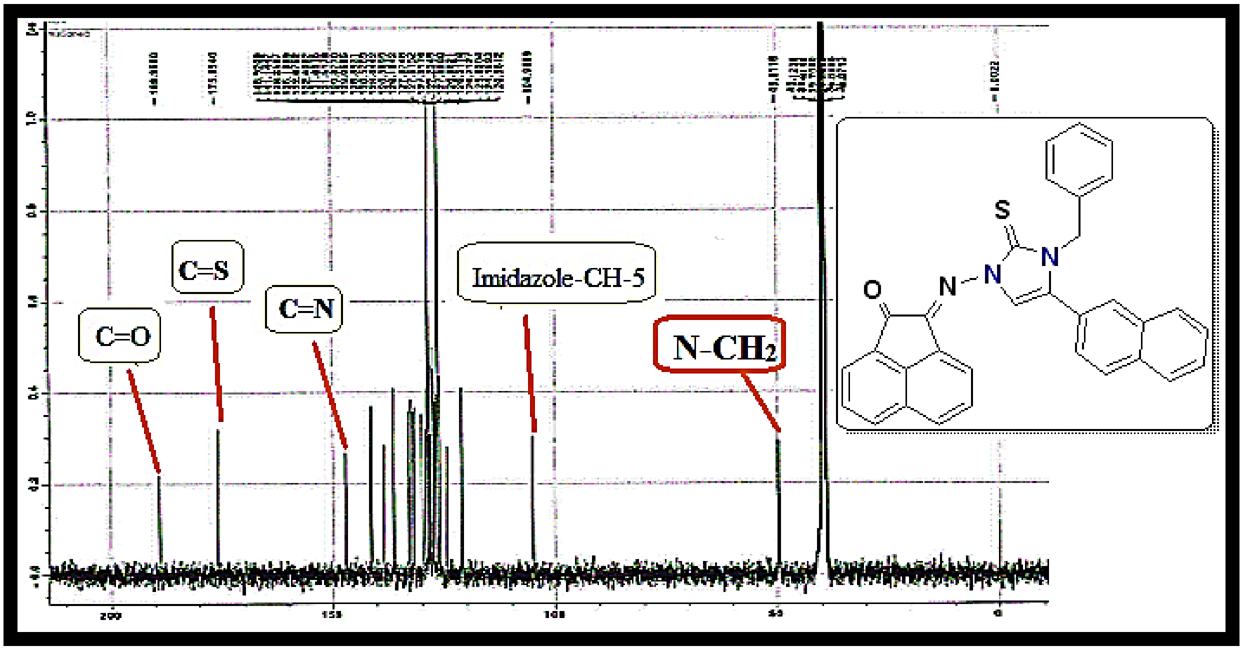

SI Figure 85. ^{13}C NMR spectrum of compound **5m**

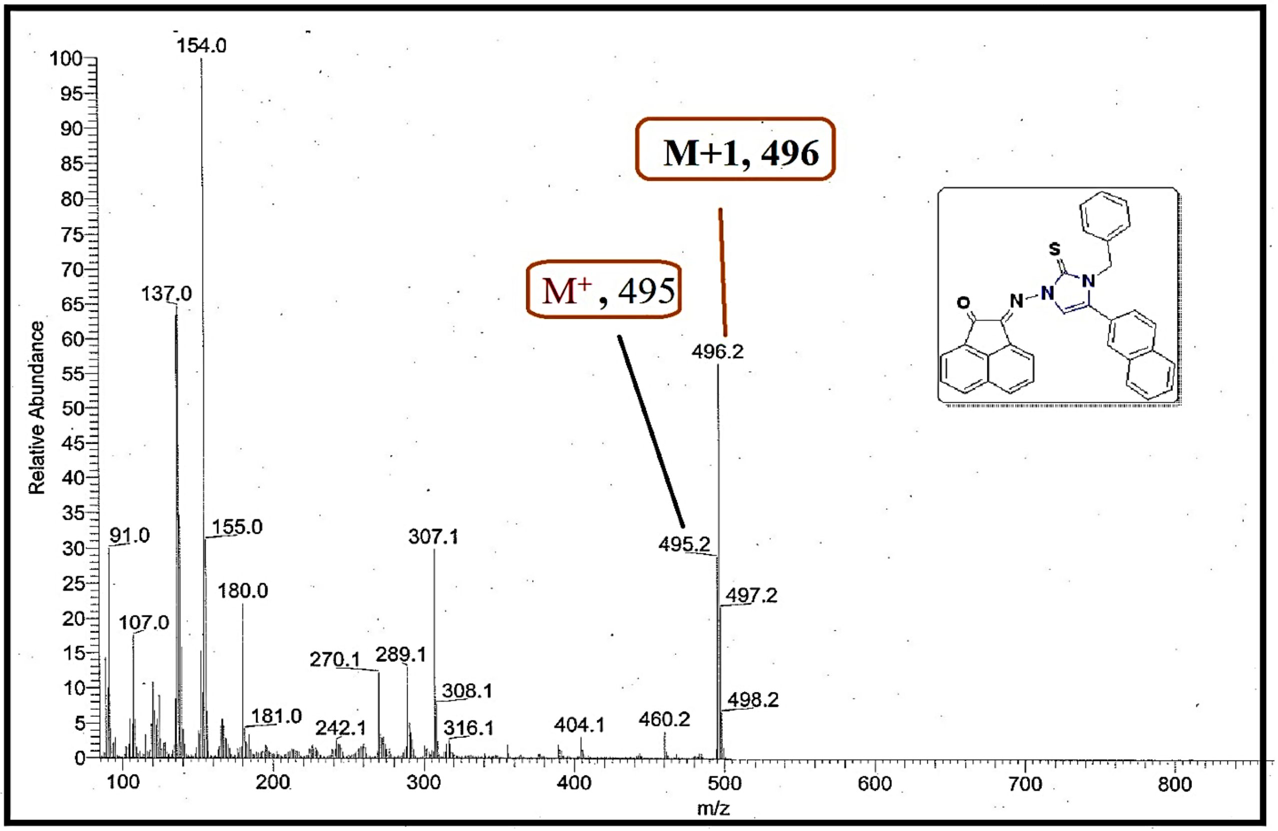

SI Figure 86. Mass spectroscopy of **5m**

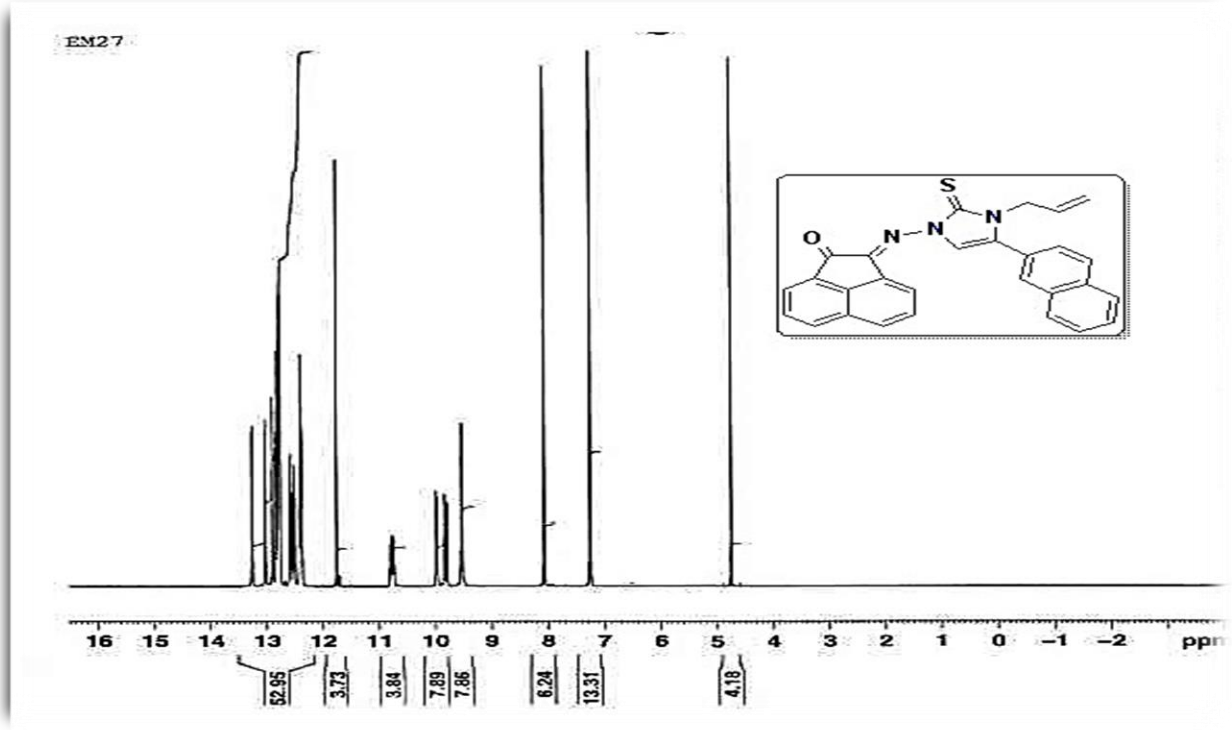

SI Figure 87.^{1}H NMR spectrum of compound **5n**

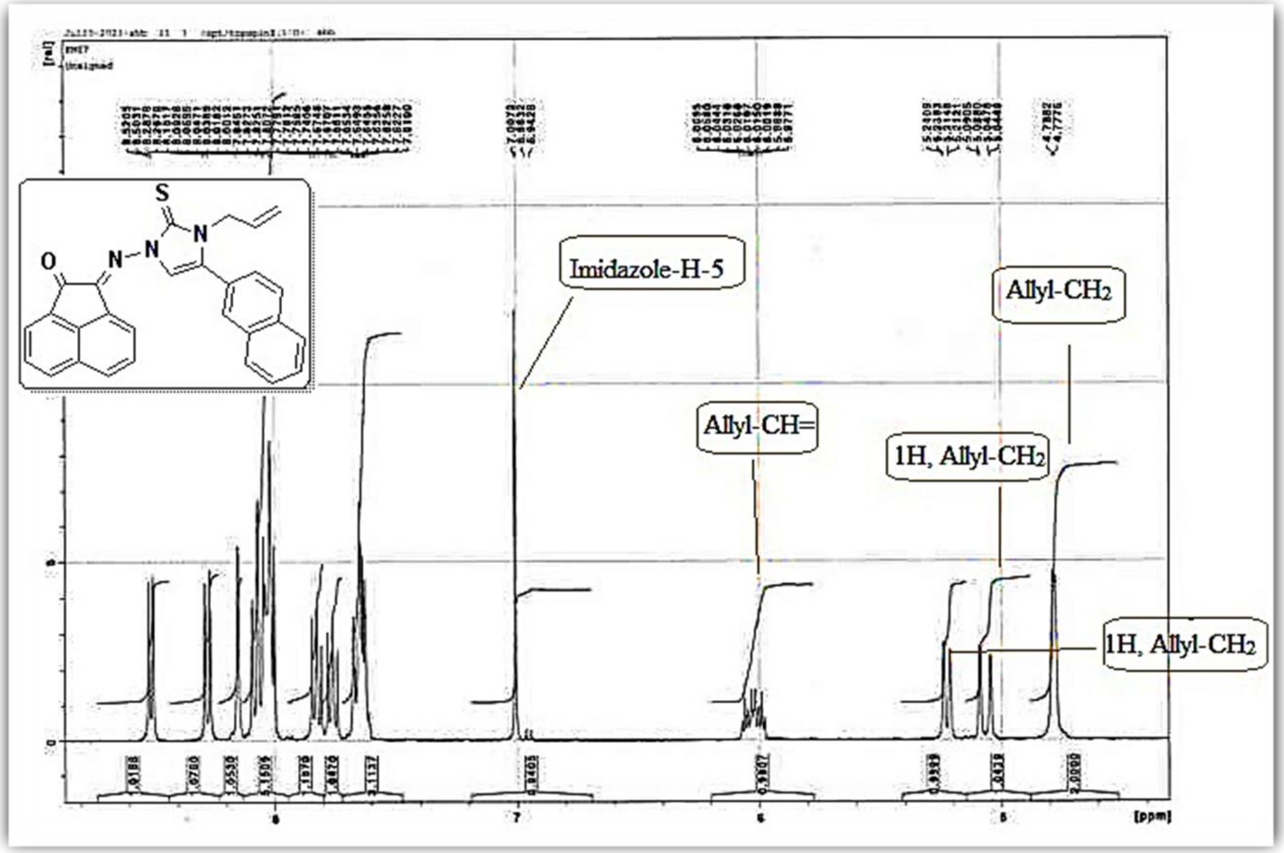

SI Figure 88. Expanded ^{1}H NMR spectrum of compound **5n**

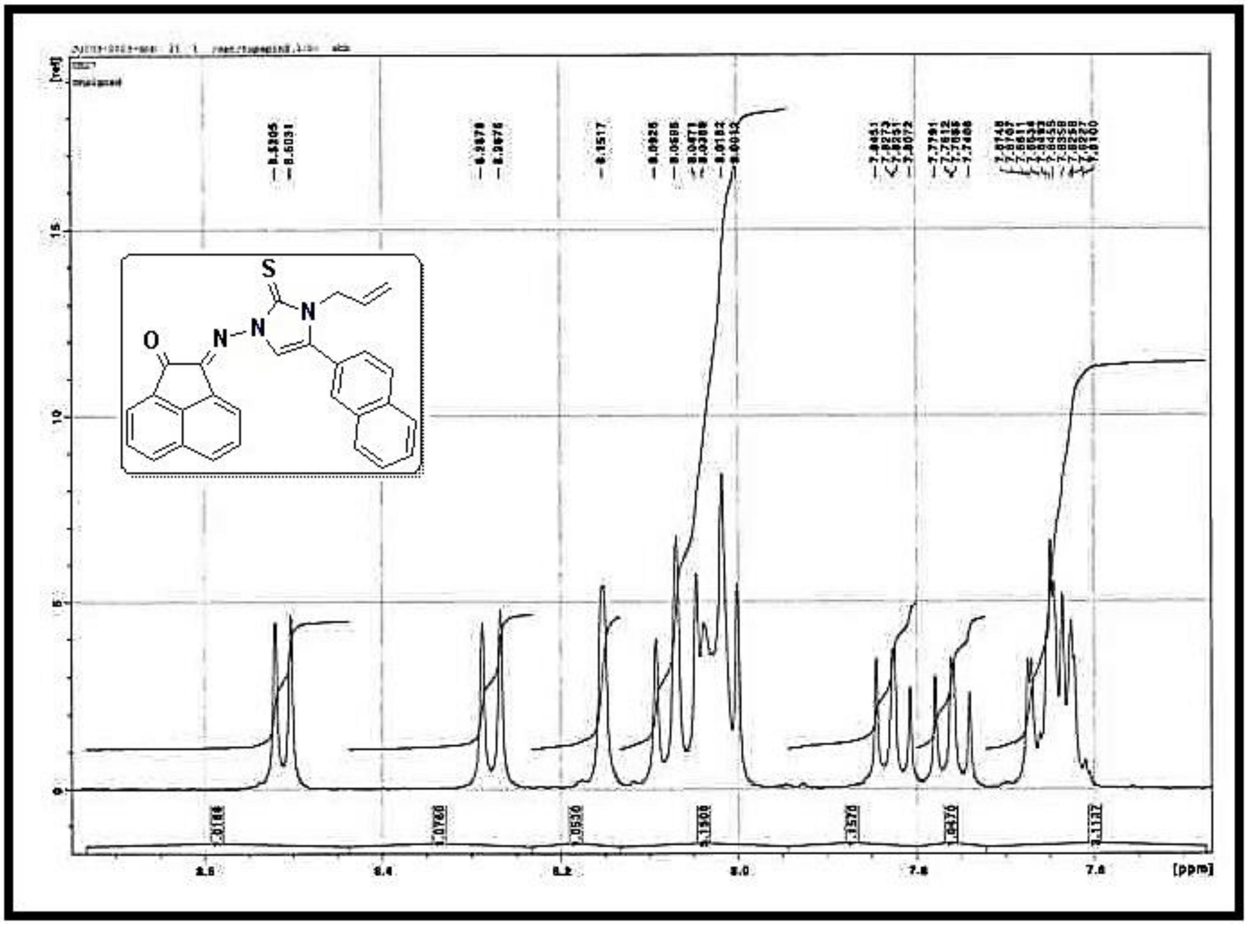

SI Figure 89. Expanded ^{1}H NMR spectrum of compound **5n**

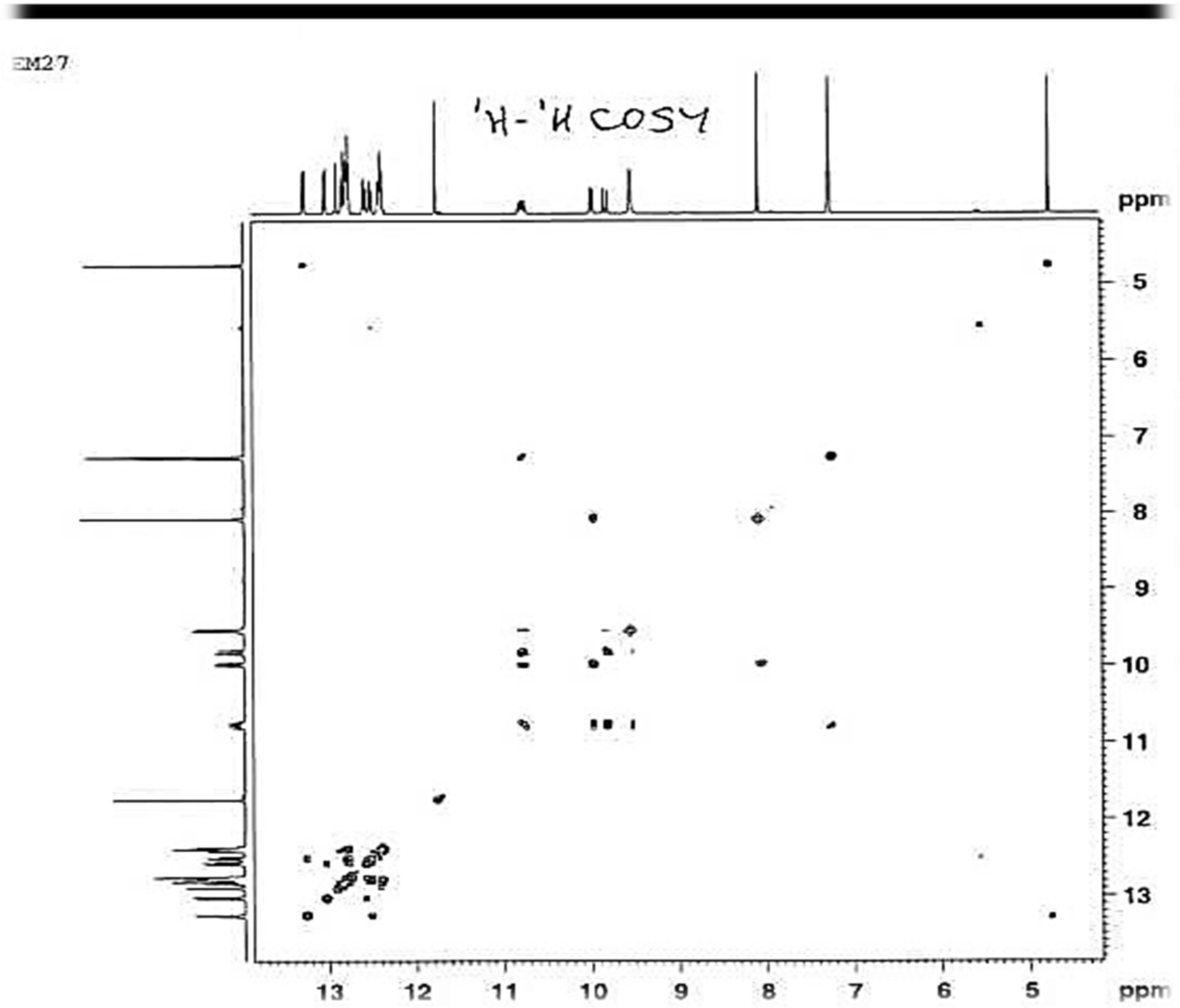

SI Figure 90. COSY H-H NMR spectrum of compound **5n**

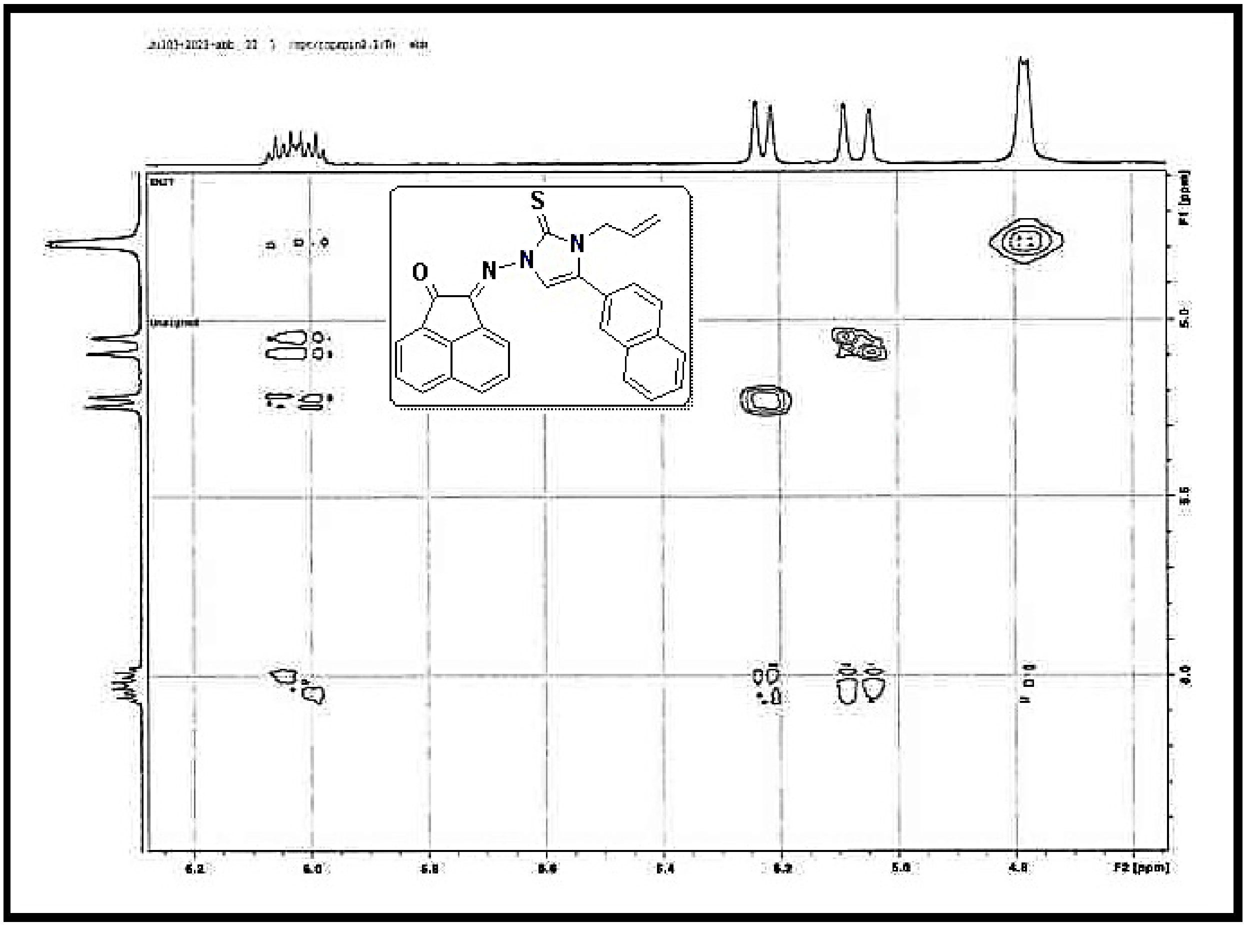

SI Figure 91. Expanded COSY H-H NMR spectrum of compound **5n**

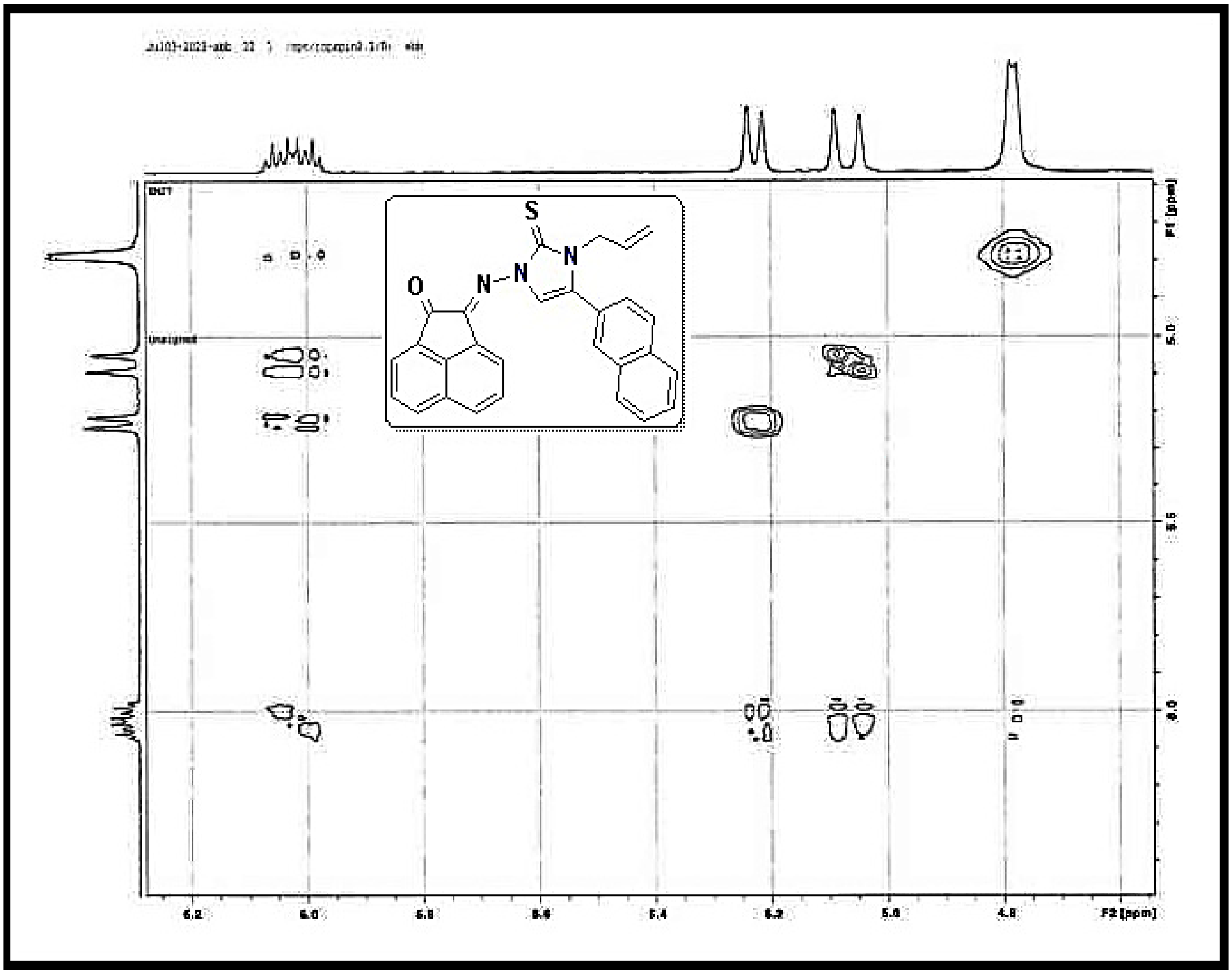

SI Figure 92. Expanded COSY H-H NMR spectrum of compound **5n**

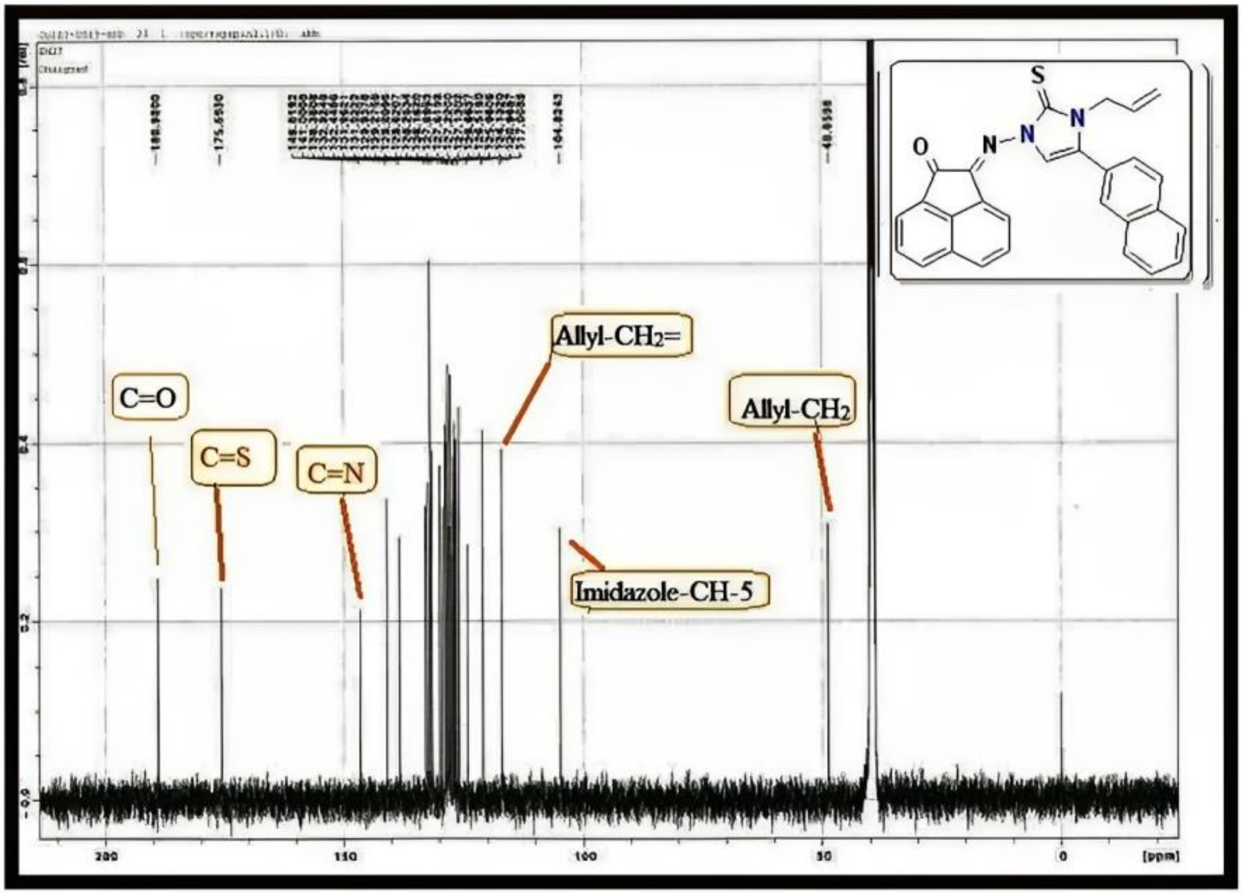

SI Figure 93. ^{13}C NMR spectrum of compound **5n**

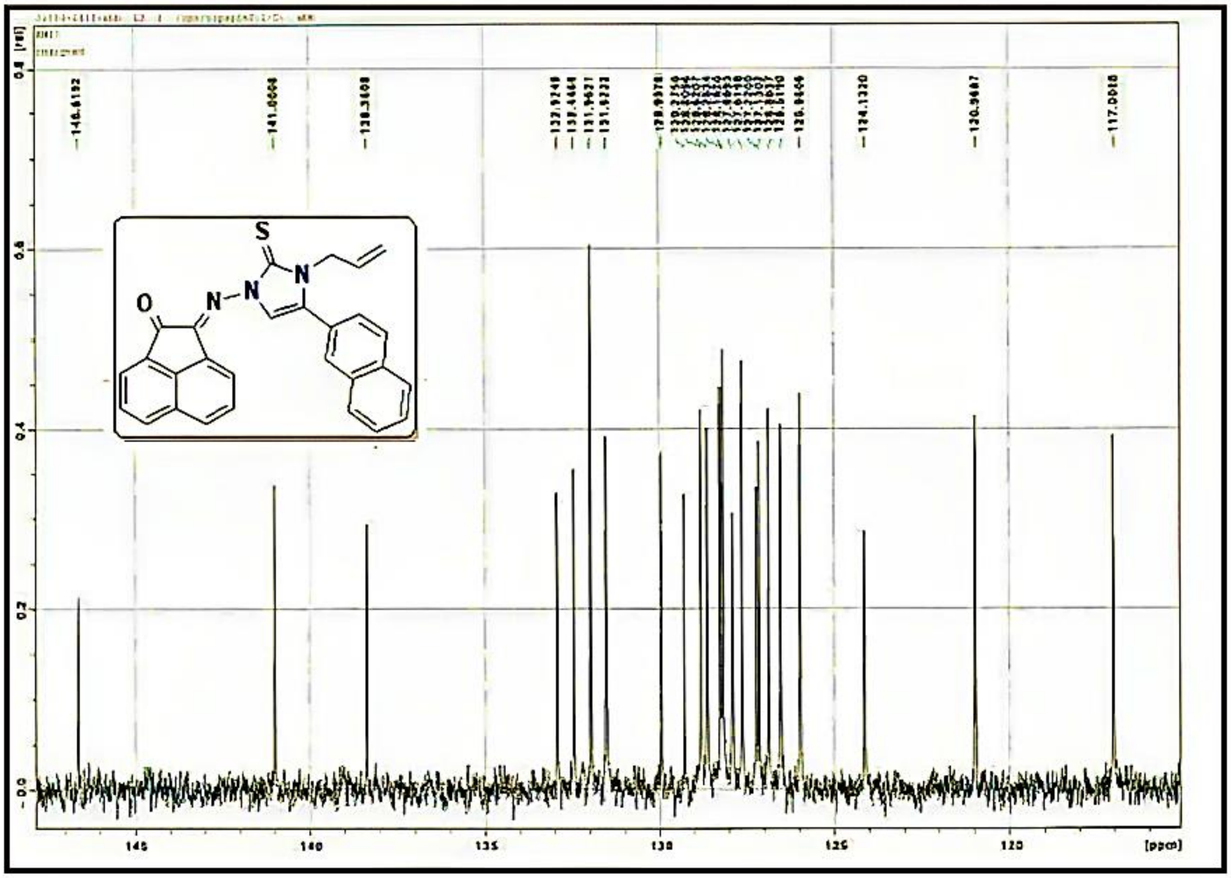

SI Figure 94. ^{13}C NMR spectrum of compound **5n**

Instrumentation

All materials were obtained from commercial suppliers and used without further purification. Reactions were monitored by TLC (Kieselgel 60 F_{254} precoated plates, E. Merck, Germany), the spots were detected by exposure to UV lamp at 254 nm. Melting points were determined on an electrothermal melting point apparatus (Stuart Scientific Co.) and were uncorrected. NMR spectra were measured in DMSO-d_6 on a Bruker AV-400 spectrometer (Bruker Bio Spin Corp., Billerica, MA, USA) (400 MHz for ^{1}H, 101 MHz for ^{13}C, and 40.15 MHz for ^{15}N) at Florida Institute of Technology, USA. The ^{1}H and ^{13}C chemical shifts are given relative to internal standard TMS = 0, and external liquid ammonia = 0 for ^{15}N. Coupling constants are stated in Hz. Correlations were established using ^{1}H-^{1}H COSY, and ^{1}H-^{13}C and ^{1}H-^{15}N HSQC and HMBC experiments. Vario EL III German CHN Elemental analyzer model was used for Elemental analysis.

Biological evaluation

All chemicals, solvents, media and kits were purchased from commercial suppliers.

DNA interaction studies

Materials

Calf thymus DNA (ctDNA) and the terbium(III) chloride were obtained from Sigma-Aldrich Canada Ltd. (Oakville, Ontario), sodium chloride was obtained from EMD Chemicals Inc. (Gibbstown, New Jersey), and Tris was obtained from ICN Biomedicals (Aurora, Ohio). All chemicals were used as received.

Fluorescence measurements[1]

The fluorescence measurements were done using PerkinElmer LS 55 fluorescence spectrophotometer.To confirm DNA damage, aliquots of each DNA-Compound mixture were mixed with $TbCl_3$ after 24 h of incubation in buffer solution (40 mM Tris, 10 mM NaCl, pH 7.5) to give final concentrations of 1 μg/mL ctDNA, 3 mM $TbCl_3$ and 50 μM of each compound in buffer solution (40 mM Tris, 10 mM NaCl, pH 7.5). For calf thymus DNA (ctDNA) experiment, ctDNA was mixed with different concentrations of each compound in the range of 0.1 pM - 200 μM. After 24 h of incubation, Tb^{3+}solution was added to the ctDNA/compound mixtures in buffer solution (40 mM Tris, 10 mM NaCl, pH 7.5) to give final concentrations of 1 μg/mL ctDNA and 3 mM Tb^{3+}solution. Fluorescence spectra were measured between 290 and 800 nm with excitation at 270 nm of 100 μL solutions in a 1 cm path length Suprasil quartz fluorescence cuvette.

***In vitro* anticancer screening**

The anticancer effect of the most active synthesized compounds **5b, 5e, 5h** and **5j** was determined against three cancer cell lines namely: hepatocellular carcinoma (HepG2), human breast adenocarcinoma (MCF-7) and human colon cancer (HCT-116) utilizing MTT assay[2]. Compounds

5b and **5h** were screened for their effects on the normal breast cell line MCF-10a. Cells at a density of 1 × 10^4 were seeded in a 96-well plate at 37°C for 48 hr under 5% CO_2. After incubation, the cells were treated with different concentrations of the prepared molecules and incubated for 24 hr. MTT dye was added after 24 h of drug treatment and incubated for 4 h at 37°C. Also, 100 µl of dimethyl sulphoxide was added to each well to dissolve the purple formazan formed. The color intensity of the formazan product, which represents the growth condition of the cells, was quantified using an enzyme-linked immunosorbent assay (ELISA) plate reader at 570 nm. The experiments were carried out with at least three replicates and they were repeated at least three times.

Table 1. *In vitro* IC_{50} values (µM) of compounds **5b**, **5e**,**5h** and **5j** against hepatocellular carcinoma (HepG-2), human breast adenocarcinoma (MCF-7), human colon cancer (HCT-116) and normal breast cell line (MCF-10a) cell lines.

**Compound ID**	**HepG2**	**MCF-7**	**HCT-116**	**MCF-10a (normal cell)**
**5b**	$7.397^{b}±0.14$	$5.105^{c}±0.15$	$17.063^{c}±0.32$	$89.759^{b}± 2.09$
**5e**	$40.47^{a}±0.79$	$22.706^{a}±0.42$	$28.989^{b}±0.54$	NT
**5h**	$4.929^{c}±0.1$	$2.65^{d}±0.06$	$43.45^{a}±0.83$	$130.576^{a}± 3.28$
**5j**	$7.964^{b}±0.17$	$9.030^{b}±0.21$	$10.847^{d}±0.24$	NT
**Dox**	$2.875^{d}±0.07$	$8.240^{b}±0.22$	$4.61^{e}±0.16$	$32.416^{c}±0.95$
**F**	**1779.3451**	**1027.2661**	**1023.7563**	**455.1012**
**p**	$<0.001^{*}$	$<0.001^{*}$	$<0.001^{*}$	$<0.001^{*}$

NT; not tested

F: **F for One way ANOVA test**, Pairwise comparison bet. each 2 groups was done using **Post Hoc Test (Tukey)**
p: p value for comparing between the studied groups
*: Statistically significant at $p \leq 0.05$
Means with **any Common letter** $^{(a-e)}$ are not significant (**OR** Means with **totally Different letters** $^{(a-e)}$ are significant)

Annexin V-FITC/PI Apoptosis Induction Analysis [3]

Apoptosis detection in MCf-7 cells was assessed using BioVision® annexin-V-FITC apoptosis detection kit according to the manufacturer's instructions and quantified by flow cytometry at 488

nm using FITC signal detector (usually FL1) and PI staining by the phycoerythrin emission signal detector (usually FL2). Briefly, 1–5 × 10^5 cells were collected by centrifugation. Cells were treated with compounds **5b** and **5h** at their IC_{50} concentrations for 24 h and resuspended in 500 µl of binding buffer. Annexin-V-FITC and PI were added. They were then incubated at room temperature for 5 min in the dark. Annexin-V-FITC binding was analyzed using a specific signal detector.

Table 2a: Comparison between the different studied groups according to apoptosis results

Apoptosis results	5h	5b	Dox	Control	F	p
**Total**	$52.5^a \pm 0.68$	$46.6^b \pm 0.57$	$46.2^b \pm 0.53$	$2.3^c \pm 0.39$	1780.305^*	$<0.001^*$
**Early**	$16.5^a \pm 0.64$	$15.3^a \pm 0.50$	$8.3^b \pm 0.53$	$0.37^c \pm 0.11$	229.642^*	$<0.001^*$
**Late**	$28.4^b \pm 0.62$	$22.7^c \pm 0.43$	$31.3^a \pm 0.62$	$0.26^d \pm 0.06$	835.679^*	$<0.001^*$
**Necrosis**	$7.5^a \pm 0.68$	$8.7^a \pm 0.44$	$6.6^a \pm 0.61$	$1.6^b \pm 0.44$	31.542^*	$<0.001^*$

3 Replica for each group **Data was expressed using Mean ± SEM.**

SEM: Standard error of mean

F: **F for One way ANOVA test**, Pairwise comparison bet. each 2 groups was done using **Post Hoc Test (Tukey)**

p: p value for comparing between the studied groups

*: Statistically significant at $p \le 0.05$

Means with **any Common letter** $^{(a\text{-}d)}$ are not significant (**OR** Means with **totally Different letters** $^{(a\text{-}d)}$ are significant)

Table 2b: Comparison between the different studied groups according to apoptosis results

Apoptosis results	5h	5b	Dox	Control	F	p
**Total**	52.5 ± 0.68	46.6 ± 0.57	46.2 ± 0.53	2.3 ± 0.39	1780.305^*	$<0.001^*$
$p_{control}$	$<0.001^*$	$<0.001^*$	$<0.001^*$			
**Sig. bet. grps.**	$p_1<0.001^*, p_2<0.001^*, p_3=0.960$					
**Early**	16.5 ± 0.64	15.3 ± 0.50	8.3 ± 0.53	0.37 ± 0.11	229.642^*	$<0.001^*$
$p_{control}$	$<0.001^*$	$<0.001^*$	$<0.001^*$			
**Sig. bet. grps.**	$p_1=0.321, p_2<0.001^*, p_3<0.001^*$					
**Late**	28.4 ± 0.62	22.7 ± 0.43	31.3 ± 0.62	0.26 ± 0.06	835.679^*	$<0.001^*$
$p_{control}$	$<0.001^*$	$<0.001^*$	$<0.001^*$			
**Sig. bet. grps.**	$p_1<0.001^*, p_2=0.013^*, p_3<0.001^*$					

**Necrosis**	7.5 ± 0.68	8.7 ± 0.44	6.6 ± 0.61	1.6 ± 0.44	31.542*	<0.001*
$p_{control}$	<0.001*	<0.001*	0.001*			
**Sig. bet. grps.**	p_1=0.435,p_2=0.657,p_3=0.093					

3 Replica for each group **Data was expressed using Mean ± SEM.**

SEM: Standard error of mean

F: **F for One way ANOVA test**, Pairwise comparison bet. each 2 groups was done using **Post Hoc Test (Tukey)**

p: p value for comparing between the studied groups

p_0: p value for comparing between **Control** and each other group

p_1: p value for comparing between **5h** and **5b**

p_2: p value for comparing between **5h** and **Dox**

p_3: p value for comparing between **5b** and **Dox**

*: Statistically significant at $p \le 0.05$

Cell Cycle Analysis [4, 5]

DNA content in the cell cycle analysis was quantified with the help ofa FACS Calibur flow cytometer at 488 nm according to the manufacturer's instructions. Briefly, 2×10^5 cells/well were treated with selected molecules **5b** and **5h** at their IC_{50} concentrations for 24 hr. After treatment, cells were washed two times and resuspended in ice-cold phosphate buffer saline PBS. After washing, 0.7 ml absolute ethanol was then added, followed by incubation at −20°C for 20 min. After washing, 500 µl RNase was added, followed by incubation for 30 min. PI was then added, followed by incubation for 30 min (light was avoided).

Table 3a: **Comparison between the different studied groups according to cell cycle arrest**

Cell cycle arrest (%)	5h	5b	Dox	Control	F	p
**G0-G1**	$48.6^d \pm 0.73$	$52.7^c \pm 0.30$	$63.5^a \pm 0.51$	$56.9^b \pm 0.66$	121.899*	<0.001*
**G2 /M**	$9.7^b \pm 0.36$	$13.4^a \pm 0.41$	$2.4^c \pm 0.73$	$15.6^a \pm 0.39$	138.810*	<0.001*
**S**	$41.7^a \pm 0.39$	$33.8^b \pm 0.70$	$34.2^b \pm 0.22$	$27.5^c \pm 0.28$	174.250*	<0.001*

3 Replica for each group **Data was expressed using Mean ± SEM.**

SEM: Standard error of mean

F: **F for One way ANOVA test**, Pairwise comparison bet. each 2 groups was done using **Post Hoc Test (Tukey)**

p: p value for comparing between the studied groups

*: Statistically significant at $p \le 0.05$

Means with **any Common letter** $^{(a-d)}$ are not significant (**OR** Means with **totally Different letters** $^{(a-d)}$ are significant)

Table 3b: Comparison between the different studied groups according to cell cycle arrest

Cell cycle arrest (%)	5h	5b	Dox	Control	F	p
**G0-G1**	48.6 ± 0.73	52.7 ± 0.30	63.5 ± 0.51	56.9 ± 0.66	121.899*	<0.001*
$p_{control}$	<0.001*	0.004*	<0.001*			
**Sig. bet. grps.**	p_1=0.004*,p_2<0.001*,p_3<0.001*					
**G2 /M**	9.7 ± 0.36	13.4 ± 0.41	2.4 ± 0.73	15.6 ± 0.39	138.810*	<0.001*
$p_{control}$	<0.001*	0.056	<0.001*			
**Sig. bet. grps.**	p_1=0.003*,p_2<0.001*,p_3<0.001*					
**S**	41.7 ± 0.39	33.8 ± 0.70	34.2 ± 0.22	27.5 ± 0.28	174.250*	<0.001*
$p_{control}$	<0.001*	<0.001*	<0.001*			
**Sig. bet. grps.**	p_1<0.001*,p_2<0.001*,p_3=0.949					

3 Replica for each group **Data was expressed using Mean ± SEM.**

SEM: Standard error of mean

F: **F for One way ANOVA test**, Pairwise comparison bet. each 2 groups was done using **Post Hoc Test (Tukey)**

p: p value for comparing between the studied groups

p_0: p value for comparing between **Control** and each other group

p_1: p value for comparing between **5h** and **5b**

p_2: p value for comparing between **5h** and **Dox**

p_3: p value for comparing between **5b** and **Dox**

*: Statistically significant at $p \le 0.05$

In vitro Topoisomerase II inhibitory assay[6].

The most active anti-proliferative members were analyzed for their Topo II inhibitory activities. Doxorubicin was used as a positive control. Compounds **5b** and **5h** were selected to be evaluated against topo II [MBS#942146] using human DNA topoisomerase II-β (TOPII-β) ELISA kit

according to manufacturer's instructions. Prepare all reagents, working standards, and samples. Add 100 µL of standard and sample per well and incubate for 2 h at 37°C.Remove the liquid of each well. Add 120 µL of biotin-antibody to each well and incubate for 1 h at 37°C.Aspirate each well and wash three times. Add 100 µL of horseradish Peroxidase (HRP-avidin) to each well and incubate for 1 h at 37°C. Repeat the aspiration/ wash process for five times. Add 90 µL of 3,3',5,5'-tetramethylbenzidine(TMB) substrate to each well and incubate for 15-30 min at 37°C, protect from light. Add 50 µL of stop solution to each well and determine the optical density of each well within 5 min, using a ROBONEK P2000 ELISA reader to 450 nm. The values of % activity versus a series of compound concentrations (2.5µM, 5µM, 10µM, 15µM) were then plotted using non-linear regression analysis of sigmoidal dose-response curve. The IC_{50} values for compounds **5b** and **5h** against topo II-β was determined by the concentration causing a half-maximal percent activity and the data were compared with podo as standard. All experiments were done in triplicates.

***In-silico* studies**

Molecular Docking

The crystallographic structure of Human topoisomerase II beta in complex with DNA and etoposide (PDB ID: 3QX3) was retrieved from Protein Data Bank. Computer-aided docking experiments were performed using Molecular Operating Environment (MOE 2020.09) software (Chemical Computing Group, Montreal, Canada)[7].

The database of tested compounds was prepared by addition of hydrogens, calculation of partial charges and energy minimization using Amber 10: EHT Force Field with root mean square deviation (RMSD) gradient of 0.1 kcal/mol. Furthermore, the downloaded protein was prepared by removal of repeating chains and keeping some nucleotides and water molecules which are

essential in ligand interaction. Then, for optimizing structural issues, 3D protonation and calculation of partial charges, MOE QuickPrep protocol was performed. The default protocol in the MOE Dock application was employed to find the favorable binding conformations and best scoring values of the test compounds, using triangle matcher as placement method and London dG as the main scoring function. Force field-based scoring function (GBVI/WSA dG) was used as an additional refinement step using induced fit receptor method, to select poses displaying maximal hydrogen-bond, ionic and hydrophobic interactions to the protein. The output database comprised the energy scores in kcal/mol for the complexes formed between the ligands conformers and the binding sites. Eventually, the produced docking poses were visually examined and interactions with active site residues were analyzed. The pose exhibiting the top score with the best ligand-enzyme interaction was set as default.

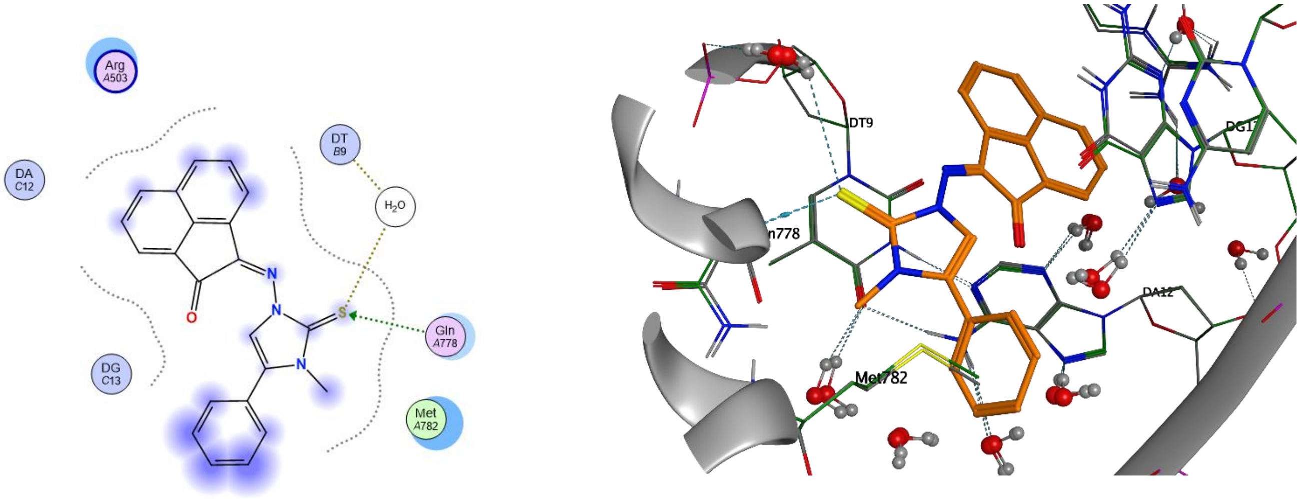

SI Figure 95. The 2D and 3D representation of binding interaction of compound **5a** at the active site of DNA-Topo IIβ (PDB ID: 3QX3)

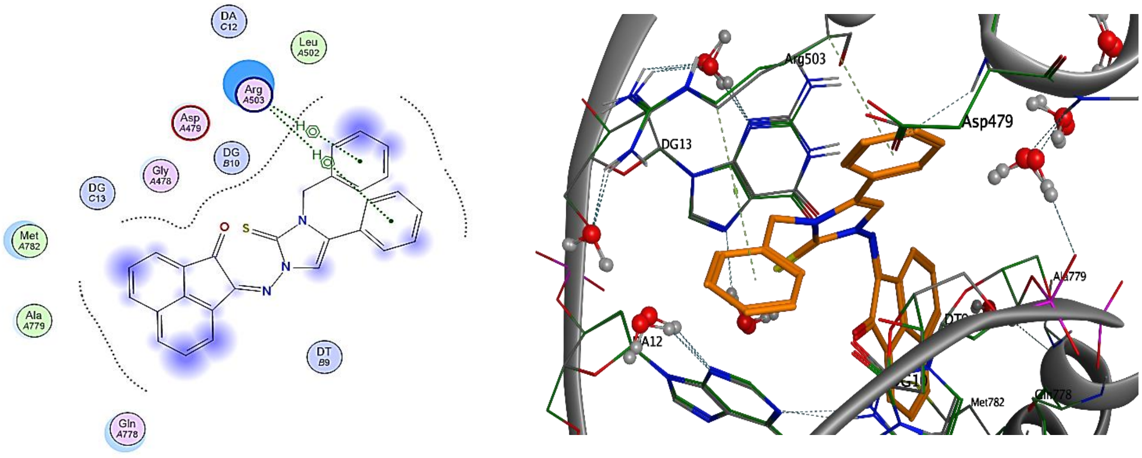

SI Figure 96. The 2D and 3D representation of binding interaction of compound **5c** at the active site of DNA-Topo IIβ (PDB ID: 3QX3)

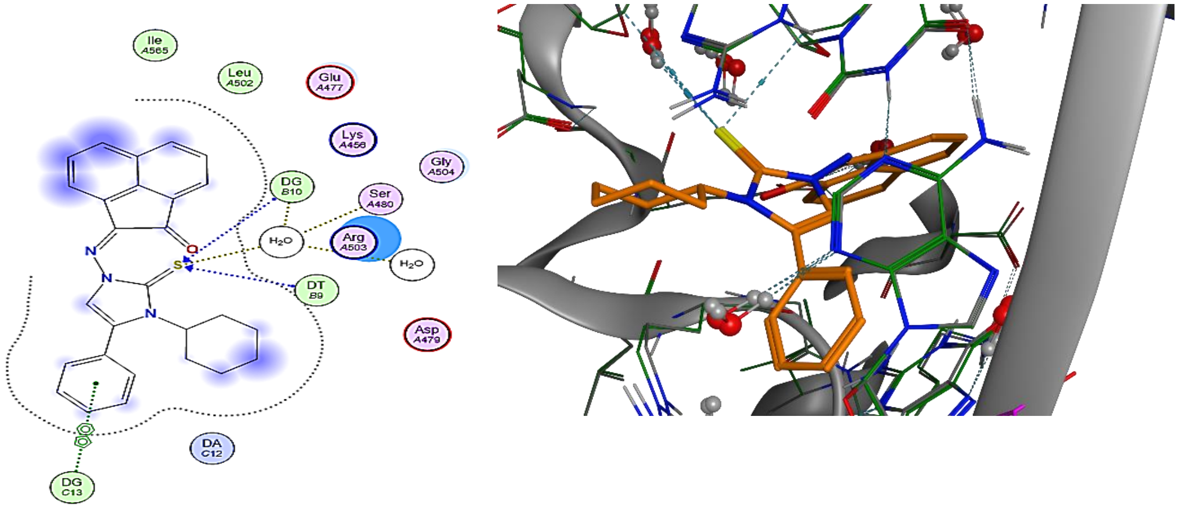

SI Figure 97. The 2D and 3D representation of binding interaction of compound **5d** at the active site of DNA-Topo IIβ (PDB ID: 3QX3)

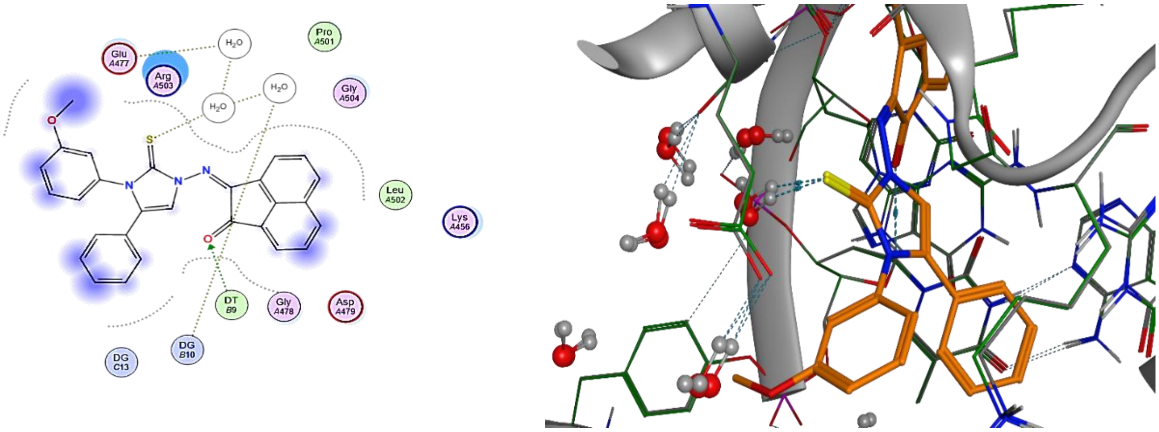

SI Figure 98. The 2D and 3D representation of binding interaction of compound **5e** at the active site of DNA-Topo IIβ (PDB ID: 3QX3)

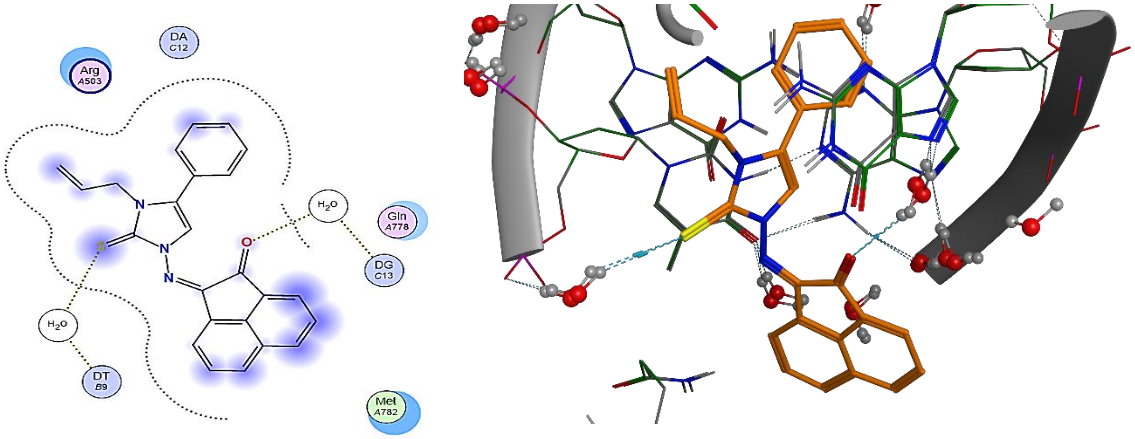

SI Figure 99. The 2D and 3D representation of binding interaction of compound **5f** at the active site of DNA-Topo IIβ (PDB ID: 3QX3)

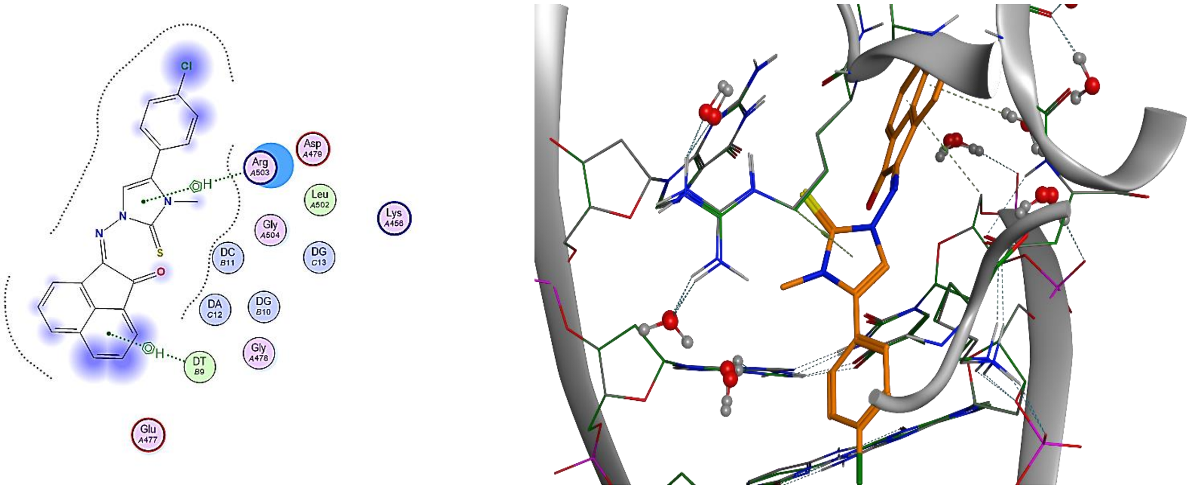

SI Figure 100. The 2D and 3D representation of binding interaction of compound **5g** at the active site of DNA-Topo IIβ (PDB ID: 3QX3)

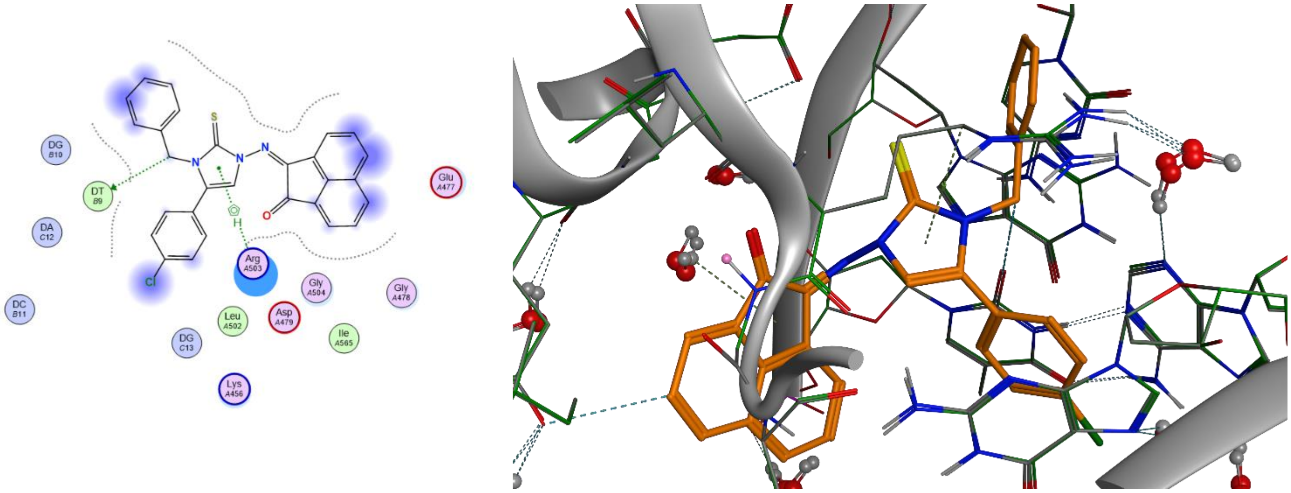

SI Figure 101. The 2D and 3D representation of binding interaction of compound **5i** at the active site of DNA-Topo IIβ (PDB ID: 3QX3)

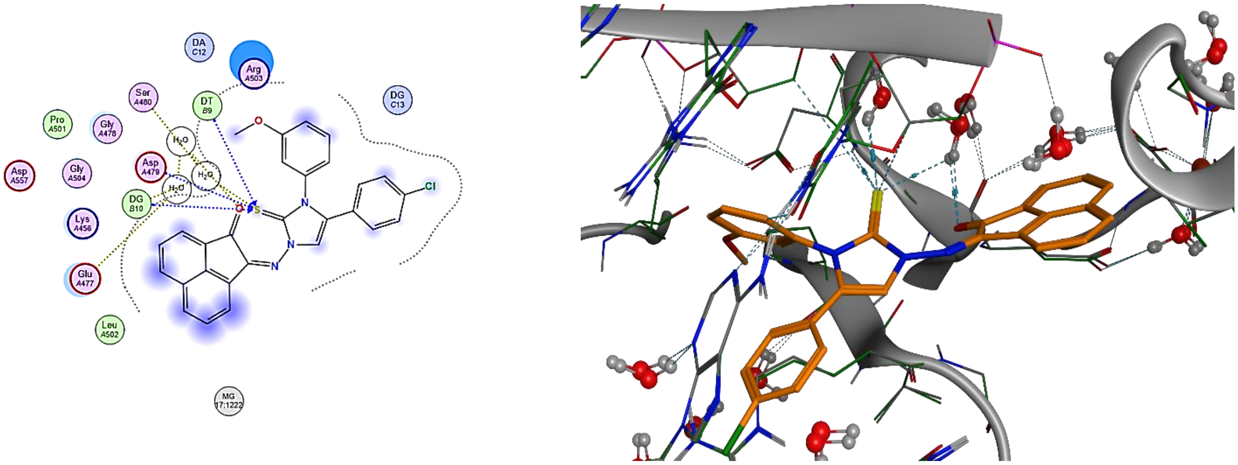

SI Figure 102. The 2D and 3D representation of binding interaction of compound **5j** at the active site of DNA-Topo IIβ (PDB ID: 3QX3)

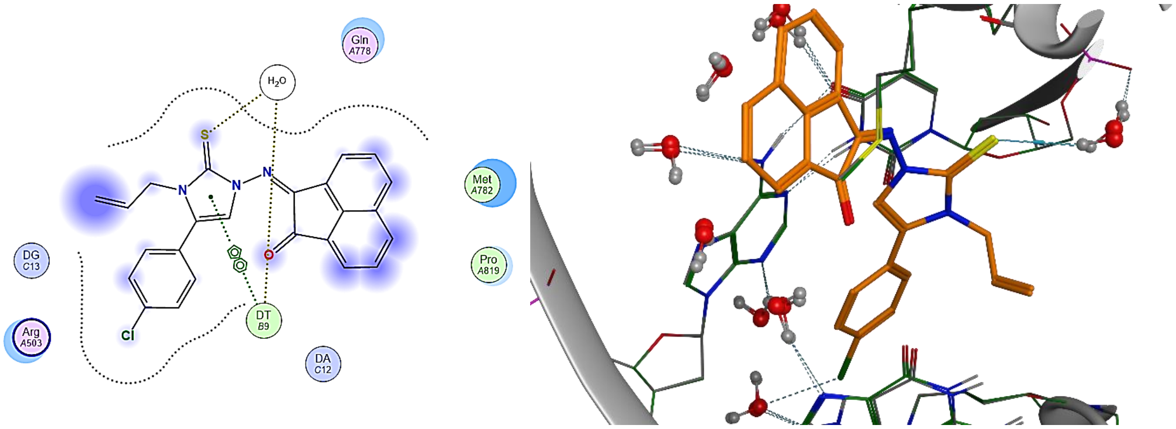

SI Figure 103. The 2D and 3D representation of binding interaction of compound **5k** at the active site of DNA-Topo IIβ (PDB ID: 3QX3)

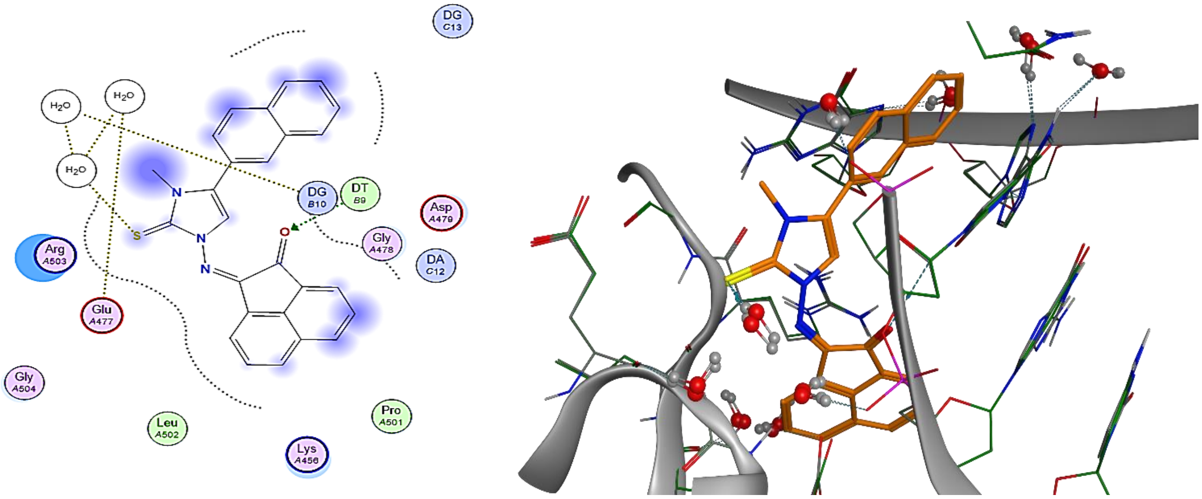

SI Figure 104. The 2D and 3D representation of binding interaction of compound **5l** at the active site of DNA-Topo IIβ (PDB ID: 3QX3)

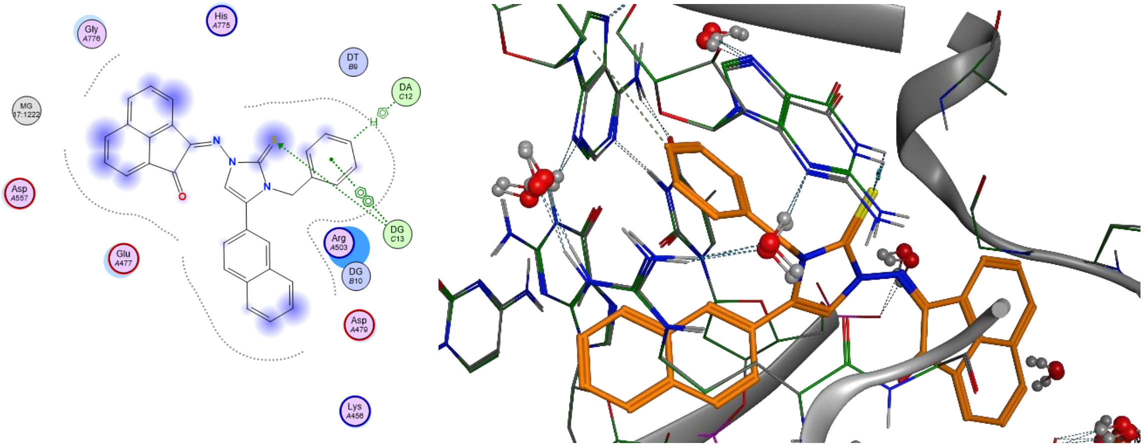

SI Figure 105. The 2D and 3D representation of binding interaction of compound **5m** at the active site of DNA-Topo IIβ (PDB ID: 3QX3)

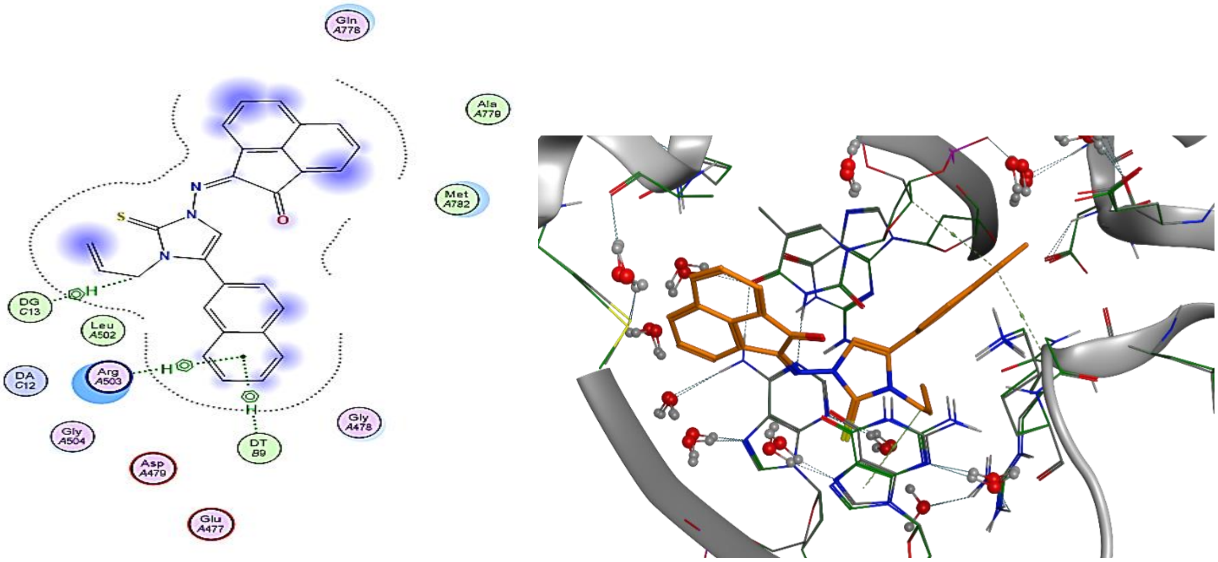

SI Figure 106. The 2D and 3D representation of binding interaction of compound **5n** at the active site of DNA-Topo IIβ (PDB ID: 3QX3)

References

[1] El-Yazbi AF ,Wong A, Loppnow GR. A luminescent probe of mismatched DNA hybridization: Location and number of mismatches. Anal ChimActa. 2017;994:92-99.

[2] Mosmann T. Rapid colorimetricassay for cellular growth and survival: Application to proliferation and cytotoxicity assays. J lmmunol Methods. 1983;65:55-63.

[3] Riccardi C, Nicoletti I. Analysis of apoptosis by propidium iodide staining and flow cytometry. Nat Protoc. 2006;1(3):1458-1461.

[4] Zaki I, Abdelhameid MK, El-Deen IM, Abdel Wahab AHA, Ashmawy AM, Mohamed KO. Design, synthesis and screening of 1, 2, 4-triazinone derivatives as potential antitumor agents with apoptosis inducing activity on MCF-7 breast cancer cell line. Eur J Med Chem. 2018;156:563-579.

[5] Zaki I, Ramadan HMM, El-Sayed EH, Abd El-Moneim M. Design, synthesis, and cytotoxicity screening of new synthesized imidazolidine-2-thiones as VEGFR-2 enzyme inhibitors. Arch Pharm (Weinheim). 2020;353(11):e2000121.

[6]https://www.mybiosource.com/top2b-human-elisa-kits/dna-topoisomerase-2-beta/763112(accessed in June 2023).

[7] Molecular operating environment (MOE), chemical computing group inc.:Montreal, qc, canada. Http://www.Chemcomp.Com (last accessed 20 June 2023).
